# Discovery of a Potent Antiosteoporotic Drug Molecular Scaffold Derived from *Angelica sinensis* and Its Bioinspired Total Synthesis

**DOI:** 10.1021/acscentsci.3c01414

**Published:** 2024-02-21

**Authors:** Jian Zou, Zuo-Cheng Qiu, Qiang-Qiang Yu, Jia-Ming Wu, Yong-Heng Wang, Ke-Da Shi, Yi-Fang Li, Rong-Rong He, Ling Qin, Xin-Sheng Yao, Xin-Luan Wang, Hao Gao

**Affiliations:** † Institute of Traditional Chinese Medicine and Natural Products, College of Pharmacy/International Cooperative Laboratory of Traditional Chinese Medicine Modernization and Innovative Drug Development of Chinese Ministry of Education of China/Guangdong Province Key Laboratory of Pharmacodynamic Constituents of TCM and New Drugs Research, Jinan University, Guangzhou 510632, People’s Republic of China; ‡ Translational Medicine R&D Center, Institute of Biomedical and Health Engineering/Key Laboratory of Biomedical Imaging Science and System, Shenzhen Institutes of Advanced Technology, Chinese Academy of Sciences, Shenzhen 518057, People’s Republic of China; § College of Traditional Chinese Medicine, Jinan University, Guangzhou 510632, People’s Republic of China

## Abstract

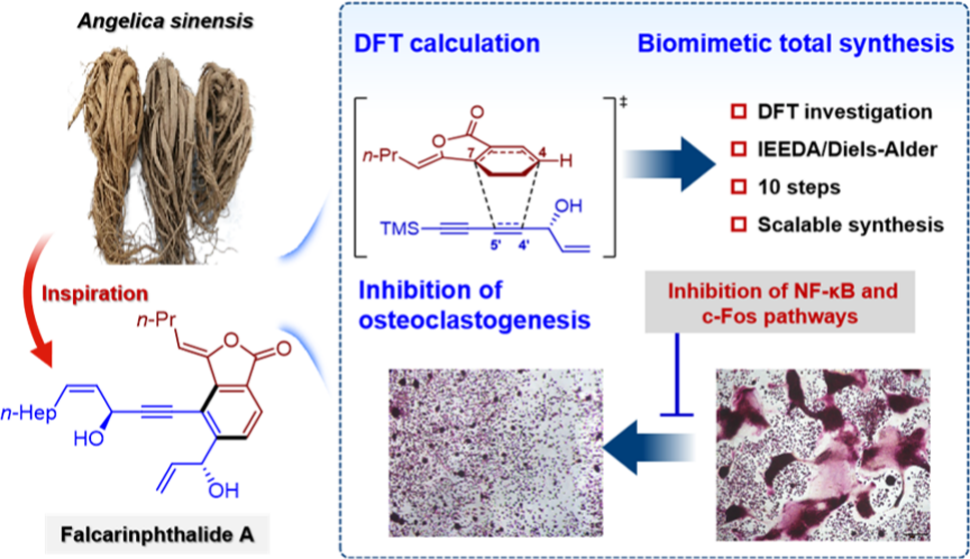

*Angelica sinensis*, commonly known as Dong Quai in Europe and America and as Dang-gui in China, is a medicinal plant widely utilized for the prevention and treatment of osteoporosis. In this study, we report the discovery of a new category of phthalide from *Angelica sinensis*, namely falcarinphthalides A and B (**1** and **2**), which contains two fragments, (3*R*,8*S*)-falcarindiol (**3**) and (*Z*)-ligustilide (**4**). Falcarinphthalides A and B (**1** and **2**) represent two unprecedented carbon skeletons of phthalide in natural products, and their antiosteoporotic activities were evaluated. The structures of **1** and **2**, including their absolute configurations, were established using extensive analysis of NMR spectra, chemical derivatization, and ECD/VCD calculations. Based on LC-HR-ESI-MS analysis and DFT calculations, a production mechanism for **1** and **2** involving enzyme-catalyzed Diels−Alder/retro-Diels−Alder reactions was proposed. Falcarinphthalide A (**1**), the most promising lead compound, exhibits potent in vitro antiosteoporotic activity by inhibiting NF-κB and c-Fos signaling-mediated osteoclastogenesis. Moreover, the bioinspired gram-scale total synthesis of **1**, guided by intensive DFT study, has paved the way for further biological investigation. The discovery and gram-scale total synthesis of falcarinphthalide A (**1**) provide a compelling lead compound and a novel molecular scaffold for treating osteoporosis and other metabolic bone diseases.

## Abstract 2

Inspired by the wisdom of traditional Chinese medicines, a new type of phthalide with potent antiosteoporotic activity, falcarinphthalide A, was discovered from *Angelica sinensis*.

## Introduction

Osteoporosis is a systemic metabolic bone disease characterized by loss of bone mass and strength, leading to increased bone fragility and susceptibility.^1^ The high incidence, rate of disability, and economic burden of osteoporosis have rendered it as a major global public health concern, drawing the attention of the World Health Organization.^2^ The current available first-line drugs for osteoporosis include bisphosphonates, hormone, and antibody-based drugs.^3^ Although these drugs have specific clinical efficacy for osteoporosis, their side effects, long-term safety, and high cost cannot be ignored.^4^ Traditional Chinese medicines (TCMs), which have been practiced in China for thousands of years, offer unique advantages in treating age-related diseases such as osteoporosis, and provide potential medicines with excellent efficacy and safety profiles. Numerous active ingredients derived from TCMs, such as flavonoids, phenylpropanoids, and terpenoids, have demonstrated remarkable effects on osteoporosis. Some of them are approved as drugs for the treatment of osteoporosis or are currently in the clinical trial stage.^2,5−7^ For instance, *Rhizoma drynariae* total flavonoids (Qianggu capsule, approved by NMPA 2003), which is rich in naringin and neoeriocitrin, and *Psoralea corylifolia* total glycosides (clinical trial stage I, approved by NMPA 2022, CXZL2101036), which is abundant in psoralenoside and isopsoralenoside.

*Angelica sinensis*, belonging to the family of Apiaceae, is a medicinal and edible plant with a long history of use in Europe and America, where it is known as Dong Quai. Its dried root, named as Dang-gui (Angelicae Sinensis Radix) in China, is a well-known TCM with the effect of nourishing and tonifying the blood.^8^ Traditionally, it is used for treating gynecological disorders, cerebral-cardiovascular diseases, and osteoporosis.^9−12^ In particular, Dang-gui is one of the most commonly used TCMs for the treatment of osteoporosis.^13−16^ Phytochemical investigations of Dang-gui have revealed that this plant is rich in phthalides (e.g., (*Z*)-ligustilide), phenylpropanoids (e.g., ferulic acid), polyacetylenes (e.g., (3*R*,8*S*)-falcarindiol), and so on.^17^ Among them, phthalides, for example, 3*H*-isobenzofuran-1-one, are the most commonly seen natural products widely distributed in Apiaceae plants. To date, approximately 180 natural phthalides have been isolated, and according to characteristics of phthalide units, they are generally classified into three categories: (i) 8-unsubstituted phthalides (e.g., mycophenolic acid and phthalidochromene), (ii) 8-substituted phthalides (e.g., *n*-butylphthalide, hydrastine, and penicidone A), and (iii) polymeric phthalides (e.g., levistilide A, triligustilide A, and triangeliphthalide A).^18−29^ In our previous research on Dang-gui, six pairs of active phthalide polymers with new skeletons were discovered.^24,25^ In continuing our studies on Dang-gui, two novel phthalides falcarinphthalides A and B (**1** and **2**) with unprecedented carbon skeletons and their biosynthetic precursors (3*R*,8*S*)-falcarindiol (**3**) and (*Z*)-ligustilide (**4**) were isolated. Falcarinphthalide A-B (**1** and **2**) are the first examples of phthalide heteropolymers, representing a new category of phthalides in natural products. The details of the isolation, structure elucidations, production mechanism, antiosteoporotic activities and mechanisms, and total synthesis of these phthalides are reported herein. This work presents the discovery of a new category of phthalide, namely falcarinphthalides A and B (**1** and **2**), originating from Dang-gui. Among these compounds, falcarinphthalide A (**1**) emerges as an exceptionally promising lead candidate, demonstrating remarkable in vitro antiosteoporotic activity. Its effectiveness lies in its ability to inhibit NF-κB and c-Fos signaling-mediated osteoclastogenesis. Furthermore, the bioinspired gram-scale total synthesis of compound **1**, guided by intensive DFT investigations, has opened up new avenues for comprehensive biological exploration in this field. The discovery of falcarinphthalide A (**1**) offers a groundbreaking molecular framework for potential therapeutic interventions targeting osteoporosis and other metabolic bone disorders.

**Figure 1:**
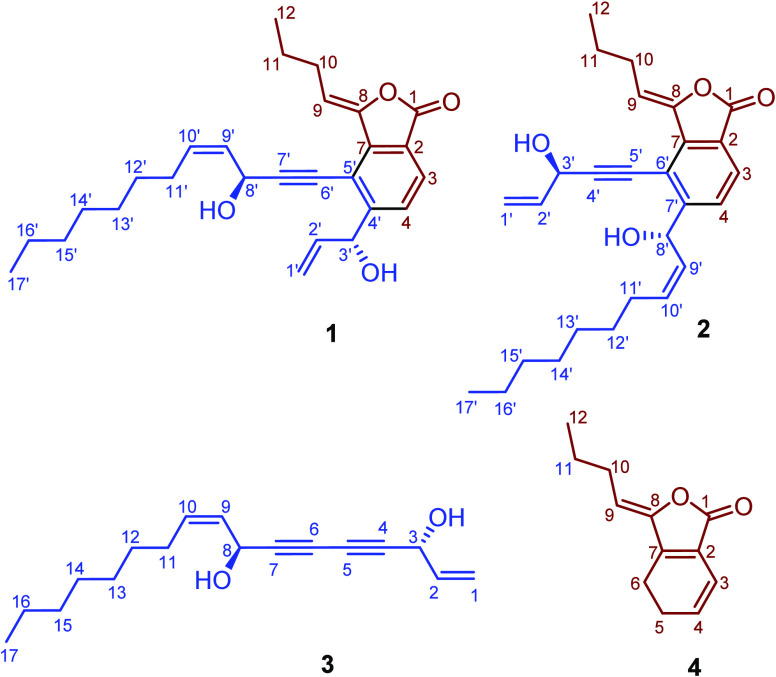
Chemical structures of **1**−**4**.

## Results and Discussion

### Structure Elucidation

**Figure 2:**
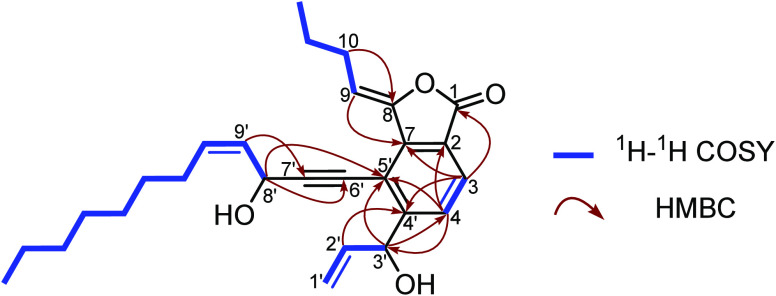
Key ^1^H−^1^H COSY and HMBC correlations of **1**.

Falcarinphthalide A (**1**) was isolated as a yellow oil. The molecular formula of **1** was established as C_27_H_34_O_4_ (11 degrees of unsaturation) from its HR-ESI-MS (*m*/*z* 423.2533 [M + H]^+^, calcd for [C_27_H_35_O_4_]^+^: 423.2535). Based on the molecular formula information, the degree of unsaturation, and the analyses of ^1^H−^1^H COSY and HMBC data (Figure 2), the planar structure was established as shown in Figure 1. The geometries of the double bonds of Δ^8^ and Δ^9′^ in **1** were assigned as *Z* configuration by the NOESY correlations between H-9 and H-8′/H-9′, and the small coupling constant between H-9′ and H-10′ (*J*_H-9′/H-10′_ = 10.4 Hz) in Table S2 (solvent: C_6_D_6_). The assignments of all proton and carbon resonances are provided in Table S1 (solvent: CDCl_3_) and Table S2 (solvent: C_6_D_6_).

**Figure 3:**
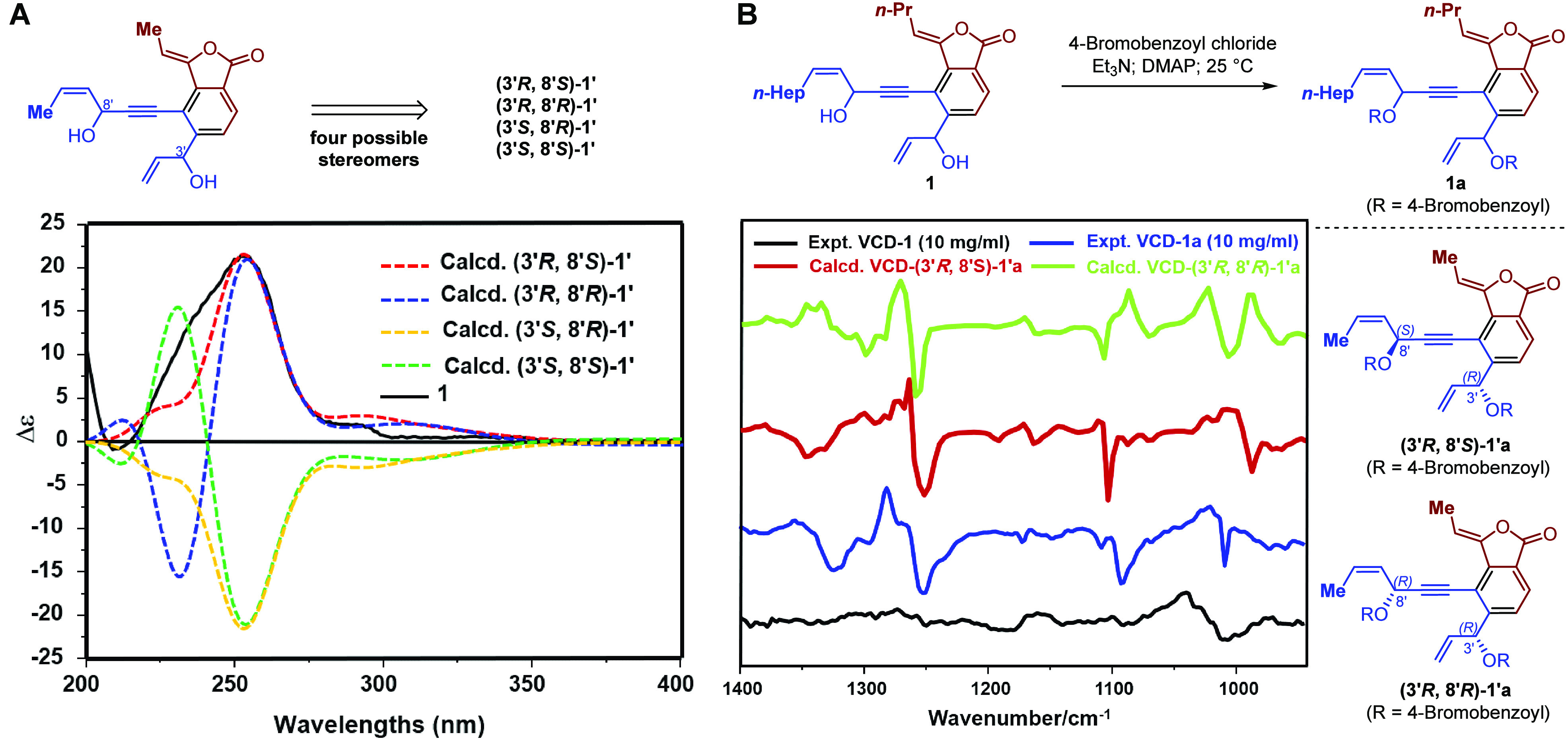
(A) Experimental ECD spectrum of **1**, and calculated ECD spectra of (3′*R*,8′*S*)-**1′**, (3′*R*,8′*R*)-**1′**, (3′*S*,8′*R*)-**1′**, and (3′*S*,8′*S*)-**1′** (UV correction = −18 nm, bandwidth σ = 0.3 eV). (B) Experimental VCD spectra of **1** and **1a** and calculated VCD spectra of (3′*R*,8′*S*)-**1′a** and (3′*R*,8′*R*)-**1′a** in the CDCl_3_ solution.

The absolute configuration of **1** was determined by ECD quantum-chemical calculation methods. Since the flexible side chains far away from the chiral carbons in **1** had an insignificant effect on the ECD spectrum,^30−32^ four possible simplified structures of stereomers (3′*R*,8′*S*)-**1′**, (3′*R*,8′*R*)-**1′**, (3′*S*,8′*R*)-**1′**, and (3′*S*,8′*S*)-**1′** were used for the ECD calculations at the B3LYP/TZVP level. The predicted ECD curve of (3′*R*,8′*S*)-**1′** was almost identical to the experimental one of **1** (Figure 3A), which suggested the absolute configuration of **1** as 3′*R*,8′*S*. Recently, vibrating circular dichroism (VCD) has become an attractive technique used for the stereochemistry determination of natural products.^33^ For this deduction to be confirmed, the VCD experiment of **1** was carried out. In light of the lack of strong enough VCD Cotton effects, **1** was treated with 4-bromobenzoyl chloride to yield the 4-bromobenzoic acylated product (**1a**),^34,35^ which had enhanced VCD Cotton effects (Figure 3B). Then two possible simplified structures of (3′*R*,8′*S*)-**1′a** and (3′*R*,8′*R*)-**1′a** were used for the VCD calculations at the B3LYP/6-311+G (2d,p) level (Figure 3B). The predicted VCD curve of (3′*R*,8′*S*)-**1′a** was almost identical to the experimental one of **1a** (Figure 3B), which was consistent with the result of ECD calculation. Therefore, the absolute configuration of **1** was established as (3′*R*,8′*S*)-**1**.

Falcarinphthalide B (**2**) was isolated as a yellow oil. The molecular formula of **2** was established as C_27_H_34_O_4_ (11 degrees of unsaturation) from its HR-ESI-MS (*m*/*z* 423.2534 [M + H]^+^, calcd for [C_27_H_35_O_4_]^+^: 423.2535). Based on the molecular formula information, the degree of unsaturation, and the analyses of ^1^H−^1^H COSY and HMBC data, the planar structure was established as shown in Figure 1. The geometries of the double bonds of Δ^8^ and Δ^9′^ in **2** were assigned as *Z* configuration by the NOESY correlations between H-9 and H-3′, and the small coupling constant between H-9′ and H-10′ (*J*_H-9′/H-10′_ = 10.5 Hz). The assignments of all proton and carbon resonances are provided in Table S4. By the same procedure of ECD calculations as that of **1** (Figure S6), the absolute configuration of **2** was determined as 3′*R*,8′*S*. Two related known compounds (**3** and **4**) were identified as (3*R*,8*S*)-falcarindiol (**3**) and (*Z*)-ligustilide (**4**) by comparison of the NMR data and optical rotation with literature values.^36−38^

### Discussion of Production Mechanism

**Figure 4:**
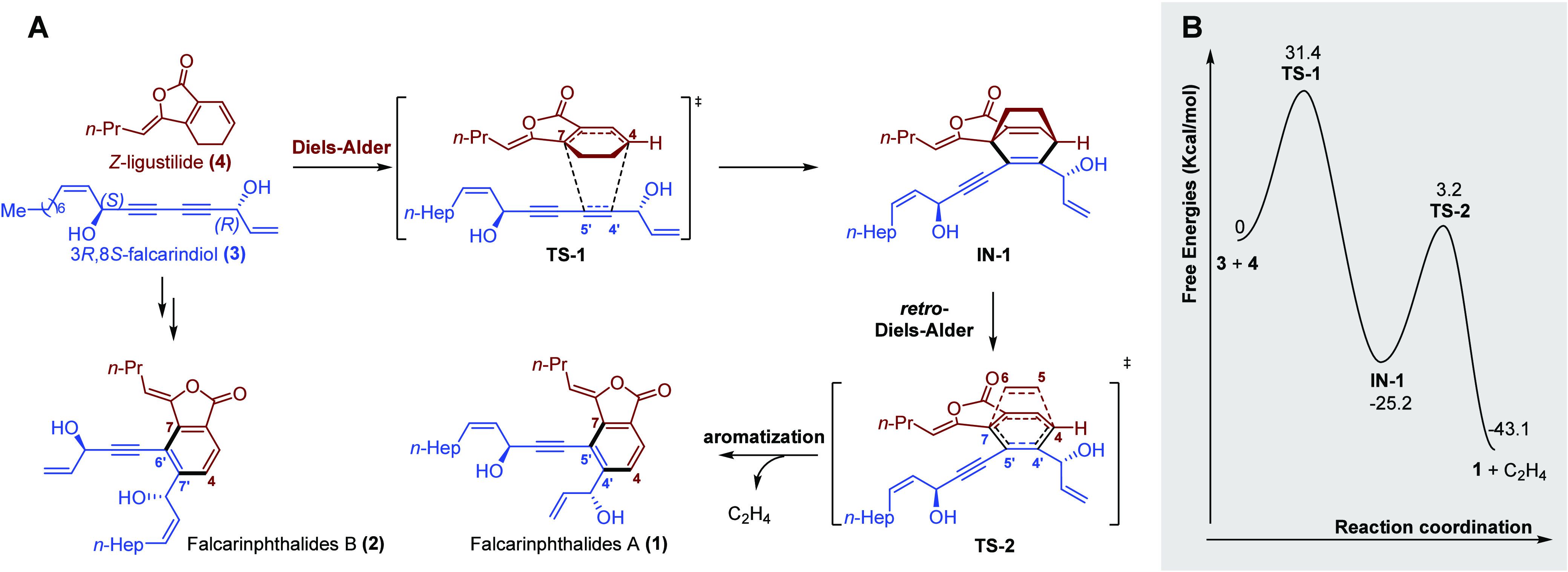
(A) The plausible modes of formation of compound **1**. (B) DFT-calculated energies profiles for Diels−Alder/retro-Diels−Alder reactions of **1** via **TS-1** and **TS-2**. Free energies are given in kcal mol^−1^.

As mentioned above, the phytochemical investigations of Dang-gui had shown that this plant was rich in phthalides and polyacetylenes. Remarkably, (*Z*)-ligustilide (**4**) has been recognized as the primary constituent of the phthalides, while (3*R*,8*S*)-falcarindiol (**3**) was the main constituent of the polyacetylenes.^17^ Based on these findings, a plausible mechanism for the formation of falcarinphthalide A (**1**) has been proposed. Compounds **3** and **4** are believed to act as its precursors, undergoing a Diels−Alder/retro-Diels−Alder reaction to facilitate its synthesis (Figure 4A). Compound **1** through C-4′ and C-5′ of the (3*R*,8*S*)-falcarindiol (**3**) moiety tethered to C-4 and C-7 of (*Z*)-ligustilide (**4**). Compound **2**, featuring a distinct linkage style (4−7′/7−6′), is derived from (3*R*,8*S*)-falcarindiol (**3**) and Z-ligustilide (**4**) through the same reaction referring to the formation of compound **1**.

In order to reveal the production mechanism of the new type of phthalides, compound **1** was selected as a representative example. An LC-HR-ESI-MS analysis of the extract of fresh Dang-gui was carried out, and this extract was obtained through cooling extraction with 95% EtOH/H_2_O in the dark. The LC-HR-ESI-MS analysis indicated that the ion peak (*m*/*z* 421.2389/C_27_H_33_O_4_ at *t*_R_ = 57.6 min) and the MS^2^ spectrum in the extract were consistent with the retention time, the ion peak (*m*/*z* 421.2385/C_27_H_33_O_4_), and the MS^2^ spectra in the chromatogram of **1**, which strongly suggested that **1** was produced in this plant (see Supporting Information). A computational study was then carried out to investigate the mechanistic details of the proposed Diels−Alder/retro-Diels−Alder cascade (Figure 4B). The DFT calculations showed that the cascade reaction is highly exergonic (−43.1 kcal/mol), suggesting that it is favorable in thermodynamics, but the overall activation barrier is relatively high (31.4 kcal/mol), indicating that this Diels−Alder/retro-Diels−Alder process is infeasible in kinetics.^39−41^ In recent years, there has been a continuous discovery of naturally sourced Diels−Alderase enzymes, such as MPS, PPS, AbyU, MaDA, PyrE3, and so on.^42−48^ These enzymes possess unique catalytic properties that enable them to accelerate the rate of the Diels−Alder reaction and promote the formation of the desired products. Therefore, combining the DFT calculations, it is speculated that a Diels−Alderase is involved in the formation of falcarinphthalides A and B (**1** and **2**).

**Figure 5:**
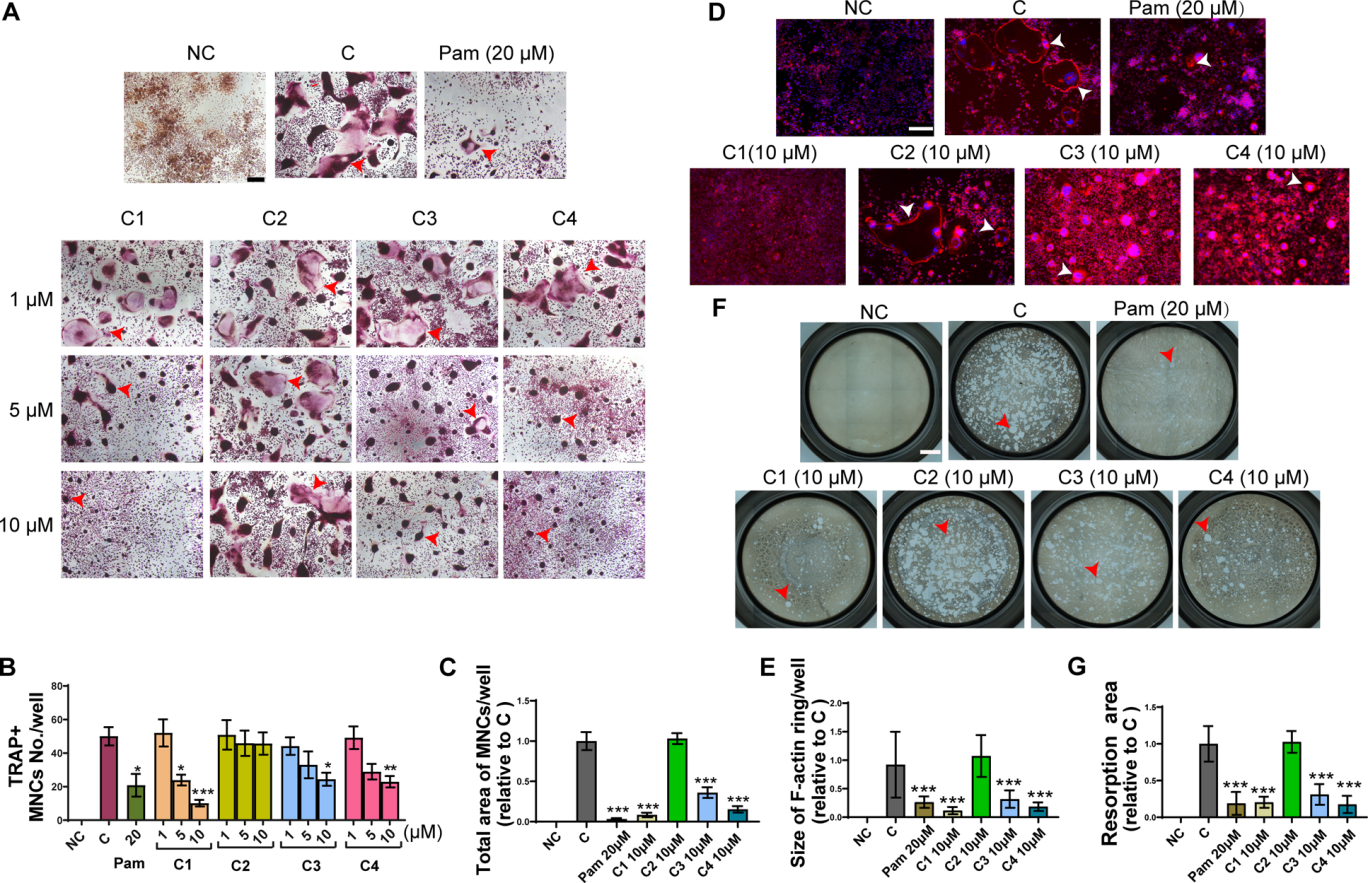
Compounds **1**, **3**, and **4**, but not **2**, inhibit osteoclast formation and bone resorption via suppressing osteoclastogenesis. (A) Typical images of tartrate-resistant acid phosphatase (TRAP) staining cells treated with different dosages of **1**−**4** and positive control (pamidronate; 20 μM) for receptor activator of nuclear factor-κB ligand (RANKL) and macrophage colony stimulating factor (M-CSF) induced 4 days. TRAP staining-positive (TRAP^+^) multinucleated cells (MNCs, nuclei ≥3) were counted as osteoclasts. Scale bar = 100 μm. Red arrow: osteoclast. (B) Number of TRAP^+^ MNCs in each field. (C) The total area of MNCs per well relative to control. (D) Typical images of F-actin ring treated with **1**−**4** (10 μM) and positive control (pamidronate; 20 μM). Scale bar = 100 μm. White arrow: F-actin ring. (E) Quantitative analyses of size of F-actin rings in each field. (F) Typical images of resorption pits per well (96 well plate) treated with **1**−**4** (10 μM) and positive control (pamidronate; 20 μM). Red arrow: resorbed pits. Scale bar = 1000 μm. (G) Quantitative analyses of the percentage of the area of pits resorbed by osteoclasts in each well relative to control. The data were expressed as mean ± SD * *P* < 0.05, ** *P* < 0.01, ***P < 0.001 vs Control; *n* ≥ 3. NC: Control without RANKL-induced; C: Control with RNAKL and M-CSF induced; TRAP: tartrate-resistant acid phosphatase; Pam: pamidronate; C1−C4: Compounds **1**−**4**.

### In Vitro Antiosteoporotic Activity and Mechanisms

In terms of bioactivity, considering that targeting osteoclast is one of the primary therapeutic strategies for the treatment of osteoporosis and other metabolic bone diseases,^49^ the effects of compounds **1**−**4** on osteoclastogenesis, osteoclastic F-actin formation, and osteoclastic bone resorption were evaluated by using RANKL and M-CSF induced RAW264.7 cells. As shown in Figure 5, compounds **1**, **3**, and **4** exerted antiosteoclastogenic activities in a dose-dependent manner (Figure 5A−C), resulting in the disruption of F-actin ring formation (essential for osteoclast attachment to the bone matrix) (Figure 5D,E) and inhibition of bone resorbed pits formation (Figure 5F,G). It is worth noting that compound **2** did not demonstrate any of the aforementioned effects, indicating that the diverse forms of linkage of falcarinphthalides play a crucial role in modulating their antiresorptive activity.

**Figure 6:**
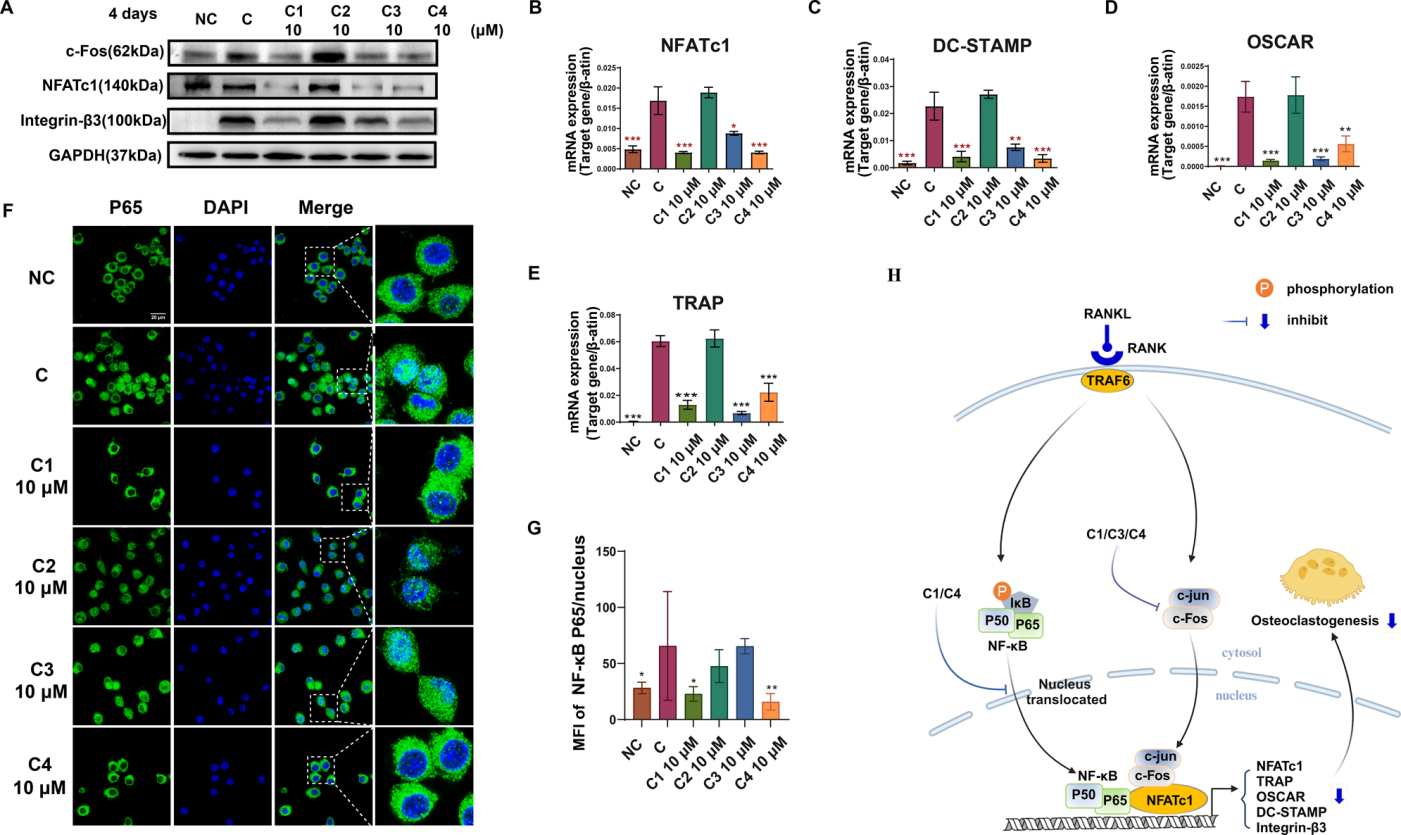
Compounds **1**, **3**, and **4** block osteoclastogenesis via suppressing RANKL-induced activation of of NF-κB/c-Fos signaling. (A) Analysis of protein expression and (B−E) relative mRNA levels of osteoclastogenesis-related markers in RAW264.7 cells cultured in the presence or absence of compounds **1**−**4** with RANKL and M-CSF. (F) Confocal microscopy images showing the nuclear translocation of NF-κB p65 in RANKL-induced RAW264.7 cells after 1 h incubation, with or without pretreatment using compounds **1**−**4** (10 μM) for 4 h. NF-κB p65 is represented in green, while cell nuclei (DAPI) are shown in blue. Scale bar = 20 μm. (G) Quantification of mean per-pixel fluorescence intensity (MFI) of NF-κB p65 in the nucleus. (H) A proposed scheme illustrating the inhibitory effects of compounds **1**, **3**, and **4** on osteoclastogenesis. Data are expressed as mean ± SD *P < 0.05, **P < 0.01, ***P < 0.001 vs control. *n* ≥ 3.

Next, an exploration was undertaken to investigate the antiosteoclastogenic mechanism of falcarinphthalide A (**1**). It is well established that c-Fos and nuclear factor of activated T cells 1 (NFATc1) play crucial roles as transcription regulators in osteoclastogenesis.^50^ These present findings demonstrated that compounds **1**, **3**, and **4** effectively reduced the expression of c-Fos and NFATc1 (Figure 6A,B). This reduction subsequently resulted in decreased levels of osteoclastogenesis related molecules, including integrin-β3, dendritic cell-specific transmembrane protein (DC-STAMP), osteoclast-associated receptor (OSCAR), and TRAP (Figure 6A−E). As is known, the RANKL-induced nuclear factor kappa B (NF-κB) signaling pathway is vital for osteoclastogenesis,^51^ with the nuclear translocation of p65 being a pivotal event in this process. These present results revealed that compounds **1** and **4** effectively suppressed the nuclear translocation of NF-κB p65, while compound **3** did not have this effect (Figure 6F,G). Notably, compound **2** also did not display any of the aforementioned inhibitory effects in these pathways. Taken together, these findings indicated that falcarinphthalide A (**1**), characterized by a specific linkage style (4−4′/7−5′), inhibits RANKL-induced osteoclastogenesis through the suppression of both NF-κB and c-Fos pathways **(**Figure 6H**)**. It was observed that compound **1** at the concentration of 5 μM demonstrated a comparable antiosteoclatogenesis effect to 20 μM pamidronate, a commonly used clinical antiosteoporotic bisphosphonate. This suggests that compound **1** has a potent antiresortpion effect. Additionally, bisphosphonates have a half-life of 1−10 years due to their irreversible binding to bone via their P−C−P backbone structure, leading to accumulation and long-term induction of osteoclast apoptosis. This disrupts the normal cross talk between osteoclast and osteoblast.^52,53^ In contrast, compound **1**, without a P−C−P backbone structure, may potentially avoid these adverse events. Furthermore, compound **1** was able to directly target osteoclastogenesis, which is different from estrogen’s indirect suppression of osteoclastic bone resorption via promoting secretion of the OPG/RANKL ratio in osteoblasts.^54^ Thus, falcarinphthalide A (**1**) was discovered as a potent lead compound with distinct antiresorptive mechanisms.

### DFT-Guided Bioinspired Total Synthesis

**Figure 7:**
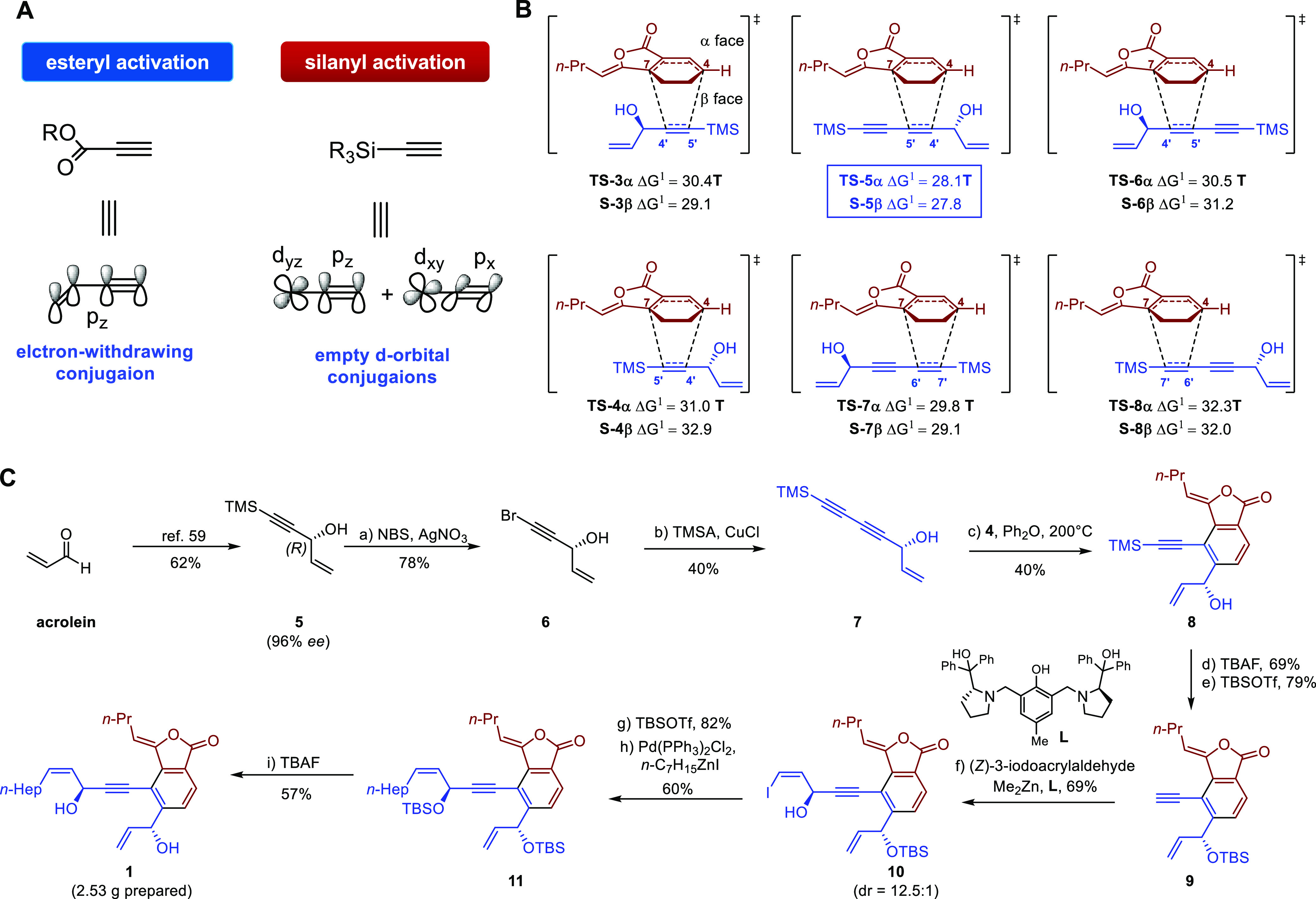
(A) Comparison of esteryl and silanyl activations. (B) DFT-calculated transition states for the proposed Diels−Alder reaction (only the transition states with β-face selectivity are shown for clarity). (C) Gram-scale total synthesis of falcarinphthalide A (**1**).

The unique bioactivity of **1** encouraged us to explore its total synthesis for further biological study. The proposed biosynthetic pathway and relevant computational study in Figure 4 provide a practical blueprint for the total synthesis of falcarinphthalide A (**1**). Due to the low reactivity of compound **3** as a dienophile, as illuminated in the above calculations, all attempts of the direct Diels−Alder reaction between compounds **3** and **4** failed (Table S12); thus, a preactivated alkynyl dienophile was needed. Although the ester-activated alkynyl dienophiles were reported to be competent in Diels−Alder reaction with compound **4**,^55,56^ the subsequent elongation of the side chain needs nucleophilic addition, which may break the lactone ring of (*Z*)-ligustilide. Herein, silane was chosen as the activation group due to its ease of removal and ability to circumvent nucleophilic addition.^57^ Unlike the esteryl group that activates the alkyne through the electron-withdrawing conjugation, the silanyl group uses the empty d orbitals to accommodate the electrons of the alkynyl (Figure 7A). Accordingly, two silicane dienophiles were designed, (*R*)-5-(trimethylsilyl)pent-1-en-4-yn-3-ol (**5**) and (*R*)-7-(trimethylsilyl)hepta-1-en-4,6-diyn-3-ol (**7**) as the dienophile, and additional DFT computational study was performed to ascertain their regioselectivity in the proposed Diels−Alder/retro-Diels−Alder reaction. As shown in Figure 7B, the activation energy of 12 possible Diels−Alder transition states with different enantioselectivities (α- or β-face addition) and regioselectivities were calculated (**TS-3** and **TS-4** for dienophile **5**; **TS-5**, **TS-6**, **TS-7**, and **TS-8** for dienophile **7**). According to the calculation results, **TS-5β** was expected as the most favorable transition state bearing an energy barrier of 27.8 kcal/mol. These trends could be explained by frontier molecular orbital (FMO) theory,^58^ which showed that **7** had a smaller HOMO_dienophile_−LUMO_diene_ gap than **5** and also indicated an inverse electron demand Diels−Alder reaction (IEDDA, Figure S18).

Guided by the quantum chemistry computations investigation of the IEDDA reaction, a decisive choice was made to undertake the total synthesis of falcarinphthalide A (**1**). The gram-scale total synthesis of **1** began with the preparation of enantiopure alcohol **7** (Figure 7C). According to Pu’s procedure,^59^ the allylic alcohol **5** was obtained in 62% yield with 96% ee, which was then subjected to oxidative bromination to give **6** in the presence of AgNO_3_, and NBS.^38,60^ Thus, **7** could be easily obtained in 40% yield by adopting the Cadiot−Chodkiewicz cross-coupling reaction between **6** and TMSA.^61,62^ As expected, the IEDDA/reverse Diels−Alder cascade of **4** and **7** was performed at 200 °C in Ph_2_O and gave the desired cycloadduct **8** with 40% yield. Then the TMS group of **8** was removed in the presence of TBAF, and the hydroxyl group was protected with TBS and gave **9** in 55% yield over two steps. At this stage, the construction of the C-8′ stereocenter was achieved by converting **9** to the corresponding zinc reagent and reacting with the (*Z*)-3-iodoacrylaldehyde in the presence of L (0.17 equiv),^63,64^ which gave iodide **10** in 69% yield. The newly formed hydroxyl group in **10** was then protected, and the corresponding product could proceed with the Negishi cross-coupling reaction in the presence of Pd(PPh_3_)_2_Cl_2_ and heptylzinc(II) iodide,^65^ giving **11** with 60% yield. The global deprotection finally afforded falcarinphthalide A (**1**) with 57% yield, and the ^1^H and ^13^C NMR spectra, ECD spectra, and optical rotation of synthesized **1** agreed with the natural product.

## Conclusion

In summary, falcarinphthalides A and B (**1** and **2**), a new category of phthalides possessing unprecedented carbon skeletons, were isolated from an antiosteoporotic TCM (Dang-gui). This discovery reveals an entirely new subclass of TCM chemical constituent, and these two compounds represent a new category of phthalide in natural products. The bioassays revealed that falcarinphthalide A (**1**) and its biosynthetic precursors (**3** and **4**) displayed potent antiosteoclastogenic activities, whereas falcarinphthalide B (**2**) was inactive, which indicates that the antiosteoclastogenic activity of falcarinphthalides is highly relevant to its linkage styles. Significantly, falcarinphthalide A (**1**) exerts a multifaceted mechanism of action by inhibiting RANKL-induced osteoclastogenesis through the suppression of both the NF-κB and c-Fos pathways. Guided by the proposed biosynthetic pathway and intensive computational study, the total synthesis of **1** has been successfully achieved in 10 steps, featuring bioinspired IEDDA/reverse Diels−Alder cascade as the critical step. The attainment of a gram-scale total synthesis of **1** provides ample material basis for further biological study. Falcarinphthalide A (**1**) exhibits a distinctive structure and mechanism that sets it apart from existing drugs such as bisphosphonates and estrogens. These distinguishing features hold great potential for overcoming the adverse side effects commonly associated with current medications. However, in the next phase of research, it is necessary to identify its target receptors, study its in vivo efficacy, and even pursue structural modifications. Overall, these ground-breaking findings not only extend the natural product skeleton categories but also highlight the potential of natural products as a source of novel molecular scaffold for treating osteoporosis.

## Supporting Information Available

General experimental procedures; plant material; extraction and isolation; NMR data assignments of **1**, **2**, and **1a**; structural characterizations of **1**−**4**; quantum chemical ECD/VCD calculation details; antiosteoclastogenic activity assays of **1**−**4**; the related operation of LC-HR-ESI-MS analysis; the DFT calculation details of chemical reactions; total synthesis of **1**; the 1D and 2D NMR spectra of **1**, **2**, and **1a**; the experimental spectra of total synthesis of **1**; Tables S1−S14 and Figures S1−S67 (PDF)
