# Supplementary material for: Discovery of a Potent Antiosteoporotic Drug Molecular Scaffold Derived from Angelica sinensis and Its Bioinspired Total Synthesis
Source: ACS Cent Sci. 2024 Feb 21;10(3):628–36. doi: 10.1021/acscentsci.3c01414 (PMC10979506; doi:10.1021/acscentsci.3c01414)
Supplement: Supplementary file 1 — oc3c01414_si_001.pdf [file oc3c01414_si_001.pdf]

Title: Discovery of a potent anti-osteoporotic drug molecular scaffold derived from *Angelica sinensis* and its bioinspired total synthesis

**Jian Zou**	***Institute of Traditional Chinese Medicine and Natural Products, College of Pharmacy / International Cooperative Laboratory of Traditional Chinese Medicine Modernization and Innovative Drug Development of Chinese Ministry of Education of China / Guangdong Province Key Laboratory of Pharmacodynamic Constituents of TCM and New Drugs Research, Jinan University, Guangzhou 510632, People's Republic of China.***
**Zuo-Cheng Qiu**	***Institute of Traditional Chinese Medicine and Natural Products, College of Pharmacy / International Cooperative Laboratory of Traditional Chinese Medicine Modernization and Innovative Drug Development of Chinese Ministry of Education of China / Guangdong Province Key Laboratory of Pharmacodynamic Constituents of TCM and New Drugs Research, Jinan University, Guangzhou 510632, People's Republic of China; Translational Medicine R&D Center, Institute of Biomedical and Health Engineering / Key Laboratory of Biomedical Imaging Science and System, Shenzhen Institutes of Advanced Technology, Chinese Academy of Sciences, Shenzhen 518057, People's Republic of China; College of Traditional Chinese Medicine, Jinan University, Guangzhou 510632, People's Republic of China.***
**Qiang-Qiang Yu**	***Institute of Traditional Chinese Medicine and Natural Products, College of Pharmacy / International Cooperative Laboratory of Traditional Chinese Medicine Modernization and Innovative Drug Development of Chinese Ministry of Education of China / Guangdong Province Key Laboratory of Pharmacodynamic Constituents of TCM and New Drugs Research, Jinan University, Guangzhou 510632, People's Republic of China.***
**Jia-Ming Wu**	***Institute of Traditional Chinese Medicine and Natural Products, College of Pharmacy / International Cooperative Laboratory of Traditional Chinese Medicine Modernization and Innovative Drug Development of Chinese Ministry of Education of China / Guangdong Province Key Laboratory of Pharmacodynamic Constituents of TCM and New Drugs Research, Jinan University, Guangzhou 510632, People's Republic of China.***
**Yong-Heng Wang**	***Institute of Traditional Chinese Medicine and Natural Products, College of Pharmacy / International Cooperative Laboratory of Traditional Chinese Medicine Modernization and Innovative Drug Development of Chinese Ministry of Education of China / Guangdong Province Key Laboratory of Pharmacodynamic Constituents of TCM and New Drugs Research, Jinan University, Guangzhou 510632, People's Republic of China.***
**Ke-Da Shi**	***Translational Medicine R&D Center, Institute of Biomedical and Health Engineering / Key Laboratory of Biomedical Imaging Science and System, Shenzhen Institutes of Advanced Technology, Chinese Academy of Sciences, Shenzhen 518057, People's Republic of China.***
**Yi-Fang Li**	***Institute of Traditional Chinese Medicine and Natural Products, College of Pharmacy / International Cooperative Laboratory of Traditional Chinese Medicine Modernization and Innovative Drug Development of Chinese Ministry of Education of China / Guangdong Province Key Laboratory of Pharmacodynamic Constituents of TCM and New Drugs Research, Jinan University, Guangzhou 510632, People's Republic of China.***
**Rong-Rong He**	***Institute of Traditional Chinese Medicine and Natural Products, College of Pharmacy / International Cooperative Laboratory of Traditional Chinese Medicine Modernization and Innovative Drug Development of Chinese Ministry of Education of China / Guangdong Province Key Laboratory of Pharmacodynamic Constituents of TCM and New Drugs Research, Jinan University, Guangzhou 510632, People's Republic of China.***
**Ling Qin**	***Translational Medicine R&D Center, Institute of Biomedical and Health Engineering / Key Laboratory of Biomedical Imaging Science and System, Shenzhen Institutes of Advanced Technology, Chinese Academy of Sciences, Shenzhen 518057, People's Republic of China.***
**Xin-Sheng Yao**	***Institute of Traditional Chinese Medicine and Natural Products, College of Pharmacy / International Cooperative Laboratory of Traditional Chinese Medicine Modernization and Innovative Drug Development of Chinese Ministry of Education of China / Guangdong Province Key Laboratory of Pharmacodynamic Constituents of TCM and New Drugs Research, Jinan University, Guangzhou 510632, People's Republic of China.***
**Xin-Luan Wang (Corresponding author)**	***Translational Medicine R&D Center, Institute of Biomedical and Health Engineering / Key Laboratory of Biomedical Imaging Science and System, Shenzhen Institutes of Advanced Technology, Chinese Academy of Sciences, Shenzhen 518057, People's Republic of China; E-mail: xl.wang@siat.ac.cn.***
**Hao Gao (Corresponding author)**	***Institute of Traditional Chinese Medicine and Natural Products, College of Pharmacy / International Cooperative Laboratory of Traditional Chinese Medicine Modernization and Innovative Drug Development of Chinese Ministry of Education of China / Guangdong Province Key Laboratory of Pharmacodynamic Constituents of TCM and New Drugs Research, Jinan University, Guangzhou 510632, People's Republic of China; Email:tghao@jnu.edu.cn.***

**Pages**	**119**	**Tables**	**14**	**Figures**	**67**

Supplementary Information

Discovery of a potent anti-osteoporotic drug molecular scaffold derived from *Angelica sinensis* and its bioinspired total synthesis

Jian Zou,[+a] Zuo-Cheng Qiu,[+a,b,c] Qiang-Qiang Yu,[a] Jia-Ming Wu,[a] Yong-Heng Wang,[a] Ke-Da Shi,[b] Yi-Fang Li,[a] Rong-Rong He,[a] Ling Qin,[b] Xin-Sheng Yao,[a] Xin-Luan Wang[*b] and Hao Gao[*a]

[a] *Institute of Traditional Chinese Medicine and Natural Products, College of Pharmacy / International Cooperative Laboratory of Traditional Chinese Medicine Modernization and Innovative Drug Development of Chinese Ministry of Education of China / Guangdong Province Key Laboratory of Pharmacodynamic Constituents of TCM and New Drugs Research, Jinan University, Guangzhou 510632, People's Republic of China.*

[b] *Translational Medicine R&D Center, Institute of Biomedical and Health Engineering / Key Laboratory of Biomedical Imaging Science and System, Shenzhen Institutes of Advanced Technology, Chinese Academy of Sciences, Shenzhen 518057, People's Republic of China.*

[c] *College of Traditional Chinese Medicine, Jinan University, Guangzhou 510632, People's Republic of China.*

[+] *These authors contributed equally to this work.*

[*] *Corresponding author :Hao Gao (Email: tghao@jnu.edu.cn); Xin-Luan Wang (Email: xl.wang@siat.ac.cn)*

Table of Content

Experimental Procedures 4

1. General experimental procedures 4
2. Plant material 5
3. Extraction and isolation 6

Results and Discussion 7

1. NMR data assignments of 1−2, and 1a 7
2. Preparation of the 3',8'-bis-*O*-(4-bromobenzoyl) derivative of 1 (1a) 11
3. Structural characterizations of 1−4 12
4. Quantum chemical ECD calculations of 1′−2′ 13
5. Quantum chemical VCD calculations of 1′a 21
6. Anti-osteoclastogenic activity assays of 1−4 27
7. The related operation of LC-HR-ESI-MS analysis 30
8. The DFT calculation details of chemical reactions 35
9. Total synthesis of falcarinphthalide A (1) 56
10. The 1D and 2D NMR spectra of 1−2, and 1a 76
11. The experimental spectra of total synthesis of falcarinphthalide A (1) 100

Experimental Procedures

1. General experimental procedures

Methanol (MeOH) was purchased from Yuwang Industrial Co. Ltd (Yucheng, China). Acetonitrile (MeCN) was obtained from Oceanpak Alexative Chemical Co. Ltd (Gothenburg, Sweden). Ethanol (EtOH), Ethyl acetate (EtOAc), Cyclohexane, Petroleum ether, and chloroform ($CHCl_3$) were analytical grade from Fine Chemical Co. Ltd (Tianjin, China).

Unless otherwise mentioned, all reactions were carried out under an argon atmosphere with dry solvents, unless otherwise noted. Reagents were purchased at the highest commercial quality and used without further purification, unless otherwise stated. Solvents purification was conducted according to Purification of Laboratory Chemicals (Peerrin, D. D.; Armarego, W. L. and Perrins, D. R., Pergamon Press: Oxford, 1980). Concentration of solutions was accomplished using a EYELA rotary evaporator with a water aspirator (Tokyo Rikakikai Co. Ltd, Tokyo, Japan). Yields refer to chromatographically and spectroscopically (^{1}H NMR) homogeneous materials. This was generally followed by removal of residual solvents on a vacuum line held at 0.1-1 torr.

Reactions were monitored by thin-layer chromatography (TLC) carried out on 0.2 mm Tsingdao silica gel plates (SGF254, 0.2 mm, Yantai Chemical Industry Research Institute, China). Visualization on TLC was achieved by use of UV light at 254 nm, exposure to iodine vapor. Staining was performed with an ethanolic solution of phosphomolybdic acid (PMA) and cerium sulfate, or by oxidative staining with an aqueous basic potassium permanganate ($KMnO_4$) solution and subsequent heating. Tsingdao silica gel (200-300 mesh) was used for flash column chromatography.

UV data were recorded by using a JASCO V-550 UV/vis spectrometer (Jasco International Co. Ltd, Tokyo, Japan). IR data were recorded on a JASCO FT/IR-480 plus spectrometer (Jasco International Co. Ltd, Tokyo, Japan). Optical rotations were measured on a JASCO P1020 digital polarimeter (Jasco International Co. Ltd, Tokyo, Japan). The ECD spectra were recorded in MeOH by using a Chirascan qCD spectrophotometer (Applied Photophysics Ltd., London, Britain) at room temperature. The IR and VCD spectra of **1** in $CDCl_3$ solution were measured at a resolution of 4 cm^{-1} using Synchrocell (2.75 sec per cycle) on a BioTools ChiralIR-2X VCD spectrometer (BioTools Ltd., Florida, America). HRESIMS spectra were obtained on Waters Synapt G2 TOF mass spectrometer (Waters Corporation, Milford, America). 1D and 2D NMR spectra were acquired with Bruker AV 400/AV 600 spectrometers (Bruker BioSpin Group, Faellanden, Switzerland) by using the solvent signals ($CDCl_3$: δ_H 7.26/δ_C 77.0; C_6D_6: δ_H 7.16/δ_C 128.0) as internal standards. Column chromatography (CC) was carried out on silica gel (200–300 mesh) (Qingdao Haiyang Chemical Group Corporation, Qingdao, China), and HP-20 (Mitsubishi Chemical Co. Ltd. Japan). TLC was performed on precoated silica gel plate (SGF254, 0.2 mm, Yantai Chemical Industry Research Institute, China). Analytical HPLC was performed on a Dionex HPLC system equipped with an Ultimate 3000 pump, an Ultimate 3000 diode array detector, an Ultimate 3000 column compartment, an Ultimate 3000 autosampler (Dionex, America), and an Alltech (Grace) 2000ES evaporative light scattering detector (Alltech America) by using a Phenomenex Gemini C_{18} column (4.6 × 250 mm, 5 μm), and a Phenomenex Biphenyl column (4.6 × 250 mm, 5 μm). Semi-preparative HPLC was carried out on Shimadzu LC-6AD system equipped with UV detectors, using a Phenomenex Biphenyl column (10.0 × 250 mm, 5 μm). Preparative HPLC was carried out on Shimadzu LC-6AD system equipped with UV detectors, using a Phenomenex Gemini C_{18} column (21.2 × 250 mm, 5 μm). Medium pressure liquid chromatography (MPLC) was performed on ODS column (4.0 × 30 cm, 50 μm) and equipped with a dual pump gradient system, a UV preparative detector, and a Dr Flash II fraction collector system (Shanghai Lisui E-Tech Co., Ltd., Shanghai, China).

2. Plant material

The dried root of *Angelica sinensis* was collected from the GAP base (CFDA, No. 5, 5.23, 2014.) of *Angelica sinensis* from Zhanyi County of Yunnan province in China by Zhanyi Yikang Chinese Herbal Medicine Co. Ltd. in November 2014. The plant materials were identified by Dr. Ying Zhang in the College of Pharmacy, Jinan University, Guangzhou, China.

3. Extraction and isolation

The dried root of *Angelica sinensis* (45.0 kg) was refluxed four times with 125 L of 95% EtOH–H_2O for 2 h each time. After filtration, the EtOH was removed under reduced pressure to yield a concentrated solution (10 L). The solution was passed through a HP-20 macroporous resin column (20 × 125 cm) and successively eluted with 0, 40, and 95% EtOH–H_2O, yielding three fractions (F1–F3, 3.8, 0.4, and 1.6 kg, respectively). A portion (800.0 g) of F3 was subjected to open silica-gel CC, which was eluted with Cyclohexane–EtOAc–MeOH (39:1:0, 0:100:0, and 0:0:100) to yield three fractions (F3.1–F3.3, 271.9, 383.3, and 117.7 g, respectively). A portion (0.5 g) of F3.1 was purified using preparative HPLC eluted with MeOH–H_2O (55:45, v/v) at a flow rate of 8 mL/min to yield **4** (t_R: 55.0 min, 20.5 mg). F3.2 was subjected to silica-gel CC, which was eluted with Petroleum ether–EtOAc–MeOH (98:2:0, 95:5:0, 91:9:0, 80:20:0, 70:30:0, 60:40:0, 50:50:0, 0:100:0, and 0:0:100) to yield nine fractions (F3.2.1–F3.2.9, 1.2, 30.3, 60.3, 80.5, 40.3, 30.3, 33.3, 41.3, and 31.3 g, respectively). F3.2.5 was subjected to MPLC, which was eluted successively with MeOH–H_2O (60:40, 70:30, 80:20, 90:10, and 100:0) to yield five fractions (F3.2.5.1–F3.2.5.5, 5.5, 18.3, 9.5, 3.3, and 2.4 g respectively). F3.2.5.3 was subjected to MPLC, which was eluted with MeOH–H_2O (80:20) for 500 min to yield six fractions (F3.2.5.3.1–F3.2.5.3.6, 1.3, 2.1, 2.0, 1.5, 0.8, and 1.4 g respectively). F3.2.5.3.4 was isolated using semi-preparative HPLC eluted with MeOH–H_2O (85:15, v/v) at a flow rate of 3 mL/min to yield **1** (t_R: 19.4 min, 25.0 mg) and **2** (t_R: 21.9 min, 35.0 mg). F3.2.5.3.5 was isolated using semi-preparative HPLC eluted with MeOH–H_2O (90:10, v/v) at a flow rate of 3 mL/min to yield **3** (t_R: 14.2 min, 20.9 mg).

Results and Discussion

1. NMR data assignments of 1−2, and 1a

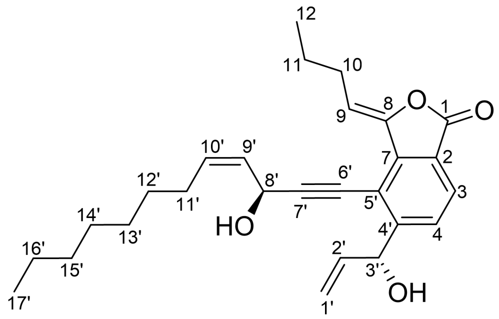

1

Table S1. NMR data of **1** in $CDCl_3$ (600 MHz for ^1H; 151 MHz for ^{13}C)

No.	δ_C, mult.	δ_H (J in Hz)*	^1H, ^1H-COSY	HMBC	NOESY
1	166.5, qC				
2	124.1, qC				
3	125.7, CH	7.85, d (8.0)	4	1, 4, 7, 4′	
4	126.5, CH	7.67, d (8.0)	3	1, 2, 3, 3′, 5′	
7	138.8, qC				
8	145.0, qC				
9	114.7, CH	6.43, t (7.7)	10	7, 8, 11	8′, 9′
10	28.3, CH_2	2.47	9, 11	8, 9, 11, 12	
11	22.5, CH_2	1.55	10, 12	9, 10, 12	
12	13.9, CH_3	0.99, t (7.4)	11	10, 11	
1′	116.1, CH_2	5.20, dt (10.3, 1.2), Ha	1′b, 2′, 3′	3′	
		5.42, dt (17.0, 1.2), Hb	1′a, 2′, 3′	2′, 3′	
2′	138.0, CH	5.95, ddd (17.0, 10.3, 5.6)	1′a, 1′b, 3′	3′, 4′	
3′	72.2, CH	5.71, br d (5.6)	1′a, 1′b, 2′	4, 1′, 2′, 4′, 5′	
4′	151.8, qC				
5′	113.1, qC				
6′	78.8, qC				
7′	102.0, qC				
8′	58.8, CH	5.48, d (7.2)	9′	5′, 6′, 7′, 9′, 10′	9, 11′
9′	128.0, CH	5.67	8′, 10′, 11′	7′, 8′, 11′	9
10′	134.4, CH	5.68	9′, 11′	8′, 11′, 12′	
11′	27.8, CH_2	2.17	9′, 10′, 12′	9′, 10′, 12′, 13′	8′
12′	29.3, CH_2	1.41	11′, 13′	10′, 11′, 13′, 14′	
13′	29.1, CH_2	1.29	12′, 14′	11′, 12′, 14′, 15′	
14′	29.2, CH_2	1.28	13′, 15′	12′, 13′, 15′, 16′	
15′	31.7, CH_2	1.23	14′, 16′	13′, 14′, 16′, 17′	
16′	22.6, CH_2	1.25	15′, 17′	14′, 15′, 17′	
17′	14.0, CH_3	0.84, t (6.9)	16′	15′, 16′	

*Indiscernible signals from overlap or complex multiplicity are reported without designating multiplicity.

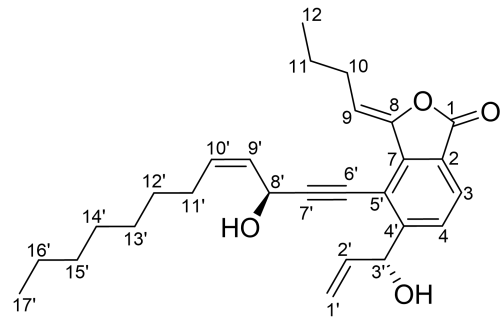

Table S2. NMR data of **1** in C_6D_6 (600 MHz for ^{1}H; 151 MHz for ^{13}C)

	**1**			
No.	δ_C, mult.	δ_H (J in Hz)*	^{1}H, ^{1}H-COSY	HMBC
1	166.3, qC			
2	124.7, qC			
3	126.0, CH	7.66, d (8.0)	4	1, 4, 7, 4′
4	126.7, CH	7.41, d (8.0)	3	1, 2, 3, 3′, 5′
7	139.1, qC			
8	145.8, qC			
9	114.1, CH	6.59, t (7.7)	10	7, 8, 11
10	28.8, CH_2	2.52	9, 11	8, 9, 11, 12
11	22.9, CH_2	1.57	10, 12	9, 10, 12
12	14.1, CH_3	0.97, t (7.4)	11	10, 11
1′	115.4, CH_2	5.06, dt (10.3, 1.2), Ha	1′b, 2′, 3′	3′
		5.47, dt (17.0, 1.2), Hb	1′a, 2′, 3′	2′, 3′
2′	138.7, CH	5.89, ddd (17.0, 10.3, 5.6)	1′a, 1′b, 3′	3′, 4′
3′	72.3, CH	5.64, br d (5.6)	1′a, 1′b, 2′	4, 1′, 2′, 4′, 5′
4′	152.3, qC			
5′	113.3, qC			
6′	79.2, qC			
7′	102.8, qC			
8′	59.1, CH	5.43, d (8.5)	9′	5′, 6′, 7′, 9′, 10′
9′	129.2, CH	5.80, ddt (10.4, 8.5, 1.2)	8′, 10′, 11′	7′, 8′, 11′
10′	133.6, CH	5.50	9′, 11′	8′, 11′, 12′
11′	28.1, CH_2	2.03	9′, 10′, 12′	9′, 10′, 12′, 13′
12′	29.7, CH_2	1.28	11′, 13′	10′, 11′, 13′, 14′
13′	29.5, CH_2	1.22	12′, 14′	11′, 12′, 14′, 15′
14′	29.6, CH_2	1.21	13′, 15′	12′, 13′, 15′, 16′
15′	32.2, CH_2	1.21	14′, 16′	13′, 14′, 16′, 17′
16′	23.0, CH_2	1.25	15′, 17′	14′, 15′, 17′
17′	14.3, CH_3	0.88, t (6.9)	16′	15′, 16′

*Indiscernible signals from overlap or complex multiplicity are reported without designating multiplicity.

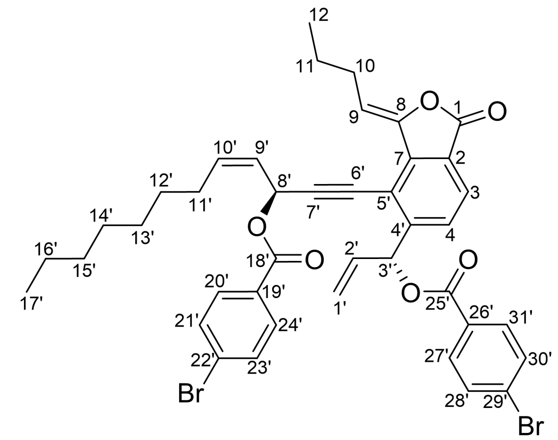

1a

Table S3. NMR data of **1a** in $CDCl_3$ (600 MHz for ^1H; 151 MHz for ^{13}C)

		**1a**		
No.	δ_C, mult.	δ_H (J in Hz)*	^1H, ^1H-COSY	HMBC
1	165.9, qC			
2	124.8, qC			
3	125.8, CH	7.87, d (8.0)	4	1, 7, 4′
4	127.0, CH	7.62, d (8.0)	3	1, 2, 3′, 5′
7	139.2, qC			
8	145.0, qC			
9	115.1, CH	6.51, t (7.9)	10	7, 8, 11
10	28.4, CH_2	2.43	9, 11	8, 9, 11, 12
11	22.5, CH_2	1.49	10, 12	9, 10, 12
12	13.9, CH_3	0.94, t (7.3)	11	10, 11
1′	118.5, CH_2	5.32, dt (10.6, 1.2), Ha	1′b, 2′, 3′	3′
		5.45, dt (17.1, 1.2), Hb	1′a, 2′, 3′	2′, 3′
2′	134.0, CH	6.08, ddd (17.1, 10.6, 6.0)	1′a, 1′b, 3′	3′, 4′
3′	74.5, CH	6.91, br d (6.0)	1′a, 1′b, 2′	4, 1′, 2′, 4′, 5′, 25′
4′	148.3, qC			
5′	113.9, qC			
6′	79.7, qC			
7′	98.7, qC			
8′	61.2, CH	6.64, d (8.3)	9′	5′, 6′, 7′, 9′, 10′, 18′
9′	123.5, CH	5.79	8′, 10′, 11′	7′, 8′, 11′
10′	137.2, CH	5.83	9′, 11′	8′, 11′, 12′
11′	28.1, CH_2	2.27	9′, 10′, 12′	9′, 10′, 12′, 13′
12′	29.2, CH_2	1.42	11′, 13′	10′, 11′, 13′, 14′
13′	29.2, CH_2	1.30	12′, 14′	11′, 12′, 14′, 15′
14′	29.1, CH_2	1.25	13′, 15′	12′, 13′, 15′, 16′
15′	31.7, CH_2	1.19	14′, 16′	13′, 14′, 16′, 17′
16′	22.6, CH_2	1.22	15′, 17′	14′, 15′, 17′
17′	14.0, CH_3	0.82, t (6.9)	16′	15′, 16′
18′	164.6, qC			
19′	128.5, qC			
20′, 24′	131.3, CH	7.94, d (8.5)	21′, 23′	18′, 22′
21′, 23′	131.8, CH	7.60, d (8.5)	20′, 24′	19′
22′	128.6, qC			
25′	164.3, qC			
26′	128.5, qC			
27′, 31′	131.2, CH	7.89, d (8.5)	28′, 30′	25′, 29′
28′, 30′	131.9, CH	7.57, d (8.5)	27′, 31′	26′
29′	128.6, qC			

*Indiscernible signals from overlap or complex multiplicity are reported without designating multiplicity.

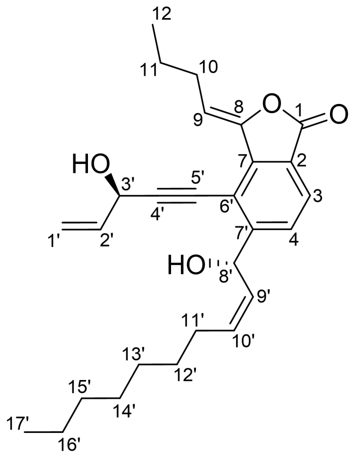

2

Table S4. NMR data of **2** in $CDCl_3$ (600 MHz for ^{1}H; 151 MHz for ^{13}C)

		**2**			
No.	δ_C, mult.	δ_H (J in Hz)*	^{1}H, ^{1}H-COSY	HMBC	NOESY
1	166.5, qC				
2	124.0, qC				
3	125.9, CH	7.88, d (8.1)	4	1, 4, 7, 7′	
4	126.9, CH	7.71, d (8.1)	3	1, 2, 3, 6′, 8′	
7	139.0, qC				
8	145.1, qC				
9	114.9, CH	6.43, t (7.7)	10	7, 8, 11	3′
10	28.3, CH_2	2.47	9, 11	8, 9, 11, 12	
11	22.4, CH_2	1.55	10, 12	9, 10, 12	
12	13.9, CH_3	0.99, t (7.4)	11	10, 11	
1′	117.3, CH_2	5.34, dt (10.3, 1.2), Ha	1′b, 2′, 3′	3′	
		5.56, dt (17.0, 1.2), Hb	1′a, 2′, 3′	2′, 3′	
2′	136.1, CH	6.09, ddd, (17.0, 10.3, 5.6)	1′a, 1′b, 3′	3′, 4′	
3′	63.8, CH	5.20, br d (5.6)	1′a, 1′b, 2′	1′, 2′, 4′, 5′, 6′	9
4′	100.1, qC				
5′	80.4, qC				
6′	112.7, qC				
7′	153.1, qC				
8′	67.6, CH	5.98, d (8.6)	9′	4, 6′, 7′, 9′, 10′	11′a, 11′b
9′	129.9, CH	5.52, ddt (10.5, 8.6, 1.2)	8′, 10′, 11′a, 11′b	7′, 8′, 10′, 11′	
10′	134.6, CH	5.60	9′, 11′a, 11′b	8′, 9′, 11′, 12′	
11′	28.2, CH_2	2.17, Ha	9′, 10′, 11′b, 12′	9′, 10′, 12′, 13′	8′
		2.35, Hb	9′, 10′, 11′a, 12′	9′, 10′, 12′, 13′	8′
12′	29.3, CH_2	1.37	11′a, 11′b, 13′	10′, 11′, 13′, 14′	
13′	29.2, CH_2	1.28	12′, 14′	11′, 12′, 14′, 15′	
14′	29.5, CH_2	1.26	13′, 15′	12′, 13′, 15′, 16′	
15′	31.8, CH_2	1.25	14′, 16′	13′, 14′, 16′, 17′	
16′	22.6, CH_2	1.28	15′, 17′	14′, 15′, 17′	
17′	14.0, CH_3	0.87, t (7.0)	16′	15′, 16′	

*Indiscernible signals from overlap or complex multiplicity are reported without designating multiplicity.

2. Preparation of the 3',8'-bis-*O*-(4-bromobenzoyl) derivative of 1 (1a)

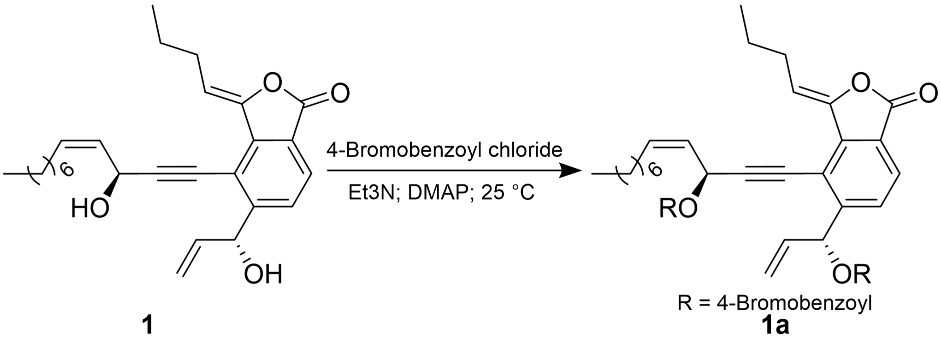

1 **1a**

Preparation of the 3',8'-bis-*O*-(4-bromobenzoyl) derivative of **1** (**1a**) was performed according to the method described by Scott E. Denmark et al.[1-2] with minor modifications. **1** (10.0 mg), triethylamine (7 μl), and dimethylaminopyridine (DMAP) (10.0 mg) were dissolved in CH_2Cl_2 (5 mL). Then, 4-bromobenzoyl chloride (15.0 mg) was slowly added to the above mixture (**1**, trimethylamine, DMAP, and CH_2Cl_2) at three times for 15 min at 25 °C. The mixture was diluted with H_2O (20 mL) and then extracted with CH_2Cl_2 (20 mL) three times. The CH_2Cl_2 layer was concentrated under reduced pressure, then subjected to open silica-gel CC, which was eluted with Petroleum ether–EtOAc (100:0, 0:100) to yield **1a** (12.1 mg)

3. Structural characterizations of 1−4

Falcarinphthalide A (**1**): yellow oil; $[\alpha]_D^{27}$ +158.6 (*c* 0.5, $CHCl_3$); UV (MeOH) λ_{max} (logε) 208 (4.26), 255 (4.47), 325 (3.54) nm； IR (KBr) ν_{max} 3421, 2959, 2927, 1766, 1596, 1462, 1273, 1019, 729 cm^{-1}; HRESIMS (positive) *m/z* 423.2533 $[M + H]^+$ (calcd. for $[C_{27}H_{35}O_4]^+$, 423.2535); CD (4.1×10^{-4} M, MeOH) λ_{max} (△*ε*): 254 (+21.18), 291 (+1.88) nm;.^{1}H and ^{13}C NMR see **Table S1** and **Table S2**.

3',8'-bis-*O*-(4-bromobenzoyl) derivative of 1 (1a): white powder; $[\alpha]_D^{27}$ -90.1 (*c* 0.5, $CHCl_3$); UV (MeOH) λ_{max} (logε) 203 (4.68), 252 (4.75), 294 (3.81) nm; IR (KBr) ν_{max} 2927, 2855, 1783, 1726, 1590, 1483, 1398, 1262, 756, 728 cm^{-1}; HRESIMS (positive) *m/z* 789.1235 $[M + H]^+$ (calcd. for $C_{41}H_{41}O_6Br^{81}Br$, 789.1245); ^{1}H and ^{13}C NMR data see **Table S3**.

Falcarinphthalide B (**2**): yellow oil; $[\alpha]_D^{27}$ +79.4 (*c* 0.5,$CHCl_3$); UV (MeOH) λ_{max} (logε) 202 (4.34), 258 (4.73), 329 (3.74)nm； IR (KBr) ν_{max} 3438, 2956, 2927, 2852, 1783, 1758, 1463, 1092, 1019, 729 cm^{-1}; HRESIMS (positive) *m/z* 423.2534 $[M + H]^+$ (calcd. for $[C_{27}H_{35}O_4]^+$, 423.2535); CD (2.1×10^{-4} M, MeOH) λ_{max} (△*ε*): 217 (+1.12), 231 (+4.92), 260 (-15.4) nm;.^{1}H and ^{13}C NMR see **Table S4**.

(3*R*, 8*S*)-falcarindiol (**3**): yellow oil; $[\alpha]_D^{27}$ +250.6 (*c* 0.5,$CHCl_3$); HRESIMS (positive) *m/z* 259.1704 $[M - H]^-$ (calcd. for $[C_{17}H_{23}O_2]^-$, 259.1693); ^{1}H NMR ($CDCl_3$) *δ* 5.93 (ddd, H-2, *J* = 16.9, 10.1, 5.5 Hz, 1H), 5.60 (dt, H-10, *J* = 10.5, 7.4 Hz, 1H), 5.48 (m, H-1a, H-9, 2H), 5.25 (br d, H-1b, *J* = 10.2 Hz, 1H), 5.19 (br d, H-8, *J* = 8.2 Hz, 1H), 4.93 (br d, H-3, *J* = 5.3 Hz, 1H), 2.10 (m, H-11, 2H), 1.37 (m, H-12, 2H), 1.27 (m, H-13 ~ H-16, 8H), 0.87 (t, H-17, *J* = 6.7 Hz, 3H); ^{13}C NMR ($CDCl_3$), 117.3 (C-1), 135.8 (C-2), 63.4 (C-3), 79.8 (C-4), 70.2 (C-5), 68.7 (C-6), 78.3 (C-7), 58.5 (C-8), 127.6 (C-9), 134.6 (C-10), 27.6 (C-11), 29.2 (C-12), 29.1 (C-13), 29.1 (C-14), 31.8 (C-15), 22.6 (C-16), 14.1 (C-17).[3]

(*Z*)-ligustilide (**4**): yellow oil; HRESIMS (positive) *m/z* 191.1065 $[M + H]^+$ (calcd. for $[C_{12}H_{15}O_2]^+$, 191.1067); ^{1}H NMR ($CDCl_3$) *δ* 6.28 (br d, H-3, *J* = 9.6 Hz, 1H), 5.99 (dt, H-4, *J* = 9.6, 4.5 Hz, 1H), 2.45 (m, H-5, 2H), 2.59 (m, H-6, 2H), 5.21 (t, H-9, *J* = 7.8 Hz, 1H), 2.37 (q, H-10, *J* = 7.8 Hz, 2H), 1.49 (m, H-11, 2H), 0.95 (t, H-12, *J* = 7.3 Hz, 3H); ^{13}C NMR ($CDCl_3$), 167.6 (C-1), 124.0 (C-2), 117.2 (C-3), 129.9 (C-4), 22.4 (C-5), 18.5 (C-6), 148.6 (C-7), 147.0 (C-8), 112.9 (C-9), 28.1 (C-10), 22.4 (C-11), 13.8 (C-12).[4]

4. Quantum chemical ECD calculations of 1′–2′

4.1 Quantum chemical ECD calculations of 1′

The molecules of (3'*R*, 8'*S*)–**1′** and (3'*R*, 8'*R*)–**1′** were converted into SMILES codes before their initial 3D structures were generated with CORINA version 3.4. Conformer databases were generated in CONFLEX version 7.0 by using the MMFF94s force-field, with an energy window for acceptable conformers (ewindow) of 5 kcal/mol above the ground state, a maximum number of conformations per molecule (maxconfs) of 300, and an RMSD cutoff (rmsd) of 0.5Å. Then each acceptable conformer was optimized with HF/6-31G(d) method in Gaussian09.[5] Further optimization at the B3LYP/TZVP level determined the dihedral angles. From this, (17 for (3'*R*, 8'*S*)–**1′** and 14 for (3'*R*, 8'*R*)–**1′**) stable conformers (**Figure S1** and **Figure S2**) were determined. The optimized conformers were used for the ECD calculations, which were performed with Gaussian09 (B3LYP/TZVP). The solvent effects were taken into account by the polarizable-conductor calculation model (PCM, MeOH as the solvent). Comparisons of the experimental and calculated spectra (**Figure S3**) were performed with the software SpecDis.[6-7] This was also used to apply a UV shift to the ECD spectra, Gaussian broadening of the excitations, and Boltzmann weighting (**Table S5** and **Table S6**) of the spectra.

Table S5. Stable conformers of (3'*R*, 8'*S*)–**1′** at the B3LYP/TZVP level in MeOH

conformers	contribution %	conformers	contribution %
1	11.51	10	4.74
2	9.35	11	4.73
3	8.00	12	4.27
4	7.21	13	4.05
5	6.92	14	3.96
6	6.82	15	3.91
7	6.68	16	3.77
8	5.92	17	2.85
9	5.30		

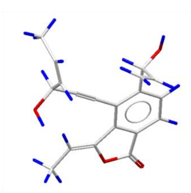
C1 (11.51)

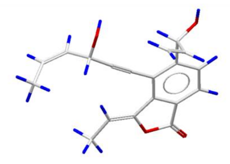
C2 (9.35)

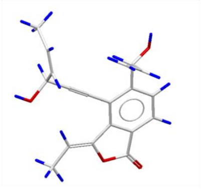
C3 (8.00)

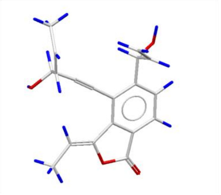
C4 (7.21)

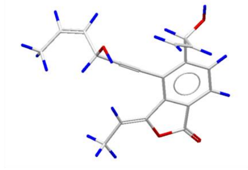
C5 (6.92)

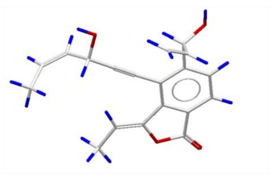
C6 (6.82)

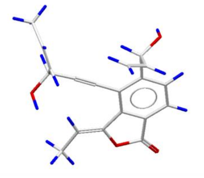
C7 (6.68)

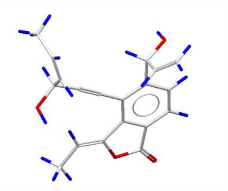
C8 (5.92)

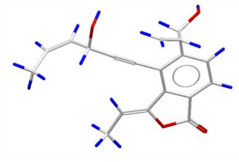
C9 (5.30)

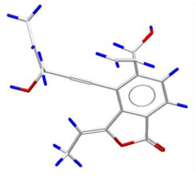
C10 (4.74)

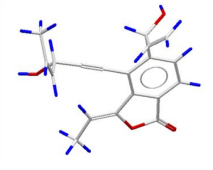
C11 (4.73)

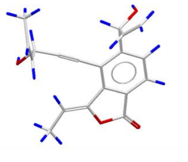
C12 (4.27)

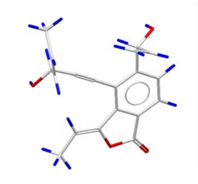
C13 (4.05)

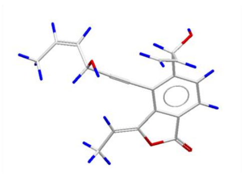
C14 (3.96)

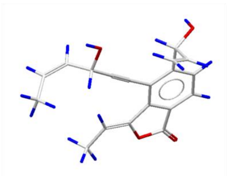
C15 (3.91)

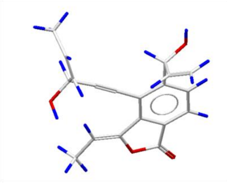
C16 (3.77)

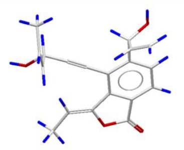
C17 (2.85)

Figure S1. Stable conformers of (3'*R*, 8'*S*)–**1′** (the relative populations are in parentheses)

Table S6. Stable conformers of (3'*R*, 8'*R*)–**1′** at the B3LYP/TZVP level in MeOH

conformers	contribution %	conformers	contribution %
1	13.19	8	6.53
2	11.84	9	6.35
3	8.44	10	5.26
4	8.37	11	4.82
5	7.32	12	4.76
6	7.26	13	4.72
7	7.15	14	4.00

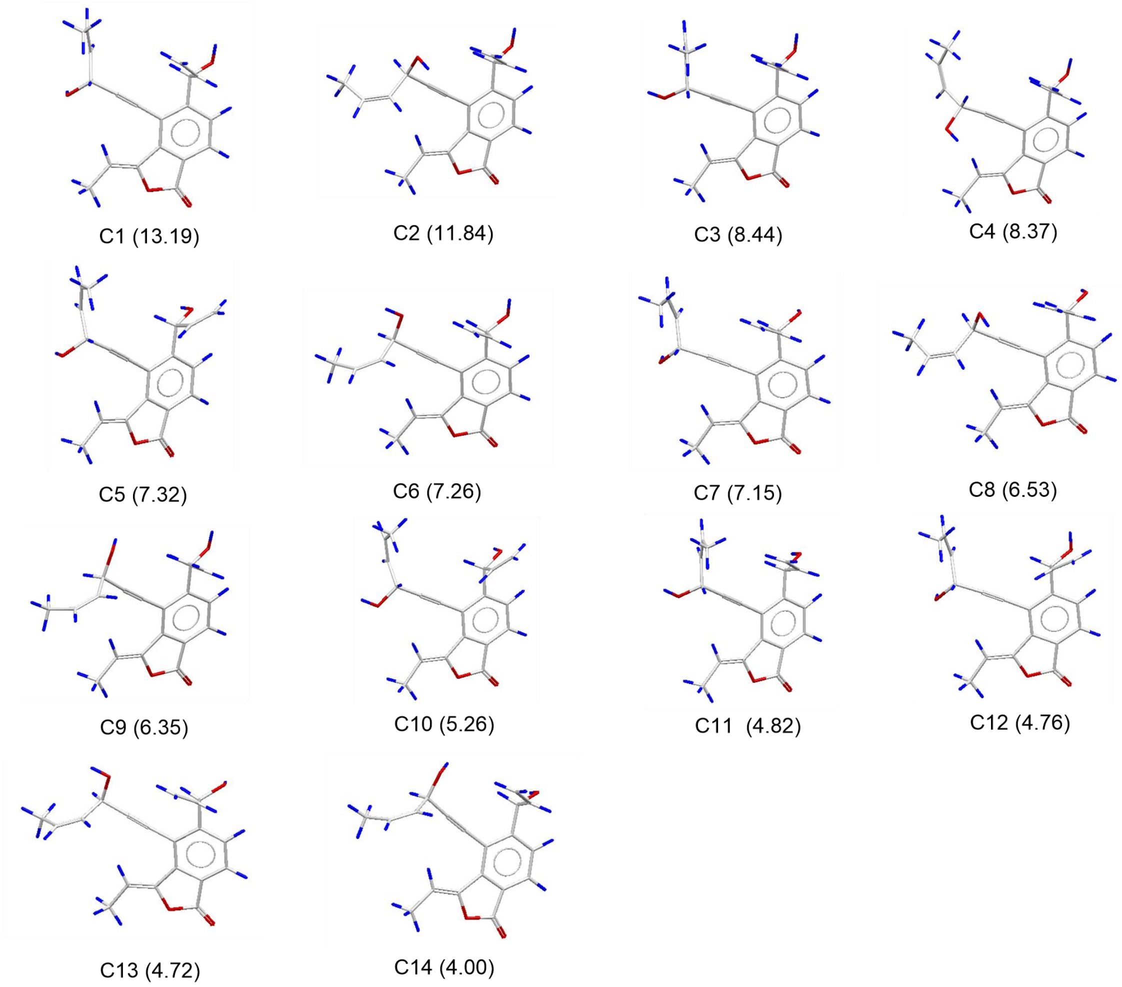

Figure S2. Stable conformers of (3'*R*, 8'*R*)–**1′** (the relative populations are in parentheses)

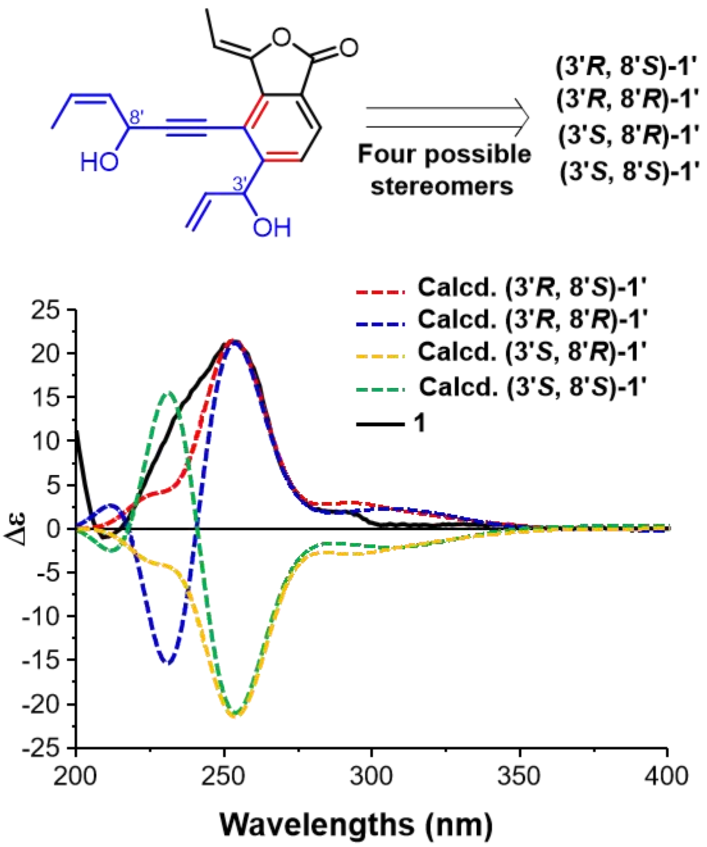

Figure S3. Experimental ECD spectrum of **1**, and calculated ECD spectra of (3'*R*, 8'*S*)–**1′**, (3'*S*, 8'*R*)–**1′**, (3'*S*, 8'*S*)–**1′**, and (3'*R*, 8'*R*)–**1′** (UV correction = –18 nm, band width σ = 0.3 eV)

4.2 Quantum chemical ECD calculations of 2′

The molecules of (3'*R*, 8'*S*)–**2′** and (3'*R*, 8'*R*)–**2′** were converted into SMILES codes before their initial 3D structures were generated with CORINA version 3.4. Conformer databases were generated in CONFLEX version 7.0 by using the MMFF94s force-field, with an energy window for acceptable conformers (ewindow) of 5 kcal/mol above the ground state, a maximum number of conformations per molecule (maxconfs) of 300, and an RMSD cutoff (rmsd) of 0.5Å. Then each acceptable conformer was optimized with HF/6-31G(d) method in Gaussian09.[5] Further optimization at the wb97XD/TZVP level determined the dihedral angles. From this, (27 for (3'*R*, 8'*S*)–**2′** and 15 for (3'*R*, 8'*R*)–**2′**) stable conformers (**Figure S4** and **Figure S5**) were determined. The optimized conformers were used for the ECD calculations, which were performed with Gaussian09 (wb97XD/TZVP). The solvent effects were taken into account by the polarizable-conductor calculation model (PCM, MeOH as the solvent). Comparisons of the experimental and calculated spectra (**Figure S6**) were performed with the software SpecDis.[6-7] This was also used to apply a UV shift to the ECD spectra, Gaussian broadening of the excitations, and Boltzmann weighting (**Table S7** and **Table S8**) of the spectra.

Table S7. Stable conformers of (3'*R*, 8'*S*)–**2′** at the wb97XD/TZVP level in MeOH

conformers	contribution %	conformers	contribution %
1	10.48	15	2.81
2	6.56	16	2.80
3	5.90	17	2.55
4	5.27	18	2.52
5	4.63	19	2.51
6	4.31	20	2.23
7	4.22	21	2.22
8	3.86	22	2.17
9	3.56	23	1.89
10	3.32	24	1.71
11	3.23	25	1.50
12	2.91	26	1.36
13	2.87	27	1.13
14	2.83		

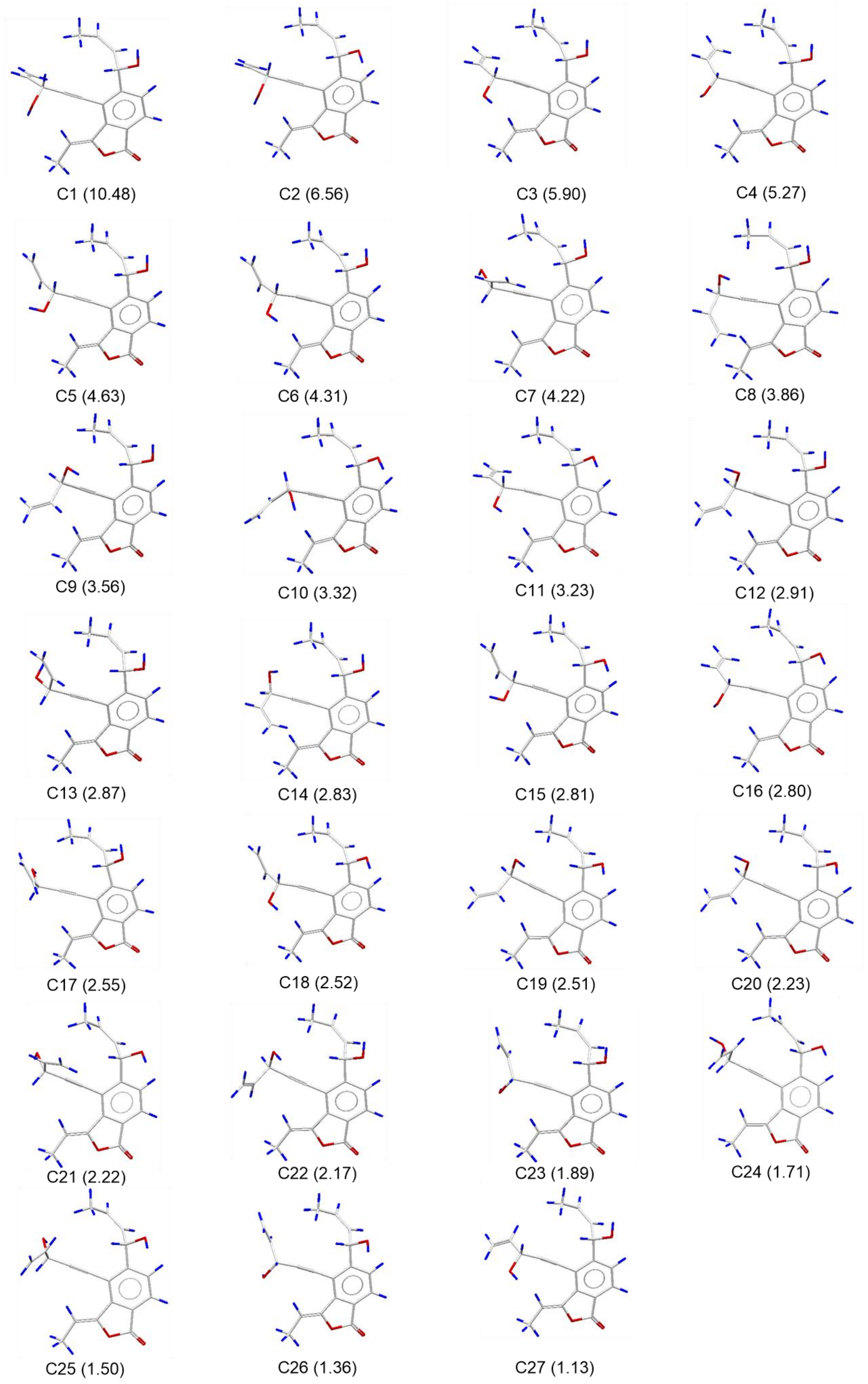

Figure S4. Stable conformers of (3'*R*, 8'*S*)–**2′** (the relative populations are in parentheses)

Table S8. Stable conformers of (3'*R*, 8'*R*)–**2′** at the wb97XD/TZVP level in MeOH

conformers	contribution %	conformers	contribution %
1	14.17	9	6.17
2	10.69	10	5.16
3	10.56	11	4.49
4	9.73	12	3.41
5	7.86	13	2.67
6	7.59	14	2.18
7	6.68	15	1.98
8	6.65		

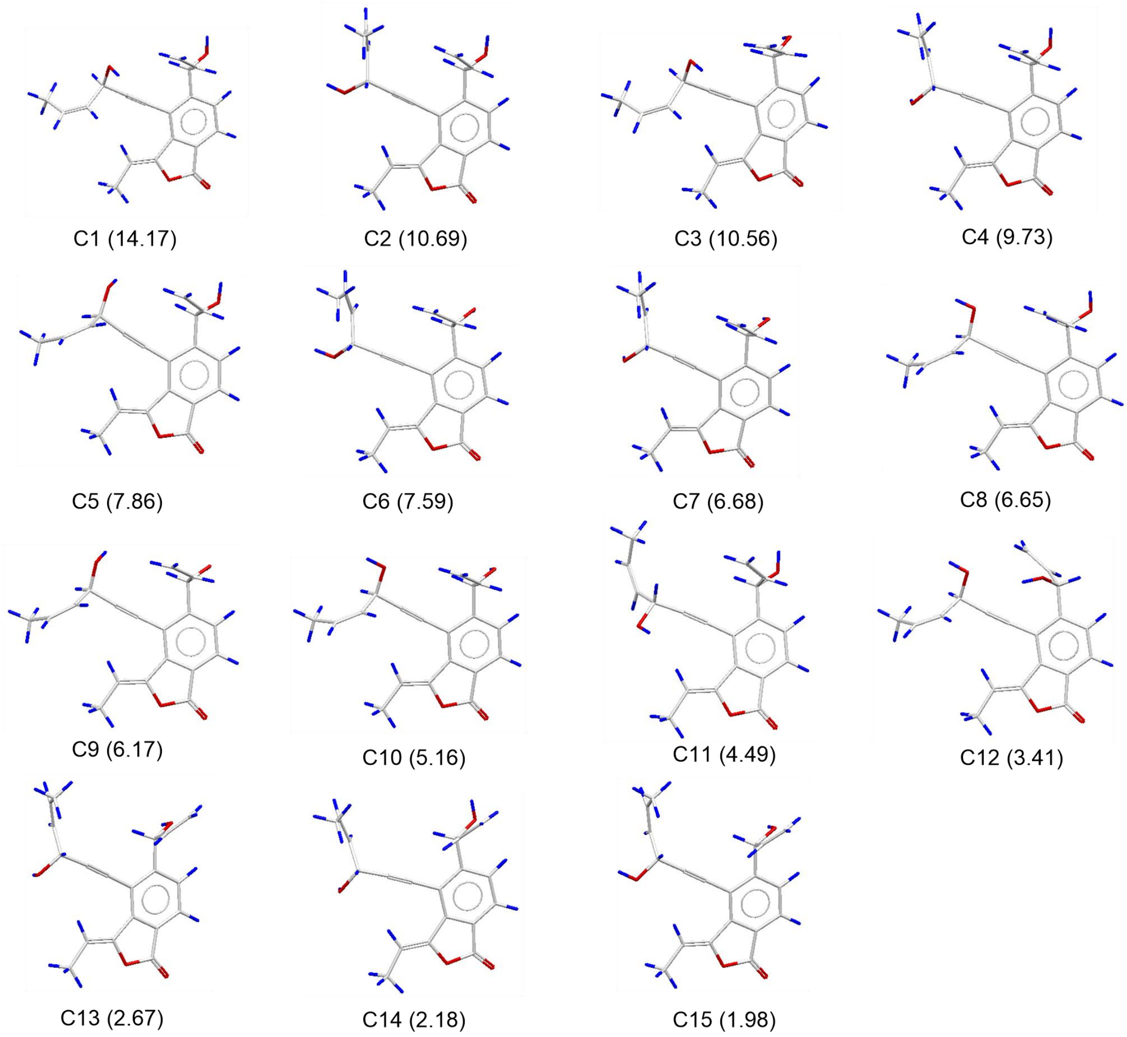

Figure S5. Stable conformers of (3'*R*, 8'*R*)–**2′** (the relative populations are in parentheses)

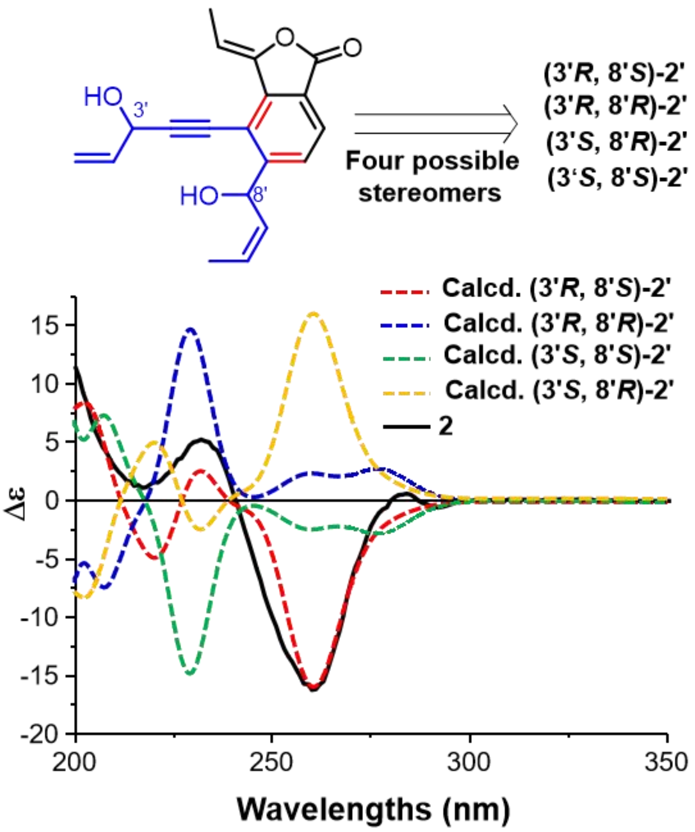

Figure S6. Experimental ECD spectrum of **2**, and calculated ECD spectra of (3'*R*, 8'*S*)–**2′**, (3'*S*, 8'*R*)–**2′**, (3'*S*, 8'*S*)–**2′**, and (3'*R*, 8'*R*)–**2′** (UV correction = –15 nm, band width σ = 0.16 eV)

5. Quantum chemical VCD calculations of 1′a

The IR and VCD spectrum of **1a** in $CDCl_3$ was measured at a resolution of 4 cm^{-1} using Synchrocell (2.75 sec per cycle) on a BioTools ChiralIR-2X VCD spectrometer equipped with a cell (150 mm in optical length) with BaF_2 windows. The ZnSe photoelastic modulator of the instrument was set to 1400 cm^{-1}.

The molecules of (3'*R*,8'*S*)-**1'a** and (3'*R*,8'*R*)-**1'a** were converted into SMILES codes before their initial 3D structures were generated with CORINA version 3.4. Conformer databases were generated in CONFLEX version 7.0 using the MMFF94s force-field, with an energy window for acceptable conformers (ewindow) of 5 kcal/mol above the ground state, a maximum number of conformations per molecule (maxconfs) of 300, and an RMSD cutoff (rmsd) of 0.5Å. Then each acceptable conformer was optimized with HF/6-31G(d) method in Gaussian09. [5] Further optimization at the B3LYP/6-31G+(d) level determined the dihedral angles. From this, (31 for (3'*R*,8'*S*)-**1'a** and 28 for (3'*R*,8'*R*)-**1'a**) stable conformers (**Figure S7** and **Figure S8**) were determined. The optimized conformers of (3'*R*,8'*S*)-**1'a** and (3'*R*,8'*R*)-**1'a** were used for the VCD calculations, which were performed with Gaussian09 (B3LYP/6-311+G (2d,p)). The solvent effects were taken into account by the polarisable-conductor calculation model (PCM, $CHCl_3$ as the solvent). Comparisons of the experimental and calculated IR and VCD spectra (**Figure S9**) were performed with the software Origin. Calculated frequencies were empirically scaled using a 0.9984 scaling factor, and boltzmann weighting (**Table S9** and **Table S10**) of the spectra.

Table S9. Stable conformers of (3'*R*, 8'*S*)–**1′a** at the B3LYP/6-31G+(d) level in $CHCl_3$

conformers	contribution %	conformers	contribution %
1	11.91	17	2.75
2	6.72	18	2.74
3	6.71	19	2.10
4	6.71	20	2.06
5	5.38	21	1.94
6	4.70	22	1.80
7	3.82	23	1.80
8	3.41	24	1.79
9	3.24	25	1.78
10	3.03	26	1.56
11	3.03	27	1.55
12	2.98	28	1.42
13	2.85	29	1.35
14	2.85	30	1.26
15	2.75	31	1.26
16	2.75		

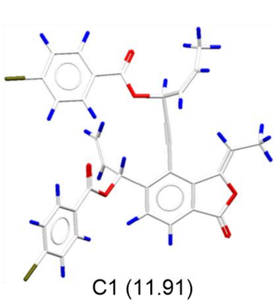

C1 (11.91)

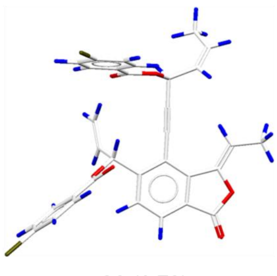

C2 (6.72)

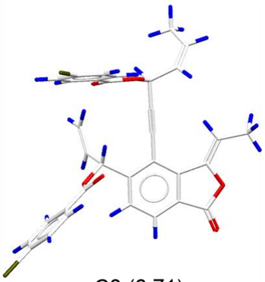

C3 (6.71)

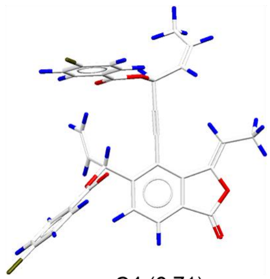

C4 (6.71)

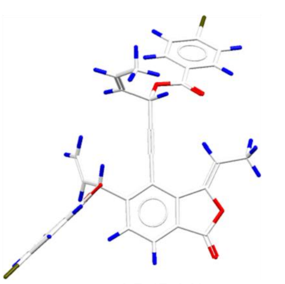

C5 (5.38)

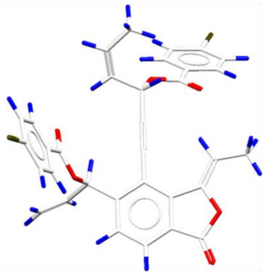

C6 (4.70)

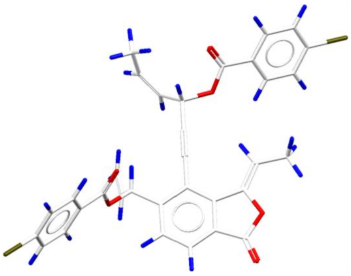

C7 (3.82)

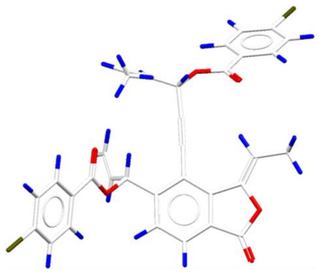

C8 (3.41)

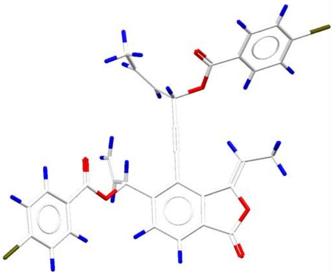

C9 (3.24)

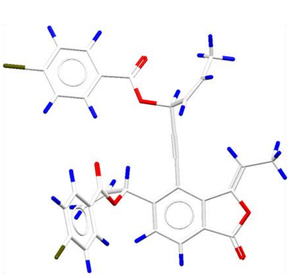

C10 (3.03)

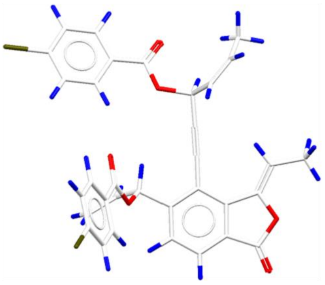

C11 (3.03)

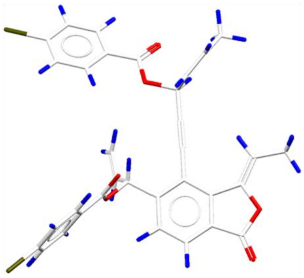

C12 (2.98)

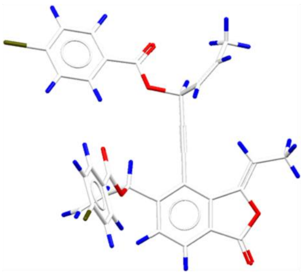

C13 (2.85)

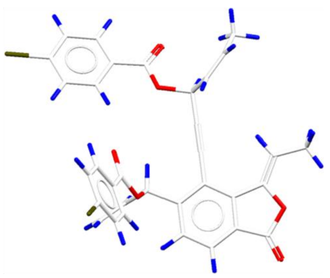

C14 (2.85)

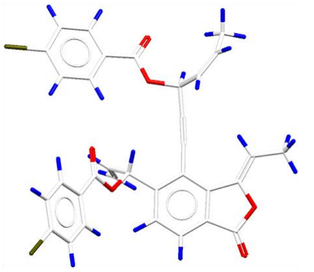

C15 (2.75)

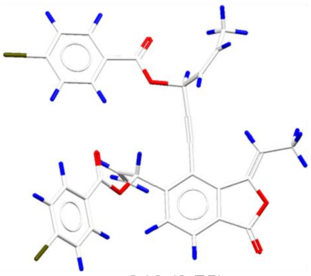

C16 (2.75)

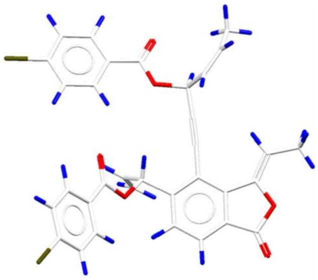

C17 (2.75)

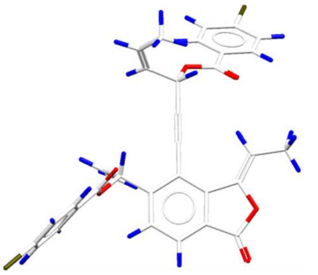

C18 (2.74)

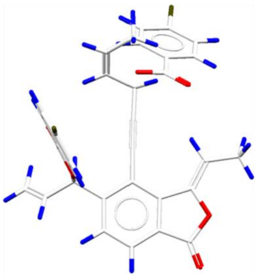

C19 (2.10)

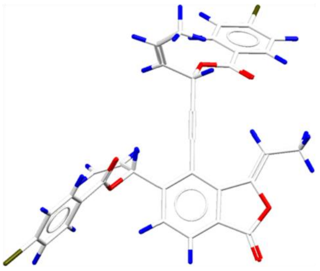

C20 (2.06)

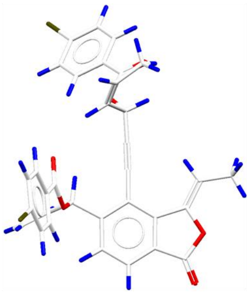

C21 (1.94)

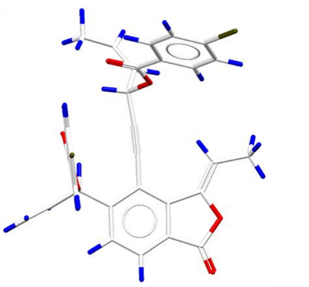

C22 (1.80)

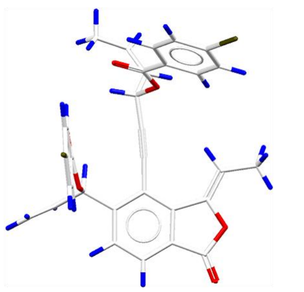

C23 (1.80)

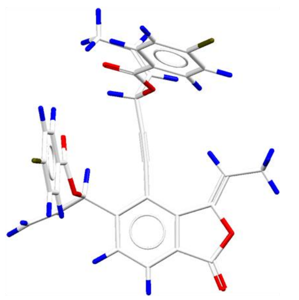

C24 (1.79)

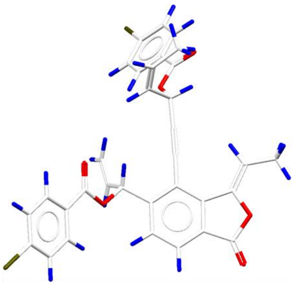

C25 (1.78)

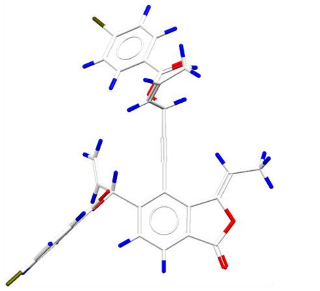

C26 (1.56)

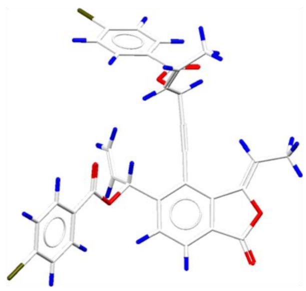

C27 (1.55)

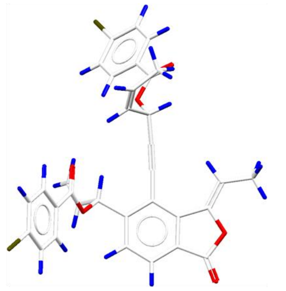

C28 (1.42)

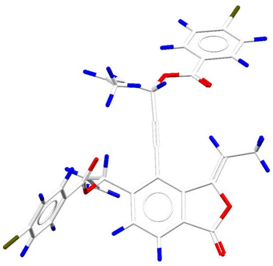

C29 (1.35)

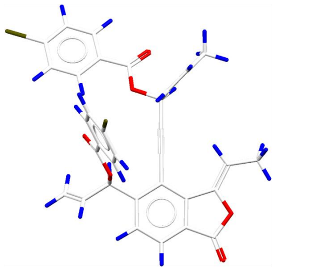

C30 (1.26)

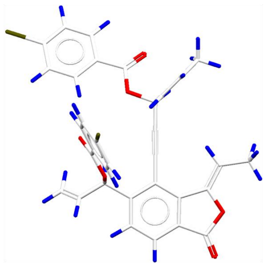

C31 (1.26)

Figure S7. Stable conformers of (3'*R*, 8'*S*)–**1′a** (the relative populations are in parentheses)

Table S10. Stable conformers of (3'*R*, 8'*R*)–**1′a** at the B3LYP/6-31G+(d) level in $CHCl_3$

conformers	contribution %	conformers	contribution %
1	8.28	15	2.69
2	8.27	16	2.63
3	8.07	17	2.37
4	7.92	18	2.16
5	7.91	19	1.99
6	7.13	20	1.84
7	3.59	21	1.84
8	3.59	22	1.81
9	3.42	23	1.81
10	3.27	24	1.76
11	2.89	25	1.72
12	2.71	26	1.68
13	2.70	27	1.68
14	2.70	28	1.56

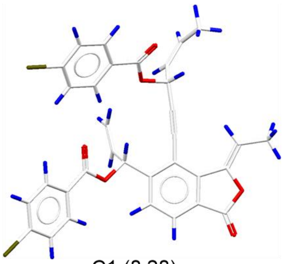
C1 (8.28)

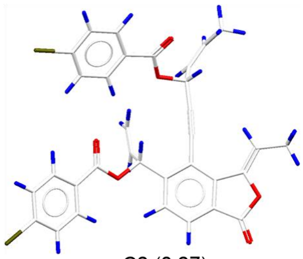
C2 (8.27)

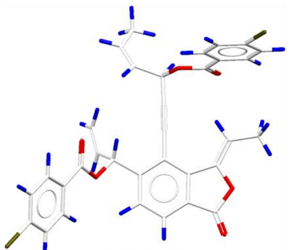
C3 (8.07)

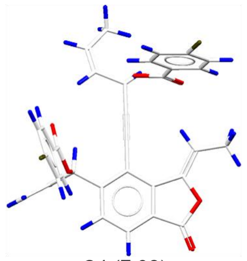
C4 (7.92)

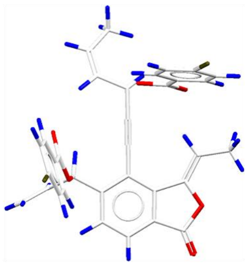
C5 (7.91)

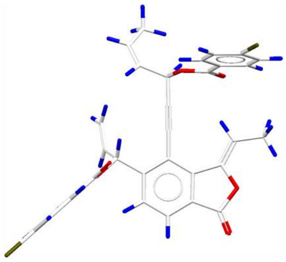
C6 (7.13)

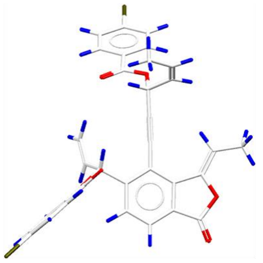
C7 (3.59)

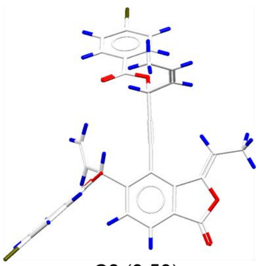
C8 (3.59)

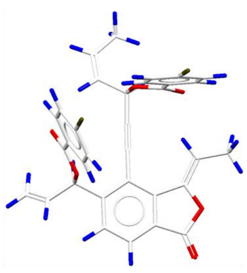
C9 (3.42)

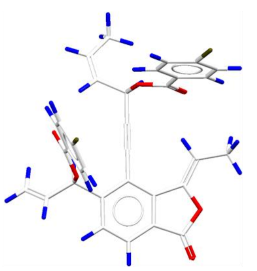
C10 (3.27)

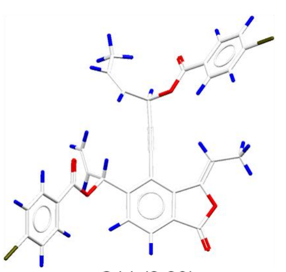
C11 (2.89)

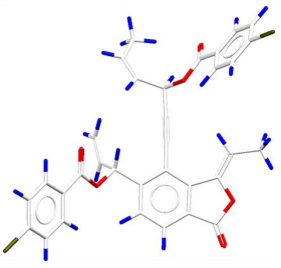
C12 (2.71)

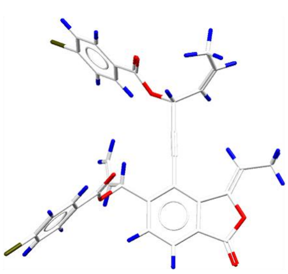
C13 (2.70)

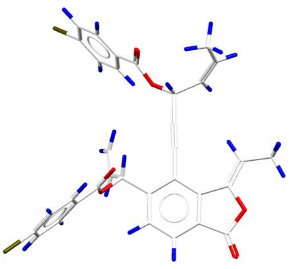
C14 (2.70)

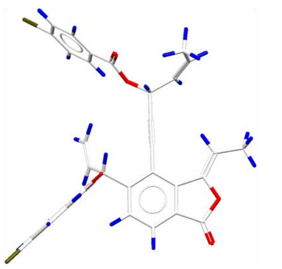
C15 (2.69)

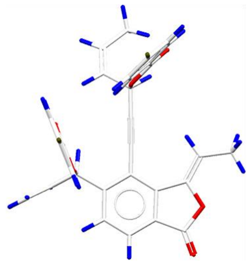
C16 (2.63)

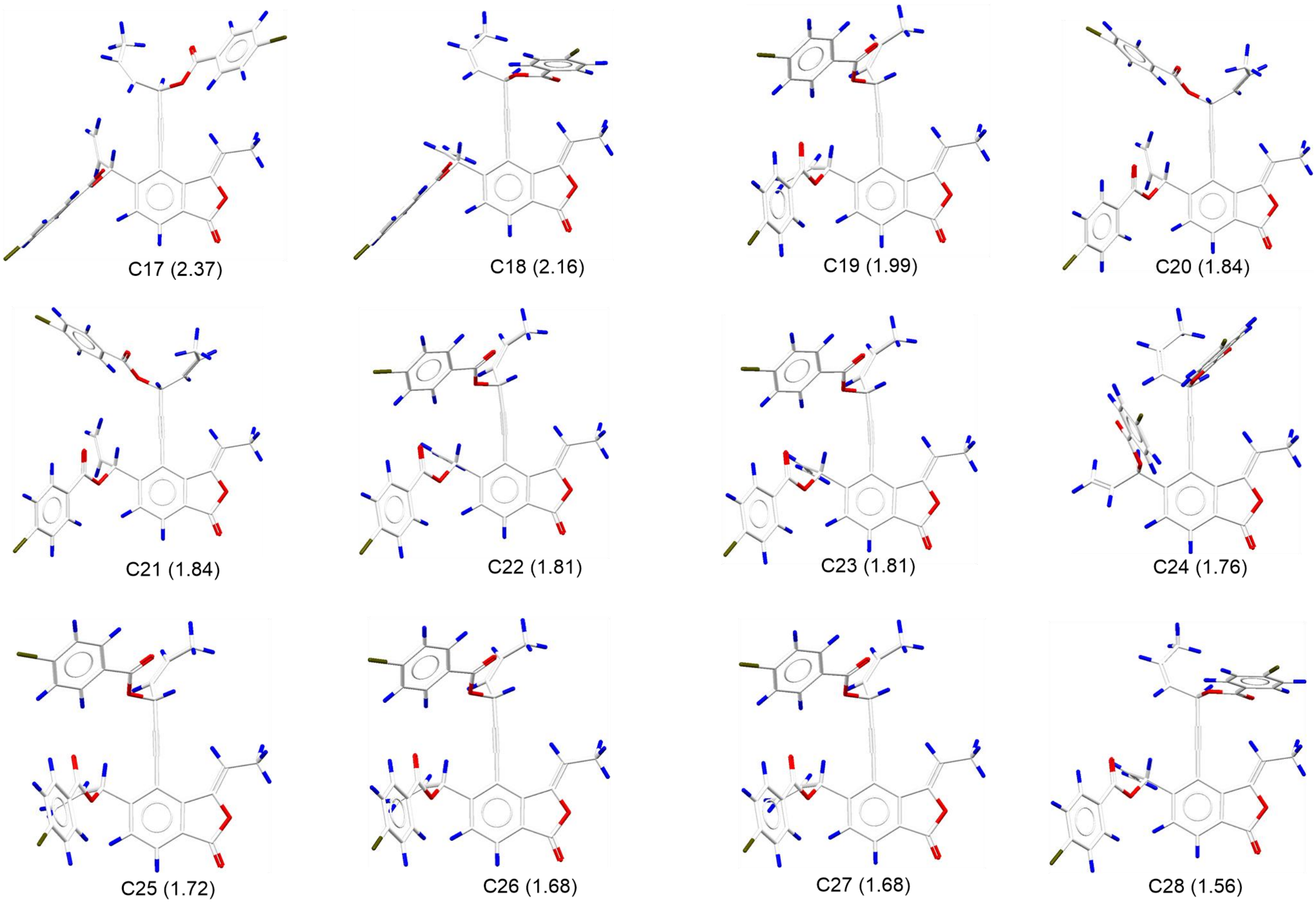

Figure S8. Stable conformers of (3'*R*, 8'*R*)–**1′a** (the relative populations are in parentheses)

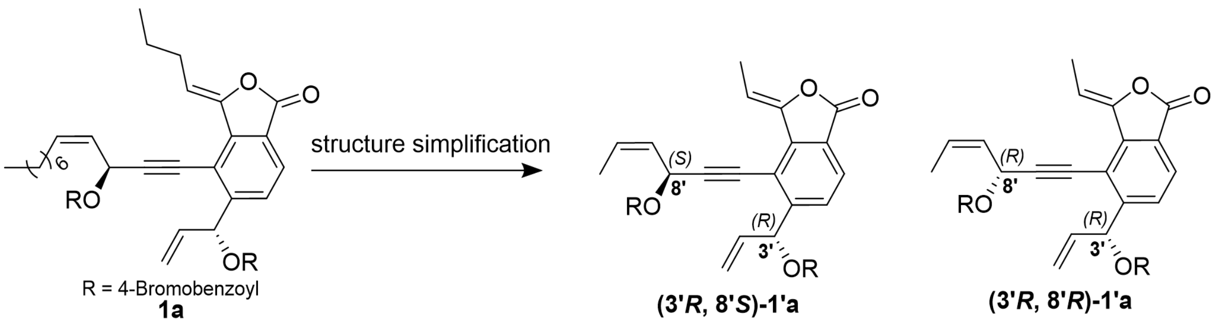

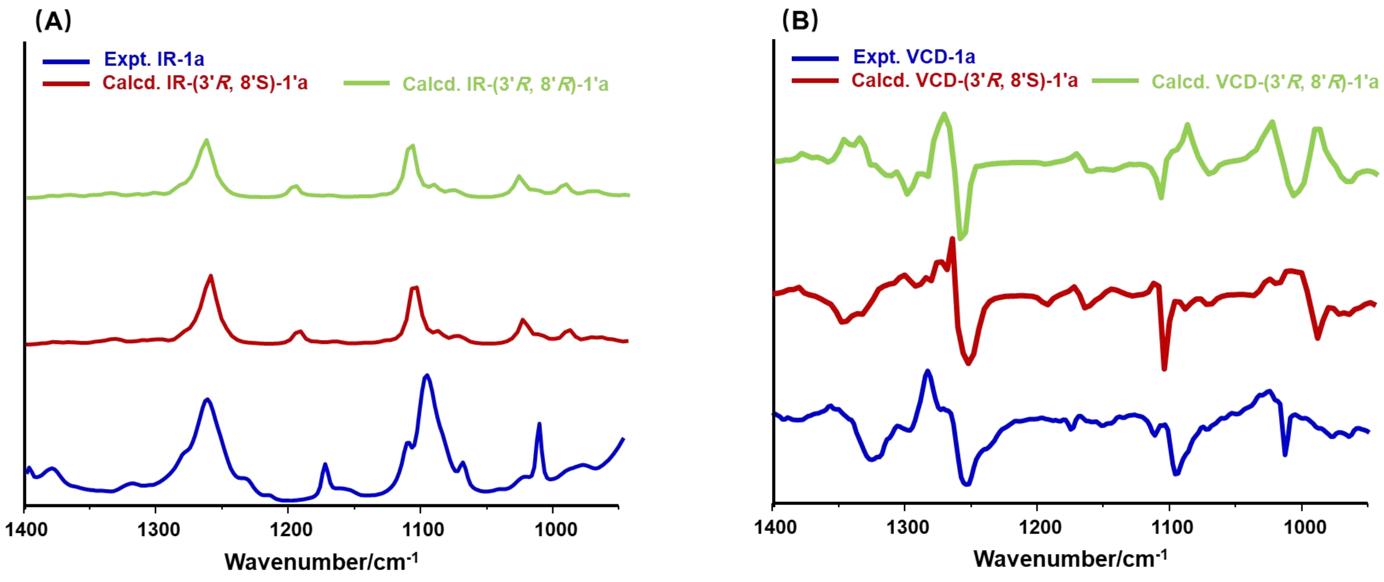

Figure S9. (**A**) Experimental IR spectrum of **1a**, and calculated IR spectra of (3'*R*, 8'*S*) –**1′a** and (3'*R*, 8'*R*) –**1′a** in the $CDCl_3$ solution; (**B**) Experimental VCD spectrum of **1a**, and calculated VCD spectra of (3'*R*, 8'*S*) –**1′a** and (3'*R*, 8'*R*) –**1′a** in the $CDCl_3$ solution.

6. Anti-osteoclastogenic activity assays of 1−4

6.1 Materials and methods

For immunoblotting analysis, the following antibodies were used: rabbit monoclonal anti-Integrin-β3 (#13166, dilution 1:1000), anti-NFATc1 (#8032, dilution 1:1000), monoclonal anti-NF-κB p65(#8242, dilution 1:1000), polyclonal anti-c-Fos(#4384, 1:500) were purchased from Cell Signaling Technology (Beverly, MA, USA), Phalloidin-iFluor 555 was purchased from Abcam (USA, #176756). Murine sRANKL and M-CSF were the products of Peprotech (Rocky Hill, NJ, USA).

6.2 Cell culture

RAW 264.7 cells (ATCC, USA) were routinely cultured in modified Eagle medium alpha (α-MEM), supplemented with 10% fetal bovine serum (FBS), penicillin 100 U/mL and streptomycin 100 μg/mL at 37°C in a humidified atmosphere of 5% CO_2. Cells were cultured in a new culture dish every three days. Cells used for the experiments were of passage 8-15.

6.3 Cytotoxicity assay

Cell cytotoxicity was measured using CCK-8 assay. RAW264.7 cells were seeded at 3,000 cells/well in 96 well plates. After 24 h of incubation, cells were treated with vehicle (0.1% DMSO) as control group (C), pamidronate (20 μM), and different concentrations of compounds 1-4 (0.1, 1, 5, and 10 μM) . After 4 days, CCK-8 solution was added, and cells were incubated at 37°C for 2 h. Finally, absorbance was measured at 450 nm. All compounds showed no cytotoxic effect on RAW264.7 cells at different concentrations (0.1, 1, 5, and 10 *μ*M) (**Figure S10**).

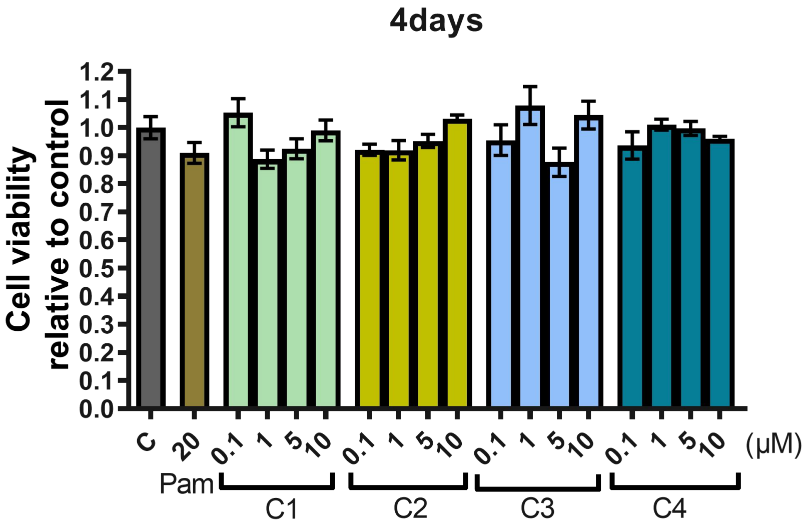

Figure S10. The viability of RAW264.7 cells was determined at 4 days after treatment with or without **1-4** under normal α-MEM medium for 4 days. The data were acquired by three independent experiments and are expressed as mean±SD. **P* < 0.05, ***P* < 0.01, ****P* < 0.001 *vs.* Control. Pam: pamidronate.

6.4 Osteoclast differentiation and TRAP staining

RAW264.7 cells were seeded at 3,000 cells/well in 96 well plates. After 24 h of incubation, cells were treated with vehicle (0.1% DMSO) as control group (C), pamidronate (20 μM), and different concentrations of compounds **1**-**4** (1, 5, and 10 μM) in the absent or present of osteoclast differentiation medium. After 4 days, TRAP staining was performed according to our previous method.[8] The number and spread area of multinucleated osteoclasts per well was quantified under microscopy using Image-Pro plus 6.0 software (Media Cybernetics, Silver Spring, MD, USA).

6.5 Immunofluorescence staining for F-actin ring formation of osteoclasts

RAW264.7 cells were treated with vehicle (0.1% DMSO, NC and C group), pamidronate (20 μM), Compounds **1**-**4** (10 μM) in the absent or present of osteoclast differentiation medium for 4 days. Cells were incubated with Phalloidin-iFluor 555 (Abcam, USA) to label the F-actin ring according to a previously described method.[9] After F-actin ring staining, cells were washed three times with PBS followed by staining nuclei with DAPI (Beyotime Biotechnology, China) for 5 min, F-actin rings were visualized using a fluorescence microscope (Leica, DMI8) and confocal microscope (Nikon, A1R). Six wells were selected in each group to calculate the size of the F-actin ring cells using Image-Pro plus 6.0 software (Media Cybernetics, Silver Spring, MD, USA).

6.6 Resorption pit assay

RAW264.7 cells were seeded at 3000 cells/well onto the Corning® Osteo Assay Surface 96 well multiple well plate (Corning Incorporated Life Science, NY, USA). After 24 h of incubation, cells were treated with vehicle (0.1% DMSO, NC and C group), pamidronate (20 μM), Compounds **1**-**4** (10 μM) in the absent or present of osteoclast differentiation medium. The drug treatment medium was replaced every 2 days. At day 7, The cells were removed by treating each well with 5% sodium hypochlorite for 5 minutes and wash with water and dry. The wells were imaged with microscopy (Olympus IX71, USA) to identify resorbed pits. The area of bone resorbed pits was quantified using Image-Pro Plus 6.0 software (Media Cybernetics, Silver Spring, MD, USA).

6.7 RT-PCR

RAW264.7 cells was seeded at 3,0000 cells/well onto 12 well plates, after 24 h of incubation, cells were treated with treatment of vehicle (0.1% DMSO, NC and C group), pamidronate (20 μM), Compounds **1**-**4** (10 μM) for 2 days in the absent or present of osteoclast differentiation medium. At day 2, total RNA was extracted using RNeasy kit (Qiagen, USA) according to the manufacturer's instructions. The reverse transcription was performed using PrimeScript™ RT Master Mix (TaKaRa, Japan). The primers sequences used in the assay were listed in **Table S11**. The 10 μL of the final reaction solution contained 1 μL of the diluted cDNA product, 5 μL of 2×TB Green Premix Ex Taq II (TaKaRa, Japan), 0.5 μL each of forward and reverse primers and 3 μL nuclease free water. The amplification conditions and procedures were as the following: 50°C for 2 min, 95°C for 10 min, 40 cycles of 95°C for 15 sec, 60°C for 1 min. The fluorescence signal was recorded by Roche Light Cycler 480 Detection System, and then the signal was converted into numerical values. Relative gene expression was determined by employing the Comparative CT-method. The mRNA levels of all genes were normalized by β-actin as internal control. These analyses were performed in duplicates for each sample using cells from two different cultured wells, and each experiment was repeated three times.

Table S11. The sequence of primers used in quantitative RT-PCR

Gene	Forward primers(5'-3')	Reverse primers(5'-3')
m-NFATc1	TCCAAAGTCATTTTCGTGGA	CTTTGCTTCCATCTCCCAGA
m-DC-STAMP	CTTGCA ACCTAAGGGCAAAG	TCAACAGCTCTGTCGTGACC
m-OSCAR	GGGGTAACGGATCAGCTCCCCAGA	CCAAGGAGCCAGAACGTCGAAACT
m-TRAP	ACACAGTGATGCTGTGTGGCAACTC	CCAGAGGCTTCCACATATATGATGG
m-β-actin	ACTGCTCTGGCTCCTAGCAC	CCACCGATCCACACAGAGTA

6.8 Western Blot

RAW264.7 cells were seeded at 100,000 cells/well in 6 well plates. After 24 h, cells were treated with vehicle (1% DMSO), pamidronate (20 μM), compounds **1-4** (10 μM) in the absence or presence of osteoclast differentiation medium. Following that cells were homogenized in RIPA lysis buffer containing 1% protease inhibitor cocktail (Roche, USA) and 1% phosphate inhibitor cocktail (Roche, USA). The total protein of cell lysates was quantified with BCA protein assay kit (Beyotime Biotechnology, China). PageRuler Prestained Protein Ladder (00758179, Thermo Fisher, USA) was used as a loading marker. Equal amounts of protein (40-80 μg) in each sample were separated by 10 % SDS-polyacrylamide gel electrophoresis (SDS-PAGE) and transferred to polyvinylidene fluoride (PVDF) membranes (Millipore) at 300 mA for 70 minutes, following blocked with 5% milk in TBST and probed with primary antibodies and subsequently HRP AffiniPure Goat Anti-rabbit or Anti-mouse lgG (H+L) (1:2000 dilution, EarthOx, USA) as secondary antibodies. Band signals were detected using Millipore Immobilon Western chemiluminescent horseradish peroxidase substrate (Millipore Corporation, Billerica, MA, USA), followed by analysis with Tanon 5200—Chemiluminescent Imaging System (Bio-Tanon, Shanghai, China) and quantitated using Image J analysis software (Wayne Rasband, NIH, USA). Relative protein expressions were normalized to the GAPDH expression level.

6.9 Immunofluorescence staining of NF-κB nuclear translocation

The effect of compounds **1-4** on nuclear localization of NF-κB (P65) was determined by immunofluorescence assays. RAW264.7 cells were seeded on glass slides (Lab-Tek chambered#1.0 Borosilicate Coverglass System, 155411) at a density of 8,000 per well for 24h. After cells were induced with 100 ng RANKL for 1 h with or without pretreatment with 10 μM of compounds **1-4** for 4 h. Subsequently, cells were rinsed with PBS for 3 times and fixation in 4% formaldehyde for 30 min at 4℃. Following that, cells were permeabilized for 10 min in 0.1% Triton X-100 in PBS. After a brief washing in PBS, cells were blocked with 5% bovine serum albumin at 4℃ for 1h and then incubated with rabbit anti-p65 antibody (#8242, 1:250) overnight. Cells were then incubated with Alexa Fluor 488-labeled Goat Anti-Rabbit lgG (H+L) (Beyotime, A0423) for 2 h and stained with DAPI for 10 min. The nuclear translocation of NF-κB(P65) was examined under a confocal microscope (Nikon, A1R). The mean per-pixel fluorescence intensity of NF-κB P65 in the nucleus were measured in each cell, the the MFI/nucleus was calculated for each image by image J-Pro plus software, data was presented as MFI ±SD of NF-κB P65/nucleus and representative of three independent experiments.

6.10 Statistical analyses

Each experiment was performed at least 3 times, and all quantitative data are presented as the mean ± standard deviation (SD). The analysis of variance (ANOVA), followed by the Tukey posthoc test, was used to compare the parameters among groups. $P < 0.05$ was considered statistically significant.

7. The related operation of LC-HR-ESI-MS analysis

The fresh plant material (50 g) was extracted using cooling extraction twice with 95% ethanol/water (500 mL) for 2 hours each time in the dark place. After evaporation under reduced pressure, the extract was obtained. The extract and compounds **1** and **2** were analyzed directly by LC-HR-ESI-MS (**Figure S11–S14**).

Chromatography was performed using a Dionex Ultimate 3000 UPLC system (Thermo Fisher Scientific). The separation was performed on a Phenomenex Gemini C_{18} column (21.2 × 250 mm, 5 μm, Phenomenex Inc.). The analysis was conducted on the gradient elution component using (A) methanol and (B) water as the mobile phase at 1.0 mL/min. The gradient of 0−2 min, 10% A, 2−20 min, 10−60% A, 20−50 min, 60−80% A, 50−60 min, 80−100% A. The injection volume of the sample solution was 10 μL. Mass spectrometry was carried out on a Q-executive hybrid-quadrupole-orbitrap mass spectrometer (Thermo Fisher Scientific) equipped with an ESI interface and controlled by Xcalibur 4.0 software (Thermo Fisher Scientific). Mass spectra were acquired in positive ion mode with a mass range of 100−1500.

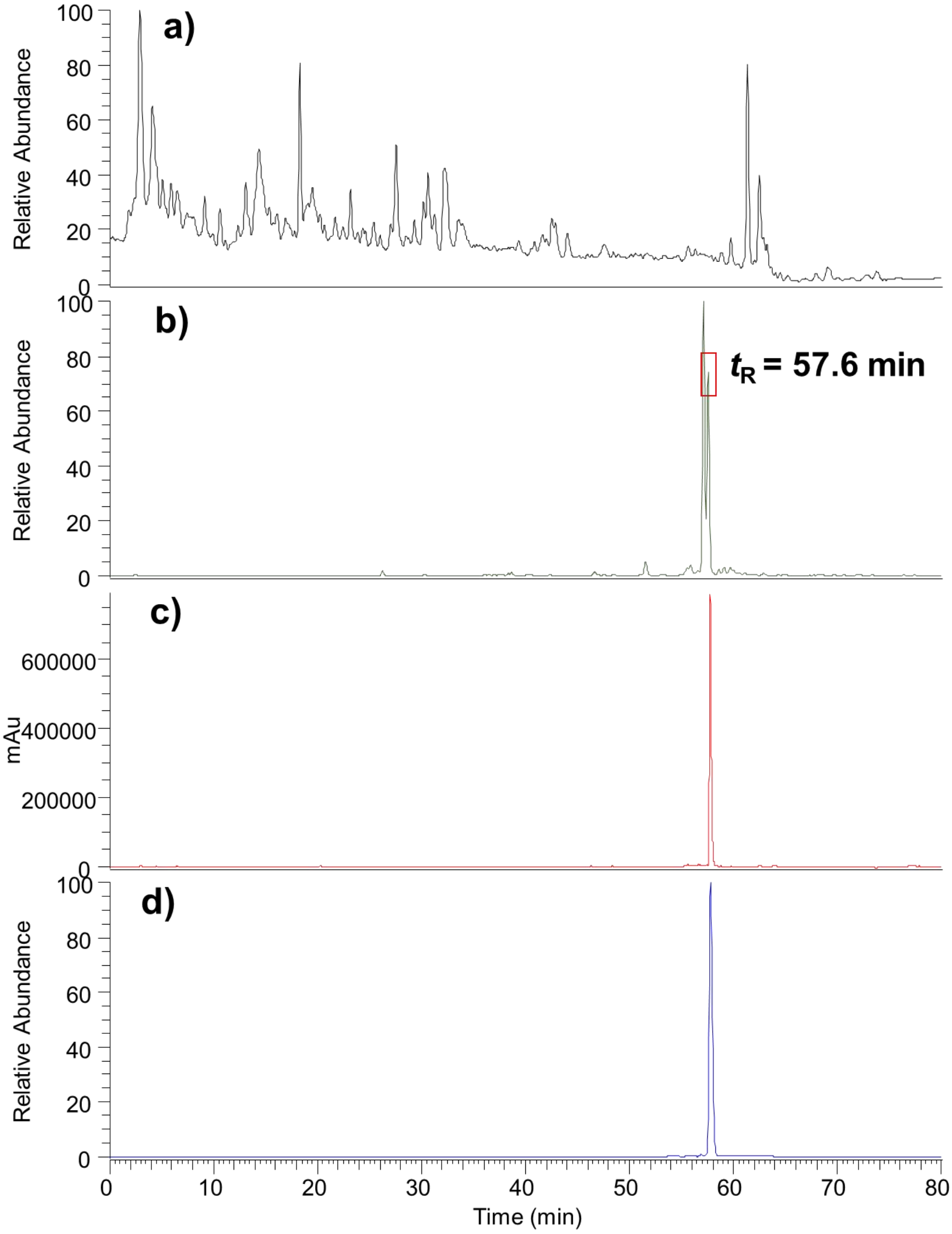

Figure S11. a) the (-)-TIC (Total Ion Chromatogram) of the extract; b) the extracted ion chromatogram of *m/z* 421.2389 of the extract; c) the LC-UV spectrum of compound **1** at 254 nm; d) the extracted ion chromatogram of *m/z* 421.2385 of compound **1**.

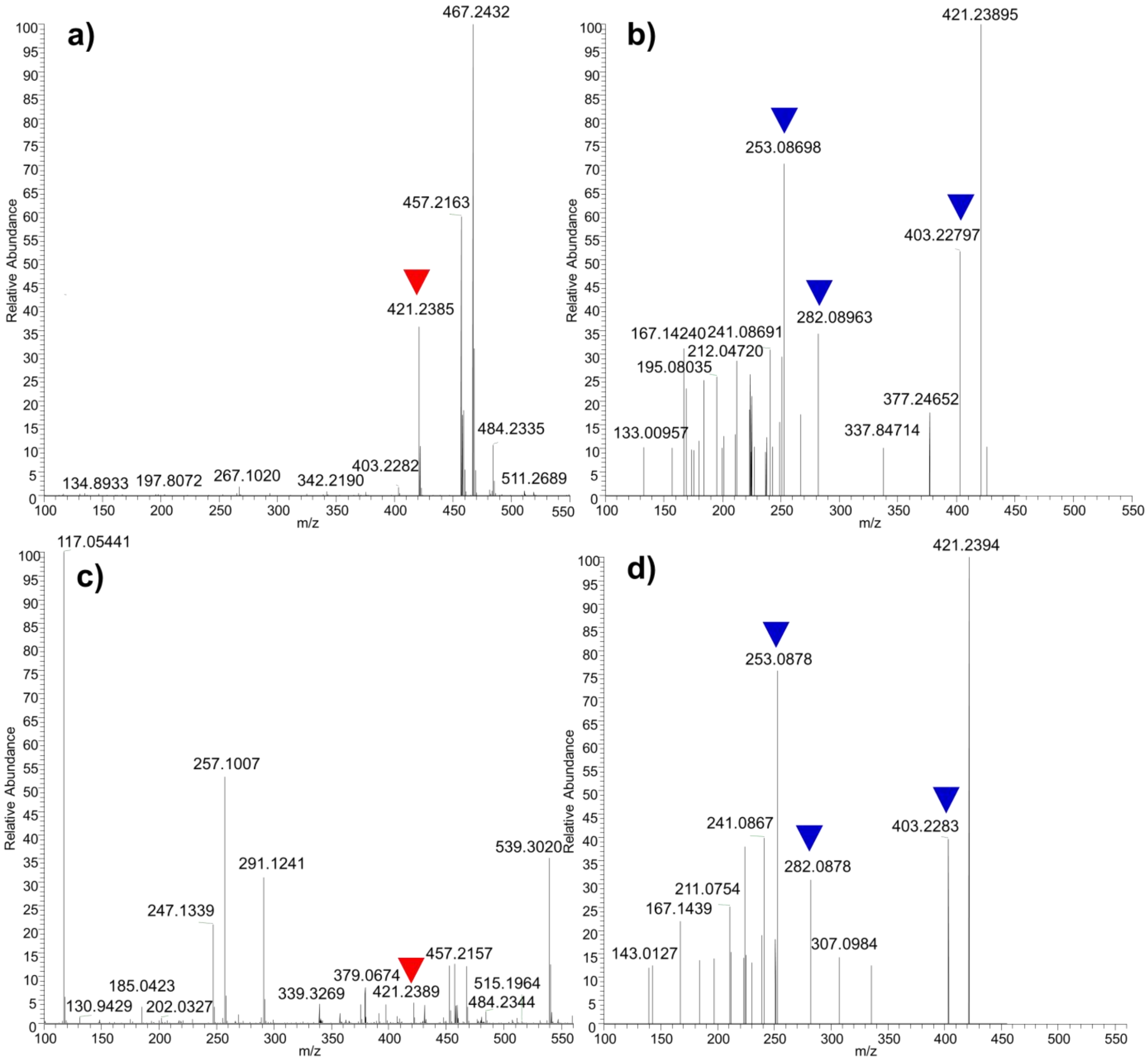

Figure S12. a) the MS spectrum of the compound **1** ([M-H]$^-$ = 421.2385, $C_{27}H_{33}O_4$); b) the MS^2 spectrum of *m/z* 421.2385; c) the MS spectrum of the peak at t_R = 57.6 min ([M-H]$^-$ = 421.2389, $C_{27}H_{33}O_4$) in the extract; d) the MS^2 spectrum of *m/z* 421.2389

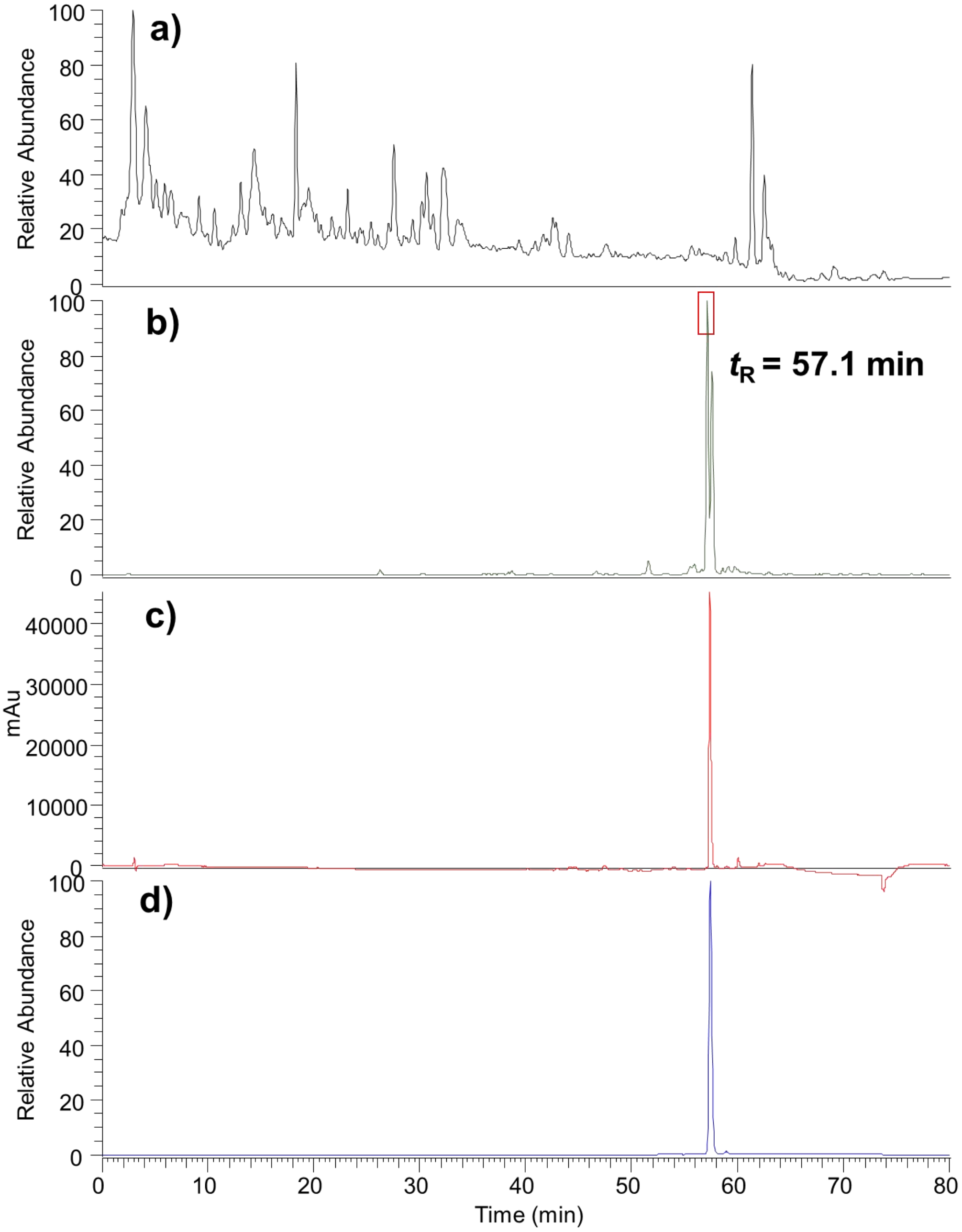

Figure S13. a) the (-)-TIC (Total Ion Chromatogram) of the extract; b) the extracted ion chromatogram of *m/z* 421.2389 of the extract; c) the LC-UV spectrum of compound **2** at 254 nm; d) the extracted ion chromatogram of *m/z* 421.2383 of compound **2**

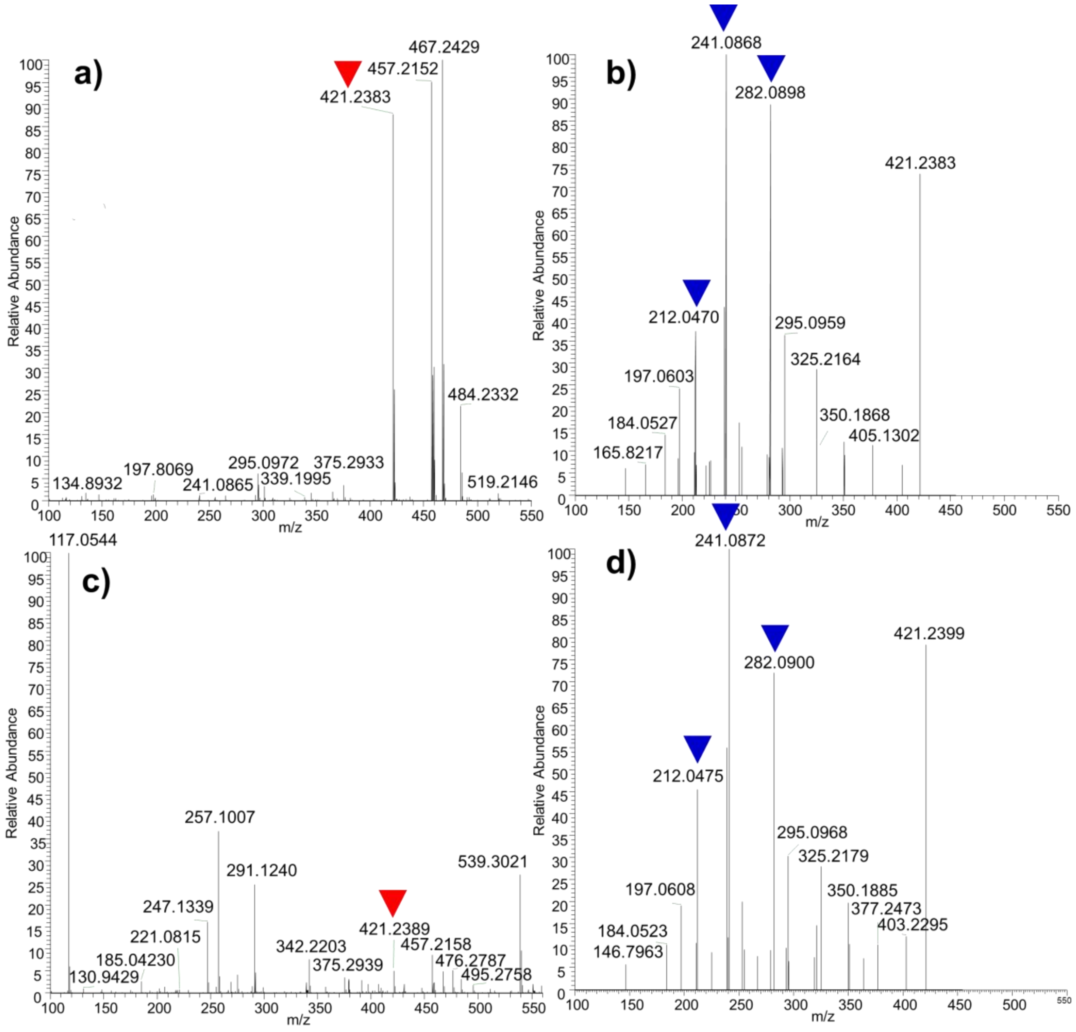

Figure S14. a) the MS spectrum of the compound **2** ([M-H]$^-$ = 421.2383, $C_{27}H_{33}O_4$); b) the MS^2 spectrum of *m/z* 421.2383; c) the MS spectrum of the peak at t_R = 57.1 min ([M-H]$^-$ = 421.2389, $C_{27}H_{33}O_4$) in the extract; d) the MS^2 spectrum of *m/z* 421.2389

8. The DFT calculation details of chemical reactions

The molecular geometries of the complexes were optimized using density functional theory (DFT) calculations at the B3LYP/6-31G(d) level.[10] Frequency calculations were also performed at the same level of theory to identify all the stationary points as minima (zero imaginary frequencies) or transition states (one imaginary frequency), and the free energies at 298.15 K. An IRC[11-12] analysis were performed to confirm that all the stationary points were smoothly connected to each other. A single point energy calculation at the M062X/6–311+G(d,p) level[13] based on each optimized geometry was carried out. The Gibbs free energy was calculated from the electron energy of M062X/6–311+G(d,p) level plus the thermal correction of B3LYP/6-31G(d) level. All calculations were performed using the Gaussian 09 package.[5]

8.1 Possible reaction pathways

Two possible reaction pathways (Path A and Path B) were proposed. The path A start from (3*R*, 8*S*)-falcarindiol (**3**) and (*Z*)-ligustilide (**4**), then through Diels-Alder/Retro-Diels-Alder reactions to produce **1**. The path B start from (3*R*, 8*S*)-falcarindiol (**3**) and (*Z*)-butylidenephthalide, then through the same reactions to produce **1**.

As **Figure** (**S15─S16**) shown, the path B produce **1** via **TS_1'** and **TS_2'**. Due to the energy barriers of path B were 45.8 kcal/mol and 36.9 kcal/mol, and higher than **TS_1** and **TS_2**. Therefore, path B was ruled out. Compound **2** through the same reaction of the formation of compound **1**.(**Figure S17**)

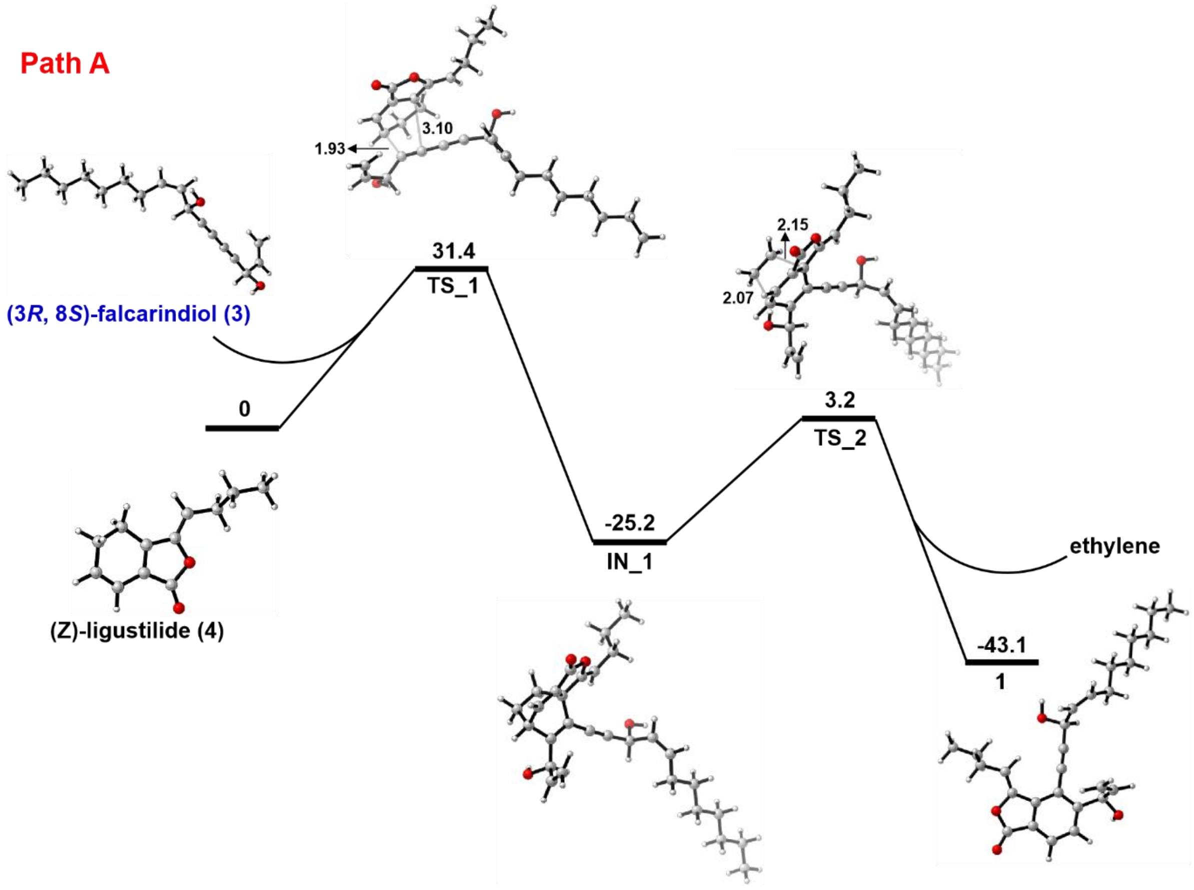

Figure S15. DFT calculations for path A; relative energies from M062X/6–311+G(d,p)//B3LYP/6–31G(d) are shown.

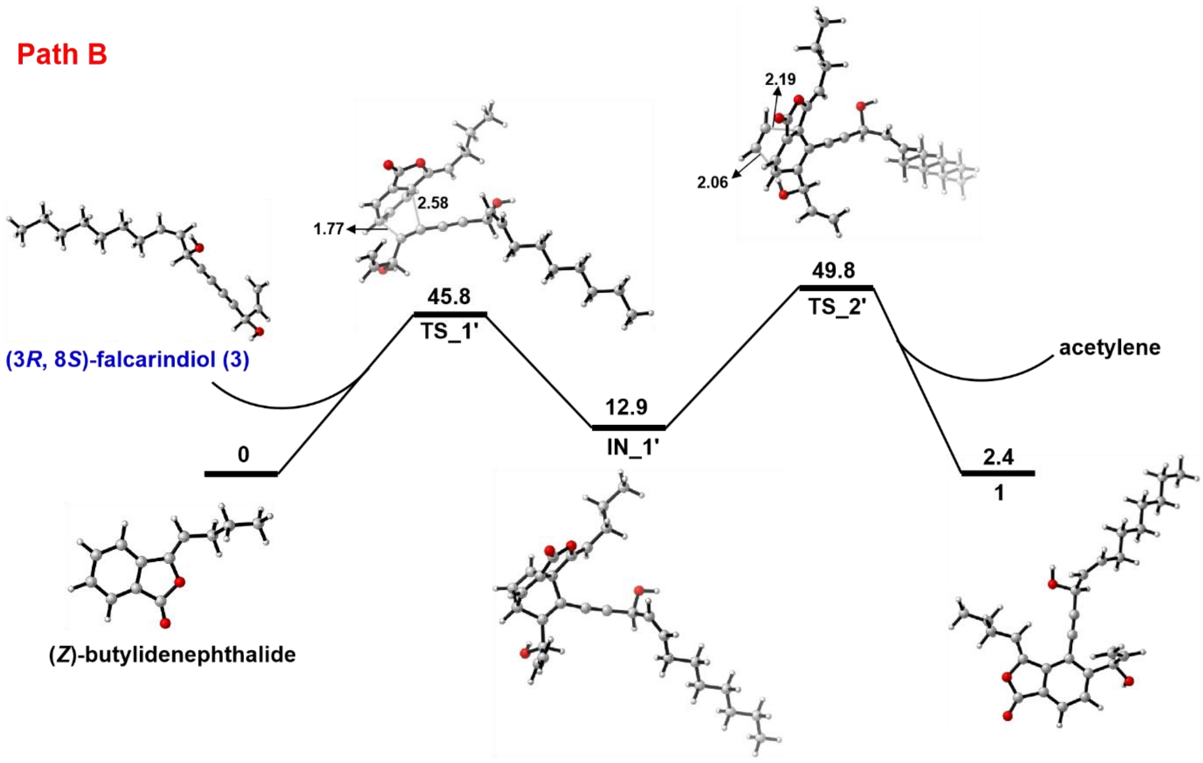

Figure S16. DFT calculations for path B; relative energies from M062X/6–311+G(d,p)//B3LYP/6–31G(d) are shown.

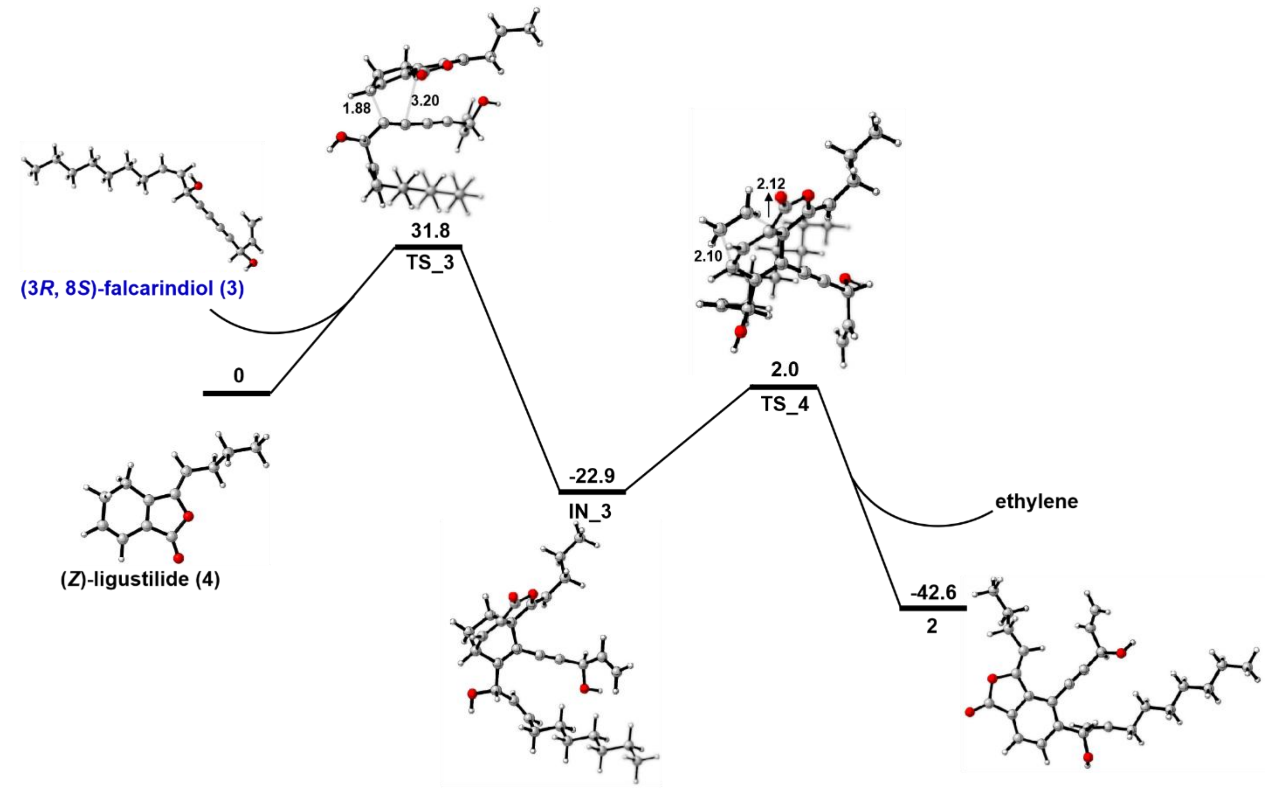

Figure S17. DFT calculations for **2**; relative energies from M062X/6–311+G(d,p)//B3LYP/6–31G(d) are shown.

8.2 FMO analysis

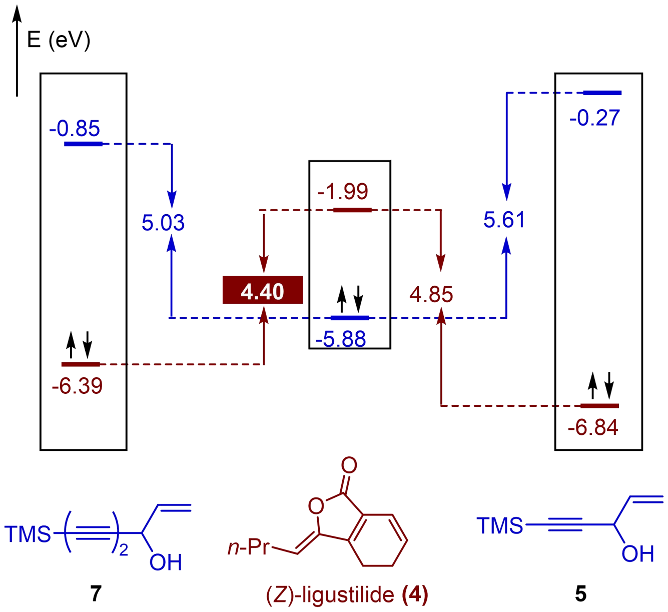

Figure S18. FMO analyses of **4**, **5**, and **7**.

8.2 Cartesian coordinates

Z-ligustilie (4)

C	3.78362500	-0.67536900	0.27174700
C	3.22061400	0.53636900	0.11383800
C	1.77573700	0.61274700	-0.00695900
C	0.98961000	-0.47872800	-0.18675900
C	0.91367200	1.80598200	0.00991000
O	-0.39953200	1.35473200	-0.16118000
C	-0.38547600	-0.02885200	-0.29322100
O	1.17497100	2.97595200	0.13440500
H	4.86010700	-0.77186300	0.39069900
H	3.80396000	1.45171200	0.08770800
C	-2.89787400	-0.16108800	-0.57333200
H	-2.84977700	0.93237300	-0.54473100
H	-3.33762400	-0.43629400	-1.54474100
C	-3.82945400	-0.67407100	0.54559200
H	-3.84473800	-1.77291400	0.53109500
H	-3.41116700	-0.38391700	1.51782000
C	-5.25768900	-0.13678200	0.41110500
H	-5.70784000	-0.44098000	-0.54188600
H	-5.89891400	-0.50958100	1.21770900
H	-5.27364300	0.95898600	0.45103200
C	-1.51496000	-0.73166100	-0.48611700
H	-1.40821300	-1.81190100	-0.56685600
C	1.58480200	-1.84764200	-0.34405100
H	1.73050500	-2.03533800	-1.41954400
H	0.91048800	-2.63231900	0.01652400
C	2.94361100	-1.93277900	0.39281200
H	3.50135700	-2.80235000	0.02743100
H	2.76468400	-2.11983400	1.46455600

M062X/6–311+G(d,p) Total Energy: -616.0377546 Hartree
B3LYP/6-31G(d) Thermal correction to Gibbs Free Energy: 0.194518 Hartree

(3*R*, 8*S*)-falcarindiol (3)

C	-5.48611000	-0.78898700	-0.26862700
C	-4.38937300	-0.28051800	-0.40790500
C	-3.15708400	0.29133100	-0.54934000
C	-6.79541700	-1.45113000	-0.19493500
C	-2.05890000	0.79829200	-0.67206200
C	-0.71780500	1.37870500	-0.78934800
C	-0.19294700	1.78367000	0.57203300
C	0.88909100	1.30376100	1.19838300
O	-0.80528300	2.51910500	-1.65458300
C	1.84089200	0.22710900	0.75045700
C	3.30556800	0.70661000	0.71758200
C	4.29304300	-0.40179500	0.33030200
C	5.75460800	0.06463100	0.30271500
C	6.74227600	-1.04271300	-0.08832800
C	8.20515700	-0.58020900	-0.11180900
C	9.18371100	-1.69203600	-0.50511700
O	-7.54517200	-1.28304200	-1.40484100
C	-7.68238900	-0.96180600	0.92951300
C	-7.32004400	-0.13981900	1.91097200
H	-6.61333600	-2.52938900	-0.02858400
H	-0.05824900	0.62027000	-1.23830000
H	-0.78339800	2.56567700	1.04659800
H	1.12978100	1.74146500	2.16896800
H	0.05846700	2.96121700	-1.60693200
H	1.76627300	-0.61907900	1.45097100
H	1.56273000	-0.17020000	-0.23265700
H	3.39538000	1.54440700	0.01183600
H	3.57614900	1.10951500	1.70438200
H	4.02072800	-0.80235200	-0.65745900
H	4.19170200	-1.23976500	1.03585400
H	6.02736400	0.46193600	1.29170600
H	5.85462500	0.90521600	-0.39998100
H	6.64018300	-1.88507500	0.61216400
H	6.47162600	-1.43812000	-1.07883400
H	8.30734000	0.26197500	-0.81103500
H	8.47629700	-0.18721400	0.87846300
H	10.21851700	-1.33087200	-0.51196900
H	9.12938900	-2.53417400	0.19592000
H	8.96007100	-2.08115400	-1.50620300
H	-6.98479100	-1.58570000	-2.13760400
H	-8.69076400	-1.36899300	0.88080600
H	-8.01927100	0.14283500	2.69272400
H	-6.31883900	0.27849500	1.96764900

M062X/6–311+G(d,p) Total Energy: -812.3869388 Hartree
B3LYP/6-31G(d) Thermal correction to Gibbs Free Energy: 0.313629 Hartree

TS_1

Imaginary frequency: -383.11 cm^{-1}

C	-3.02088300	-2.55970800	-0.37852900
C	-4.66526500	-1.82743500	-1.08426500
C	-5.14118800	-1.03992400	-0.02322000
C	-4.57066800	0.23878300	0.11388400
C	-3.67558400	0.76836500	-0.79201800
C	-2.01181300	-1.79970400	-0.31813200
C	-4.58752600	1.11612800	1.30370200
O	-3.67225200	2.13615700	1.06327000
C	-3.09195500	1.93829100	-0.19179400
O	-5.21727800	1.04795000	2.32952300
C	-0.94892500	-0.97724500	-0.32971700
C	-3.29937000	-4.00586200	-0.01604100
C	0.01237700	-0.21389300	-0.37771800
C	1.28001100	0.51273900	-0.38133800
C	1.79879900	0.66484900	1.03262300
C	2.95004600	0.19170400	1.52754600
O	1.07906800	1.80916200	-0.98217300
C	4.00669800	-0.63285300	0.84258200
C	5.40032200	0.02349400	0.90168100

C	6.50349200	-0.84644100	0.28547200
C	7.89351300	-0.19895600	0.34165800
C	8.99998200	-1.07153900	-0.26558000
C	10.39062500	-0.42536200	-0.20970900
C	11.48994100	-1.30501200	-0.81471200
O	-3.96901000	-4.72368600	-1.05776100
C	-4.13445000	-4.19615500	1.23115300
C	-4.15739900	-3.38588400	2.28867900
H	-5.16266000	-2.77846700	-1.26060200
H	-5.74413800	-1.46010600	0.77395900
H	-2.31564000	-4.46906900	0.15563800
H	2.00194500	-0.05116000	-0.99152700
H	1.13711100	1.24165900	1.67623000
H	3.16971000	0.42730300	2.57045600
H	1.92518000	2.27970700	-0.89250100
H	4.06148500	-1.61365100	1.33989400
H	3.74149000	-0.83834800	-0.20119300
H	5.36389000	0.99405300	0.38694700
H	5.65130300	0.24433900	1.94928600
H	6.24896300	-1.07078900	-0.76110500
H	6.53215800	-1.81568900	0.80509300
H	8.14488800	0.02988300	1.38797800
H	7.86512100	0.76828300	-0.18188400
H	9.02887800	-2.03862900	0.25844900
H	8.74934900	-1.30153300	-1.31204400
H	10.36265800	0.53962300	-0.73571100
H	10.64030500	-0.19411700	0.83566600
H	12.46926200	-0.81578300	-0.76112200
H	11.56678300	-2.26315400	-0.28590100
H	11.28520400	-1.52588600	-1.86966600
H	-3.39411600	-4.69867700	-1.83949900
H	-4.71101900	-5.11996600	1.22307900
H	-4.75036200	-3.62675000	3.16638100
H	-3.58969600	-2.46023900	2.32046300
C	-1.51388000	3.89659800	0.09952200
H	-1.93784300	3.95302300	1.10762100
H	-0.43459600	3.71693300	0.20288300
C	-1.72473300	5.24000100	-0.63129700
H	-1.32116300	5.16602100	-1.65065900
H	-2.80135600	5.42550500	-0.73772300
C	-1.06575300	6.41364000	0.10036900
H	0.01762900	6.26591000	0.19155900
H	-1.23079200	7.35738600	-0.43187900
H	-1.47095800	6.52610900	1.11326700
C	-2.09963000	2.73366200	-0.63863100
H	-1.68975500	2.50396000	-1.61824500
C	-3.45953200	0.21948600	-2.17063500
H	-2.38739500	0.09290000	-2.35608900
H	-3.80162000	0.95623700	-2.91106600
C	-4.24641300	-1.10905300	-2.37317400
H	-3.67266600	-1.78987000	-3.00967600
H	-5.17679400	-0.88989000	-2.91379900

M062X/6–311+G(d,p) Total Energy: -1428.398363 Hartree

B3LYP/6-31G(d) Thermal correction to Gibbs Free Energy: 0.531872 Hartree

IN_1

C	-2.76320100	-2.29532900	-0.46700200
C	-4.26781100	-2.53918300	-0.42959000
C	-4.85157900	-1.85507500	0.79111200
C	-4.50045000	-0.56751000	0.85428600
C	-3.62540300	-0.05399100	-0.25935700
C	-2.40587900	-0.99125300	-0.39228700
C	-4.81340400	0.53135400	1.77968200
O	-4.16099700	1.65565600	1.30593600
C	-3.42558700	1.39098200	0.14088500
O	-5.48781500	0.55450600	2.77831400
C	-1.08649600	-0.48207900	-0.48228500
C	-1.82296700	-3.45069600	-0.69514900
C	0.01475800	0.02149400	-0.56465100
C	1.32666900	0.67209200	-0.63828400
C	1.77603800	1.12659700	0.73344400
C	2.91117800	0.79711000	1.36364500
O	1.19094300	1.80021700	-1.52095800
C	4.01711100	-0.11649800	0.90695600
C	5.37638500	0.60656200	0.81894100
C	6.53308000	-0.32219500	0.42836500
C	7.88445900	0.39844100	0.33140500
C	9.04567900	-0.52485800	-0.05986300
C	10.39559300	0.19797500	-0.16033600
C	11.55072500	-0.72991400	-0.55106600
O	-2.20416000	-4.17787000	-1.87798300
C	-1.79887200	-4.46525500	0.42598600
C	-2.08963700	-4.22378600	1.70389300
H	-4.50299400	-3.60229900	-0.48302500
H	-5.50388200	-2.35819600	1.49817900
H	-0.80410000	-3.05162400	-0.82233200
H	2.05146200	-0.04125200	-1.05754700
H	1.07855000	1.81097500	1.21342900
H	3.07986200	1.25199000	2.34146200
H	2.00384800	2.32221900	-1.41779800
H	4.10750700	-0.94529000	1.62565500
H	3.78452600	-0.57730600	-0.06016200
H	5.29946600	1.42664100	0.09104200
H	5.59761800	1.07938200	1.78676400
H	6.30640100	-0.80079300	-0.53595500
H	6.60705400	-1.13824300	1.16237200
H	8.11022100	0.87711300	1.29589600
H	7.80746400	1.21527000	-0.40161100
H	9.12573700	-1.34062300	0.67417200
H	8.81975400	-1.00559500	-1.02349900
H	10.31570000	1.01244100	-0.89441300
H	10.62105800	0.67830000	0.80251300
H	12.49900200	-0.18396800	-0.61423200
H	11.67834000	-1.53520900	0.18300900
H	11.37046100	-1.19837800	-1.52660500
H	-2.04971000	-3.59111900	-2.63529200
H	-1.47509800	-5.45516400	0.10735100
H	-2.00802100	-5.00303500	2.45641400
H	-2.40821100	-3.24317400	2.04849500
C	-2.60011600	3.77741600	0.01191400
H	-3.11522000	3.90417800	0.97008100

H	-1.53796000	4.00814300	0.18577400
C	-3.15662400	4.78444400	-1.01444700
H	-2.65780800	4.62954800	-1.98138100
H	-4.22119200	4.57311600	-1.18017600
C	-2.97889800	6.23989500	-0.57077500
H	-1.91869400	6.48423400	-0.42892400
H	-3.38501700	6.93584600	-1.31379500
H	-3.49217800	6.42955200	0.37988900
C	-2.71449800	2.35157700	-0.44950800
H	-2.15815300	2.07178300	-1.34067300
C	-4.45031500	-0.27298400	-1.58927800
H	-3.84227000	0.04244300	-2.44260400
H	-5.33482300	0.37026300	-1.56030000
C	-4.84385300	-1.76970500	-1.68153800
H	-4.43749800	-2.23572400	-2.58414400
H	-5.93148500	-1.89029400	-1.71108100

M062X/6–311+G(d,p) Total Energy: -1428.494021 Hartree

B3LYP/6-31G(d) Thermal correction to Gibbs Free Energy: 0.53737 Hartree

TS_2

Imaginary frequency: -559.88 cm^{-1}

C	-2.51986700	-2.32047000	-0.23716600
C	-3.89982700	-2.77178000	-0.29323300
C	-4.85298800	-2.09334200	0.54302200
C	-4.63274000	-0.75845700	0.67802000
C	-3.51518300	-0.15618700	0.00294000
C	-2.30457900	-0.96555000	-0.09918100
C	-5.47289700	0.30902500	1.24084500
O	-4.84980000	1.51008500	0.93508900
C	-3.63710300	1.29486900	0.26571100
O	-6.51292300	0.26259300	1.84869700
C	-1.01480900	-0.36604100	-0.17093500
C	-1.40906300	-3.31092200	-0.52008300
C	0.06673200	0.17877900	-0.24417400
C	1.34890600	0.88603700	-0.32871100
C	1.85846500	1.25805100	1.04653000
C	3.02185800	0.89628400	1.60305600
O	1.12238000	2.06865600	-1.11617500
C	4.10218200	0.00940100	1.04444900
C	5.45757800	0.73376500	0.91605400
C	6.58660000	-0.18573200	0.43279400
C	7.93509700	0.53050400	0.28014400
C	9.06578500	-0.39142600	-0.19540600
C	10.41368000	0.32372500	-0.35801900
C	11.53745300	-0.60602500	-0.82821200
O	-1.58918200	-3.92134300	-1.81428700
C	-1.30116600	-4.44955200	0.46664200
C	-1.80114400	-4.47556800	1.70161600
H	-4.04416800	-3.83274400	-0.47618800
H	-5.74778600	-2.58396600	0.91347600
H	-0.45269600	-2.76683500	-0.51048400
H	2.07528000	0.23629600	-0.83865400
H	1.18100800	1.90728900	1.59884500
H	3.23502400	1.29269400	2.59741500
H	1.90975100	2.62630800	-1.00149000
H	4.22871000	-0.85209000	1.71776800
H	3.81720700	-0.40640800	0.07113000
H	5.34814100	1.58043800	0.22379800
H	5.73010400	1.16873600	1.88848700
H	6.30298500	-0.63409200	-0.53100600
H	6.69673500	-1.02385000	1.13668600
H	8.21665000	0.98420900	1.24200300
H	7.82442700	1.36490000	-0.42824000
H	9.18059200	-1.22266700	0.51621200
H	8.78175300	-0.85050500	-1.15421400
H	10.30004700	1.15164600	-1.07226500
H	10.69642200	0.78487500	0.59904700
H	12.48542600	-0.06634700	-0.93467800
H	11.69830200	-1.42473800	-0.11592300
H	11.29936800	-1.05605000	-1.80002800
H	-1.38828500	-3.24382100	-2.47935100
H	-0.71499600	-5.28741400	0.09126900
H	-1.63346600	-5.32557400	2.35712600
H	-2.38641800	-3.65417900	2.10620300
C	-3.12879000	3.76734700	0.26878700
H	-4.06025700	3.85290100	0.83770000
H	-2.32535300	4.15782600	0.91285100
C	-3.20521700	4.64529100	-0.99701500
H	-2.27681400	4.53096900	-1.57324900
H	-4.01458600	4.27505400	-1.63983100
C	-3.43488200	6.12513800	-0.67407200
H	-2.62025600	6.52798900	-0.05929400
H	-3.49294600	6.72784200	-1.58764400
H	-4.37009900	6.26859400	-0.11923400
C	-2.84256400	2.32664400	-0.04396400
H	-1.91728100	2.10347500	-0.56691500
C	-4.21137800	-0.51815300	-1.99397700
H	-3.34146300	-0.07683800	-2.47018700
H	-5.06678100	0.14659400	-1.92383700
C	-4.43048400	-1.90869600	-2.09851600
H	-3.74105400	-2.51305500	-2.67943900
H	-5.45496200	-2.26912000	-2.12213300

M062X/6–311+G(d,p) Total Energy: -1428.444438 Hartree

B3LYP/6-31G(d) Thermal correction to Gibbs Free Energy: 0.532949 Hartree

1

C	-2.81185400	-2.43350100	-0.46199500
C	-4.08701700	-2.99838900	-0.63380200
C	-5.23935000	-2.21877200	-0.59040400
C	-5.08611200	-0.85362300	-0.36841500
C	-3.83564000	-0.25612700	-0.18967000
C	-2.66167600	-1.04348300	-0.23500700
C	-6.11870700	0.19613300	-0.28531100
O	-5.45284000	1.38955400	-0.05839700
C	-4.07103100	1.17556500	0.01028300
O	-7.31865400	0.13211800	-0.38205600
C	-1.37485400	-0.45159100	-0.06472400
C	-1.59517100	-3.34634500	-0.52647400
C	-0.30010600	0.09122900	0.08041500

C	0.96683600	0.80393700	0.27591300
C	1.59396600	0.44780600	1.60586900
C	2.80281500	-0.09155800	1.80996300
O	0.66496400	2.20936900	0.22072100
C	3.83465400	-0.51401000	0.79890900
C	5.18975600	0.19142000	1.00737700
C	6.27417800	-0.28772500	0.03374800
C	7.62546900	0.41107900	0.23464400
C	8.71189100	-0.06736700	-0.73756300
C	10.06376700	0.63065000	-0.53790100
C	11.14288800	0.14699500	-1.51247500
O	-1.76644800	-4.39234400	-1.49012700
C	-1.26892400	-4.04228800	0.77661000
C	-1.79261000	-3.78261900	1.97327000
H	-4.14702800	-4.06654700	-0.81367100
H	-6.22736100	-2.64844300	-0.72403300
H	-0.72107600	-2.73543900	-0.80307800
H	1.64728600	0.53938800	-0.54681500
H	0.96573500	0.69010800	2.46144300
H	3.09960400	-0.25177400	2.84804000
H	1.45599400	2.67260300	0.54236700
H	3.98877400	-1.60021500	0.89042000
H	3.48670200	-0.34302200	-0.22648900
H	5.05102900	1.27653500	0.90103900
H	5.52739900	0.02750800	2.04094900
H	5.93221100	-0.12735600	-0.99951800
H	6.40591800	-1.37424200	0.14472900
H	7.96673200	0.25072800	1.26815200
H	7.49231400	1.49756200	0.12424900
H	8.84563500	-1.15393700	-0.62742700
H	8.37099000	0.09261500	-1.77143900
H	9.93019500	1.71625900	-0.64855000
H	10.40454900	0.47040700	0.49495500
H	12.09473900	0.66365300	-1.34437000
H	11.32388500	-0.92931400	-1.40127400
H	10.84604100	0.32589700	-2.55336700
H	-1.87576100	-3.97223100	-2.35859900
H	-0.51131300	-4.81692900	0.66437800
H	-1.47056000	-4.32970800	2.85490900
H	-2.55178900	-3.02034600	2.12363400
C	-3.64077000	3.63443600	0.39723400
H	-4.72806100	3.73533400	0.31880000
H	-3.36214400	3.96492400	1.41050100
C	-2.94386100	4.55956300	-0.62211600
H	-1.85721700	4.41386500	-0.55447800
H	-3.23294200	4.25448600	-1.63621500
C	-3.28767000	6.03659800	-0.40656400
H	-2.97984300	6.37433500	0.59096700
H	-2.78533700	6.67294800	-1.14408400
H	-4.36697600	6.21000300	-0.49625600
C	-3.23439100	2.20076000	0.23061400
H	-2.17336400	1.97812700	0.27442600

M062X/6–311+G(d,p) Total Energy: -1349.930577 Hartree
B3LYP/6-31G(d) Thermal correction to Gibbs Free Energy: 0.479828 Hartree

ethylene

C	0.66545800	0.00000700	0.00000900
H	1.23983300	-0.92361200	0.00005600
H	1.23962200	0.92343800	-0.00037800
C	-0.66581500	-0.00010500	0.00020000
H	-1.23914800	-0.92384000	-0.00068600
H	-1.23816200	0.92460600	-0.00025100

M062X/6–311+G(d,p) Total Energy: -78.5634731 Hartree
B3LYP/6-31G(d) Thermal correction to Gibbs Free Energy: 0.029051 Hartree

***Z*-butylidenephthalide**

C	-3.79780000	-0.79760500	-0.24370100
C	-3.22557900	0.47359200	-0.22244400
C	-1.84636200	0.55722000	-0.04587800
C	-1.04336800	-0.57764600	0.10612900
C	-0.97701000	1.74753400	0.01726800
O	0.32256500	1.28213600	0.20665000
C	0.33506100	-0.11066800	0.26540700
O	-1.23088300	2.92160200	-0.06572300
C	-1.61663000	-1.85183900	0.08542100
C	-2.99774800	-1.94380000	-0.09090700
H	-4.86974700	-0.90840200	-0.37875200
H	-3.82213000	1.37334000	-0.33804400
H	-1.01373000	-2.74767600	0.20219300
H	-3.46671800	-2.92382200	-0.11044000
C	2.84311200	-0.19376000	0.57973000
H	2.76932100	0.89852000	0.59676200
H	3.27240900	-0.49916100	1.54656600
C	3.80522100	-0.63554300	-0.54298400
H	3.85079200	-1.73328200	-0.57206800
H	3.39390300	-0.31837100	-1.50980200
C	5.21582100	-0.06543800	-0.36444000
H	5.65982600	-0.39438700	0.58323100
H	5.87952300	-0.38850300	-1.17431700
H	5.20171900	1.03108600	-0.36143300
C	1.47475900	-0.79232200	0.44262300
H	1.39450300	-1.87759700	0.47134900

M062X/6–311+G(d,p) Total Energy: -614.8614227 Hartree
B3LYP/6-31G(d) Thermal correction to Gibbs Free Energy: 0.173008 Hartree

TS_1'

Imaginary frequency: -264.52 cm^{-1}

C	-3.16769600	-2.33883900	-0.55958500
C	-4.87268500	-1.86487200	-0.53857600
C	-5.01542300	-1.16200600	0.74147800
C	-4.42052100	0.05906100	0.77333200
C	-3.85494700	0.61006000	-0.42030800
C	-2.27489400	-1.42019800	-0.54353900
C	-4.06691100	0.94994800	1.89517500
O	-3.27088600	1.95993000	1.36530100
C	-3.12520900	1.79544800	-0.01777800
O	-4.33946300	0.90001100	3.06746700

C	-1.10575300	-0.75096000	-0.58900400
C	-3.06210700	-3.85108400	-0.66320200
C	-0.09677100	-0.05509500	-0.63861200
C	1.20437400	0.61249900	-0.65792000
C	1.67892900	0.88201400	0.75389900
C	2.80244100	0.43205300	1.32791400
O	1.07597000	1.84698300	-1.39082600
C	3.86395600	-0.46884600	0.75563100
C	5.26675400	0.16898800	0.80158800
C	6.37267300	-0.76581300	0.29518600
C	7.77069300	-0.13476800	0.33934400
C	8.88007600	-1.06713400	-0.16486700
C	10.27794100	-0.43574300	-0.12152100
C	11.38053500	-1.37372100	-0.62423300
C	-4.39865600	0.22067000	-1.68633500
C	-4.99409100	-1.00152500	-1.72980800
O	-3.85345100	-4.35581300	-1.74327400
C	-3.51827200	-4.58929200	0.57629100
C	-3.41658800	-4.14329800	1.82812900
H	-5.40465300	-2.81024300	-0.61193300
H	-5.40727900	-1.65865200	1.62240700
H	-2.00406800	-4.09304300	-0.84533600
H	1.92203700	-0.03875000	-1.18026400
H	1.01033300	1.52651000	1.32158100
H	2.99269800	0.75521300	2.35297800
H	1.92443200	2.30951500	-1.28559100
H	3.88488800	-1.40099100	1.34110400
H	3.62863200	-0.76369100	-0.27381700
H	5.26216400	1.09251600	0.20548000
H	5.49069600	0.47472200	1.83385900
H	6.14518400	-1.07385200	-0.73621900
H	6.37031900	-1.68809400	0.89487500
H	7.99686700	0.17369100	1.37086700
H	7.77147500	0.78783600	-0.26003700
H	8.88073300	-1.98960600	0.43493900
H	8.65440800	-1.37673100	-1.19638300
H	10.27788700	0.48484000	-0.72249200
H	10.50300400	-0.12551000	0.90887800
H	12.36484400	-0.89347600	-0.58188700
H	11.42955400	-2.28772700	-0.01943500
H	11.20135300	-1.67508500	-1.66380500
H	-4.32120300	0.87096700	-2.55251800
H	-5.41862700	-1.40940200	-2.64200000
H	-3.51438200	-3.95375700	-2.55925800
H	-3.91829700	-5.58050200	0.36770000
H	-3.72806900	-4.75605700	2.66930500
H	-3.01328700	-3.16111100	2.05965000
C	-1.59855900	3.80816400	-0.21262700
H	-1.70057700	3.86104200	0.87666300
H	-0.53279800	3.66715600	-0.43833300
C	-2.07455300	5.13308900	-0.84480100
H	-1.99608000	5.06223300	-1.93865700
H	-3.13944900	5.27679500	-0.62024600
C	-1.27223800	6.34039300	-0.34909600
H	-0.20700900	6.23354700	-0.58905100
H	-1.62678900	7.26985600	-0.80911200
H	-1.35739600	6.45269700	0.73860100
C	-2.34013200	2.61605500	-0.73674800
H	-2.25309000	2.39678100	-1.79828200

M062X/6–311+G(d,p) Total Energy: -1427.198535 Hartree

B3LYP/6-31G(d) Thermal correction to Gibbs Free Energy: 0.509746 Hartree

IN_1'

C	-2.75295000	-2.30352800	-0.57469400
C	-4.27676600	-2.55755100	-0.56207800
C	-4.79521000	-1.94175100	0.74533100
C	-4.47664400	-0.65060400	0.83132000
C	-3.69845300	-0.08864400	-0.34928500
C	-2.42346300	-1.00047400	-0.47394200
C	-4.68836000	0.41726100	1.81675200
O	-4.10093900	1.56470200	1.30912500
C	-3.51750200	1.35344100	0.05285100
O	-5.24549000	0.40373400	2.88532400
C	-1.13163200	-0.43076700	-0.47843400
C	-1.81054900	-3.45697500	-0.78659300
C	-0.06332600	0.14620100	-0.47563500
C	1.20954100	0.87229400	-0.43897100
C	1.70682200	1.00887400	0.98392900
C	2.86631400	0.56154900	1.48325400
O	0.97686300	2.16958900	-1.01578800
C	3.96008200	-0.20915300	0.79343300
C	5.33179200	0.48535400	0.90469400
C	6.47138500	-0.32411500	0.27269300
C	7.84126200	0.35699200	0.39170000
C	8.98325600	-0.45011500	-0.23974600
C	10.35439300	0.22748800	-0.11540200
C	11.48896400	-0.58396200	-0.74985100
C	-4.53284200	-0.40163800	-1.60483800
C	-4.84260300	-1.69310500	-1.70625400
O	-2.17809900	-4.18399400	-1.97100000
C	-1.79935800	-4.47196800	0.33503800
C	-2.06810400	-4.22043200	1.61582200
H	-4.50796300	-3.61624400	-0.66699800
H	-5.34066700	-2.51722900	1.48498200
H	-0.79153800	-3.05295700	-0.90156100
H	1.94469700	0.32355900	-1.04632800
H	1.01913600	1.54960800	1.63200500
H	3.06409300	0.77768300	2.53477000
H	1.77208100	2.69491400	-0.82687900
H	4.03442800	-1.20445900	1.25844700
H	3.72273000	-0.38560900	-0.26226700
H	5.27453500	1.47520900	0.43034000
H	5.55792600	0.66893300	1.96509700
H	6.24559400	-0.50376200	-0.78897300
H	6.51605100	-1.31644000	0.74552400
H	8.06623600	0.53486300	1.45382600
H	7.79529200	1.35035600	-0.07894800
H	9.02737500	-1.44506500	0.22800700
H	8.76097200	-0.62503000	-1.30312700
H	10.31011100	1.22199200	-0.58196100

H	10.57724800	0.40039200	0.94715700
H	12.45365200	-0.07472400	-0.64337100
H	11.58013400	-1.57167800	-0.28111800
H	11.31225800	-0.74220300	-1.82100700
H	-4.81958400	0.39113900	-2.28633700
H	-5.42727200	-2.14394700	-2.50073300
H	-2.10556900	-3.56776700	-2.71757700
H	-1.50611800	-5.47040700	0.01362700
H	-1.99901800	-5.00037800	2.36886100
H	-2.35462500	-3.23063700	1.96217800
C	-2.71376000	3.74834200	-0.12672600
H	-3.13293100	3.86492500	0.87865800
H	-1.63278800	3.94143800	-0.05053100
C	-3.33644000	4.79063100	-1.07581000
H	-2.93060900	4.64762500	-2.08719200
H	-4.41673100	4.60819900	-1.14907900
C	-3.08318900	6.23088200	-0.61941400
H	-2.00867400	6.44627900	-0.56678700
H	-3.53522900	6.95336200	-1.30863000
H	-3.50579800	6.41042300	0.37675500
C	-2.89933000	2.33578600	-0.60113800
H	-2.45809300	2.07353800	-1.56015800

M062X/6–311+G(d,p) Total Energy: -1427.253766 Hartree

B3LYP/6-31G(d) Thermal correction to Gibbs Free Energy: 0.512532 Hartree

TS_2'

Imaginary frequency: -577.29 cm^{-1}

C	-2.57954400	-2.34052500	-0.25496100
C	-3.95164500	-2.80322200	-0.37566700
C	-4.94561000	-2.15447100	0.43629000
C	-4.74707500	-0.81935200	0.59895400
C	-3.61015700	-0.19786500	-0.01929900
C	-2.38543500	-0.98643400	-0.08337900
C	-5.62710700	0.23283000	1.12953200
O	-5.01195700	1.44504500	0.84998800
C	-3.77238800	1.24987700	0.22760000
O	-6.68784700	0.16757100	1.69738500
C	-1.10374500	-0.36925400	-0.09062100
C	-1.45105000	-3.31977500	-0.52665000
C	-0.02891300	0.19328700	-0.09955300
C	1.24493300	0.92003700	-0.10534100
C	1.80616900	1.04237900	1.29455200
C	2.99664500	0.60847800	1.72911900
O	0.98076800	2.22156700	-0.65803200
C	4.06916900	-0.12948600	0.97317800
C	5.42162400	0.61054900	0.99385900
C	6.54212200	-0.16192000	0.28587800
C	7.89192000	0.56757200	0.30562600
C	9.01297500	-0.20024400	-0.40717200
C	10.36329100	0.52828900	-0.38720700
C	11.47716200	-0.24396600	-1.10210100
C	-4.23731800	-0.63766300	-2.07190300
C	-4.39511800	-1.87667800	-2.16461000
O	-1.70323300	-4.02759900	-1.74955300
C	-1.32535200	-4.35503700	0.56138000
C	-0.30480300	-4.42060700	1.41526400
H	-4.08089800	-3.85871500	-0.59937300
H	-5.84735700	-2.66202100	0.76306500
H	-0.51007600	-2.75764900	-0.59212900
H	1.95362900	0.38101400	-0.75120000
H	1.14273500	1.56142100	1.98438800
H	3.24310700	0.81335600	2.77256300
H	1.77215100	2.75747500	-0.48369200
H	4.20630800	-1.11919400	1.43561000
H	3.77151100	-0.31891200	-0.06470200
H	5.29951900	1.59690300	0.52448800
H	5.71207500	0.80378300	2.03667900
H	6.24840200	-0.35483500	-0.75662300
H	6.65494100	-1.14967900	0.75695700
H	8.18707300	0.75651300	1.34845500
H	7.77662000	1.55731700	-0.16084400
H	9.12867900	-1.19058700	0.05819700
H	8.71843200	-0.38859300	-1.45047200
H	10.24722100	1.51784100	-0.85201300
H	10.65798700	0.71546600	0.65526000
H	12.42640300	0.30311600	-1.07226000
H	11.64248400	-1.22350200	-0.63650300
H	11.22576000	-0.41768400	-2.15576100
H	-4.24111300	0.35427700	-2.48024700
H	-4.67239600	-2.71153500	-2.78175400
H	-1.80210300	-3.36042100	-2.44874300
H	-2.14947100	-5.06438500	0.62891200
H	-0.26567100	-5.16928500	2.20157200
H	0.52818700	-3.72280000	1.36481500
C	-3.30301200	3.73055500	0.22836700
H	-4.25655800	3.80672300	0.76085300
H	-2.52919500	4.13663700	0.89813500
C	-3.34348200	4.59700100	-1.04717000
H	-2.39245800	4.49079300	-1.58679800
H	-4.12353500	4.21082400	-1.71658500
C	-3.60502000	6.07612900	-0.74604400
H	-2.81964700	6.49476800	-0.10446100
H	-3.63607100	6.67020700	-1.66652200
H	-4.56249900	6.21144600	-0.22849100
C	-2.98314800	2.29254300	-0.06097100
H	-2.03256600	2.07908600	-0.54078400

M062X/6–311+G(d,p) Total Energy: -1427.188695 Hartree

B3LYP/6-31G(d) Thermal correction to Gibbs Free Energy: 0.506382 Hartree

acetylene

C	-0.60257900	-0.00030100	0.00000700
C	0.60257900	-0.00031200	-0.00000700
H	-1.66906200	0.00182900	-0.00001600
H	1.66905900	0.00185300	0.00001500

M062X/6–311+G(d,p) Total Energy: -77.3161383 Hartree

B3LYP/6-31G(d) Thermal correction to Gibbs Free Energy: 0.009013 Hartree

TS_3

Imaginary frequency: -368.93 cm^{-1}

C	-0.12623600	-2.58157500	0.60077500
C	-0.78493800	-2.00208500	-0.31352700
C	1.15446800	-3.37235300	0.66473700
H	1.86009500	-2.79988600	1.28561100
O	0.86304400	-4.62640200	1.31122800
C	-2.09860600	-0.00871700	1.81208900
C	-0.84737800	-0.00964800	2.38960600
C	-0.16453800	-1.18529000	2.75179800
C	-0.75547600	-2.40598900	2.36759000
C	-2.28728700	-2.47298100	2.31403200
C	-3.00171200	-1.20151800	1.76742000
H	0.85727700	-1.12993700	3.11536500
H	-0.30943400	-3.31606000	2.76290700
H	-2.59177100	-3.35260600	1.73983500
H	-3.36759000	-1.34617600	0.74421500
C	-2.35337300	1.32281600	1.33091800
C	-0.31335000	1.36709800	2.32234900
O	-1.27129100	2.13881600	1.67174100
O	0.72853800	1.83123000	2.71236400
C	-3.38449600	1.78899000	0.59954800
H	-4.17504700	1.08191600	0.36361000
C	-3.52178200	3.18028400	0.06666100
H	-2.62976500	3.76609000	0.31196400
H	-3.58005500	3.11481700	-1.02960500
C	-4.78280900	3.90166200	0.58681300
H	-4.73206700	3.96911600	1.68123800
H	-5.66924400	3.29474800	0.35563600
C	-4.94599400	5.30194000	-0.01198300
H	-5.84604300	5.79607500	0.37111800
H	-4.08664500	5.93856000	0.23064500
H	-5.02753300	5.25864500	-1.10519400
H	-2.61558500	-2.64529700	3.34740800
H	-3.89633600	-0.99999900	2.37346200
C	-1.58271600	-1.36218200	-1.18020800
C	-2.35266400	-0.78133700	-1.94359500
C	-3.16304000	-0.23579700	-3.02902200
H	-2.46700700	0.23383900	-3.74887500
C	-3.89156500	-1.35967200	-3.75308500
H	-3.24661500	-2.02946700	-4.31940400
C	-5.20903700	-1.54814500	-3.70953400
H	-5.68168600	-2.36737200	-4.24318700
H	-5.85432200	-0.89732800	-3.12649700
O	-4.05603500	0.76041400	-2.51798000
H	-4.54582100	1.10516900	-3.28399700
H	1.69168200	-5.13215100	1.31333400
C	1.73385400	-3.62232400	-0.70326900
H	1.08288100	-4.20611300	-1.35170500
C	2.93428000	-3.23674000	-1.15424700
H	3.19473000	-3.52971900	-2.17304200
C	3.97798200	-2.39995700	-0.46318400
H	4.93660400	-2.94060900	-0.48196100
H	3.73212300	-2.24383800	0.59360900
C	4.17507100	-1.02903000	-1.14270400
H	4.40937300	-1.18283500	-2.20613000
H	3.22511100	-0.47887300	-1.11474400
C	5.28010800	-0.18819500	-0.49128900
H	6.22707100	-0.74816300	-0.51787500
H	5.04348300	-0.03945800	0.57247300
C	5.47862400	1.17827600	-1.15987000
H	5.71456500	1.03095800	-2.22452600
H	4.53088100	1.73540700	-1.13225100
C	6.58115000	2.02306500	-0.50866800
H	6.34411100	2.17123600	0.55529100
H	7.52998800	1.46602300	-0.53370600
C	6.78056000	3.39002100	-1.17589100
H	7.01667100	3.24278500	-2.23962700
H	5.83303200	3.94646900	-1.14919100
C	7.88275000	4.22779000	-0.51962900
H	8.00067500	5.19660700	-1.01852400
H	7.65668700	4.42232000	0.53601300
H	8.85037700	3.71219400	-0.56030900

M062X/6-311+G(d,p) Total Energy: -1428.3957252 Hartree

B3LYP/6-31G(d) Thermal correction to Gibbs Free Energy: 0.529822 Hartree

IN_3

C	-1.09189600	-2.43281500	-0.05321300
C	-1.42307200	-1.13823200	-0.27441800
C	0.06679800	-3.22671200	-0.62025300
H	0.82507200	-3.30690800	0.17624100
O	-0.45879400	-4.54723900	-0.89123900
C	-2.69483000	-0.71885100	0.50878500
C	-2.49867400	-1.09152400	1.95395400
C	-2.15305700	-2.35863400	2.19359100
C	-2.03024200	-3.15991800	0.91406300
C	-3.44399200	-3.12474600	0.21855300
C	-3.84398200	-1.65105800	-0.03874300
H	-2.00275600	-2.78690900	3.17997900
H	-1.71130900	-4.18779200	1.07842200
H	-3.37438400	-3.69138400	-0.71369100
H	-3.98944800	-1.44975600	-1.10432800
C	-3.06843600	0.74385700	0.61614700
C	-2.75567100	0.07552000	2.80725600
O	-3.07929100	1.12687100	1.96343200
O	-2.71599700	0.20681800	4.00394600
C	-3.40523300	1.61257500	-0.33728600
H	-3.37939500	1.24417000	-1.36033600
C	-3.82471500	3.04026200	-0.12295800
H	-3.73924300	3.29673100	0.93818400
H	-3.13489200	3.70665900	-0.66465200
C	-5.25880400	3.32325900	-0.61187000
H	-5.95565900	2.67746600	-0.06195200
H	-5.34562400	3.03738000	-1.66973400
C	-5.66410500	4.79029800	-0.44049000
H	-6.68731000	4.96498100	-0.79196600
H	-5.61665200	5.09331100	0.61247400
H	-4.99902200	5.45477600	-1.00618200
H	-4.17585600	-3.62567800	0.86047300
H	-4.77516700	-1.39397500	0.47499100

C	-0.75776800	-0.19735300	-1.10070700
C	-0.22785500	0.66501900	-1.76951600
C	0.42963400	1.70823000	-2.56238300
H	-0.30399500	2.08268900	-3.30047700
C	0.81928600	2.87919400	-1.67175800
H	-0.02091300	3.42554000	-1.24580900
C	2.07389900	3.23862600	-1.40892000
H	2.29639900	4.08869600	-0.77085900
H	2.91890100	2.68398500	-1.80657300
O	1.53926800	1.12864200	-3.24814500
H	1.93341200	1.84018200	-3.77963200
H	0.26042100	-5.04211400	-1.31496200
C	0.69018000	-2.68400900	-1.87549700
H	-0.00121800	-2.61125400	-2.71337900
C	1.97342500	-2.35504800	-2.06849900
H	2.24410200	-2.00135600	-3.06390800
C	3.09845600	-2.31667000	-1.07010800
H	3.94813500	-2.89385200	-1.46632900
H	2.81527700	-2.79358600	-0.12399400
C	3.57090500	-0.87433000	-0.79184400
H	3.81428400	-0.38539100	-1.74522300
H	2.73447300	-0.30182100	-0.37106800
C	4.77886900	-0.80742800	0.15045600
H	5.60735900	-1.39456400	-0.27392000
H	4.52390900	-1.28990500	1.10593700
C	5.25910700	0.62453100	0.42020100
H	5.52508400	1.10235800	-0.53487100
H	4.42702900	1.21387000	0.83265600
C	6.45776300	0.70148700	1.37451600
H	6.19199900	0.22639000	2.33068600
H	7.28942100	0.10962900	0.96335700
C	6.94030600	2.13335800	1.63993400
H	7.20677800	2.60824900	0.68472300
H	6.10958600	2.72432600	2.05125600
C	8.13666300	2.20165200	2.59465000
H	8.45681100	3.23627700	2.76325900
H	7.88980900	1.76632000	3.57086000
H	8.99533900	1.64827700	2.19453500

M062X/6-311+G(d,p) Total Energy: -1428.4907642 Hartree

B3LYP/6-31G(d) Thermal correction to Gibbs Free Energy: 0.53766 Hartree

TS_4

Imaginary frequency: -561.66 cm^{-1}

C	-1.88290900	-2.10589700	0.00138700
C	-1.63866800	-0.80917800	0.40194000
C	-0.97314600	-3.26585500	0.34763600
H	-0.23980600	-2.91478700	1.08170800
O	-1.80685500	-4.28277200	0.92984300
C	-2.55689500	0.18497300	-0.14824900
C	-3.92200900	-0.23787600	-0.31546300
C	-4.17816200	-1.51643200	-0.69633800
C	-3.00732500	-2.32744200	-0.88735700
C	-2.23811800	-1.32905700	-2.56620400
C	-1.98105200	0.00422000	-2.18214900
H	-5.17058400	-1.86691700	-0.96177400
H	-3.15688900	-3.34917400	-1.22470000
H	-1.39673300	-2.00184200	-2.70098700
H	-0.95405100	0.31506000	-2.01742800
C	-2.63569300	1.63092000	0.16077400
C	-4.79651600	0.93908700	-0.21736700
O	-3.97273700	2.02834300	0.03725700
O	-5.99071600	1.05290700	-0.32870500
C	-1.68804600	2.52318200	0.46904600
H	-0.66933900	2.15159300	0.53219600
C	-1.91543800	3.98658000	0.71677500
H	-2.98745800	4.20785800	0.69517100
H	-1.55950000	4.23821700	1.72799200
C	-1.17715600	4.88515500	-0.29616600
H	-1.53918400	4.65481100	-1.30654900
H	-0.10651700	4.63733500	-0.28914900
C	-1.36288800	6.37735900	-0.00520200
H	-0.83286500	6.99491300	-0.73915200
H	-2.42269100	6.65771800	-0.03607500
H	-0.98027200	6.63842200	0.98934600
H	-3.08178600	-1.52906800	-3.22026500
H	-2.63158400	0.79217900	-2.54949100
C	-0.53962800	-0.44275100	1.22950800
C	0.37897300	-0.11939500	1.95416700
C	1.43660000	0.25171700	2.90006500
H	1.04256300	1.06508000	3.53590900
C	1.76663500	-0.92283300	3.80833600
H	0.96631400	-1.20991300	4.48864900
C	2.92931700	-1.57199800	3.80668800
H	3.11499000	-2.39909100	4.48546200
H	3.72826700	-1.30768600	3.11985800
O	2.56244500	0.73212900	2.16442400
H	3.23266100	0.99000900	2.81930100
H	-1.24123400	-5.06010700	1.06730500
C	-0.27073300	-3.83123900	-0.86935100
H	-0.92607100	-4.41277600	-1.51863100
C	1.02381000	-3.72690700	-1.19990900
H	1.34048900	-4.23652700	-2.11231300
C	2.11722500	-2.96286800	-0.50455400
H	2.93016000	-3.66049300	-0.24977500
H	1.76745800	-2.52544300	0.43628500
C	2.69700300	-1.84560700	-1.39731300
H	3.02789200	-2.27948600	-2.35258500
H	1.89422500	-1.13707200	-1.64340100
C	3.86014000	-1.09200000	-0.74098200
H	4.65612300	-1.80741200	-0.48272200
H	3.51536400	-0.64978200	0.20297500
C	4.44265000	0.01330900	-1.63146500
H	4.80557700	-0.42521100	-2.57336200
H	3.64030200	0.71224400	-1.91024400
C	5.58037800	0.79646800	-0.96401100
H	5.20974100	1.23998300	-0.02790500
H	6.38241600	0.10028000	-0.67501500
C	6.16806500	1.90225100	-1.85004500
H	6.53770100	1.45848200	-2.78553100
H	5.36692800	2.59697500	-2.13990000

C	7.30003600	2.68275700	-1.17402700
H	7.69784000	3.46396100	-1.83195100
H	6.95198300	3.16840400	-0.25379900
H	8.13178700	2.02054100	-0.90303700

M062X/6-311+G(d,p) Total Energy: -1428.447746 Hartree

B3LYP/6-31G(d) Thermal correction to Gibbs Free Energy: 0.534315 Hartree

2

C	1.24523100	-2.45205800	-0.14955900
C	1.70552500	-1.10956700	-0.13074000
C	-0.23653400	-2.74633800	0.07955700
H	-0.80884900	-2.07206900	-0.56841700
O	-0.57687100	-4.07377600	-0.34858800
C	3.08007200	-0.88368000	-0.35593100
C	3.94306200	-1.96271200	-0.56738800
C	3.49745300	-3.27893500	-0.58767300
C	2.13803100	-3.50655700	-0.38551000
H	4.19289200	-4.09374900	-0.76467300
H	1.73391500	-4.51125900	-0.42783500
C	3.89420800	0.33210000	-0.43216700
C	5.30741800	-1.43877200	-0.75646100
O	5.21791200	-0.05707000	-0.66130300
O	6.35198500	-2.00501700	-0.95536600
C	3.57407000	1.63082400	-0.34237800
H	2.52807800	1.86790300	-0.17503100
C	4.54035500	2.77265600	-0.45091000
H	5.55139300	2.39469500	-0.63304900
H	4.26794400	3.38574800	-1.32435300
C	4.53338700	3.67748200	0.79801700
H	4.81365300	3.07914400	1.67457800
H	3.50936700	4.03253000	0.98101000
C	5.47848900	4.87497200	0.66241300
H	5.45843400	5.50142100	1.56137900
H	6.51319400	4.54642300	0.50776200
H	5.20028300	5.50557200	-0.19105900
C	0.79402200	-0.03974400	0.11562100
C	-0.01460300	0.83587700	0.33956700
C	-0.96743300	1.90132300	0.67130700
H	-1.43497200	1.63275600	1.63568000
C	-0.24630800	3.22813200	0.85729700
H	0.37447000	3.29155200	1.74999400
C	-0.34465600	4.25503500	0.01512600
H	0.18213300	5.18751300	0.19470000
H	-0.94768200	4.18987500	-0.88599400
O	-1.96613600	1.94238700	-0.34831600
H	-2.61621100	2.60970300	-0.07122100
H	-0.69153600	-4.60750800	0.45286400
C	-0.64674000	-2.51548800	1.52572200
H	0.15859800	-2.47300200	2.25705200
C	-1.91561000	-2.40756100	1.94257600
H	-2.08560400	-2.26827100	3.01171300
C	-3.15330000	-2.41708900	1.08598700
H	-3.86411800	-3.14864700	1.49924400
H	-2.91736400	-2.75768000	0.07229300
C	-3.83973300	-1.03656500	1.03155700
H	-4.04562800	-0.69237600	2.05625200
H	-3.14675700	-0.30890600	0.58994200
C	-5.14417400	-1.04276100	0.22448700
H	-5.85609900	-1.74780100	0.67895400
H	-4.94116400	-1.42302400	-0.78720300
C	-5.79209100	0.34406300	0.12084500
H	-5.99695200	0.72648800	1.13237600
H	-5.06988600	1.04133000	-0.32979200
C	-7.08970200	0.35750900	-0.69693700
H	-6.88585900	-0.02631800	-1.70753900
H	-7.81033700	-0.34163500	-0.24690900
C	-7.73361000	1.74581400	-0.80467600
H	-7.93713000	2.13005800	0.20523800
H	-7.01400900	2.44394600	-1.25613300
C	-9.02909500	1.74996600	-1.62261900
H	-9.46343100	2.75457100	-1.68087700
H	-8.85110300	1.40398700	-2.64825000
H	-9.78117200	1.08729700	-1.17689200

M062X/6-311+G(d,p) Total Energy: -1349.9313314 Hartree

B3LYP/6-31G(d) Thermal correction to Gibbs Free Energy: 0.481297 Hartree

TS-3α

Imaginary frequency: -402.72 cm^{-1}

C	-0.79490200	-0.95620200	-0.42310700
C	-0.37174900	-1.69354400	0.68149700
C	0.89674000	-2.27045900	0.73440500
C	1.69695800	-2.06005600	-0.38737800
H	1.29430800	-2.65630700	1.66814300
H	2.70927100	-2.45589400	-0.37032900
C	-1.34891000	-1.49941800	1.76842200
C	-2.11080500	-0.42171700	-0.08548300
C	-3.00756900	0.21919800	-0.85415200
H	-2.72412500	0.39344800	-1.89062600
C	-4.37118000	0.67193800	-0.42089200
H	-4.49911500	0.48534700	0.65054000
O	-2.38059100	-0.71448300	1.24809500
O	-1.36628600	-1.89993500	2.90359900
C	-0.29825200	-1.25240000	-1.81920900
H	-1.04761900	-1.87625500	-2.32480300
H	-0.22164400	-0.33481200	-2.41205000
C	1.05878500	-2.01292900	-1.77375700
H	1.76268900	-1.58429800	-2.49293900
H	0.89765500	-3.05456500	-2.08386900
C	2.04267300	0.03959300	-0.25084600
C	0.96265600	0.68604100	-0.20185900
C	0.23065300	1.97458700	-0.09698200
O	1.02769100	3.06613500	-0.59242100
H	1.40912700	2.78172000	-1.43842000
H	-0.71721400	1.92365600	-0.65089900
C	-0.06234800	2.28601400	1.35351100
H	0.82017400	2.36307200	1.98592700
C	-1.28239000	2.46889000	1.85320000
H	-2.17316100	2.39503400	1.23560400

H	-1.43608100	2.68973100	2.90559500
Si	3.89570300	0.25026500	-0.16016200
C	4.48223900	-0.36892000	1.52680600
H	4.22241400	-1.42110300	1.68921700
H	5.57207800	-0.27769400	1.61382900
H	4.03162400	0.21239600	2.33904600
C	4.29891000	2.08622800	-0.37560800
H	5.32313300	2.29926900	-0.04503400
H	4.22859300	2.38179300	-1.42987400
H	3.61052000	2.72208300	0.18930800
C	4.76244600	-0.71430900	-1.54162500
H	4.38984800	-0.42210000	-2.53060500
H	5.83774100	-0.49602300	-1.52151600
H	4.65019000	-1.80003600	-1.44847500
H	-4.45691400	1.76015700	-0.56758700
C	-5.50755800	-0.01383400	-1.20753000
H	-5.35757100	0.15427000	-2.28327400
H	-5.44213300	-1.09850300	-1.05321000
C	-6.89469900	0.48825500	-0.79605900
H	-7.68410800	-0.01510500	-1.36540700
H	-7.08211100	0.30453500	0.26867800
H	-6.99409300	1.56699700	-0.96901000

M062X/6-311+G(d,p) Total Energy: -1293.873985 Hartree

B3LYP/6-31G(d) Thermal correction to Gibbs Free Energy: 0.374131 Hartree

TS-3β

Imaginary frequency: -415.75 cm^{-1}

C	-0.85383700	-0.88319900	0.63394700
C	-0.41790600	-1.93411500	-0.17579800
C	0.85249900	-2.48601700	-0.02780100
C	1.64129800	-1.89763900	0.96405300
H	1.26658000	-3.14521300	-0.78436400
H	2.65918700	-2.26119400	1.08211700
C	-1.34499600	-2.05985100	-1.31166200
C	-2.14207300	-0.45258700	0.10586500
C	-3.05305400	0.39928100	0.60528300
H	-2.81796500	0.85148900	1.56708200
C	-4.37020000	0.74772700	-0.02307500
H	-4.40288600	1.83152900	-0.21427600
H	-4.45905200	0.25298700	-0.99586400
C	-5.57401900	0.37188100	0.86526900
H	-5.46446100	0.85193000	1.84790600
H	-5.55803900	-0.71031200	1.04774100
C	-6.91333200	0.77501700	0.24137500
H	-7.06161600	0.28461200	-0.72814000
H	-7.75238800	0.49574100	0.88838600
H	-6.96446700	1.85827600	0.07632000
O	-2.35229400	-1.09764900	-1.11783000
O	-1.34618800	-2.78502500	-2.27114200
C	-0.38647600	-0.73082800	2.06074800
H	-0.35112900	0.32512200	2.34984300
H	-1.12787400	-1.20092300	2.72116400
C	0.99556000	-1.41791400	2.26182200
H	0.86915900	-2.30333900	2.89949800
H	1.68165500	-0.75130900	2.79208300
C	1.95279900	0.01972200	0.13388400
C	0.86649100	0.62576900	-0.06718600
C	0.04215700	1.79956400	-0.48168000
O	-0.52374400	1.59831300	-1.77466300
H	-1.11642500	0.82971400	-1.73598400
H	-0.75022900	1.96121400	0.26620000
C	0.87526000	3.05623400	-0.57283700
H	1.55686400	3.08927600	-1.41971500
C	0.80347300	4.06397200	0.29514500
H	0.11807400	4.04616300	1.14025700
H	1.42587100	4.94885200	0.19489800
Si	3.80650500	0.13854700	-0.06482300
C	4.26030100	1.79669400	-0.84944800
H	3.89385500	2.63917900	-0.25383500
H	5.35058400	1.89005400	-0.93282700
H	3.84093400	1.88924600	-1.85766700
C	4.40128100	-1.25854800	-1.19253900
H	5.47956200	-1.16443100	-1.37236000
H	4.22395800	-2.25290700	-0.76849400
H	3.89796500	-1.21711900	-2.16523700
C	4.63208400	0.01570800	1.63524400
H	4.41963200	-0.93202300	2.14273100
H	5.72154600	0.09476500	1.53197400
H	4.30398300	0.82861300	2.29351600

M062X/6-311+G(d,p) Total Energy: -1293.876207 Hartree

B3LYP/6-31G(d) Thermal correction to Gibbs Free Energy: 0.374299 Hartree

TS-4α

Imaginary frequency: -415.75 cm^{-1}

C	-0.04518800	-0.67845100	0.41292600
C	0.50427500	-1.52186700	-0.54661000
C	1.83200500	-1.96223600	-0.45481700
C	2.53346300	-1.55412100	0.65995100
H	2.32266000	-2.42286500	-1.30710700
H	3.59169700	-1.79540900	0.72268700
C	-0.41058700	-1.59213800	-1.69283600
C	-1.41701800	-0.41094600	-0.02960400
C	-2.45967300	0.10090500	0.64185900
H	-2.27031700	0.42396200	1.66341300
C	-3.86123500	0.23134200	0.12193100
H	-4.15173400	1.29363500	0.12820600
H	-3.90151800	-0.10080200	-0.92062100
C	-4.88579200	-0.55873200	0.96153300
H	-4.81982600	-0.23883500	2.01103100
H	-4.61439900	-1.62218000	0.94632800
C	-6.32136200	-0.38096400	0.45842400
H	-6.42193600	-0.72190000	-0.57897200
H	-7.02966200	-0.95332300	1.06791700
H	-6.62632000	0.67228100	0.49228100
O	-1.56165600	-0.88194400	-1.33222600
O	-0.30850300	-2.13005100	-2.76600000
C	0.39192200	-0.73732600	1.85862100
H	0.29024200	0.24017600	2.33813600
H	-0.29731500	-1.41269300	2.38290800

C	1.84555000	-1.27936300	1.98926000
H	1.82893600	-2.22755000	2.54557200
H	2.45678300	-0.59511600	2.58684500
C	1.39828100	1.11116800	-0.00471500
C	2.56290600	0.67174700	0.13534400
C	4.03605800	0.85227200	0.15860500
H	4.46347300	0.34178900	1.03620900
O	4.29147300	2.26317700	0.25917100
H	5.24793500	2.37823100	0.13084100
C	4.68858300	0.30254700	-1.09187200
H	4.27315800	0.67312600	-2.02745600
C	5.73198400	-0.52781100	-1.08503800
H	6.19847200	-0.86882600	-2.00505000
H	6.15967200	-0.90293900	-0.15664600
Si	0.47387500	2.68320600	-0.41686300
C	-0.54106300	3.25403100	1.07545700
H	0.09819500	3.38357100	1.95674800
H	-1.00463900	4.22501200	0.85959100
H	-1.34122800	2.55419600	1.33210500
C	-0.60552000	2.44221900	-1.94893200
H	0.01455600	2.18574400	-2.81615100
H	-1.35744100	1.65741500	-1.83702800
H	-1.12485000	3.38025000	-2.18423800
C	1.75146600	4.02197100	-0.80721900
H	2.44014300	4.17466300	0.02835800
H	2.35665900	3.75280500	-1.67958700
H	1.24495900	4.97139600	-1.02569600

M062X/6-311+G(d,p) Total Energy: -1293.873687 Hartree
B3LYP/6-31G(d) Thermal correction to Gibbs Free Energy: 0.374732 Hartree

TS-4β

Imaginary frequency: -415.75 cm^{-1}

C	-0.69805700	-1.06823900	-0.37252500
C	-0.25049800	-1.81493300	0.70699000
C	1.02401200	-2.38791600	0.75335200
C	1.85453000	-2.08494300	-0.33247300
H	1.40129900	-2.82689900	1.67173900
H	2.84573300	-2.53403000	-0.34464000
C	-1.23176800	-1.67967000	1.80077500
C	-2.01764500	-0.56709900	-0.02064100
C	-2.94478700	0.05141100	-0.77252200
H	-2.67787800	0.24926700	-1.80875700
C	-4.31524700	0.45671200	-0.31805100
H	-4.42766900	0.24727800	0.75069100
O	-2.27590300	-0.89309000	1.31079500
O	-1.23704700	-2.11757200	2.92217900
C	-0.12271700	-1.20030600	-1.75922700
H	-0.83498100	-1.76068000	-2.38152400
H	-0.01881300	-0.21960500	-2.23162900
C	1.23314500	-1.96041900	-1.72557000
H	1.94902200	-1.49406600	-2.40859900
H	1.08060100	-2.98553600	-2.08891100
C	1.38773400	0.65557000	0.02842200
C	2.32460000	-0.18678900	0.03892700
C	3.80653900	-0.23169600	0.27910800
H	4.03237000	-1.06883100	0.95952300
O	4.17959600	1.00782600	0.89051400
H	5.14822700	0.99873300	0.96547700
C	4.58215900	-0.41715200	-1.00624800
H	4.40379400	0.35075300	-1.75799700
C	5.46045600	-1.39789700	-1.22162600
H	6.01904900	-1.47397900	-2.15051100
H	5.65559800	-2.16531300	-0.47452100
Si	0.64254000	2.34027700	0.19933200
C	-0.46823500	2.38584100	1.72625800
H	0.11214600	2.17537100	2.63206400
H	-0.91621000	3.38075000	1.84404500
H	-1.28018600	1.65396100	1.67425900
C	-0.32933700	2.78284800	-1.36368100
H	0.29867200	2.71085800	-2.25953200
H	-1.20067100	2.13557500	-1.50121600
H	-0.69079100	3.81678100	-1.29780900
C	2.05041800	3.58948700	0.40612900
H	2.70639400	3.30672700	1.23465800
H	2.66894000	3.64206300	-0.49683700
H	1.64808400	4.59214400	0.60182100
H	-4.42940400	1.54494800	-0.44272700
C	-5.43981900	-0.24402300	-1.10851700
H	-5.34511300	-1.32940500	-0.97569400
H	-5.30393300	-0.05169300	-2.18208600
C	-6.83588800	0.21352900	-0.67579600
H	-7.61698600	-0.29967200	-1.24787700
H	-7.00871700	0.00505100	0.38683600
H	-6.96487100	1.29235700	-0.82729600

M062X/6-311+G(d,p) Total Energy: -1293.869804 Hartree
B3LYP/6-31G(d) Thermal correction to Gibbs Free Energy: 0.373878 Hartree

TS-5α

Imaginary frequency: -378.43 cm^{-1}

C	0.22823100	-1.57526500	0.75228700
C	1.16896500	-2.10358900	-0.10521100
C	2.55595000	-1.97270600	0.08782300
C	2.95589600	-1.14969100	1.15248100
H	3.25414700	-2.30913100	-0.67082600
H	4.02109800	-1.14324600	1.37958500
C	0.46071900	-2.61363200	-1.29928800
C	-1.06062900	-1.72091500	0.12667700
C	-2.27916300	-1.33583700	0.55601600
H	-2.32113500	-0.86452500	1.53544900
C	-3.57008300	-1.55788300	-0.17205600
H	-3.36649200	-1.92676300	-1.18263000
O	-0.89437500	-2.35259400	-1.10557600
O	0.87247400	-3.15705700	-2.29182300
C	0.54080700	-1.05624800	2.12503200
H	0.13917900	-0.04367200	2.24110400
H	0.02024500	-1.66857500	2.87467500
C	2.07031200	-1.10543600	2.40360400
H	2.29857900	-2.01865900	2.96948000
H	2.36227800	-0.26383300	3.03890700

C	2.82394400	0.64347900	0.41759200
C	1.67870900	1.14145300	0.24382500
C	0.45687600	1.68346800	0.10920800
C	-0.66509700	2.18960000	-0.00162200
C	4.23606000	1.08430800	0.14266000
Si	-2.25823800	3.07701500	-0.22540600
C	-3.07958500	2.46711400	-1.81432100
C	-1.91903800	4.93410400	-0.34155500
H	-2.85392700	5.49206500	-0.47945000
H	-1.43525800	5.30804100	0.56776600
H	-1.26223000	5.16558600	-1.18748900
C	-3.36636800	2.72622900	1.26822700
H	-4.31840400	3.26361100	1.17497500
H	-3.59234400	1.65786300	1.35852100
H	-2.89174400	3.04954400	2.20166900
H	-4.04016100	2.97292400	-1.97370800
H	-2.44829100	2.66617700	-2.68762800
H	-3.26672300	1.38826200	-1.77960700
H	4.85125200	0.89326600	1.03832600
C	4.83199100	0.33135300	-1.02566000
H	4.27709100	0.42210100	-1.95813500
C	5.97124900	-0.36027500	-0.96673000
H	6.38395900	-0.85978900	-1.83888700
H	6.53679600	-0.45373400	-0.04112000
O	4.19697500	2.48304400	-0.13912000
H	5.09538800	2.73450400	-0.41033500
H	-4.09490400	-0.59643100	-0.28045600
C	-4.50745200	-2.54196800	0.56175700
H	-4.68559700	-2.18090600	1.58423200
H	-3.99890900	-3.50942600	0.66014300
C	-5.84506800	-2.72710700	-0.16087800
H	-6.49049600	-3.43220000	0.37480800
H	-6.38602300	-1.77649600	-0.24520000
H	-5.69600700	-3.11523900	-1.17554600

M062X/6-311+G(d,p) Total Energy: -1370.01809 Hartree
B3LYP/6-31G(d) Thermal correction to Gibbs Free Energy: 0.376712 Hartree

TS-5β

Imaginary frequency: -387.35 cm^{-1}

C	0.19102400	-1.45757500	-0.64697300
C	1.16306700	-1.90301400	0.22193200
C	2.53905200	-1.68068900	0.02848700
C	2.89489000	-0.86307500	-1.05705800
H	3.25477500	-1.95799600	0.79461600
H	3.95293600	-0.78683000	-1.29253100
C	0.48865300	-2.44082700	1.42311100
C	-1.08682000	-1.68236000	-0.02120400
C	-2.32734600	-1.38864900	-0.46007700
H	-2.39667600	-0.93654800	-1.44735800
C	-3.60557500	-1.66909800	0.27154500
H	-3.38110700	-2.03850900	1.27760600
O	-0.88145600	-2.27996900	1.22168100
O	0.93336300	-2.93530700	2.42696200
C	0.47172500	-0.92978000	-2.02279700
H	-0.01379400	-1.57538200	-2.76816000
H	0.00926400	0.05644300	-2.14237800
C	2.00107400	-0.88879300	-2.30210300
H	2.24474500	-0.04037400	-2.94757600
H	2.28351800	-1.79427000	-2.85597600
C	2.68814700	0.91379000	-0.34357100
C	1.51940800	1.36763500	-0.18431500
C	0.26784300	1.83686500	-0.05881000
C	-0.88107100	2.28401200	0.04259900
C	4.07289000	1.43299000	-0.02306100
O	4.91773400	1.24004500	-1.15354200
C	5.69548500	0.01909300	1.29990100
H	6.04460400	-0.39444400	2.24133100
H	6.26322700	-0.23824400	0.40966900
C	-4.52164000	-2.67620600	-0.46256100
H	-5.48121200	-2.71432100	0.06939500
H	-4.74366900	-2.29572000	-1.46954300
C	-3.93112400	-4.08569600	-0.55975800
H	-2.98644800	-4.08524900	-1.11505900
H	-3.72726400	-4.49693500	0.43601500
H	-4.62100700	-4.76715600	-1.07028600
H	-4.15974400	-0.72623400	0.39112300
Si	-2.52820600	3.06973800	0.24346600
C	-3.60138600	2.62484500	-1.25058100
C	-2.32012700	4.94823200	0.33359800
H	-3.29282200	5.44170200	0.45492600
H	-1.68963200	5.23854900	1.18155900
H	-1.85571200	5.34184100	-0.57762800
C	-3.31794100	2.43179600	1.83769200
H	-4.31057200	2.87601000	1.98360900
H	-3.43374300	1.34259200	1.81855900
H	-2.70739500	2.68486600	2.71182300
H	-4.58680200	3.10079900	-1.17136800
H	-3.14266200	2.96331300	-2.18660800
H	-3.75659700	1.54275300	-1.32466800
C	4.61376500	0.79638200	1.24114600
H	4.04993800	1.02906100	2.14315100
H	5.78664400	1.60574100	-0.92162400
H	3.95100800	2.51080200	0.16520000

M062X/6-311+G(d,p) Total Energy: -1370.021489 Hartree
B3LYP/6-31G(d) Thermal correction to Gibbs Free Energy: 0.379684 Hartree

TS-6α

Imaginary frequency: -422.74 cm^{-1}

C	-1.66321100	-0.76940600	-0.34816400
C	-1.28999000	-1.68139600	0.64822000
C	-0.07322100	-2.36391200	0.57437700
C	0.68842200	-2.13438200	-0.55589500
H	0.32656100	-2.87689900	1.44438000
H	1.70484200	-2.51701500	-0.59575200
C	-2.18865800	-1.52439800	1.79812700
C	-2.93031100	-0.18125700	0.11094800
C	-3.81188300	0.59579300	-0.53479500
H	-3.54655000	0.88918600	-1.54729900
C	-5.11718300	1.09038600	0.01841500

H	-5.22967100	0.75836900	1.05588200
O	-3.15849000	-0.59089600	1.42410300
O	-2.19481700	-2.04665000	2.88429700
C	-1.29972800	-1.03783000	-1.79457800
H	-2.09396700	-1.66558400	-2.21968400
H	-1.28668700	-0.10870200	-2.36858900
C	0.06455200	-1.78517400	-1.89724400
H	0.78046800	-1.20765000	-2.48986000
H	-0.07997400	-2.72997300	-2.44036400
C	-0.02895100	0.68290200	-0.05853600
C	1.09564200	0.11792100	-0.10276100
C	2.46448500	0.05113500	-0.09392100
C	3.69013900	-0.02214100	-0.09066300
C	-0.69184200	2.01466300	0.01174300
C	-6.33990100	0.63918700	-0.81111500
H	-7.22821700	1.15324200	-0.42135200
H	-6.21832600	0.98039200	-1.84896100
C	-6.57139700	-0.87440200	-0.78888500
H	-5.71077100	-1.41478000	-1.19904800
H	-6.72828500	-1.23384600	0.23517300
H	-7.45307600	-1.14774300	-1.37956200
H	-5.10122300	2.19092800	0.03597100
Si	5.52867600	-0.07461700	-0.06587900
C	6.11564200	0.37816700	1.67259400
C	6.19086700	1.16689900	-1.32854500
H	7.28794200	1.15831100	-1.34205700
H	5.84243800	0.93174700	-2.34061400
H	5.86672800	2.18661800	-1.09222700
C	6.09941200	-1.82076500	-0.51457200
H	7.19479800	-1.88364900	-0.50904100
H	5.71915100	-2.56075300	0.19874500
H	5.75432000	-2.11021600	-1.51375000
H	7.21114000	0.35672800	1.72907200
H	5.78484400	1.38371900	1.95552000
H	5.72784100	-0.32334200	2.41968100
H	-1.55123800	1.96357100	0.69572200
O	-1.14984200	2.31551500	-1.32075100
H	-1.59308900	3.17847800	-1.26676200
C	0.26170200	3.08025900	0.49411900
H	1.15577000	3.21285200	-0.11256700
C	0.03628700	3.83559600	1.56932900
H	-0.85103700	3.70270800	2.18552100
H	0.73375000	4.60711200	1.88375700

M062X/6-311+G(d,p) Total Energy: -1370.017572 Hartree

B3LYP/6-31G(d) Thermal correction to Gibbs Free Energy: 0.380007 Hartree

TS-6β

Imaginary frequency: -426.22 cm^{-1}

C	1.64485700	-0.71385500	-0.39565200
C	1.34365700	-1.68139600	0.56983500
C	0.13684900	-2.38348000	0.52639700
C	-0.68526700	-2.11629300	-0.55289200
H	-0.20928800	-2.94305600	1.39054100
H	-1.69516100	-2.51744100	-0.56195300
C	2.32512700	-1.57624100	1.66127600
C	2.93931800	-0.14401400	0.00469700
C	3.76479700	0.66785600	-0.67354500
H	3.43700100	0.98250400	-1.66306800
C	5.10445300	1.15439400	-0.19958300
H	5.28143200	0.80258500	0.82228700
O	3.27271600	-0.63257100	1.26005400
O	2.41119400	-2.15888000	2.71142800
C	1.19886800	-0.89831700	-1.83218800
H	1.10062800	0.06609500	-2.34074100
H	1.99360800	-1.44384000	-2.35700100
C	-0.12893800	-1.70961900	-1.90797300
H	0.04570700	-2.63418800	-2.47648400
H	-0.89249200	-1.15729500	-2.46376700
C	0.00485000	0.67909500	0.09671800
C	-1.11119700	0.10590300	-0.00659800
C	-2.48033700	0.03984400	-0.04080900
C	-3.70495300	-0.03686700	-0.08339000
C	0.68337800	1.95444000	0.45273400
Si	-5.54323600	-0.08825800	-0.12289900
C	-6.09271600	-0.52272600	-1.87955000
C	-6.21565000	1.60854900	0.36866700
H	-7.31278900	1.61048800	0.35627900
H	-5.89207100	1.88736600	1.37782600
H	-5.87116300	2.38815700	-0.32007900
C	-6.14210200	-1.40406800	1.09484100
H	-7.23807100	-1.45523600	1.10191100
H	-5.76260600	-2.39730500	0.82961500
H	-5.81129600	-1.18213000	2.11568300
H	-7.18743700	-0.55775600	-1.94364700
H	-5.73935700	0.21997300	-2.60372900
H	-5.70881600	-1.50141600	-2.18894500
H	1.44450600	2.18767200	-0.30832700
O	1.30909000	1.73115400	1.72156600
H	1.86870500	2.50353900	1.90367400
C	-0.29770400	3.09971800	0.53276200
H	-1.09639900	2.97326100	1.26131100
C	-0.20070400	4.20820700	-0.20127000
H	0.59425500	4.34214500	-0.93301700
H	-0.91330800	5.02291100	-0.10671500
H	5.09104200	2.25430100	-0.16048500
C	6.27232900	0.71816200	-1.11188500
H	6.08670900	1.07789800	-2.13402700
H	7.18380800	1.22559300	-0.76971500
C	6.50364600	-0.79546200	-1.13091200
H	6.72335500	-1.17294800	-0.12514700
H	5.61876800	-1.32889200	-1.49608100
H	7.34666600	-1.05797500	-1.78006600

M062X/6-311+G(d,p) Total Energy: -1370.015796 Hartree

B3LYP/6-31G(d) Thermal correction to Gibbs Free Energy: 0.37931 Hartree

TS-7α

Imaginary frequency: -365.07 cm^{-1}

C	-0.33741900	-1.48928700	0.63174600
C	0.40530100	-2.25876300	-0.24724000
C	1.78623800	-2.44321400	-0.11972700
C	2.40469900	-1.69879700	0.90237200

H	2.35405700	-2.93297100	-0.90425600
H	3.46490800	-1.88527700	1.06387000
C	-0.45947200	-2.59882400	-1.39729400
C	-1.65674200	-1.34374200	0.06903600
C	-2.73377200	-0.68455600	0.53734900
O	-1.69954200	-2.01382500	-1.15502300
O	-0.23856100	-3.24170100	-2.39164100
C	0.12839700	-1.12501400	2.01458400
H	-0.05415900	-0.06374400	2.21336900
H	-0.47350300	-1.67303500	2.75318600
C	1.62764800	-1.49337900	2.20878200
H	1.69555800	-2.44074900	2.75989900
H	2.12554200	-0.73754900	2.82366100
C	-0.67661300	2.22861700	-0.39241200
C	0.28681500	1.49236000	-0.21587200
C	1.34645000	0.68531800	-0.00420100
C	2.45633300	0.10296400	0.18708200
C	-1.72523500	3.21713100	-0.63078900
Si	4.23859500	0.61413800	-0.11942200
C	5.17369800	0.63023200	1.52733000
C	4.24120800	2.34191600	-0.87746400
H	5.26630900	2.68550200	-1.06416100
H	3.70266900	2.35652200	-1.83149100
H	3.75826900	3.06813600	-0.21432300
C	5.03293200	-0.62433700	-1.30819200
H	6.06751100	-0.33071100	-1.52587400
H	5.05958800	-1.64066400	-0.89896000
H	4.49050800	-0.66200600	-2.25963100
H	6.21784500	0.92783700	1.36853300
H	4.72856200	1.34644500	2.22759000
H	5.18413200	-0.35175600	2.01424700
H	-2.28392700	2.92695900	-1.53648800
O	-1.09306200	4.49439500	-0.83063100
H	-1.81367300	5.13723100	-0.94268900
C	-2.69545900	3.29833100	0.52980900
H	-2.23891500	3.46947800	1.50316900
C	-4.02120700	3.24674400	0.38976300
H	-4.48729900	3.08965100	-0.58118500
H	-4.69153800	3.35979900	1.23740000
H	-2.62196200	-0.19163600	1.50029000
C	-4.07533500	-0.62426100	-0.12591400
H	-4.36768600	0.43026800	-0.24073200
H	-4.01253400	-1.05457000	-1.13081800
C	-5.17446000	-1.34835600	0.68134600
H	-4.89681700	-2.40419000	0.79306900
H	-5.21442000	-0.93051300	1.69702500
C	-6.55376000	-1.23994100	0.02431200
H	-6.54831600	-1.67777200	-0.98109100
H	-7.31619700	-1.76356600	0.61201600
H	-6.86697400	-0.19284100	-0.07177800

M062X/6-311+G(d,p) Total Energy: -1370.017729 Hartree

B3LYP/6-31G(d) Thermal correction to Gibbs Free Energy: 0.379078 Hartree

TS-7β

Imaginary frequency: -365.72 cm^{-1}

C	-0.46159700	-1.54102900	-0.46049600
C	0.15264500	-2.09065900	0.65233500
C	1.52197400	-2.37085700	0.70487100
C	2.26648600	-1.95234000	-0.41498800
H	1.99321900	-2.66091000	1.63838300
H	3.32098500	-2.22241000	-0.42330000
C	-0.81902400	-2.06200300	1.76705100
C	-1.79962000	-1.16840400	-0.07601000
C	-2.77261800	-0.57342600	-0.79314000
H	-2.54720300	-0.36810600	-1.83768200
C	-4.13932500	-0.21507100	-0.29165900
H	-4.19273800	-0.38765100	0.78831200
O	-1.98665800	-1.48727100	1.26933500
O	-0.72531900	-2.43050200	2.90971200
C	0.13260200	-1.57568100	-1.84150300
H	-0.45412800	-2.25924300	-2.47119100
H	0.05300400	-0.59256200	-2.31756000
C	1.60658700	-2.06922400	-1.79459300
H	2.20564000	-1.54001700	-2.54188700
H	1.63771100	-3.13358900	-2.06302900
C	-0.68564600	2.26918700	-0.32149400
C	0.26424400	1.49685100	-0.27287000
C	1.29828400	0.63198300	-0.23240100
C	2.38118100	-0.02758200	-0.20900700
C	-1.74994200	3.27040900	-0.31921700
C	-5.27596800	-0.98602600	-1.00194100
H	-6.23450600	-0.56313900	-0.67318300
H	-5.21499500	-0.80397800	-2.08424300
C	-5.25864900	-2.49267900	-0.72902700
H	-4.32440000	-2.94908300	-1.07459800
H	-5.34861500	-2.69974300	0.34395200
H	-6.08815600	-2.99517400	-1.23937900
H	-4.30451300	0.86168400	-0.45214100
Si	4.17494500	0.47972100	0.03051600
C	4.85942100	-0.40333400	1.55692600
C	4.24021900	2.34976100	0.27520000
H	5.27446700	2.69134500	0.40693000
H	3.81839300	2.87836600	-0.58681400
H	3.67097000	2.65306400	1.16092800
C	5.16980100	-0.00430900	-1.50612800
H	6.22181500	0.28257900	-1.38372300
H	5.14530600	-1.08293500	-1.69955700
H	4.79006500	0.50483400	-2.39945600
H	5.89976800	-0.10288400	1.73407200
H	4.28213800	-0.14803700	2.45274300
H	4.84623100	-1.49409300	1.45155200
H	-2.64406500	2.84423700	-0.80520000
O	-1.29280400	4.41650000	-1.05593200
H	-1.99086000	5.08743400	-0.97028200
C	-2.11357800	3.65958500	1.09917400
H	-1.28953900	4.04947900	1.69379900
C	-3.34801000	3.58161600	1.59558000
H	-4.17729700	3.18971500	1.00988200
H	-3.57647100	3.89429800	2.61053600

M062X/6-311+G(d,p) Total Energy: -1370.01827 Hartree

B3LYP/6-31G(d) Thermal correction to Gibbs Free Energy: 0.378561 Hartree

TS-8α

Imaginary frequency: -426.07 cm^{-1}

C	1.18851200	-0.97531400	-0.52626200
C	0.72245900	-1.92457800	0.39109300
C	-0.53774400	-2.50513600	0.24776600
C	-1.26204300	-2.11500100	-0.86937100
H	-0.99149600	-3.05533100	1.06699400
H	-2.29341100	-2.44354000	-0.96631400
C	1.60935800	-1.92161900	1.56235700
C	2.50264100	-0.55282300	-0.02551200
C	3.48832400	0.11747200	-0.63969100
H	3.28934300	0.45331200	-1.65538900
O	2.66461900	-1.05160700	1.26509200
O	1.55074500	-2.51125000	2.61079400
C	0.81911500	-1.07165500	-1.99059700
H	0.84139000	-0.08623900	-2.46461200
H	1.59217400	-1.67292800	-2.48707100
C	-0.56862000	-1.75443400	-2.17624300
H	-0.43903100	-2.68839700	-2.74087400
H	-1.23154500	-1.12721700	-2.77967600
C	-4.13203400	0.10187200	-0.45400000
C	-2.91786100	0.07055300	-0.36905500
C	-1.54329500	0.01010700	-0.28855800
C	-0.42257800	0.57637000	-0.10510400
C	-5.59348500	0.13344600	-0.52054300
Si	0.19475200	2.26630300	0.40356700
C	1.20092900	2.14028900	1.99658500
C	-1.33768200	3.33011600	0.72135700
H	-1.04594100	4.34305900	1.02742500
H	-1.96298500	3.41565000	-0.17419200
H	-1.95927700	2.90583100	1.51766800
C	1.20088200	3.05202900	-0.99281300
H	1.46825600	4.08171400	-0.72346700
H	2.12921200	2.50852000	-1.19130300
H	0.62203500	3.09477100	-1.92284200
H	1.55919500	3.13467200	2.29237000
H	0.58060300	1.75663700	2.81494800
H	2.06857500	1.48158800	1.90206100
H	-5.91668600	-0.46226500	-1.39180400
O	-6.00625800	1.49858400	-0.67713800
H	-6.97749800	1.49280800	-0.64552500
C	-6.20566300	-0.47069700	0.72608000
H	-5.92621500	0.01718200	1.65819200
C	-7.05352900	-1.49908900	0.71039200
H	-7.49268900	-1.89272200	1.62277700
H	-7.33574600	-1.99357800	-0.21735200
C	4.84341400	0.41007300	-0.06462500
H	4.99232200	1.50053700	-0.02574300
H	4.89341000	0.04777900	0.96744000
C	5.98912300	-0.20887500	-0.89107900
H	5.85846900	-1.29829800	-0.92082100
H	5.91615400	0.13876300	-1.93123600
C	7.37200700	0.13412400	-0.32934700
H	7.48242400	-0.22962000	0.69925700
H	8.16837200	-0.31859600	-0.93063900
H	7.53784700	1.21847000	-0.31718000

M062X/6-311+G(d,p) Total Energy: -1370.01377 Hartree

B3LYP/6-31G(d) Thermal correction to Gibbs Free Energy: 0.379038 Hartree

TS-8β

Imaginary frequency: -428.22 cm^{-1}

C	-1.33793000	-0.88960400	-0.33573800
C	-0.94460200	-1.71214100	0.72691900
C	0.25391700	-2.42366900	0.67989500
C	0.99093800	-2.29578300	-0.48942700
H	0.67039300	-2.86991900	1.57827800
H	1.98650400	-2.72919500	-0.53303100
C	-1.80478500	-1.42872000	1.88394200
C	-2.59803900	-0.26753600	0.09072500
C	-3.52912600	0.38088400	-0.62419400
H	-3.31887900	0.52138400	-1.68285300
C	-4.83557100	0.90665300	-0.10247600
H	-4.90939400	0.70859100	0.97203600
O	-2.78022500	-0.52428100	1.44768900
O	-1.78110200	-1.83629200	3.01681200
C	-1.00702500	-1.26517300	-1.76394900
H	-1.84170800	-1.86573900	-2.14911400
H	-0.94761700	-0.37589200	-2.39799700
C	0.30789900	-2.09723000	-1.83568200
H	1.01445500	-1.64752700	-2.53950200
H	0.08213900	-3.09706300	-2.23193300
C	4.05575000	-0.36767800	-0.44709200
C	2.84813500	-0.24036700	-0.35972000
C	1.47585000	-0.14563600	-0.27297200
C	0.41848000	0.55095400	-0.18579400
C	5.51297500	-0.46928500	-0.52620300
C	-6.06391600	0.31639300	-0.82921500
H	-6.95788000	0.85090500	-0.48244300
H	-5.98135700	0.52376200	-1.90567500
C	-6.24569100	-1.18761500	-0.60519300
H	-5.37907000	-1.75213800	-0.96766400
H	-6.36420400	-1.41544400	0.46087900
H	-7.13306100	-1.56063500	-1.12902700
H	-4.85316700	2.00008300	-0.22629200
Si	-0.03161400	2.35007400	0.03666100
C	-0.99664000	2.98719500	-1.46080800
C	1.59772000	3.30807600	0.15355600
H	1.40551600	4.37778300	0.30695300
H	2.20982100	2.95417400	0.99059100
H	2.19243200	3.20142000	-0.76063700
C	-1.00232100	2.58578300	1.63893000
H	-1.27177200	3.64244900	1.76317800
H	-1.92214900	1.99521700	1.66926000
H	-0.39420200	2.29639600	2.50390300
H	-1.15973700	4.06816300	-1.36480600
H	-0.44192800	2.81910300	-2.39138400
H	-1.97637500	2.51018800	-1.55551100

H	5.85844900	0.09723400	-1.40794800
O	5.85238700	-1.85736100	-0.66889200
H	6.82294000	-1.90235600	-0.64897300
C	6.16679400	0.11660400	0.70757300
H	5.87356600	-0.34739400	1.64760800
C	7.06382000	1.10179500	0.67218200
H	7.53131400	1.48255600	1.57590700
H	7.35930000	1.57293300	-0.26346300

M062X/6-311+G(d,p) Total Energy: -1370.014811 Hartree

B3LYP/6-31G(d) Thermal correction to Gibbs Free Energy: 0.379714 Hartree

9. Total synthesis of falcarinphthalide A (1)

Table S12. Outcomes of the Diels-Alder between compounds **3** and **4** under a range of different conditions

Entry	Solvent	Catalysis	Temp [°C]	Time [h]	product
1	THF	-	60	24	-
2	THF	$AlCl_3$	60	8	-
3	THF	$AlCl_3$	60	24	-
4	THF	$AlCl_3$	rt	24	-
5	DCM	$AlCl_3$	-15	8	-
6	THF	-	rt	24	-
7	DCM	$AlCl_3$	rt	8	-
8	Toluene	$AlCl_3$	100	30	-
9	DCM	$AlCl_3$	rt	24	-
10	DCM	$AlCl_3$	45	24	-
11	ACN	$LiBF_4$	rt	24	-
12	ACN	$LiBF_4$	75	24	-
13	EtOH	HCOOH	90	24	-
14	ACN	Imidazolinium salts	80	12	-
15	ACN	CF_3COOH	80	12	-
16	ACN	CF_3COOH	rt	12	-
17	DMF	$LiBF_4$	rt	4	-
18	DMF	$LiBF_4$	40	4	-
19	EtOH	$LiBF_4$	rt	4	-
20	EtOH	$LiBF_4$	40	4	-
21	DMF	$LiBF_4$	rt	8	-
22	DMF	$LiBF_4$	40	8	-
23	EtOH	$LiBF_4$	rt	8	-
24	EtOH	$LiBF_4$	40	8	-
25	DMF	$LiBF_4$	rt	12	-
26	DMF	$LiBF_4$	40	12	-
27	EtOH	$LiBF_4$	rt	12	-
28	EtOH	$LiBF_4$	40	12	-
29	MeOH/H_2O	$MgCl_2$	rt	3	-
30	ACN	$MgCl_2$	70	8	-
31	Toluene	$Ni(cod)_2$,pph_3	120	6	-
32	DCM	$MgCl_2$	rt	24	-

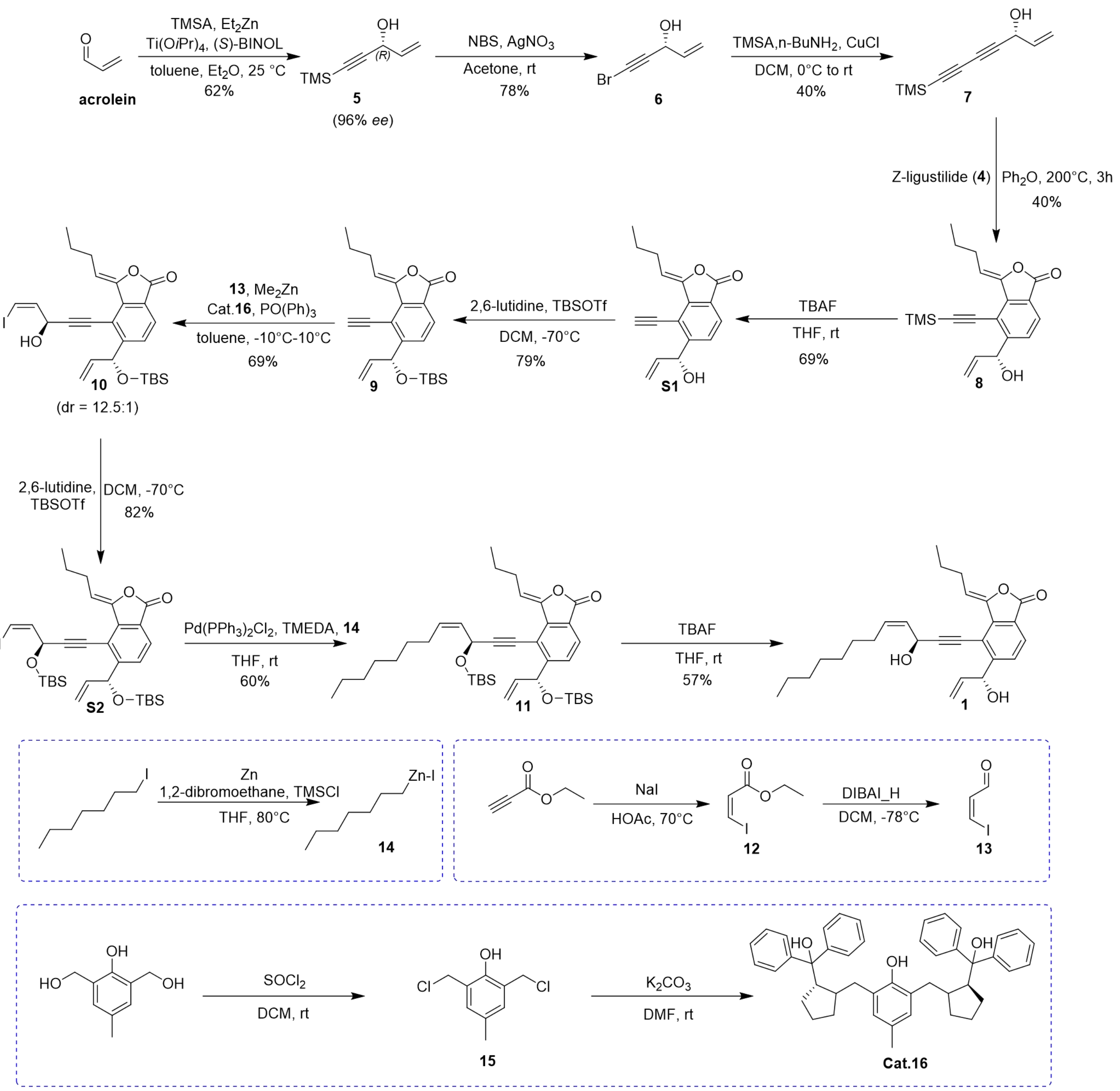

Figure S19. Total synthesis of falcarinphthalide A (**1**)

9.1 Experimental Procedures and Spectroscopic Data

Synthesis of (*R*)-5-(trimethylsilanyl)pent-1-en-4-yn-3-ol (5)

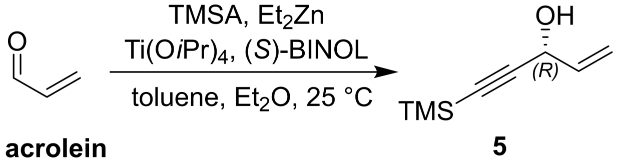

To a solution of trimethylsilylacetylene (TMSA, 13.2 mL, 97.0 mmol) in toluene (48 mL) was added diethylzinc (Et_2Zn, 97.0 mL, 1.0 M in *n*-hexanes, 97.0 mmol) at 25 °C under nitrogen and the mixture was refluxed (120 °C) for 2 hours. Then the reaction was cooled to 25 °C and (*S*)-1-(2-hydroxynaphthalen-1-yl)naphthalen-2-ol ((*S*)-BINOL, 2.78 g, 9.7 mmol), ethyl ether (Et_2O, 400 mL) and titanium(IV) isopropoxide ($Ti(OiPr)_4$, 6.91 g, 24.0 mmol) were added sequentially. Then the mixture was stirred for another 2 hours. Then the acrolein (1.6 mL, 24.0 mmol) was slowly added via syringe, the reaction was stirred for 16 hours at 25 °C. Then the reaction was quenched with water (300 mL) at 0 °C, filtered through a pad of Celite and the aqueous phase was extracted with diethyl ether twice (500 mL). The combined organic phases were washed with brine, dried over anhydrous Na_2SO_4 and concentrated under reduced pressure to give a residue, which was purified by silica gel chromatography (PE:EtOAc = 10:1) to obtain **5** (2.60 g, 90 % purity, 62 % yield, *ee* = 96% (**Figure S21**)) as yellow oil.[14]

***R**f*= 0.5 (PE:EtOAc = 8:1);

$[\alpha]_D^{27}$= -24.9 (*c* = 1.0 in $CHCl_3$);

^{1}H NMR (400 MHz, $CDCl_3$) δ 5.95 (ddd, *J* = 17.0, 10.2, 5.3 Hz, 1H), 5.46 (br d, *J* = 17.1 Hz, 1H), 5.21 (br d, *J* = 10.2 Hz, 1H), 4.86 (br d, *J* = 5.3 Hz, 1H), 0.18 (s, 9H);

^{13}C NMR (101 MHz, $CDCl_3$) δ 136.7, 116.5, 104.1, 91.1, 63.4, -0.2;

IR (neat): ν_{max} = 3429, 2956, 1590, 1255, 1111, 1030, 850 cm^{-1};

HRMS (ESI): *m/z* calcd. for $C_8H_{15}OSi$ $[M+H]^+$: 155.0892, found: 155.0899.

Synthesis of (*R*)-5-bromopent-1-en-4-yn-3-ol (6)

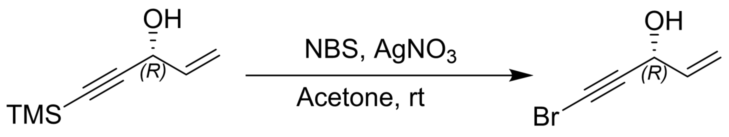

5 6

To a solution of (3*R*)-5-(trimethylsilyl)pent-1-en-4-yn-3-ol (**5**, 15.00 g, 97.2 mmol) in acetone (300 mL) was added silver nitrate ($AgNO_3$, 3.30 g, 19.4 mmol) and *N*-bromosuccinimide (NBS, 24.20 g, 13.6 mmol) at 25 °C. After stirred for 4 hours, the reaction mixture was poured into ice water (300 mL) and extracted with diethyl ether (400 mL × 2). The combined organic layers were washed with brine (300 mL), dried over Na_2SO_4 and filtered. The filtrate was concentrated under 20 °C to give a residue, which was purified by silica gel column chromatography (PE:EtOAc = 8:1 to 5:1) to give the **6** (13.5 g, 90 % purity, 78 % yield) as yellow oil. [14]

R_f = 0.4 (PE:EtOAc = 8:1);

$[\alpha]_D^{27}$ = -24.5 (*c* = 1.0 in $CHCl_3$);

^{1}H NMR (400 MHz, $CDCl_3$) δ 5.95 (ddd, *J* = 17.0, 10.1, 5.4, Hz, 1H), 5.47 (br d, *J* = 17.1 Hz, 1H), 5.25 (br d, *J* = 10.1 Hz, 1H), 4.90 (br d, 5.3 Hz, 1H);

^{13}C NMR (101 MHz, $CDCl_3$) δ 136.2, 117.0, 78.8, 64.0, 46.9;

IR (KBr): ν_{max} =3402, 2960, 2930, 2875, 1726, 1642, 1278, 1064, 849 cm^{-1};

HRMS (ESI): *m*/*z* calcd. for $C_5H_6{}^{79}BrO$ $[M+H]^+$: 160.9602, found: 160.9597.

Synthesis of (*R*)-7-(trimethylsilanyl)hept-1-en-4,6-diyn-3-ol (7)

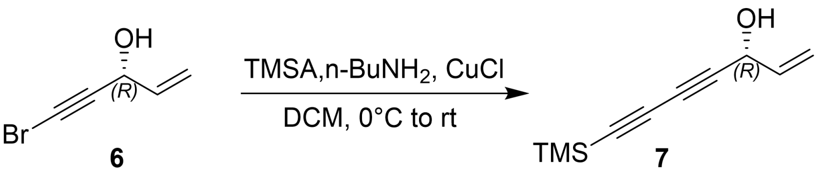

To a solution of *n*-butylamine (*n*-$BuNH_2$, 200 mL, 30 % in water) in dichloromethane (DCM, 300 mL) was added copper(I) chloride (CuCl, 2.95 g, 29.8 mmol) and trimethylsilylacetylene (TMSA, 10.70 g, 109 mmol) at 0 °C under nitrogen. Then (3*R*)-5-bromopent-1-en-4-yn-3-ol (**6**, 16.00 g, 99.4 mmol) in DCM (100 mL) was added to the above solution at 0 °C for 30 minutes, then the reaction was stirred at 25 °C for 3.5 hours. The reaction was quenched with ammonium chloride aqueous solution (300 mL) and extracted with DCM (300 mL) twice. The combined organic phases were washed with brine, dried over anhydrous Na_2SO_4 and concentrated under reduced pressure to give a residue, which was purified by silica gel chromatography (PE:EtOAc = 10:1) to obtain the **7** (9.00 g, 80 % purity, 40 % yield) as brown oil.[15]

R_f = 0.5 (PE:EtOAc = 8:1);

$[\alpha]_D^{27}$ = -53.5 (*c* = 1.0 in $CHCl_3$);

^{1}H NMR (400 MHz, $CDCl_3$) δ 5.93 (ddd, *J* = 17.0, 10.0, 5.4 Hz, 1H), 5.47 (br d, *J* = 17.0 Hz, 1H), 5.25 (br d, *J* = 10.2 Hz, 1H), 4.92 (br d, *J* = 5.3 Hz, 1H), 0.19 (s, 9H);

^{13}C NMR (101 MHz, $CDCl_3$) δ 135.7, 117.3, 88.4, 87.0, 76.0, 71.1, 63.4, -0.5;

IR (neat): ν_{max} =3358, 2956, 2107, 1604, 1255, 926, 851 cm^{-1};

HRMS (ESI): *m*/*z* calcd. for $C_{10}H_{15}OSi$ $[M+H]^+$: 179.0892, found: 179.0888.

Synthesis of (*R*,*Z*)-3-butylidene-5-(1-hydroxyallyl)-4-((trimethylsilyl)ethynyl)isobenzofuran-1(3*H*)-one (8)

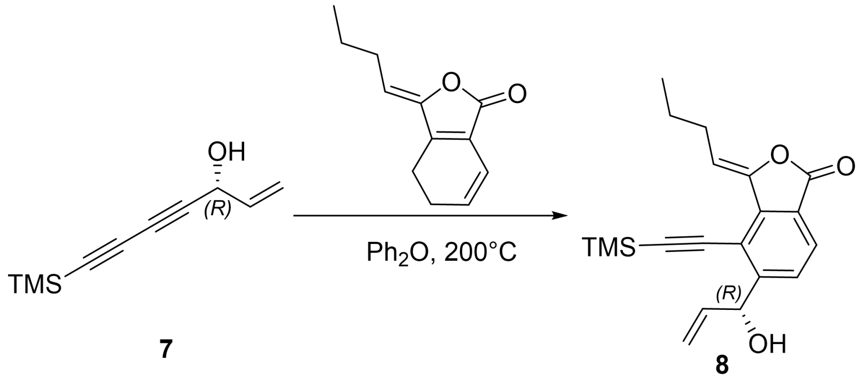

7 8

To a solution (*Z*)-3-butylidene-4,5-dihydroisobenzofuran-1(3*H*)-one (**4**, 2.40 g, 95 % purity, 12.0 mmol, reseparated from *Angelica sinensis* by the procedure in the **Extraction and isolation**) in diphenyl ether (Ph_2O, 40 mL) was added (*R*)-7-(trimethylsilanyl)hept-1-en-4,6-diyn-3-ol (**7**, 2.80 g, 95 % purity, 14.9 mmol) at 25 °C. After stirred at 200 °C under the protection of nitrogen atmosphere for 3 hours, the reaction mixture was cooled to 25 °C and directly purified by silica gel column chromatography (PE:EtOAc = 200:1 to 10:1) to give the **8** (1.30 g, 90 % purity from ^{1}HNMR, 40 % yield) as yellow oil.

R_f = 0.4 (PE:EtOAc = 5:1);

$[\alpha]_D^{27}$ = +54.5 (*c* = 1.0 in $CHCl_3$);

^{1}H NMR (400 MHz, $CDCl_3$) δ 7.87 (d, *J* = 8.0 Hz, 1H), 7.71 (d, *J* = 8.0 Hz, 1H), 6.58 (t, *J* = 7.7 Hz, 1H), 6.01 (ddd, *J* = 17.1, 10.1, 5.9 Hz, 1H), 5.83 (br d, *J* = 5.5 Hz, 1H), 5.44 (br d, *J* = 17.1 Hz, 1H), 5.23 (br d, *J* = 10.3 Hz, 1H), 2.50 (m, 2H), 1.57 (m, 2H) , 1.01 (t, *J* = 7.3 Hz, 3H), 0.33 (s, 9H);

^{13}C NMR (101 MHz, $CDCl_3$) δ 166.2, 152.1, 145.2, 138.8, 138.1, 126.6, 125.5, 124.1, 116.1, 114.4, 113.7, 108.9, 98.9, 72.6, 28.2, 22.6, 13.9, 1.0;

IR (KBr): ν_{max} = 3421, 2960, 2932, 2871, 1781, 1461, 1423, 1252, 1014, 849 cm^{-1};
HRMS (ESI): *m*/*z* calcd. for $C_{20}H_{25}O_3Si$ $[M+H]^+$: 341.1573, found: 341.1572.

Synthesis of (*R*,*Z*)-3-butylidene-4-ethynyl-5-(1-hydroxyallyl)isobenzofuran-1(3*H*)-one (S1)

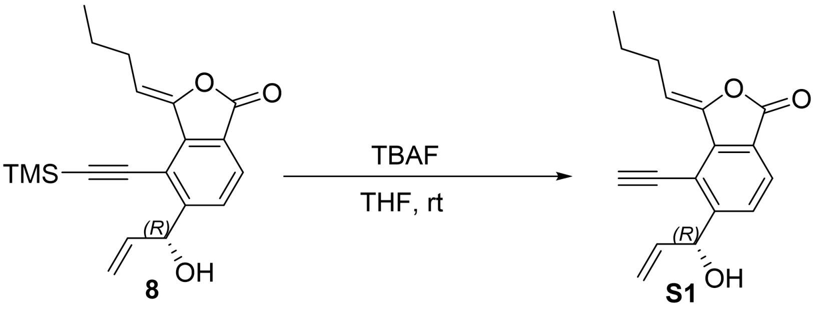

To a solution of (*R*,*Z*)-3-butylidene-5-(1-hydroxyallyl)-4-((trimethylsilyl)ethynyl)isobenzofuran-1(3*H*)-one (**8,** 17.80 g, 52.3 mmol) in tetrahydrofuran (THF, 178 mL) was added tetrabutylazanium fluoride (TBAF, 1.0 mol in THF, 52.3 mL, 52.3 mmol), then the reaction was stirred at 25 °C for 3 hours. The reaction was poured into water (300 mL) and extracted the EtOAc (300 mL) twice. The combined organic phases were washed with brine, dried over anhydrous Na_2SO_4 and concentrated under reduced pressure to give a residue, which was purified by silica gel column chromatography (PE: EtOAc = 6: 1) to obtain the **S1** (10.50 g, 92 % purity, 69 % yield) as yellow oil.

R_f = 0.3 (PE:EtOAc = 3:1);

$[\alpha]_D^{27}$ = +121.8 (*c* = 1.0 in $CHCl_3$);

^{1}H NMR (600 MHz, $CDCl_3$) δ 7.88 (d, *J* = 8.0 Hz, 1H), 7.73 (d, *J* = 8.0 Hz, 1H), 6.55 (t, *J* = 7.9 Hz, 1H), 6.01 (ddd, *J* = 17.1, 10.4, 5.5 Hz, 1H), 5.85 (br d, *J* = 5.6 Hz, 1H), 5.43 (br d, *J* = 17.1 Hz, 1H), 5.22 (br d, *J* = 10.4 Hz, 1H), 3.84 (s, 1H), 2.48 (m, 2H), 1.57 (m, 2H) , 1.00 (t, *J* = 7.4 Hz, 3H);

^{13}C NMR (151 MHz, $CDCl_3$) δ 166.1, 152.6, 145.1, 139.2, 138.1, 126.9, 125.9, 124.3, 116.1, 114.6, 112.6, 90.0, 77.9, 72.1, 28.2, 22.5, 13.8;

IR (KBr): ν_{max} =3254, 2959, 2930, 2871, 1771, 1461, 1426, 1218, 1092, 1006, 921, 728 cm^{-1};
HRMS (ESI): *m*/*z* calcd. for $C_{17}H_{17}O_3$ $[M+H]^+$: 268.1178, found: 269.1189.

Synthesis of (*R*,*Z*)-5-(1-((*tert*-butyldimethylsilyl)oxy)allyl)-3-butylidene-4-ethynylisobenzofuran-1(3*H*)-one (9)

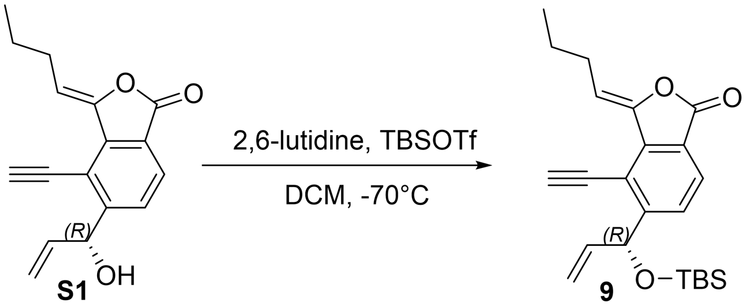

To a solution of (*R*,*Z*)-3-butylidene-4-ethynyl-5-(1-hydroxyallyl)isobenzofuran-1(3*H*)-one (**S1**, 10.50 g, 39.1 mmol) in dichloromethane (DCM, 200 mL) was added 2,6-lutidine (8.38 g, 78.2 mmol) and *tert*-butyldimethyl (trifluoromethane)sulfonylsilane (TBSOTf, 14.60 g, 58.7 mmol) at -70 °C, then the reaction was stirred at -70 °C for 3 hours. The reaction was poured into ammonium chloride aqueous solution (300 mL) and extracted with ethyl ether (Et_2O, 500 mL) twice. The combined organic phases were washed with brine, dried over anhydrous Na_2SO_4 and concentrated under reduced pressure to give a residue, which was purified by silica gel column chromatography (PE:EtOAc = 80:1) to obtain **9** (12.50 g, 95 % purity, 79 % yield) as yellow oil.

R_f = 0.6 (PE:EtOAc = 30:1);

$[\alpha]_D^{27}$ = +101.0 (*c* = 1.0 in $CHCl_3$);

^{1}H NMR (400 MHz, $CDCl_3$) δ 7.88 (d, *J* = 8.0 Hz, 1H), 7.74 (d, *J* = 8.0 Hz, 1H), 6.56 (t, *J* = 7.9 Hz, 1H), 5.92 (ddd, *J* = 17.0, 10.3, 4.8 Hz, 1H), 5.82 (br d, *J* = 4.6 Hz, 1H), 5.40 (br d, *J* = 17.0 Hz, 1H), 5.09 (br d, *J* = 10.2 Hz, 1H), 3.84 (s, 1H), 2.49 (m, 2H), 1.57 (m, 2H) , 1.00 (t, *J* = 7.26 Hz, 3H), 0.90 (s, 9H), 0.09 (s, 3H), 0.02 (s, 3H);

^{13}C NMR (101 MHz, $CDCl_3$) δ 166.1, 154.2, 145.2, 139.1, 139.0, 127.3, 125.8, 124.0, 114.2, 114.2, 111.8, 89.6, 78.1, 72.5, 28.2, 25.8, 22.5, 18.2, 13.8, -4.9, -5.0;

IR (KBr): ν_{max} =3290, 2956, 2929, 2857, 1787, 1463, 1255, 1137, 1018, 778 cm^{-1};

HRMS (ESI): *m*/*z* calcd. for $C_{23}H_{31}O_3Si$ $[M+H]^+$: 383.2042, found: 383.2044.

Synthesis of (*Z*)-5-((*R*)-1-((*tert*-butyldimethylsilyl)oxy)allyl)-3-butylidene-4-((*S*,*Z*)-3-hydroxy-5-iodopent-4-en-1-yn-1-yl)isobenzofuran-1(3*H*)-one (10)

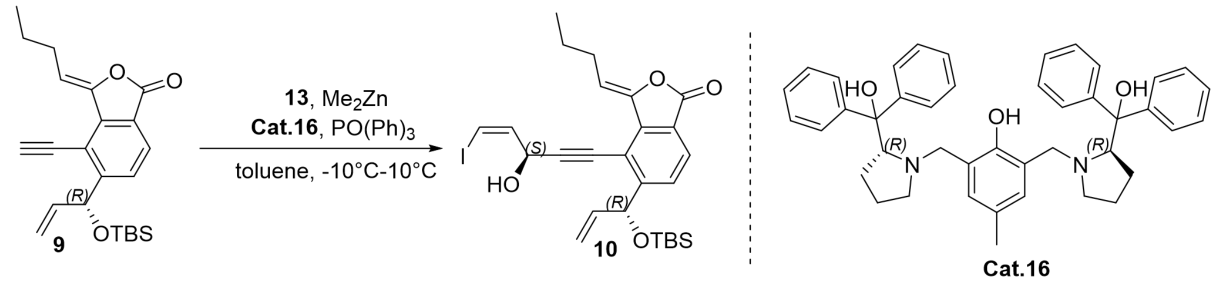

To a solution of triphenylphosphine oxide (PO(Ph)$_3$, 1.18 g, 4.2 mmol), 2,6-bis({[(2*R*)-2-(hydroxydiphenylmethyl)pyrrolidin-1-yl]methyl})-4-methylpheno[16] (**Cat. 16**, 1.60 g, 2.6 mmol) and (*R*,*Z*)-5-(1-((*tert*-butyldimethylsilyl)oxy)allyl)-3-butylidene-4-ethynylisobenzofuran-1(3*H*)-one (**9**, 6.00 g, 15.7 mmol) in toluene (150 mL) was added dimethylzinc (Me$_2$Zn, 1.0 mol, 24 mL, 24.0 mmol) at 25 ºC, then the reaction was stirred at 25 ºC for half an hour, the reaction was cooled to -10 ºC and (*Z*)-3-iodoacrylaldehyde[17] (**13**, 3.43 g, 18.8 mmol) was added. After stirred at -10 to 10 ºC for 24 hours, the reaction was poured into ammonium chloride aqueous solution (100 mL), then extracted the EtOAc (200 mL) twice. The combined organic phases were washed with brine, dried over anhydrous Na$_2$SO$_4$ and concentrated under reduced pressure to give a residue, which was purified by silica gel column chromatography (PE:EtOAc = 10:1) to obtain the **10** (7.00 g, 95 % purity, 69 % yield, dr = 12.5:1 (**Figure S62**)) as yellow oil.

R$_f$ = 0.3 (PE:EtOAc = 3:1);

$[\alpha]_D^{27}$= +205.4 (*c* = 1.0 in CHCl$_3$);

^{1}H NMR (400 MHz, CDCl$_3$) δ 7.86 (d, *J* = 8.1 Hz, 1H), 7.72 (d, *J* = 8.1 Hz, 1H), 6.63 (br d, *J* = 7.6 Hz, 1H), 6.56 (t, *J* = 7.5 Hz, 1H), 6.48(t, *J* = 7.8 Hz, 1H), 5.89 (ddd, *J* = 17.0, 10.3, 5.2 Hz, 1H), 5.76 (br d, *J* = 5.1 Hz, 1H), 5.53 (br d, *J* = 7.4 Hz, 1H), 5.40 (br d, *J* = 17.0 Hz, 1H), 5.10 (br d, *J* = 10.2 Hz, 1H), 2.48 (m, 2H), 1.57 (m, 2H) , 1.00 (t, *J* = 7.3 Hz, 3H), 0.90 (s, 9H), 0.09 (s, 3H), 0.01(s, 3H);

^{13}C NMR (101 MHz, CDCl$_3$) δ 166.2, 153.5, 145.2, 139.3, 139.1, 138.7, 127.3, 125.6, 124.0, 114.4, 114.3, 111.9, 98.8, 84.6, 80.1, 72.7, 66.4, 28.3, 25.8, 22.6, 18.3, 13.9, -4.7, -4.9;

IR (KBr):ν_{max}= 3429, 2956, 2929, 2857, 1787, 1463, 1255, 1137, 1086, 1018, 864, 848, 778 cm^{-1};

HRMS (ESI): *m*/*z* calcd. for $C_{26}H_{34}IO_4Si$ [M+H]$^+$: 565.1271, found: 565.1261.

Synthesis of (*Z*)-4-((*S*,*Z*)-3-((*tert*-butyldimethylsilyl)oxy)-5-iodopent-4-en-1-yn-1-yl)-5-((*R*)-1-((tert-butyldimethylsilyl)oxy)allyl)-3-butylideneisobenzofuran-1(3*H*)-one (S2)

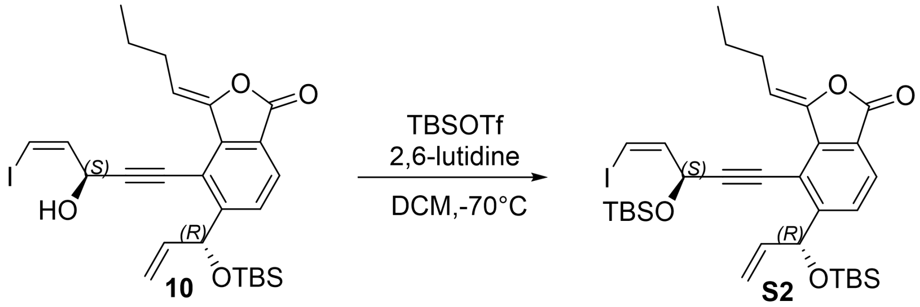

To a solution of (*Z*)-5-((*R*)-1-((tert-butyldimethylsilyl)oxy)allyl)-3-butylidene-4-((*S*,*Z*)-3-hydroxy-5-iodopent-4-en-1-yn-1-yl)isobenzofuran-1(3*H*)-one (**10**, 13.50 g, 23.9 mmol) in dichloromethane (DCM, 300 mL) was added 2,6-lutidine (5.12 g, 47.8 mmol) and *tert*-butyldimethyl(trifluoromethane)sulfonylsilane (TBSOTf, 8.90 g, 35.9 mmol) at -70 ºC, then the reaction was stirred at -70 ºC for 2 hours. The reaction was poured into the ammonium chloride aqueous solution (100 mL), then extracted the DCM (200 mL) twice. The combined organic phases were washed with brine, dried over anhydrous Na_2SO_4 and concentrated under reduced pressure to give a residue, which was purified by silica gel chromatography (PE:EtOAc = 200:1) to obtain the title **S2**(13.50 g, 95 % purity, 82 % yield) as colorless oil.

R*f* = 0.3 (PE:EtOAc = 50:1);

$[\alpha]_D^{27}$ = +246.1 (*c* = 1.0 in $CHCl_3$);

^{1}H NMR (400 MHz, $CDCl_3$) δ 7.87 (d, *J* = 8.0 Hz, 1H), 7.74 (d, *J* = 8.0 Hz, 1H), 6.47-6.53 (m, 3H), 5.93 (ddd, *J* = 17.0, 10.3, 5.1 Hz, 1H), 5.81 (br d, *J* = 5.0 Hz, 1H), 5.50 (br d, *J* = 5.4 Hz, 1H), 5.43 (br d, *J* = 16.9 Hz, 1H), 5.09 (br d, *J* = 10.2 Hz, 1H), 2.49 (m, 2H), 1.59 (m, 2H) , 1.02 (t, *J* = 7.3 Hz, 3H), 0.97 (s, 9H), 0.93 (s, 9H), 0.23 (s, 3H), 0.21 (s, 3H) , 0.12 (s, 3H), 0.10 (s, 3H);

^{13}C NMR (101 MHz, $CDCl_3$) δ 166.3, 153.5, 145.4, 140.5, 139.2, 138.6, 127.2, 125.4, 124.0, 114.1, 112.3, 99.8, 82.3, 79.0, 72.6, 67.6, 28.3, 25.8, 25.7, 22.6, 18.3, 18.1, 11.0, -4.6, -4.8, -4.9;

IR (KBr):ν_{max}= 2956, 2929, 2857, 1787, 1463, 1255, 1137, 1086, 1018, 864, 848, 778cm^{-1};

HRMS (ESI): *m*/*z* calcd. for $C_{32}H_{48}IO_4Si_2$ $[M+H]^+$: 679.2136, found: 679.2132.

Synthesis of heptyl(iodo)zinc (14)

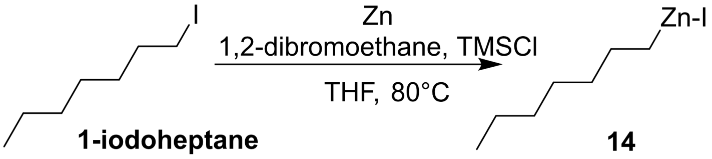

1-iodoheptane **14**

To a solution of active zinc powder (Zn, 6.95 g, 106 mmol) in tetrahydrofuran (THF, 48 mL) was added 1,2-dibromoethane (0.1 mL), and then stirred at 80 ºC under nitrogen atmosphere for 10 minutes. After cooled to 25 ºC, trimethylchlorosilane (TMSCl, 0.1 mL) was added and the reaction was stirred at 25 ºC for 5 minutes. Then 1-iodoheptane (12.00 g, 53.1 mmol) was added to the reaction for 20 minutes and stirring continued at 80 ºC for 3 hours. After cooled to 25 ºC, the solution was used for next step directly.

Synthesis of (*Z*)-5-((*R*)-1-((*tert*-butyldimethylsilyl)oxy)allyl)-4-((*S*,*Z*)-3-((tert-butyldimethylsilyl)oxy)dodec-4-en-1-yn-1-yl)-3-butylideneisobenzofuran-1(3*H*)-one (11)

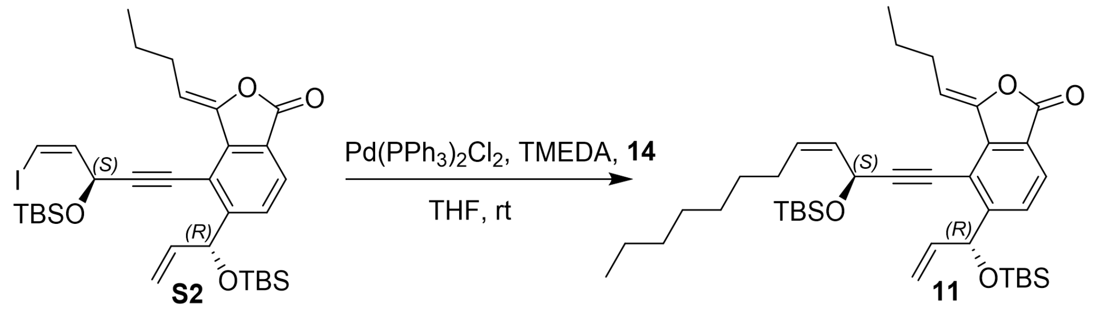

To a solution (*Z*)-4-((*S*,*Z*)-3-((*tert*-butyldimethylsilyl)oxy)-5-iodopent-4-en-1-yn-1-yl)-5-((*R*)-1-((tert-butyldimethylsilyl)oxy)allyl)-3-butylideneisobenzofuran-1(3*H*)-one (**S2**, 9.50 g, 14.0 mmol), bis(triphenylphosphine)palladium dichloride ($Pd(PPh_3)_2Cl_2$, 0.98 g, 1.40 mmol) and *N*,*N*,*N'*,*N'*-tetramethylethylenediamine (TMEDA, 3.25 g, 28.0 mmol) in tetrahydrofuran (THF, 95 mL) was added heptylzinc (II) iodide (**14**, 1.0 mol in THF) (28 mL, 28.0 mmol) at 25 ^oC under nitrogen atmosphere. After stirred at 25 ^oC under nitrogen atmosphere for 16 hours, the reaction mixture was quenched with ammonium chloride aqueous solution (100 mL), then extracted with EtOAc (200 mL) twice. The combined organic phases were washed with brine, dried over Na_2SO_4(s) and filtered. The filtrate was concentrated to give a residue, which was purified by silica gel column chromatography (PE:EtOAc = 150:1 to 140:1) to give the **11** (6.80 g, 80 % purity from ^{1}H-NMR, 60 % yield) as colorless oil.

$\mathbf{R}_f$ = 0.3 (PE:EtOAc = 50:1);

$[\alpha]_D^{27}$= +149.4 (*c* = 1.0 in $CHCl_3$);

^{1}H NMR (600 MHz, $CDCl_3$) δ 7.83 (d, *J* = 8.0 Hz, 1H), 7.71 (d, *J* = 8.0 Hz, 1H), 6.50 (t, *J* = 7.9 Hz, 1H), 5.91 (ddd, *J* = 17.0, 10.3, 5.0 Hz, 1H), 5.77 (br d, *J* = 5.0 Hz, 1H), 5.64 (m, 1H), 5.54 (m, 1H), 5.49 (br d, *J* = 8.2 Hz, 1H), 5.41 (dt, *J* = 17.0 Hz, 1.6 Hz, 1H), 5.06 (dt, *J* = 10.3 Hz, 1.6 Hz, 1H), 2.46 (m, 2H), 2.14 (m, 2H), 1.56 (m, 2H), 1.43 – 1.38 (m, 2H), 1.33–1.21 (m, 8H), 1.00 (t, *J* = 7.4 Hz, 3H), 0.93 (s, 9H), 0.90 (s, 9H), 0.85 (t, *J* = 6.9 Hz, 3H), 0.16 (s, 3H), 0.15 (s, 3H), 0.09 (s, 3H), 0.07 (s, 3H);

^{13}C NMR (151 MHz, $CDCl_3$) δ 166.4, 153.4, 145.4, 139.2, 138.7, 131.7, 129.7, 127.1, 125.1, 123.9, 114.0, 113.9, 112.7, 102.3, 78.1, 72.5, 59.8, 31.8, 29.4, 29.3, 29.1, 28.3, 27.9, 25.8, 25.7, 22.6, 18.2, 18.2, 14.0, 13.9, -4.6, -4.6, -4.9, -5.0;

IR (KBr):ν_{max}= 2956, 2929, 2857, 1787, 1463, 1255, 1137, 1086, 1018, 864, 848, 778cm^{-1};

HRMS (ESI): *m*/*z* calcd. for $C_{39}H_{63}O_4Si_2$ $[M+H]^+$: 651.4265, found: 651.4275.

Synthesis of (*Z*)-3-butylidene-5-((*R*)-1-hydroxyallyl)-4-((*S*,*Z*)-3-hydroxydodec-4-en-1-yn-1-yl)isobenzofuran-1(3*H*)-one (Compound 1)

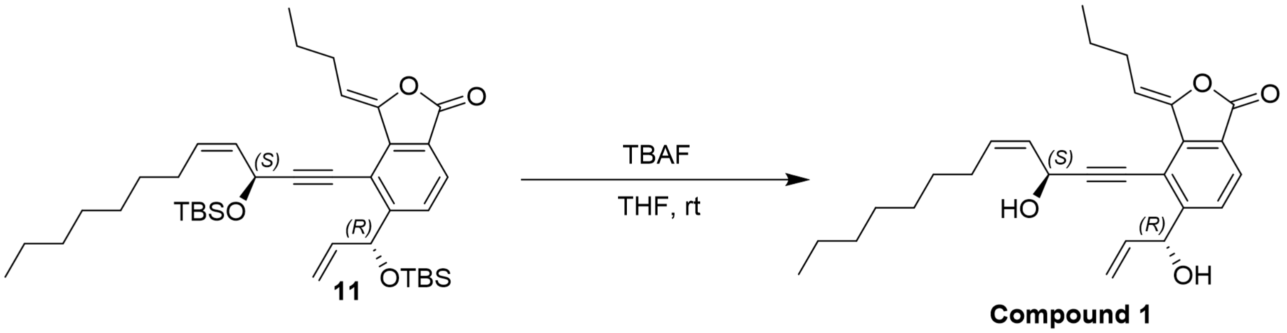

To a solution (*Z*)-5-((*R*)-1-((*tert*-butyldimethylsilyl)oxy)allyl)-4-((*S*,*Z*)-3-((*tert*-butyldimethylsilyl)oxy)dodec-4-en-1-yn-1-yl)-3-butylidene isobenzofuran-1(3*H*)-one (**11**, 6.80 g, 80 % purity, 10.4 mmol) in tetrahydrofuran (THF, 60 mL) was added tetrabutylammonium fluoride (TBAF, 1.0 M in THF, 20.8 mL, 20.8 mmol) at 25 °C under nitrogen atmosphere. After stirred at 25 °C under nitrogen atmosphere for 4 hours, the reaction mixture was poured into water (50 mL) and extracted with ethyl acetate (100 mL) twice. The combined organic phases were washed with brine, dried over Na_2SO_4 and filtered. The filtrate was concentrated to give a residue, which was purified by gel column chromatography (PE:EtOAc = 5:1 to 3:1) to give the compound (3.20 g) as colorless oil, which was separated by chiral Prep. HPLC (separation condition: Column: Chiralpak IG 5 um 30 * 250 mm; Mobile Phase: Hex:IPA = 80:20 at 30 mL/min; Temp: 30 °C; Wavelength: 254 nm) to afford the **compound 1** (2.53 g, 98.8 % purity from LCMS, 99.8 % stereopure, 57% yield) as yellow oil (**Figure S22**-**S23**).

.

R_f = 0.3 (PE: EtOAc = 1:1);

$[\alpha]_D^{27}$ = +154.1 (*c* = 1.0 in $CHCl_3$);

^{1}H NMR (600 MHz, $CDCl_3$) δ 7.86 (d, *J* = 8.0 Hz, 1H), 7.67 (d, *J* = 8.0 Hz, 1H), 6.43 (t, *J* = 7.7 Hz, 1H), 5.95 (ddd, *J* = 17.1, 10.4, 5.8 Hz, 1H), 5.70(m, 1H), 5.68 (m, 2H), 5.48 (d, *J* = 7.2 Hz, 1H), 5.42 (br d, *J* = 17.1 Hz, 1H), 5.20 (br d, *J* = 10.3 Hz, 1H), 2.47 (m, 2H), 2.18 (m, 2H), 1.56 (m, 2H), 1.42 (m, 2H), 1.31–1.22 (m, 8H), 1.00 (t, *J* = 7.4 Hz, 3H), 0.84 (t, *J* = 6.8 Hz, 3H);

^{13}C NMR (151 MHz, $CDCl_3$) δ 166.6, 151.7, 145.0, 138.9, 137.9, 134.5, 128.0, 126.4, 125.7, 124.1, 116.2, 114.8, 113.1, 102.0, 78.8, 72.3, 58.8, 31.8, 29.3, 29.2, 29.1, 28.3, 27.8, 22.6, 22.5, 14.0, 13.9;

IR (KBr): ν_{max} = 3424, 2957, 2926, 2856, 1756, 1463, 1273, 1094, 1022, 782 cm^{-1};

HRMS (ESI): *m*/*z* calcd. for $C_{27}H_{35}O_4$ $[M+H]^+$: 423.2535, found: 423.2534.

9.2 Spectra Comparison of the Natural and Synthetic NPs.

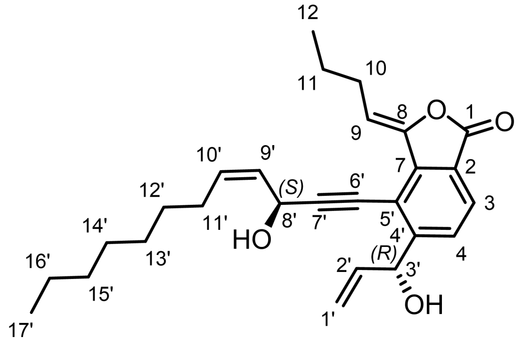

Table S13. ^{1}H NMR Comparison of Natural and Synthetic NP[a]

No.	δ_H in ppm (mult., J in Hz)		
	δ natural	δ synthetic	△δ (δ natural-δ synthetic)
3	7.85, d (8.0)	7.86, d (8.0)	-0.01
4	7.67, d (8.0)	7.67, d (8.0)	0
9	6.43, t (7.7)	6.43, t (7.7)	0
10	2.47, m	2.47, m	0
11	1.55, m	1.56, m	-0.01
12	0.99, t (7.4)	1.00, t (7.4)	-0.01
1′	5.20,dt (10.3, 1.2), Ha	5.20, br d (10.3)	0
	5.42, dt (17.0, 1.2), Hb	5.42, br d (17.1)	0
2′	5.95, ddd (17.0, 10.3, 5.6)	5.95, ddd (17.1, 10.4, 5.8)	0
3′	5.71, br d (5.6)	5.70, m	0.01
8′	5.48, d (7.2)	5.48, d (7.2)	0
9′	5.67, m	5.67, m	0
10′	5.68, m	5.69, m	-0.01
11′	2.17, m	2.18, m	-0.01
12′	1.41, m	1.42, m	-0.01
13′,14′,15′,16′	1.23-1.29	1.22-1.31	-
17′	0.84, t (6.9)	0.84, t (6.8)	0

[a] Calibrated by using residual undeuterated chloroform (δ_H = 7.26 ppm) as internal reference.

Table S14. ^{13}C NMR Comparison of Natural and Synthetic NP.[a]

No.	δ_C in ppm		
	δ natural	δ synthetic	$\triangle\delta$ (δnatural-δsynthetic)
1	166.5	166.6	-0.1
2	124.1	124.1	0.0
3	125.7	125.7	0.0
4	126.5	126.4	0.1
7	138.8	138.8	0.0
8	145.0	145.0	0.0
9	114.7	114.8	-0.1
10	28.3	28.2	0.0
11	22.5	22.5	0.0
12	13.9	13.9	0.0
1′	116.1	116.2	-0.1
2′	138.0	138.0	0
3′	72.2	72.3	-0.1
4′	151.8	151.7	0.1
5′	113.1	113.1	0.0
6′	78.8	78.8	0.0
7′	102.0	102.0	0
8′	58.8	58.8	0.0
9′	128.0	128.0	0.0
10′	134.4	134.5	-0.1
11′	27.8	27.8	0.0
12′	29.3	29.3	0.0
13′	29.1	29.1	0.0
14′	29.2	29.2	0.0
15′	31.7	31.8	-0.1
16′	22.6	22.6	0.0
17′	14.0	14.0	0.0

[a] Calibrated by using $CDCl_3$ (δ_C = 77.0 ppm) as internal reference.

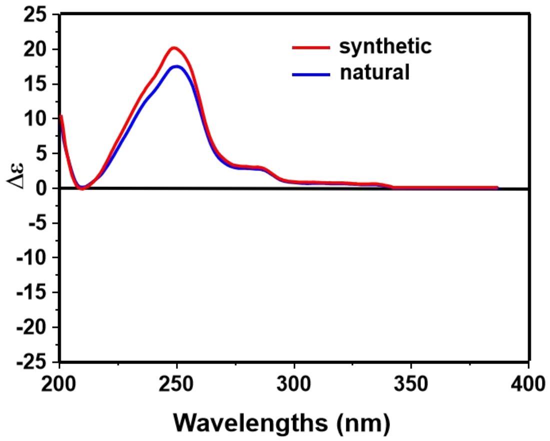

Figure S20. ECD Comparison of Natural and Synthetic NP. (Natural CD (4.7×10^{-4} M MeOH) λ_{max} ($\Delta\varepsilon$): 252(+17.41), 292(2.67) nm; Synthetic CD (4.7×10^{-4} M MeOH) λ_{max} ($\Delta\varepsilon$): 252(+20.00), 292(2.86) nm)

9.3 HPLC Chromatograms

Chiral HPLC data of 5

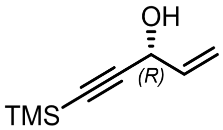

[EnantioPak Y2, MeOH:H_2O =50:50, 1.0 mL/min, λ = 208 nm]

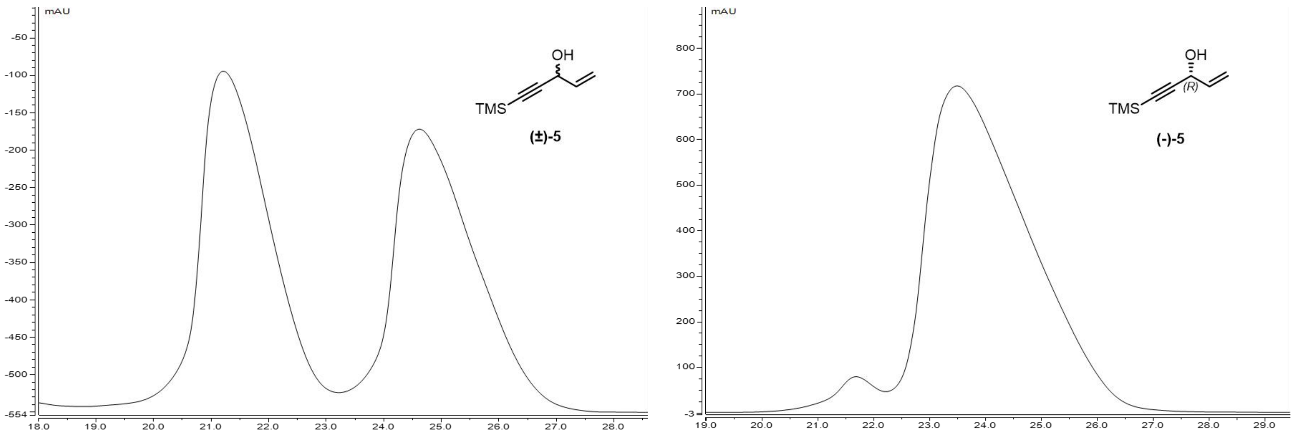

Peak	Time	Area	Area%
**1**	21.212	581.5295	50.29
**2**	24.618	574.7453	49.71

Peak	Time	Area	Area%
**1**	21.683	27.3810	1.93
**2**	23.493	1388.0510	98.07

Figure S21. HPLC chromatogram of (±)-**5** and (-)-**5.**

ee：(R-S)/(R+S)*100%=96.14%

Chiral HPLC data of 1

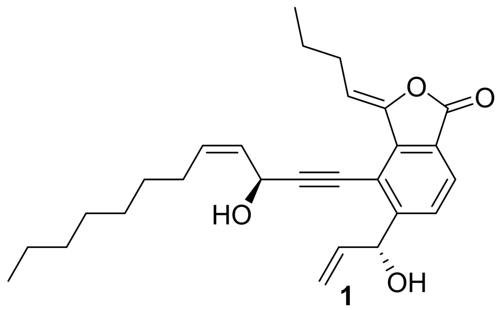

1

[Chiralpak IG, Hex : EtOH=80:20, 1 mL/min; λ = 254 nm]

<Chromatogram>

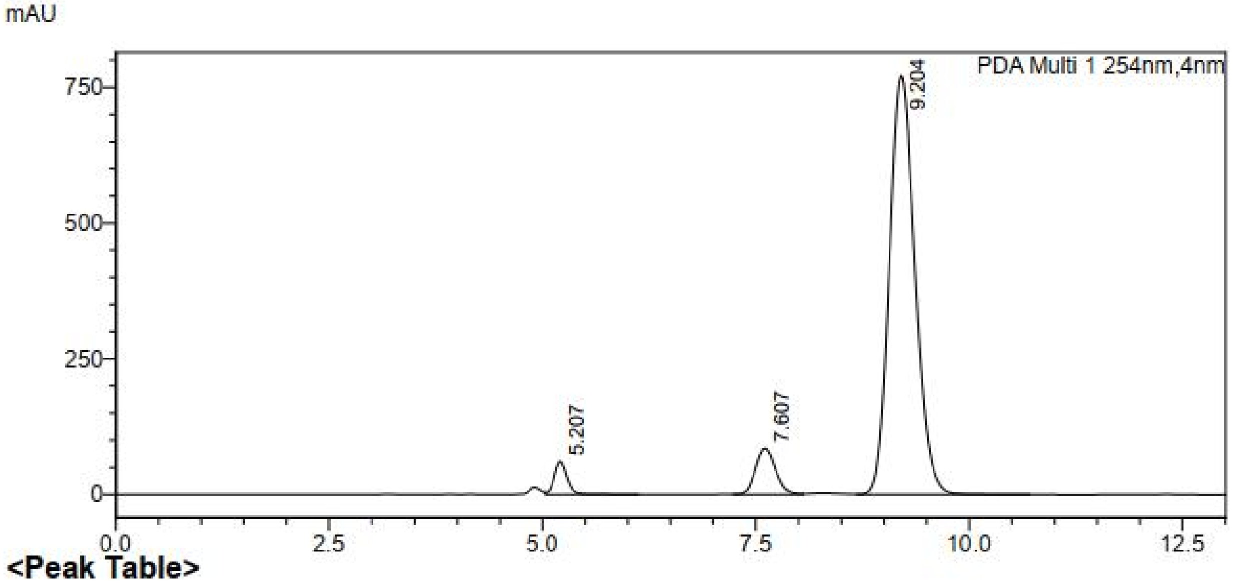

<Peak Table>

PDA Ch1 254nm

Peak#	Ret. Time	Area	Height	Conc.	Mark	Area%	Resolution(USP)
1	5.207	633261	60836	0.000	S	3.480	--
2	7.607	1384981	85058	0.000		7.611	6.714
3	9.204	16178747	771342	0.000		88.909	3.217
Total		18196988	917235			100.000	

Figure S22. HPLC chromatogram of Crude of compound **1**

<Chromatogram>

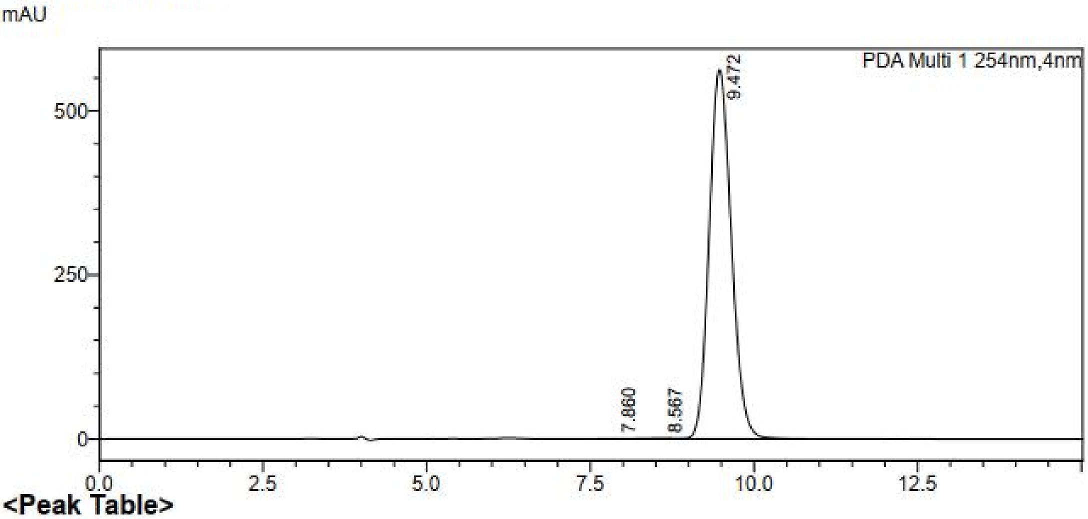

<Peak Table>

PDA Ch1 254nm

Peak#	Ret. Time	Area	Height	Conc.	Mark	Area%	Resolution(USP)
1	7.860	2525	173	0.000		0.019	--
2	8.567	24961	671	0.000	V	0.188	0.740
3	9.472	13244509	562700	0.000	V	99.793	0.866
Total		13271996	563544			100.000	

Figure S23. HPLC Chromatogram of compound **1** after purification by chiral Prep. HPLC

Supporting References

[1] S.E. Denmark, J.P. Fu and M. J. Lawler, *J. Org. Chem.* **2006**, *71*, 1523-1536;

[2] H. Zhao, G.D. Chen, J. Zou, R.R. He, S.Y. Qin, D. Hu, G.Q. Li, L.D. Guo, X.S. Yao and H. Gao, *Org. Lett.* **2017**, *19*, 38-41.

[3] Zheng, G. R.; Lu, W and Cai, J. C. *J. Nat. Prod.* **1999**, *62*, 626-628

[4] Chae, S. H.; Kim, S. I.; Yeon, S. H.; Lee, S. W. and Ahn, Y. J. *J. Agric. Food Chem.* **2011**, *59*, 8193-8198.

[5] Frisch, M. J.; Trucks, G. W.; Schlegel, H. B.; Scuseria, G. E.; Robb, M. A.; Cheeseman, J. R.; Scalmani, G.; Barone, V.; Mennucci, B.; Petersson, G. A. *et al*. Gaussian 09, Revision C1; Gaussian, Inc., Wallingford CT, **2010**.

[6] Grimblat, N.; Zanardi, M.M.; Sarotti, A.M. *J. Org. Chem.* **2013**, 25, 243-249.

[7] Bruhn, T.; Schaumlöffel, A.; Hemberger, Y; Bringmann, G; Version 1.61 ed.; University of Würzburg: Würzburg, Germany, **2013**.

[8] Zhou, C. H.; Shi, Z. L.; Meng, J. H.; Hu, B.; Zhao, C. C.; Yang, Y. T.; Yu, W.; Chen, Z. X.; Heng, B. C.; Parkman, V. J. A.; *et al. Br. J. Pharmacol.* **2018**, 175, 859-876.

[9] Qiu, Z. C.; Dong, X. L.; Dai, Y.; Xiao, G. K.; Wang, X. L.; Wong, K. C.; Wong, M. S.; Yao, X. S. *Int. J. Mol. Sci.* 2016, 17, 2116.

[10] Hariharan, P. C.; Pople, J. A. *Theor. Chim. Acta.* **1973**, 28, 213-222.

[11] Fukui, K. *J. Phys. Chem.* **1970**, 74, 4161-4163.

[12] Fukui, K. *Acc. Chem. Res.*, **1981**, 14, 363-368.

[13] Zhao, Y.; Truhlar, D. G. *Theor. Chem. Acc.* **2008**, 120, 215-241.

[14] Yun, H.; Danishefsky, S. J. *J. Org. Chem.* **2003**, 68, 4519-4522.

[15] Baldwin, J. E.; Adlington R. M.; Wilkinson P. J.; Marquez R.; Adamo M. F. A. *Heterocycles.* **2003**, 59, 81-85.

[16] Trost, B. M.; Hung, C.-I. (Joey); Mata, G. *Angew. Chem. Int. Ed.* **2020**, 59, 4240–4261.

[17] Marek, I.; Meyer, C.; Normant, J. F. *Org. Synth.***1997**,*74*, 194

10. The 1D and 2D NMR spectra of 1−2, and 1a

10.1 The 1D and 2D NMR spectra of 1 in $CDCl_3$

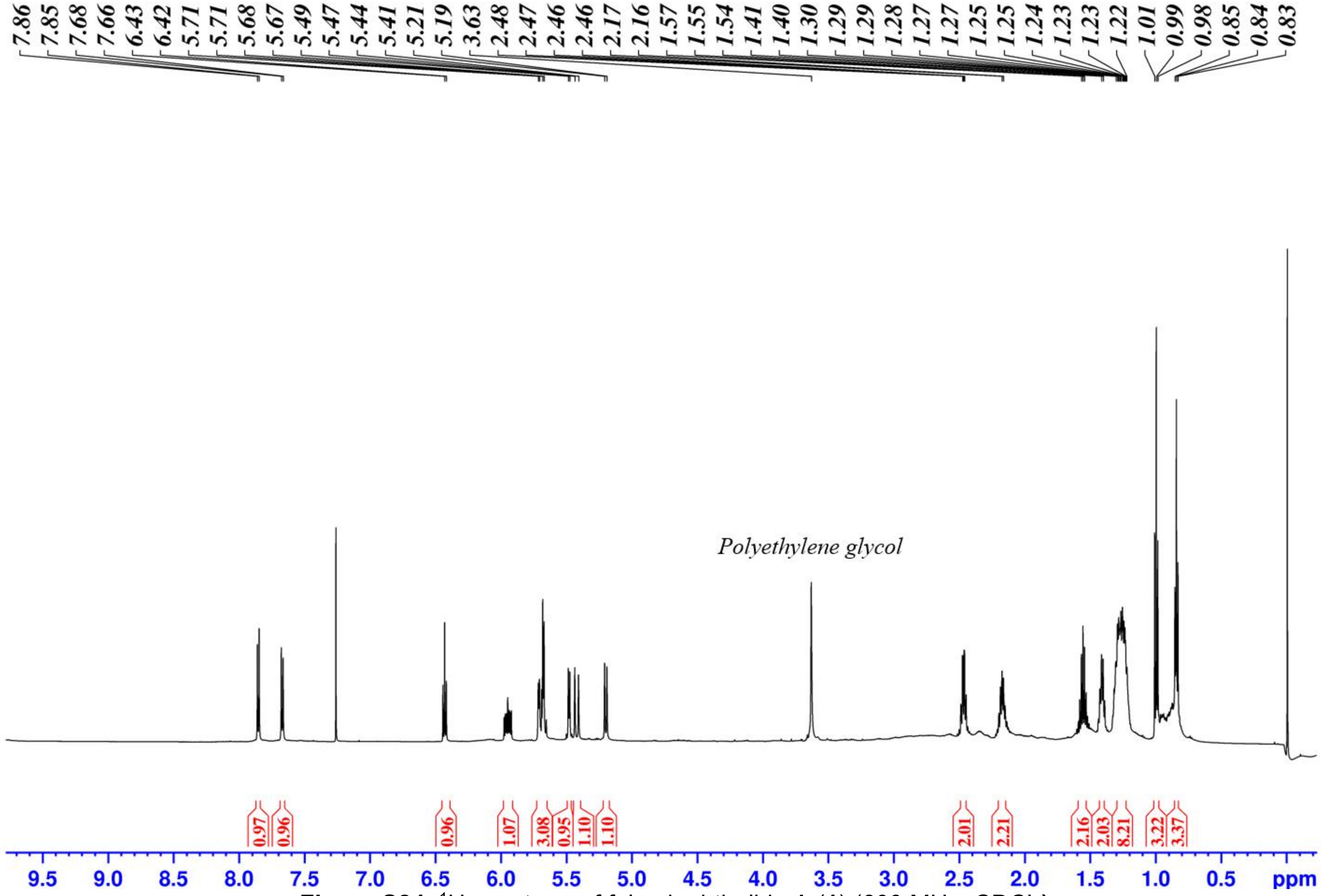

Figure S24. ^{1}H spectrum of falcarinphthalide A (**1**) (600 MHz, $CDCl_3$)

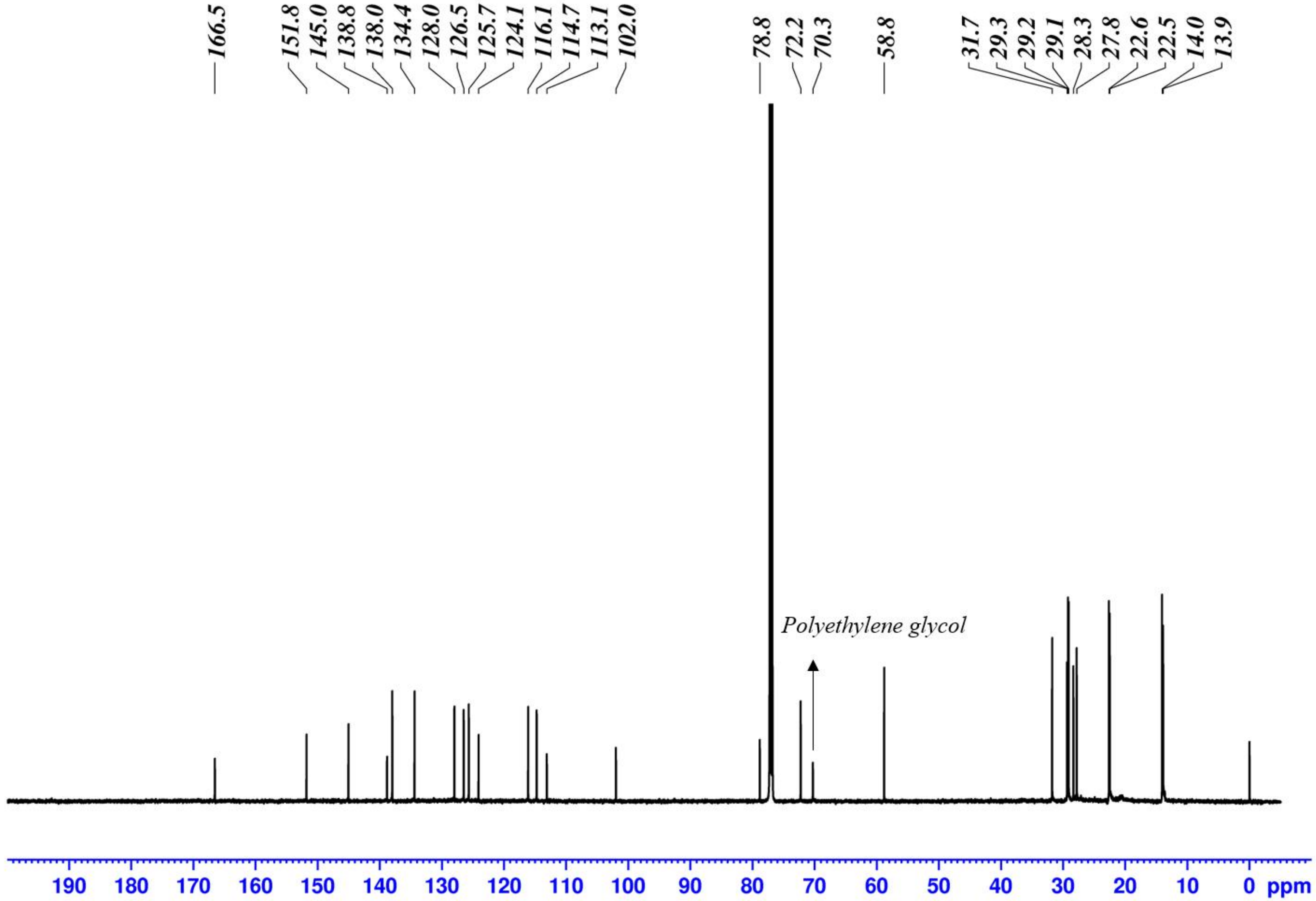

Figure S25. ^{13}C spectrum of falcarinphthalide A (**1**) (151 MHz, $CDCl_3$)

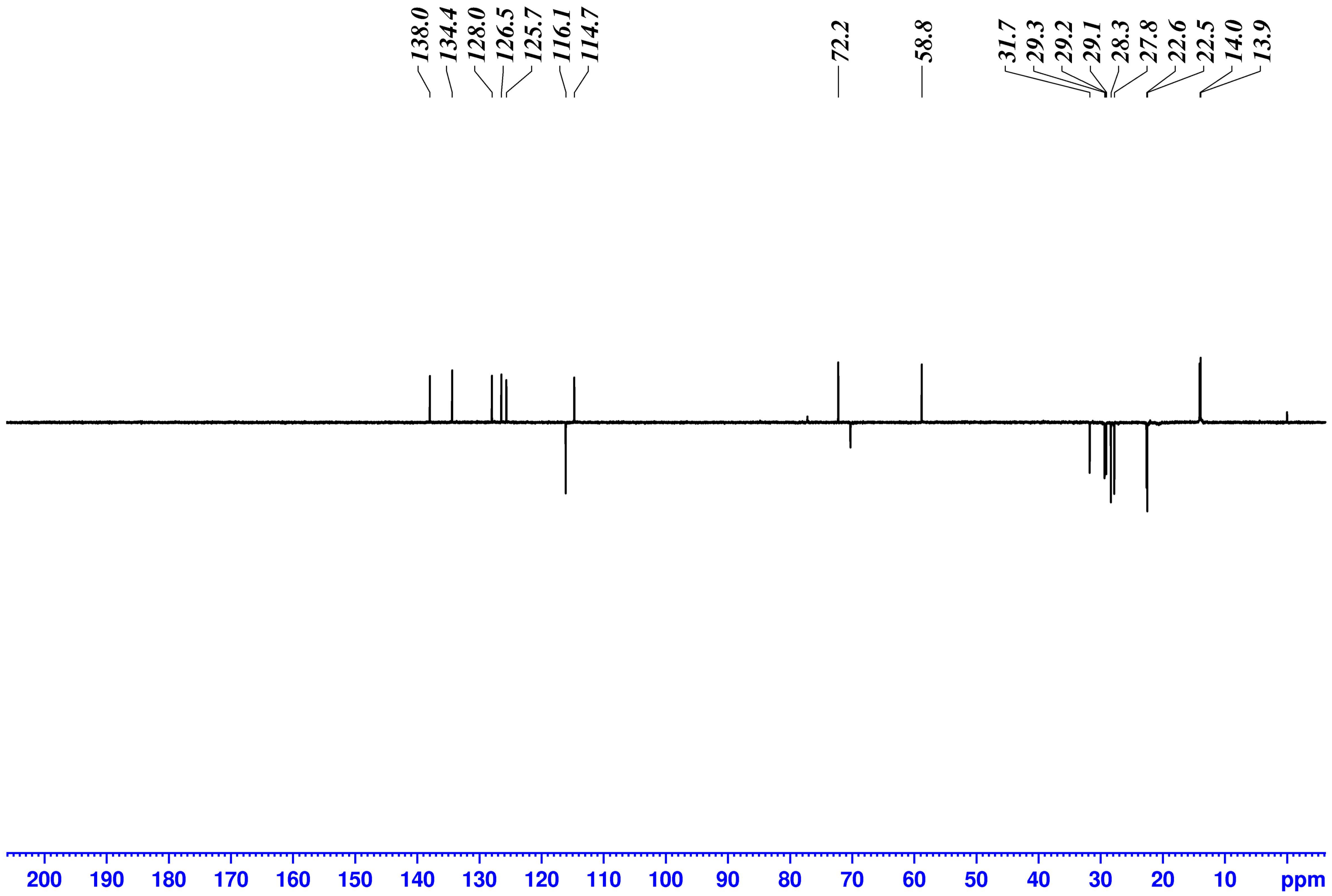

Figure S26. DEPT135 spectrum of falcarinphthalide A (**1**) (151 MHz, $CDCl_3$)

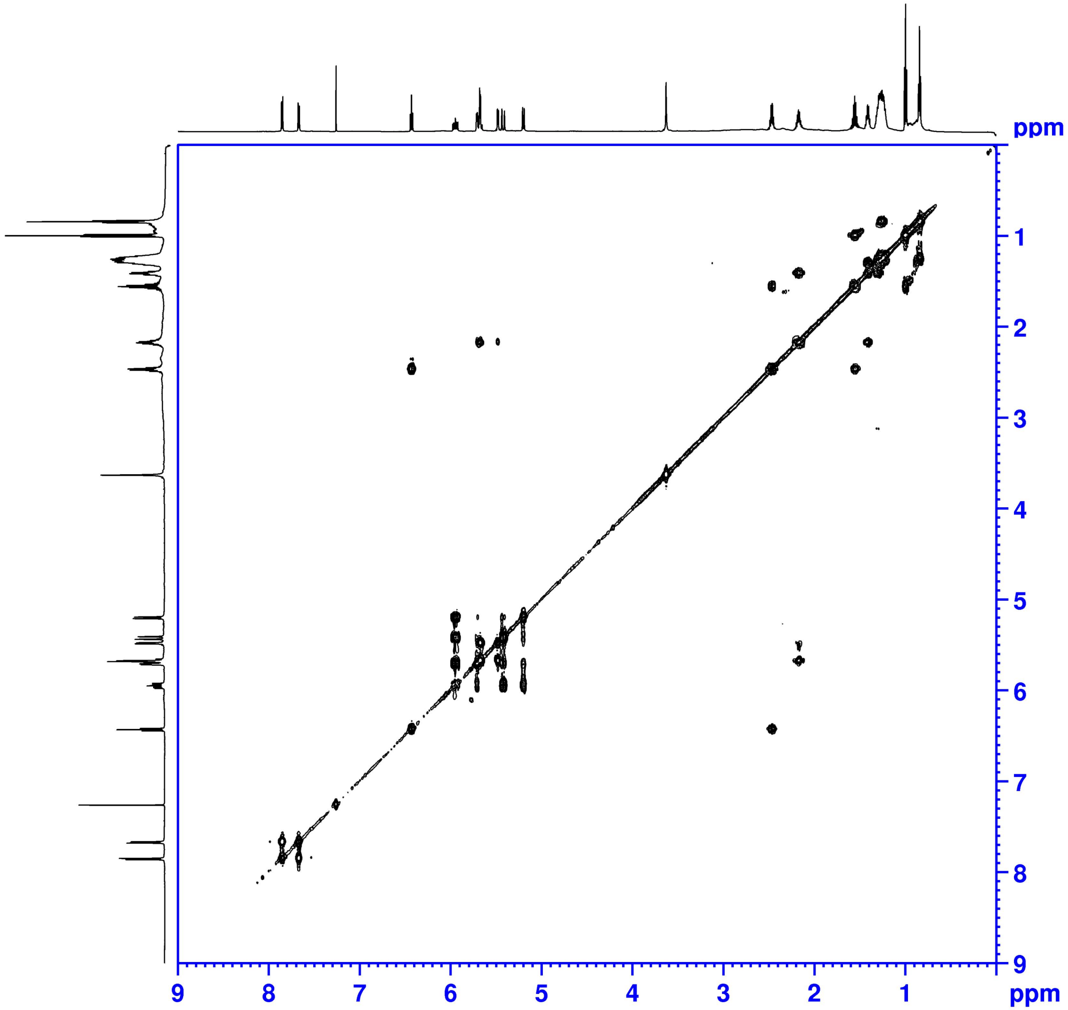

Figure S27. ^{1}H, ^{1}H-COSY spectrum of falcarinphthalide A (**1**) ($CDCl_3$)

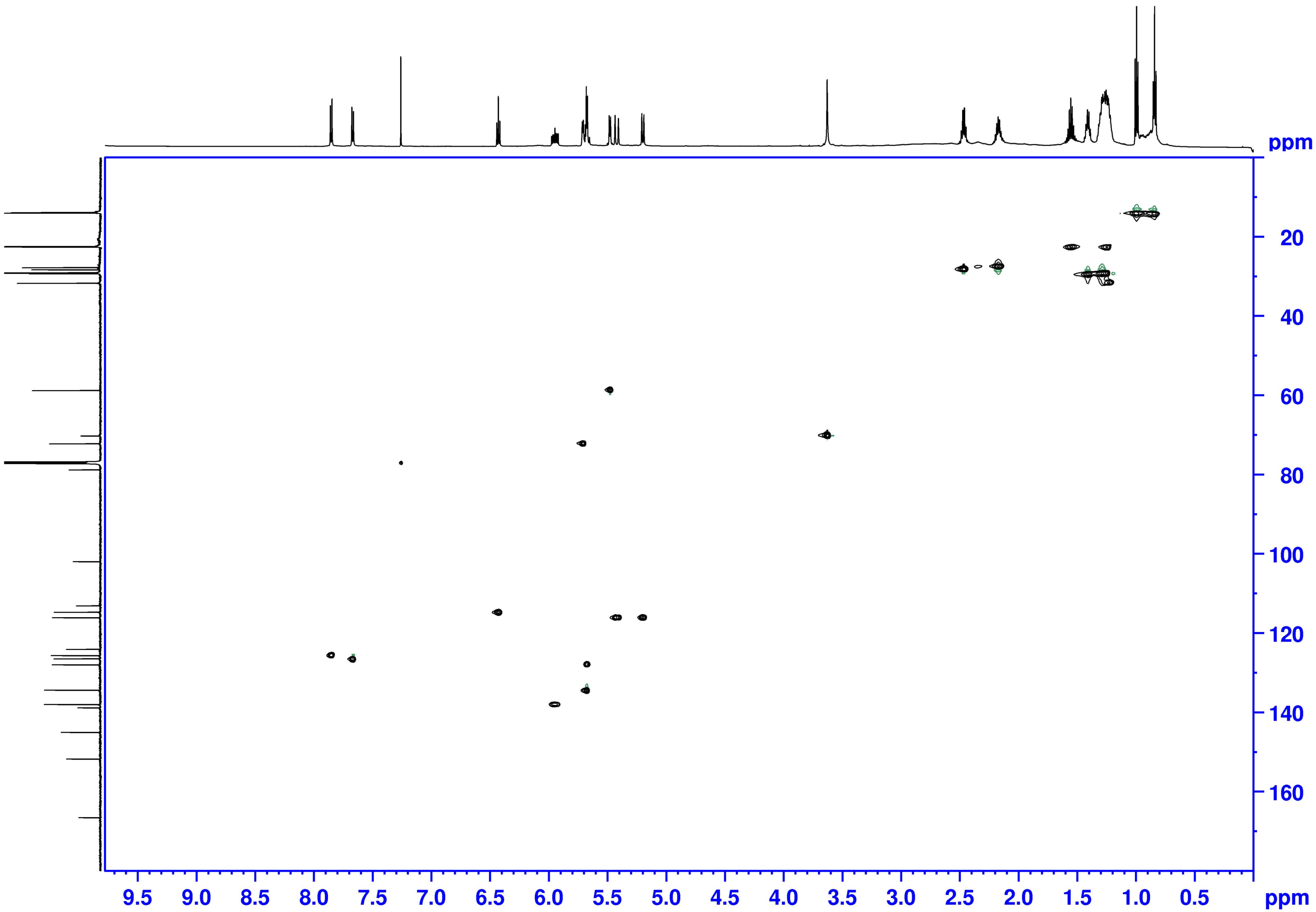

Figure S28. HSQC spectrum of falcarinphthalide A (**1**) ($CDCl_3$)

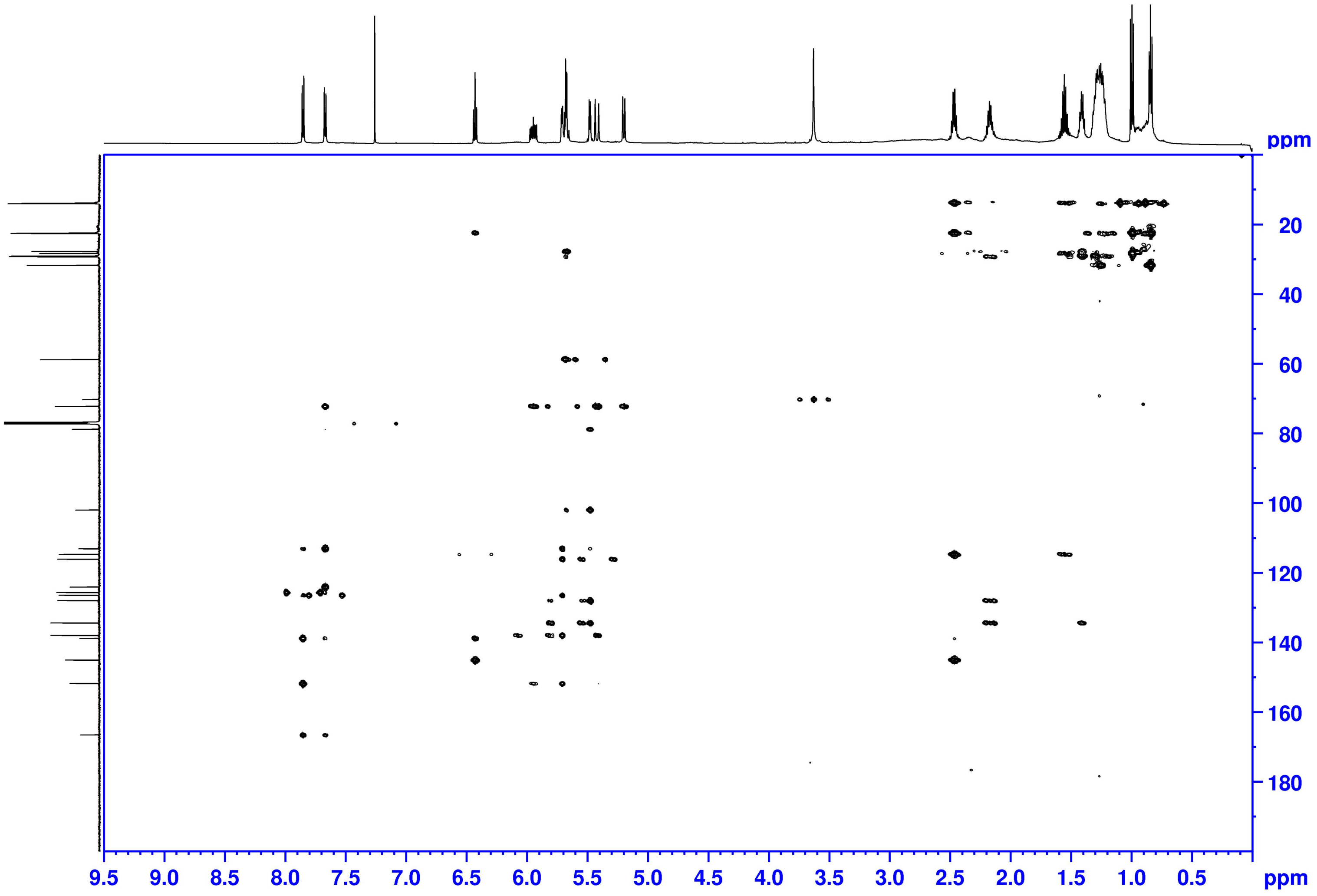

Figure S29. HMBC spectrum of falcarinphthalide A (**1**) ($CDCl_3$)

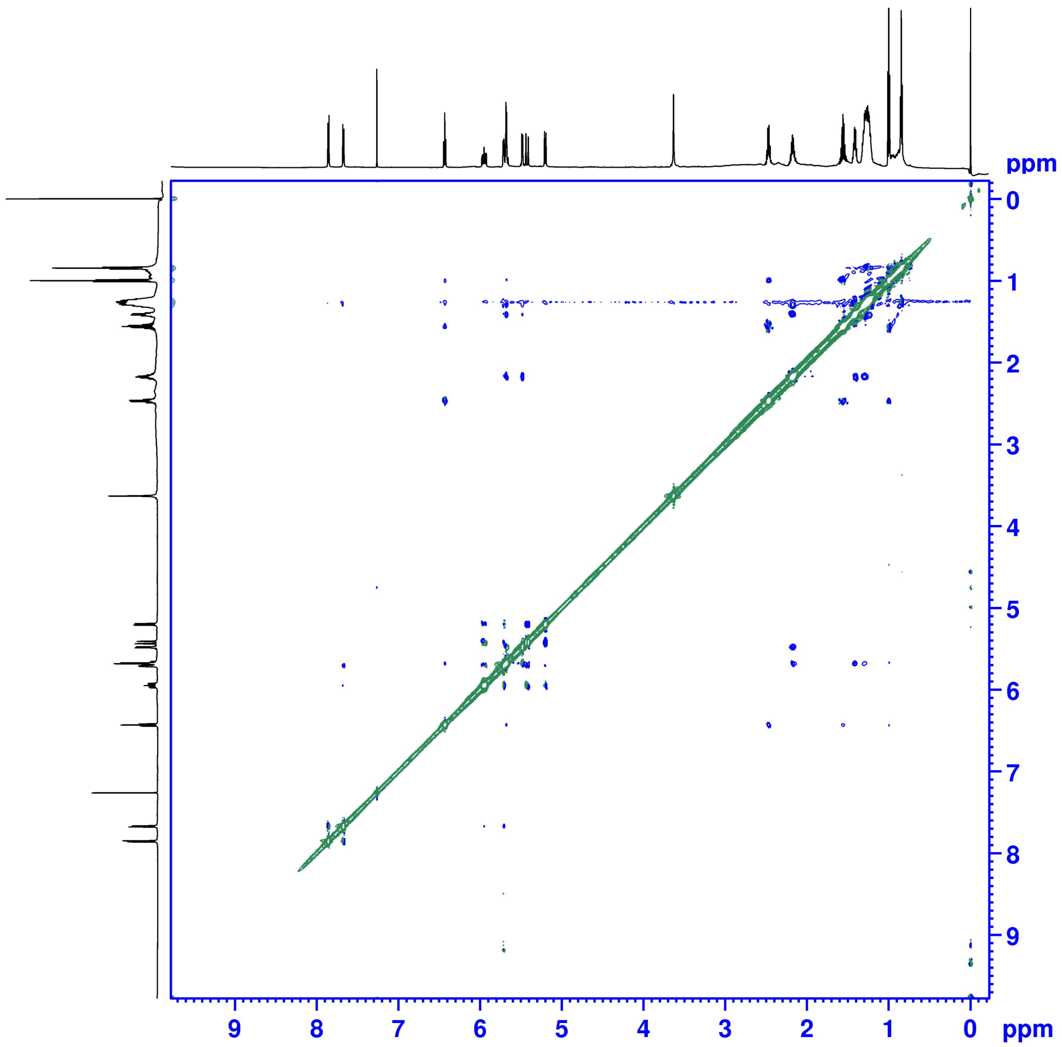

Figure S30. NOESY spectrum of falcarinphthalide A (**1**) ($CDCl_3$)

10.2 The 1D and 2D NMR spectra of 1 in C_6D_6

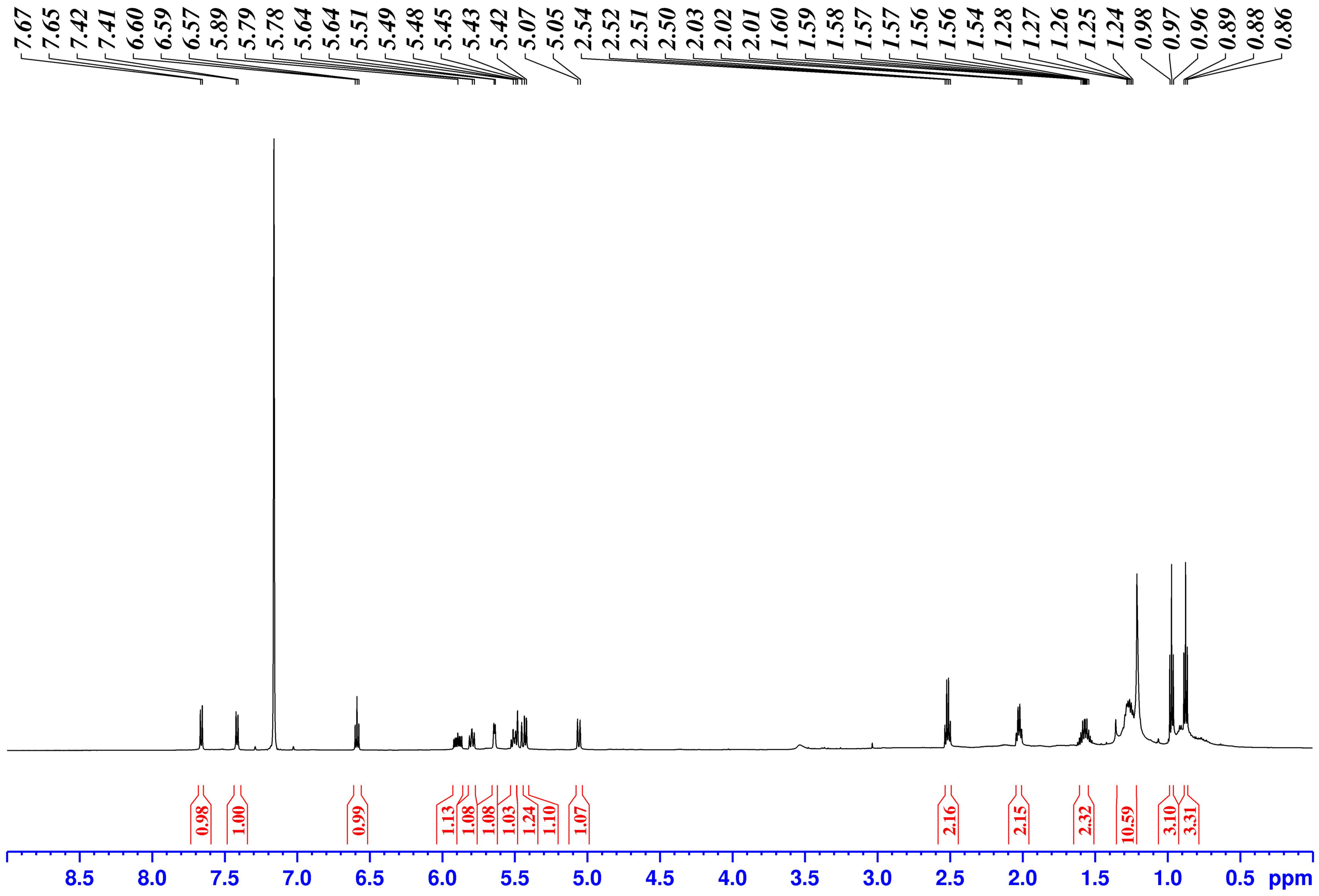

Figure S31. ^{1}H spectrum of falcarinphthalide A (**1**) (600 MHz, C_6D_6)

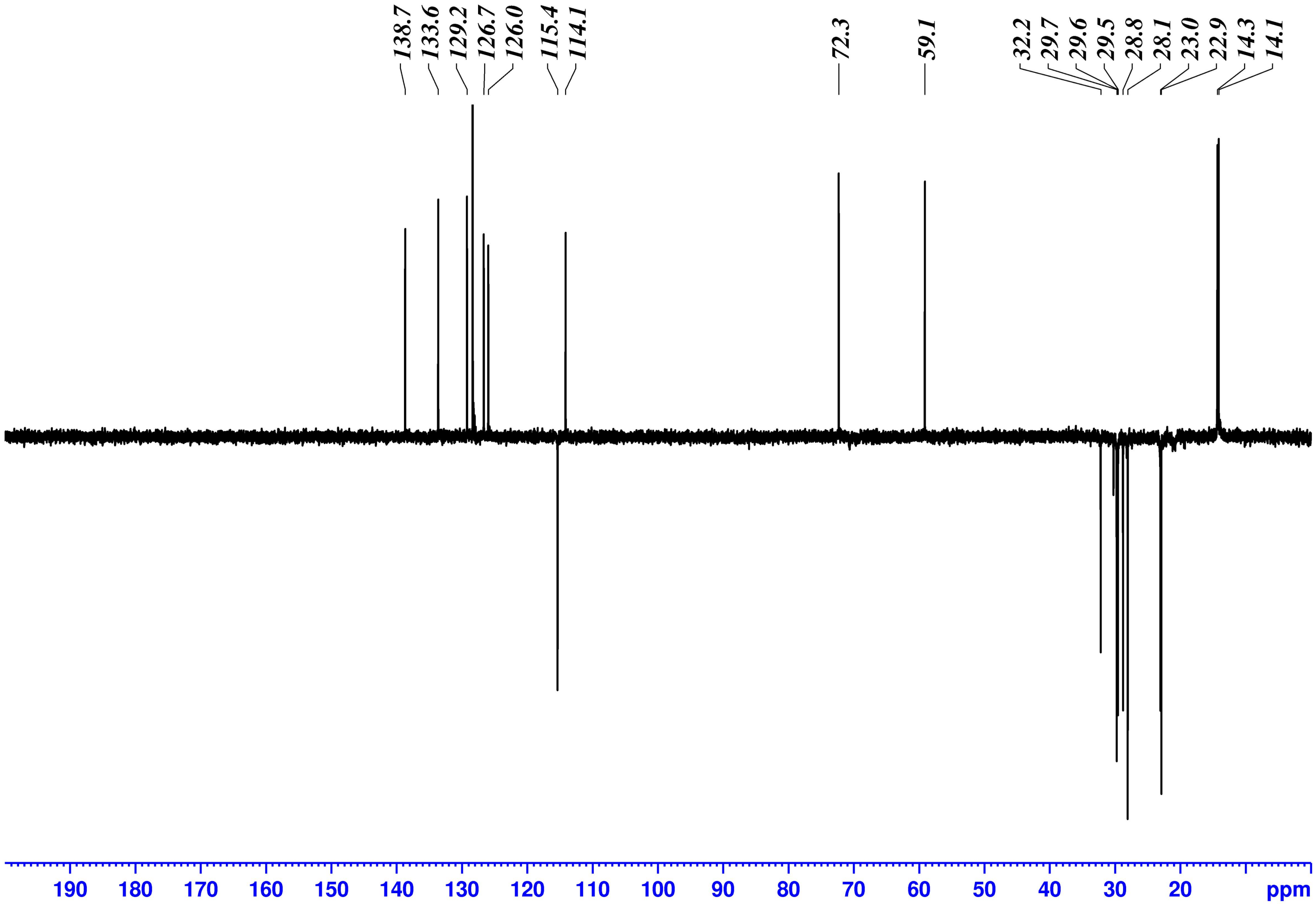

Figure S32. DEPT135 spectrum of falcarinphthalide A (**1**) (151MHz, C_6D_6)

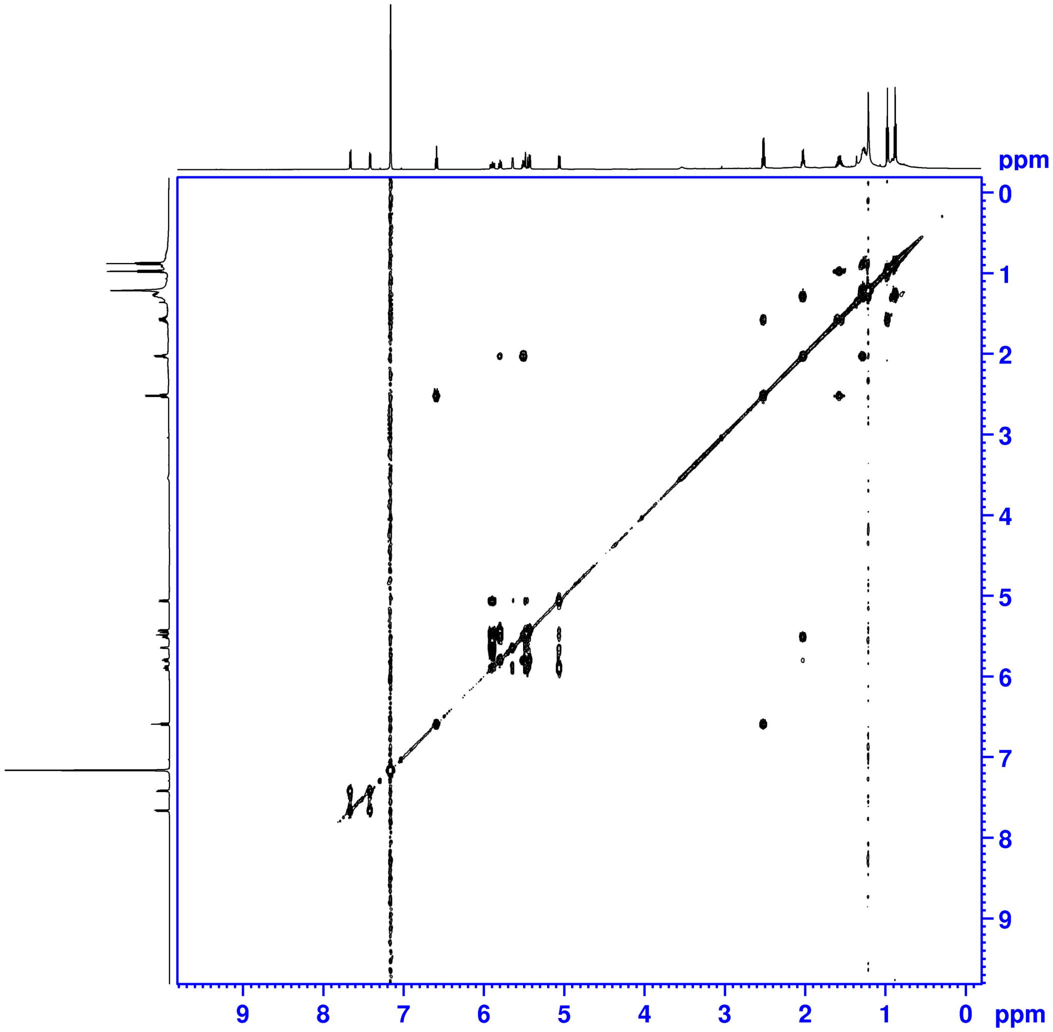

Figure S33. ^{1}H, ^{1}H-COSY spectrum of falcarinphthalide A (**1**) (C_6D_6)

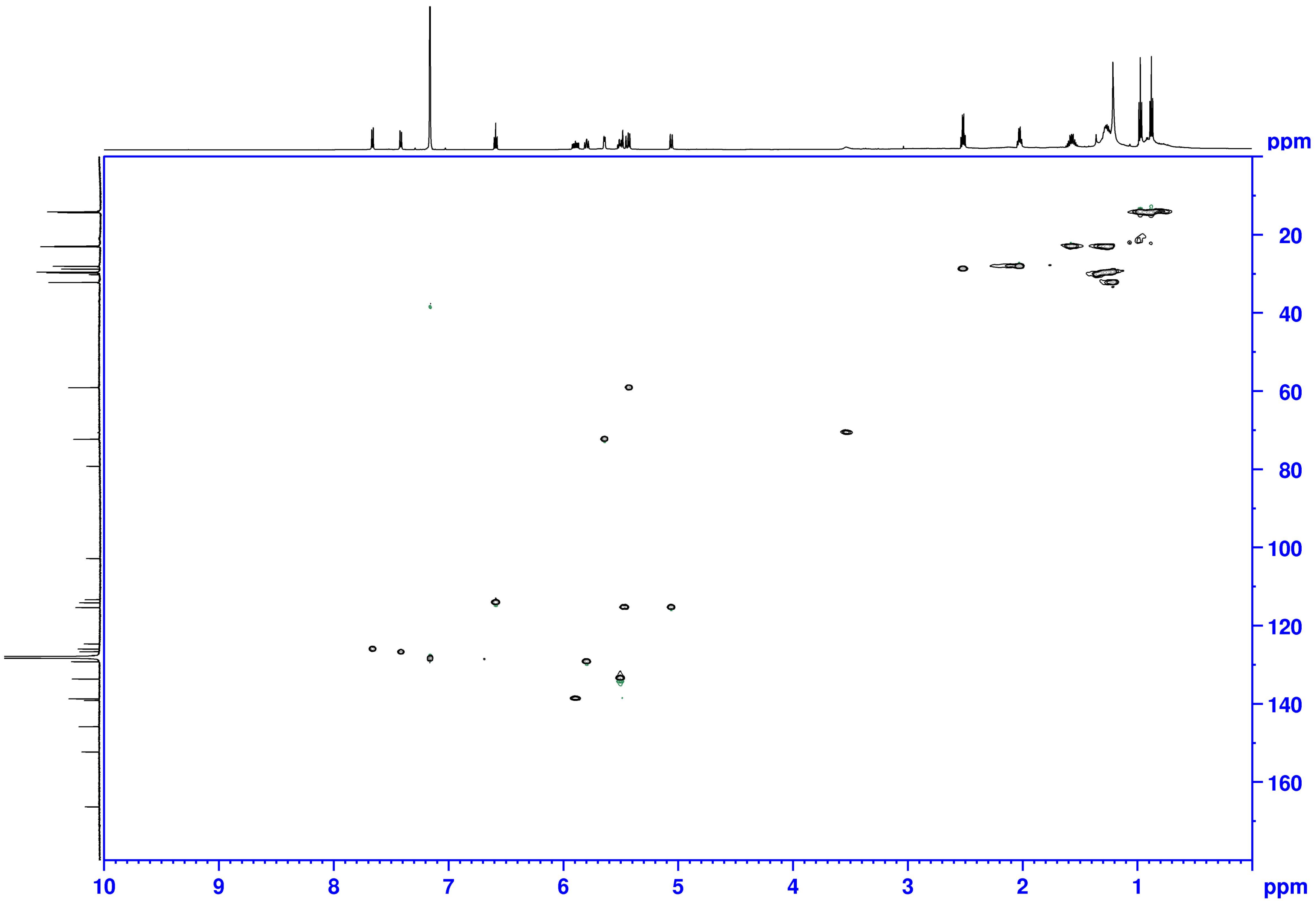

Figure S34. HSQC spectrum of falcarinphthalide A (**1**) (C_6D_6)

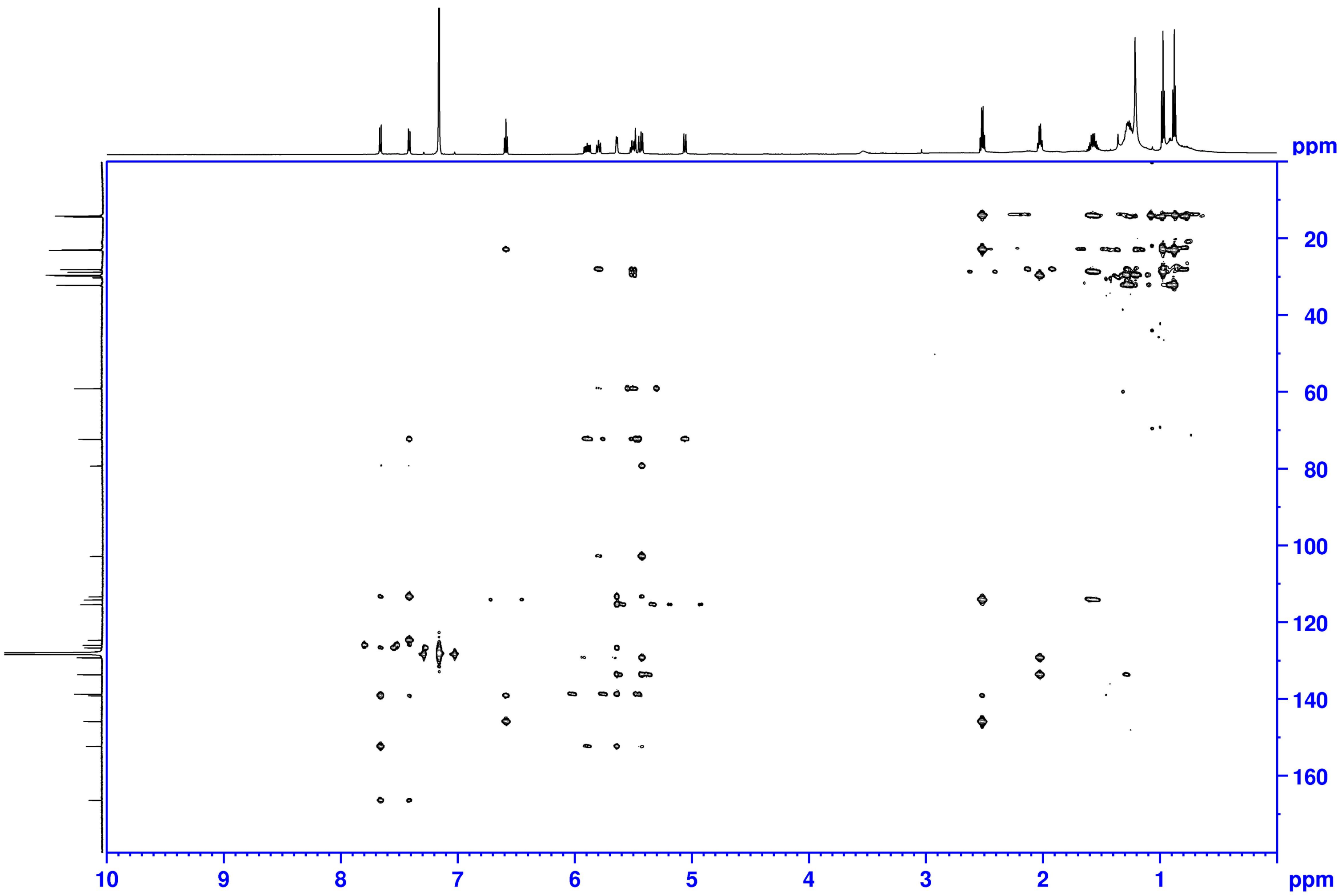

Figure S35. HMBC spectrum of falcarinphthalide A (**1**) (C_6D_6)

10.3 The 1D and 2D NMR spectra of 1a in $CDCl_3$

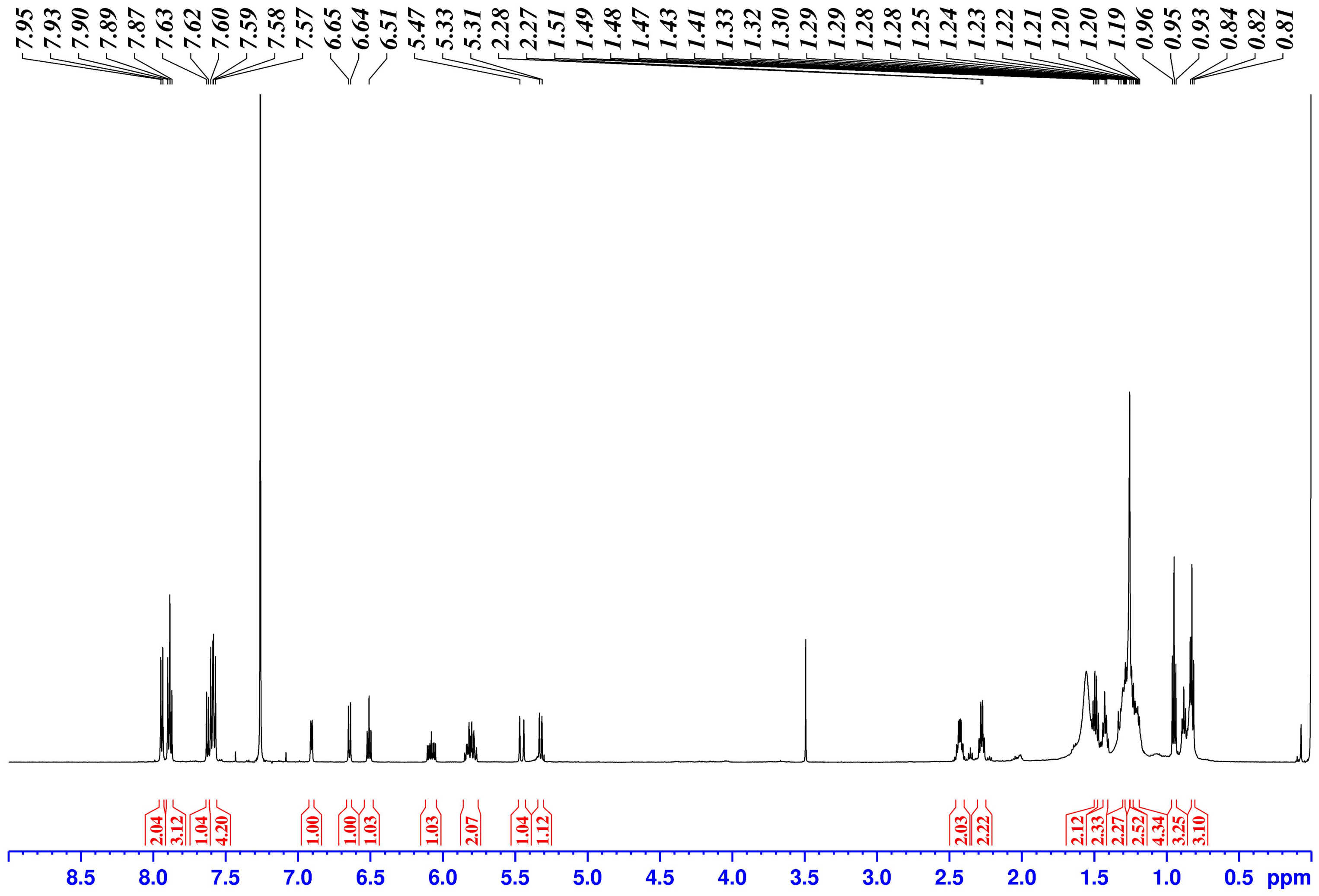

Figure S36. ^{1}H spectrum of **1a** (600 MHz,$CDCl_3$)

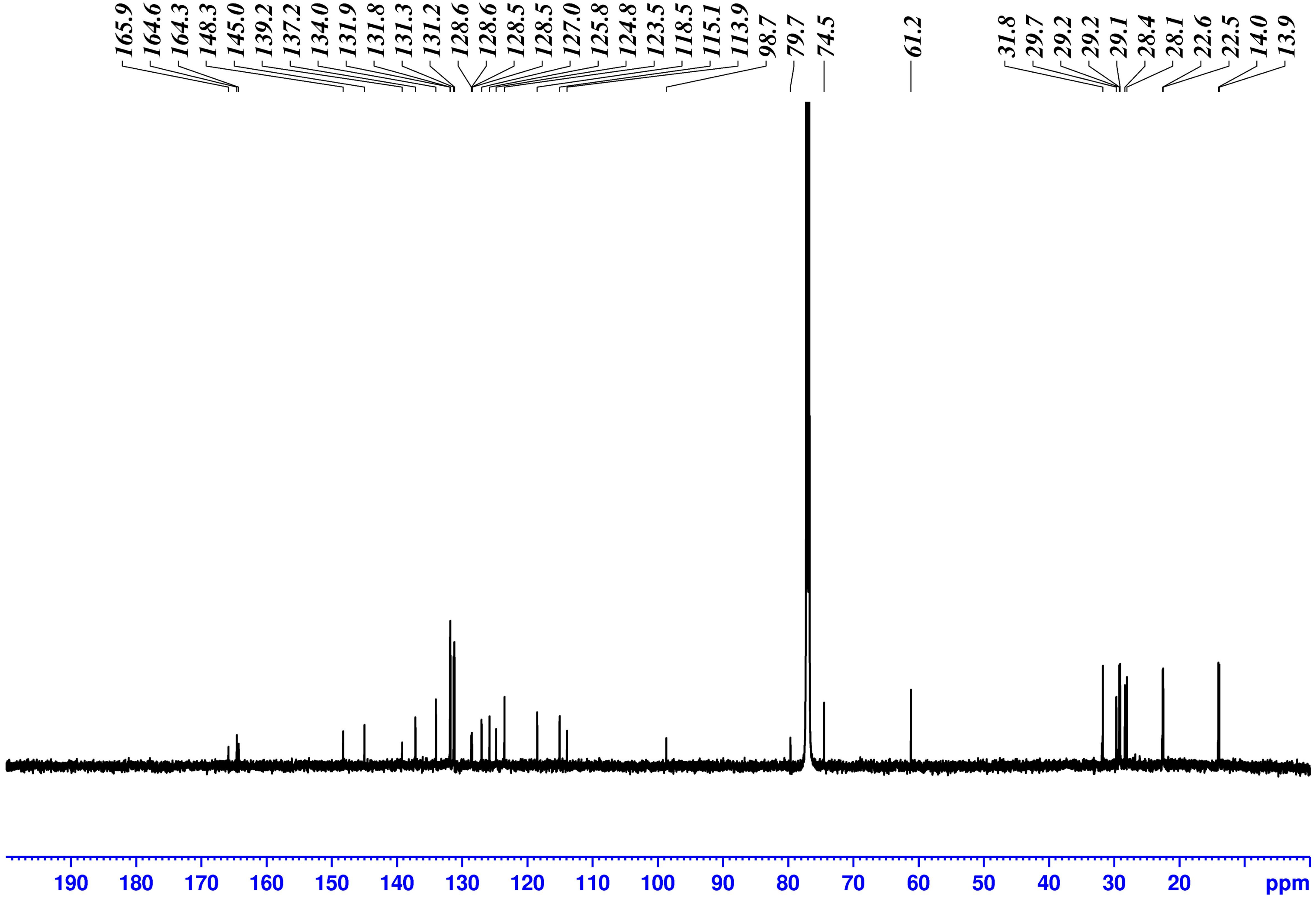

Figure S37. ^{13}C spectrum of **1a** (151 MHz,$CDCl_3$)

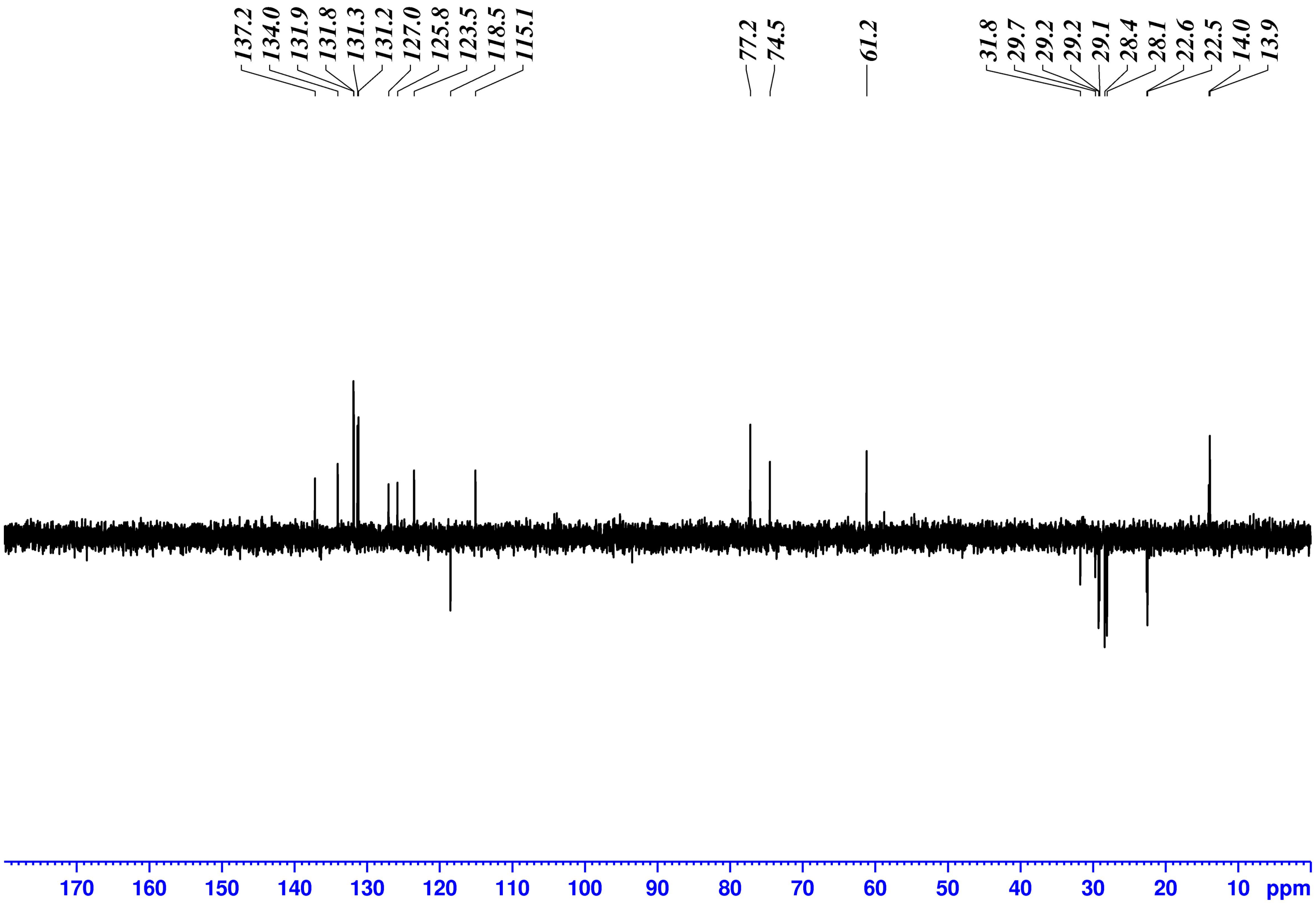

Figure S38. DEPT135 spectrum of **1a** (151 MHz,$CDCl_3$)

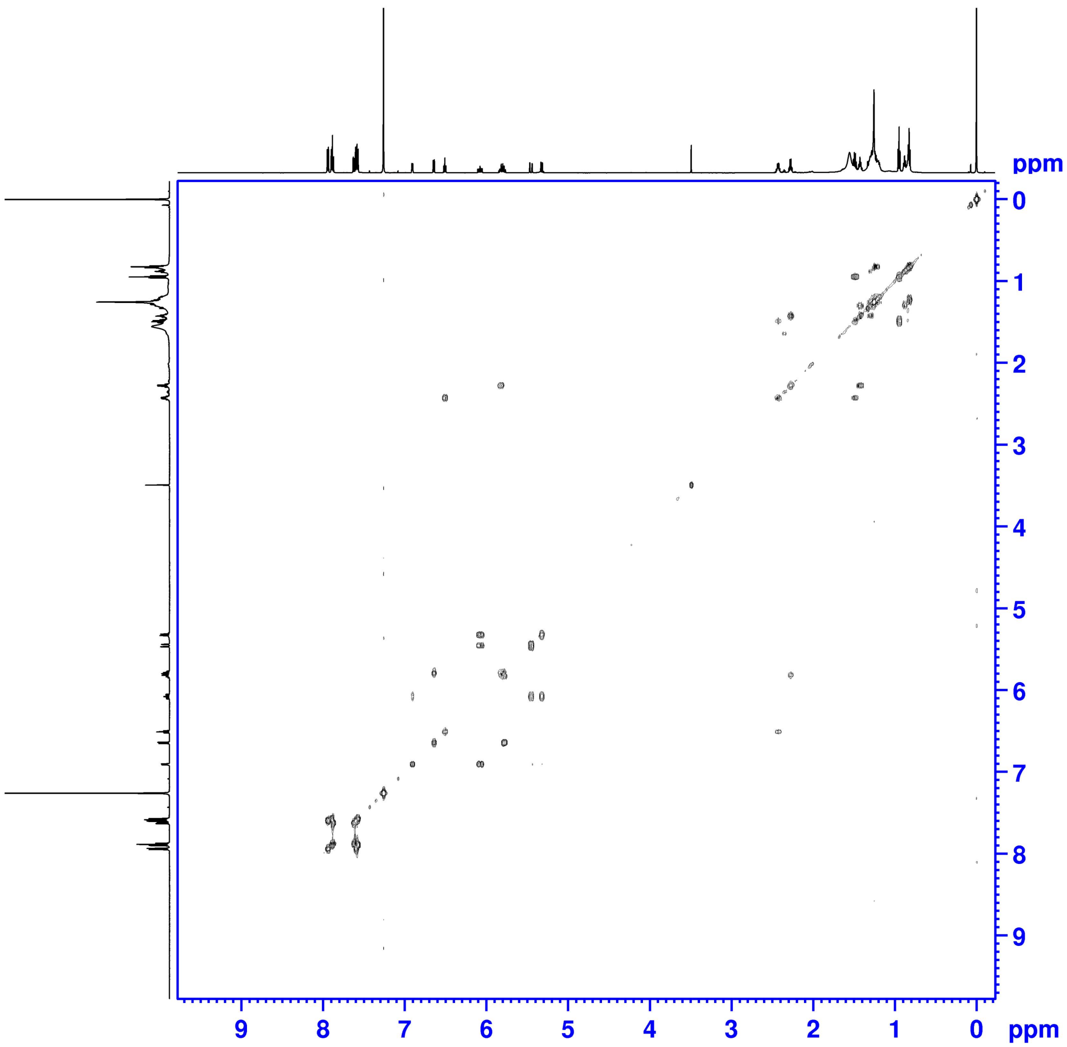

Figure S39. ^{1}H, ^{1}H-COSY spectrum of **1a** ($CDCl_3$)

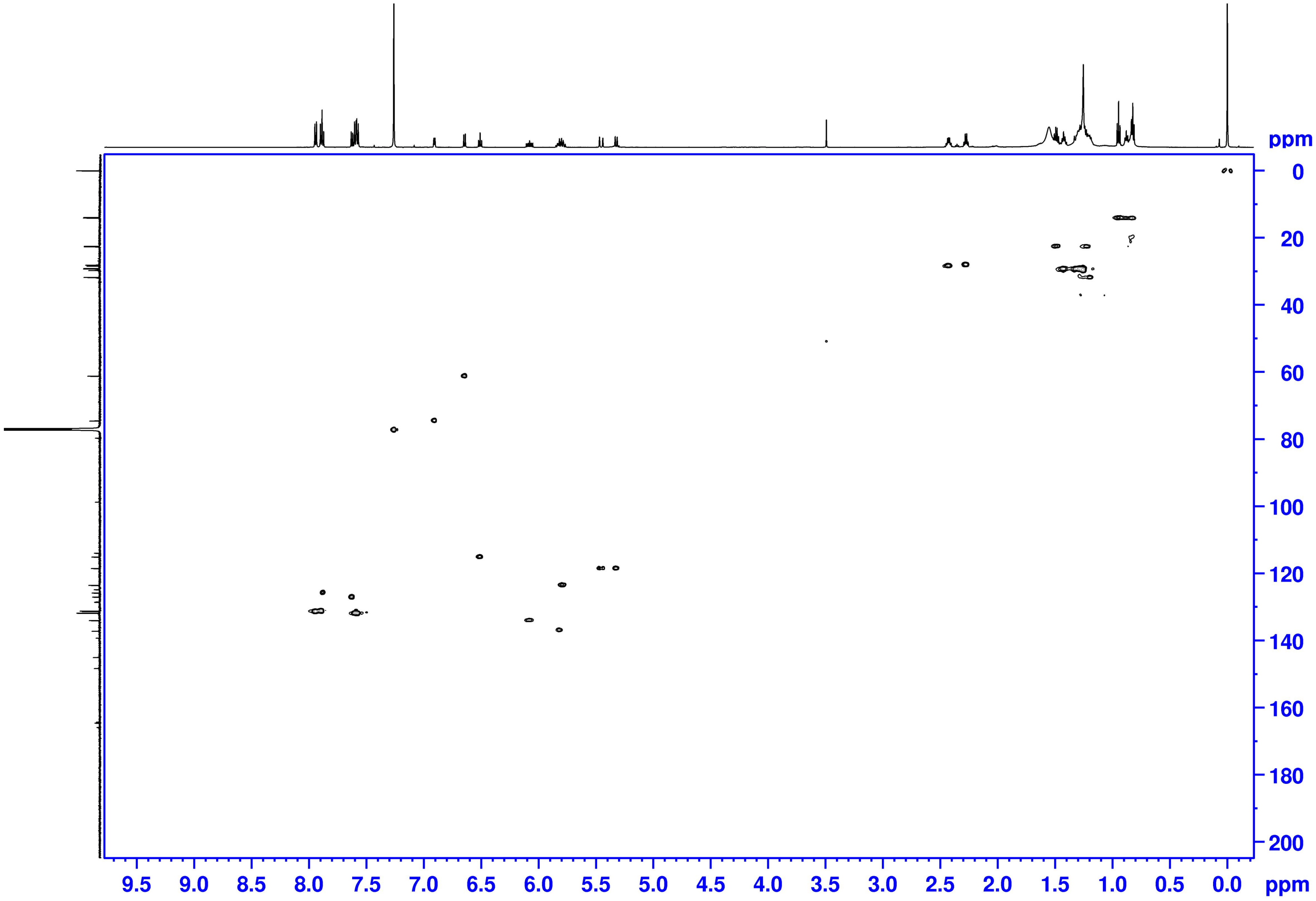

Figure S40. HSQC spectrum of **1a** (CDCl$_3$)

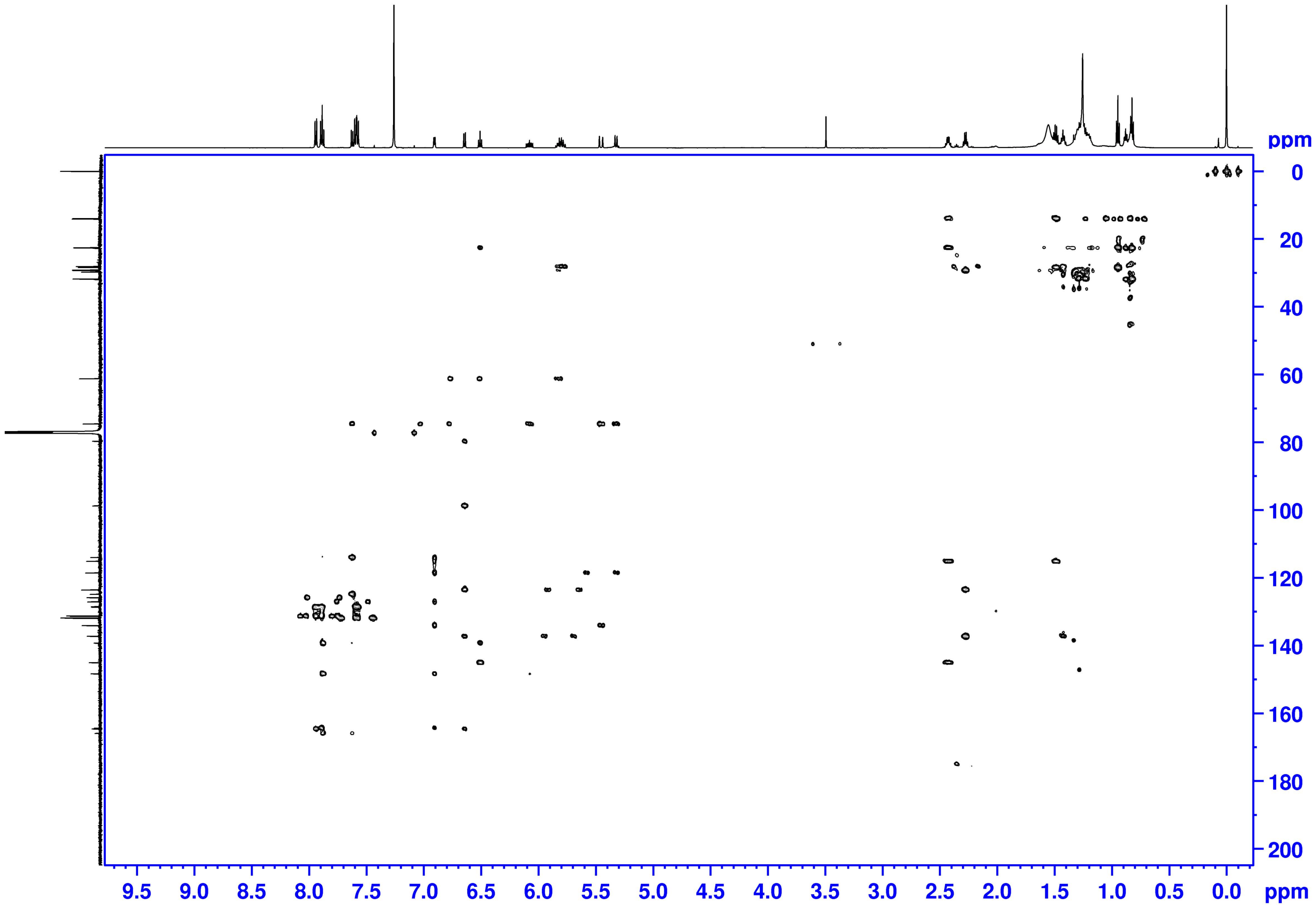

Figure S41. HMBC spectrum of **1a** ($CDCl_3$)

10.3 The 1D and 2D NMR spectra of 2 in $CDCl_3$

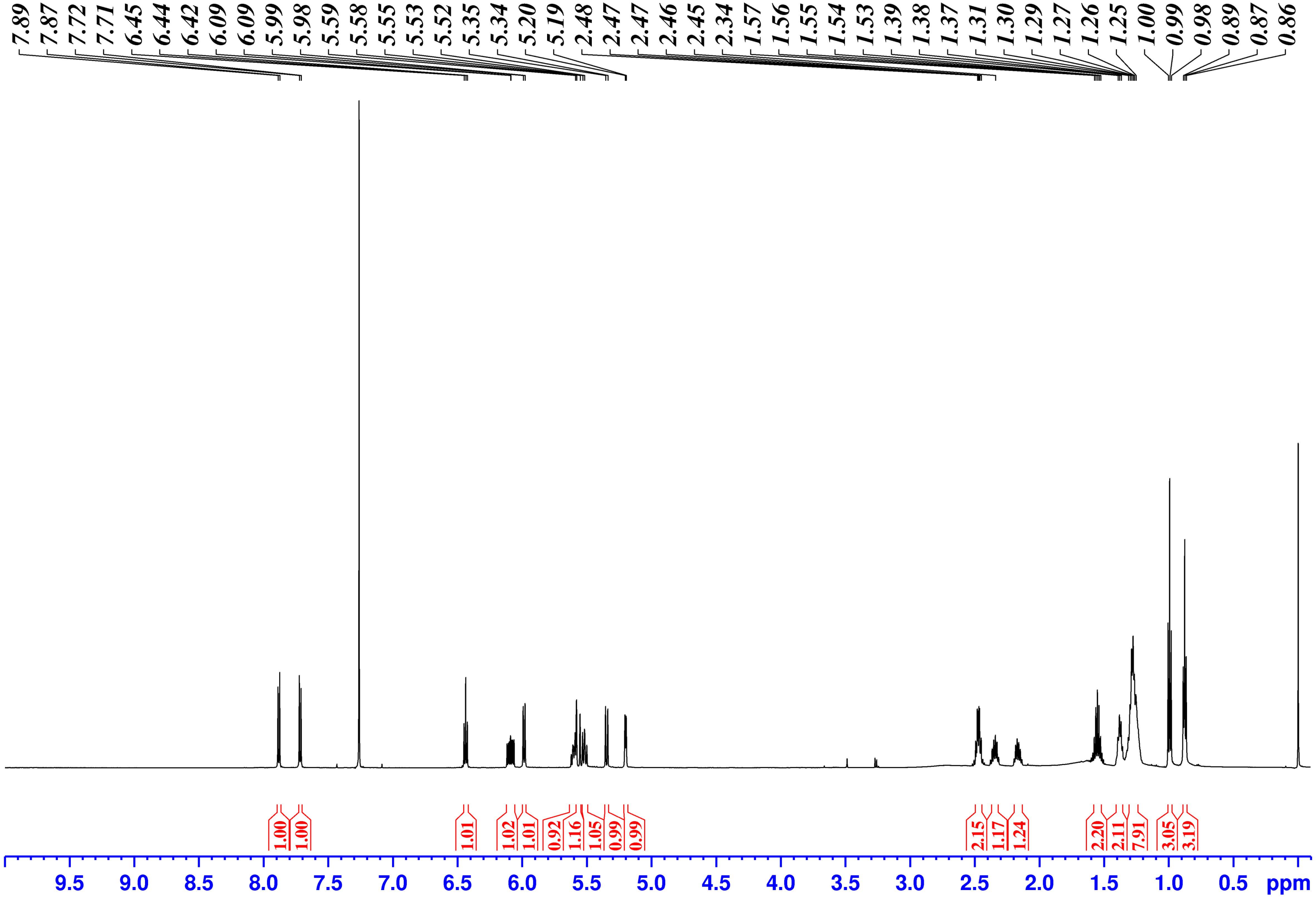

Figure S42. ^{1}H spectrum of falcarinphthalide B (**2**) (600 MHz, $CDCl_3$)

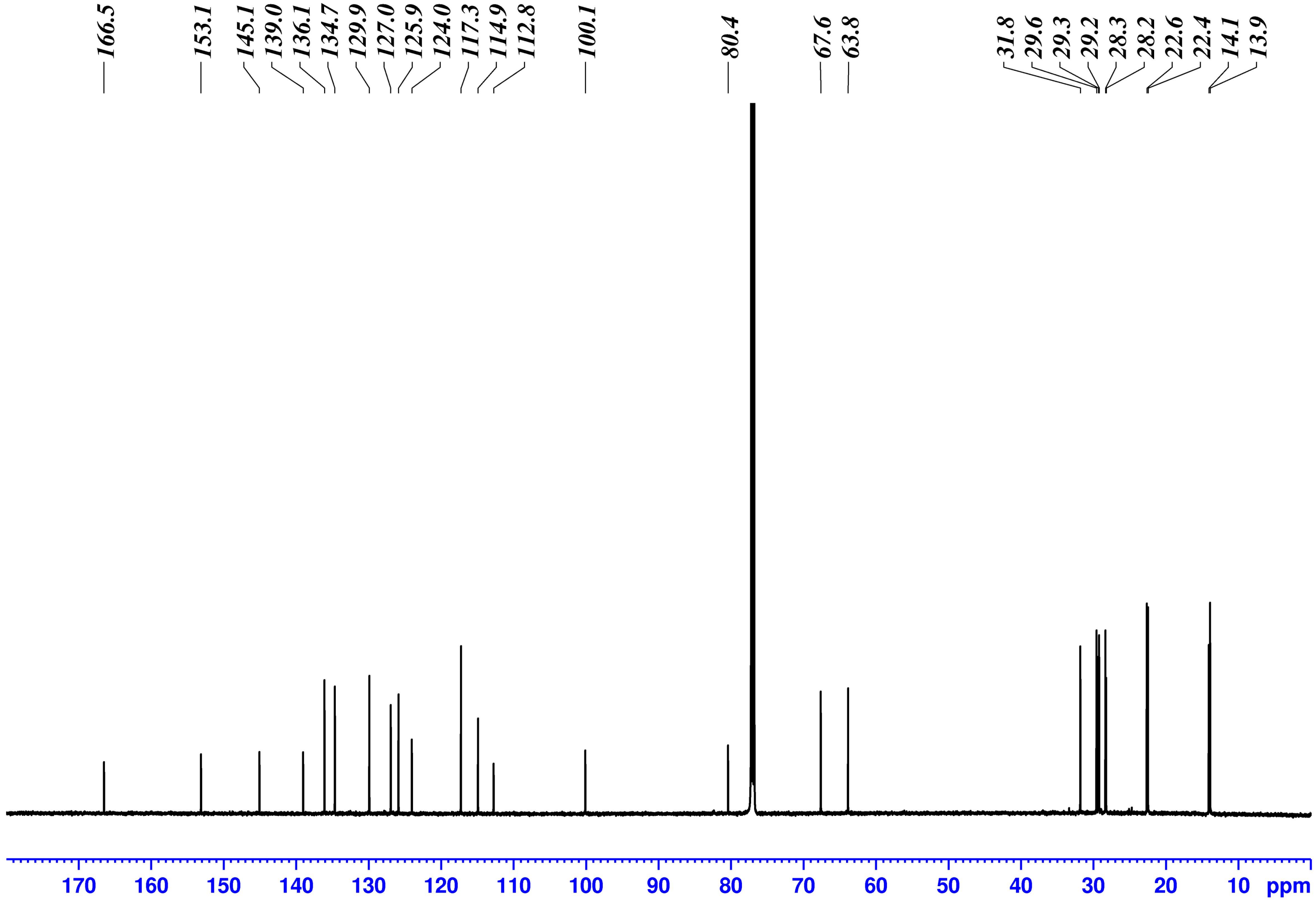

Figure S43. ^{13}C spectrum of falcarinphthalide B (**2**) (151 MHz, $CDCl_3$)

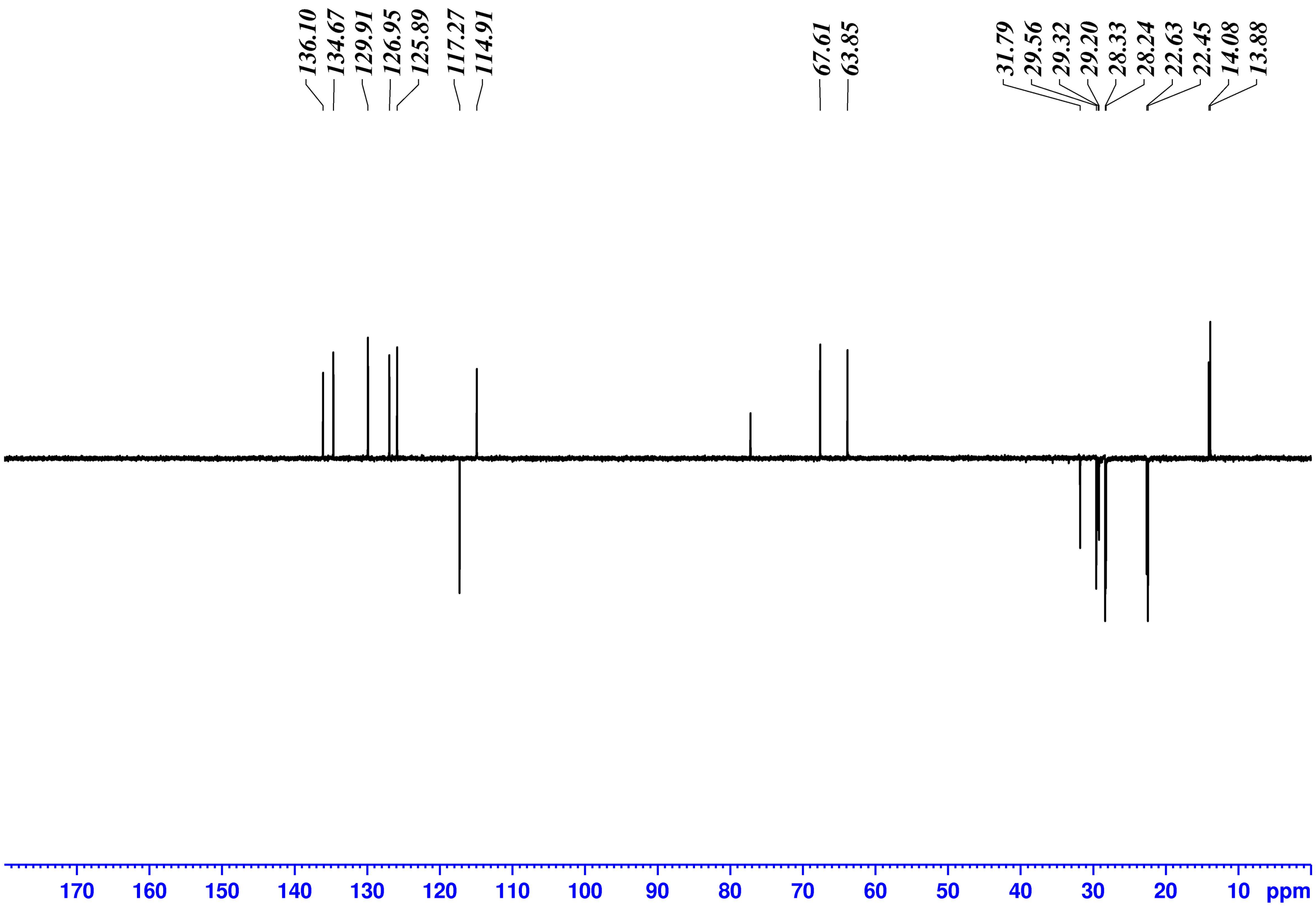

Figure S44. DEPT135 spectrum of falcarinphthalide B (**2**) (151 MHz, $CDCl_3$)

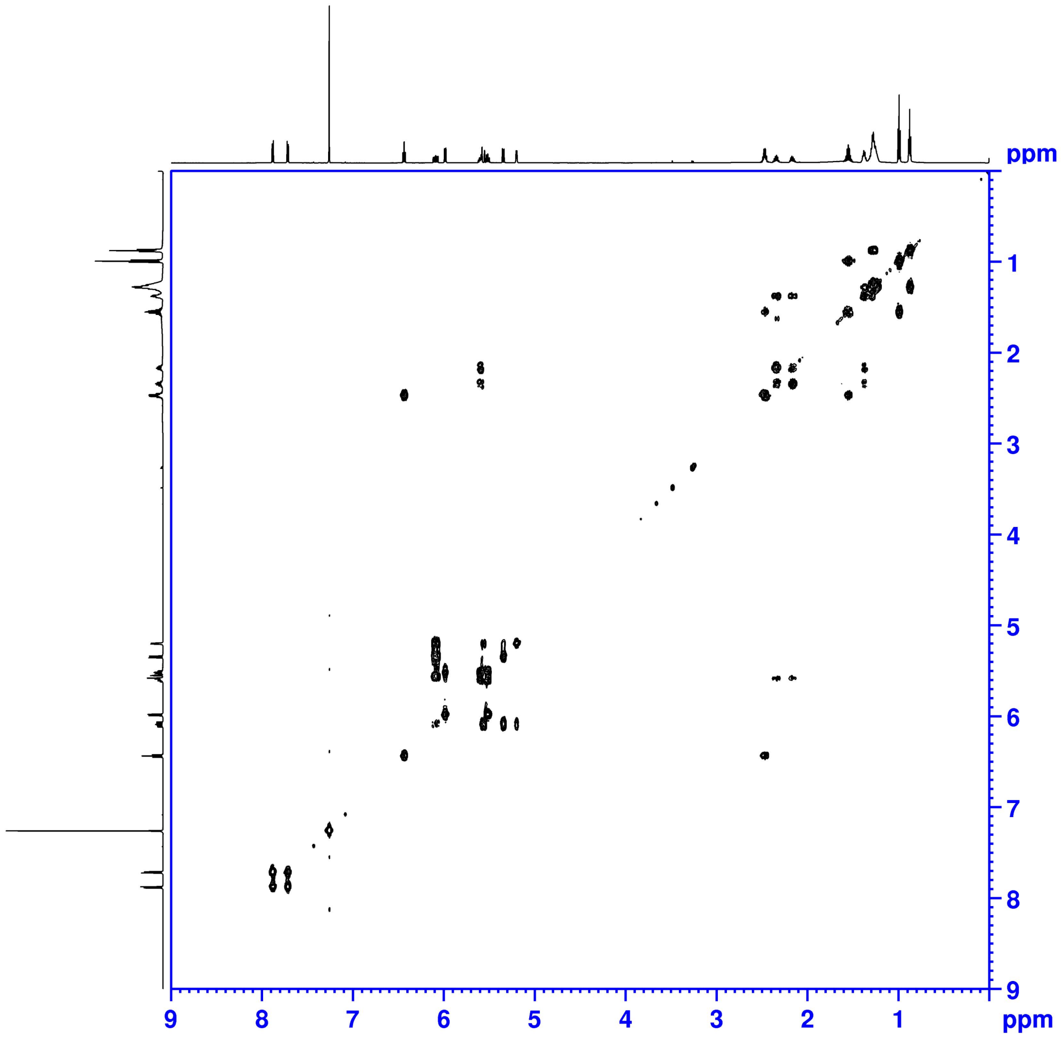

Figure S45. ^{1}H, ^{1}H-COSY spectrum of falcarinphthalide B (**2**) ($CDCl_3$)

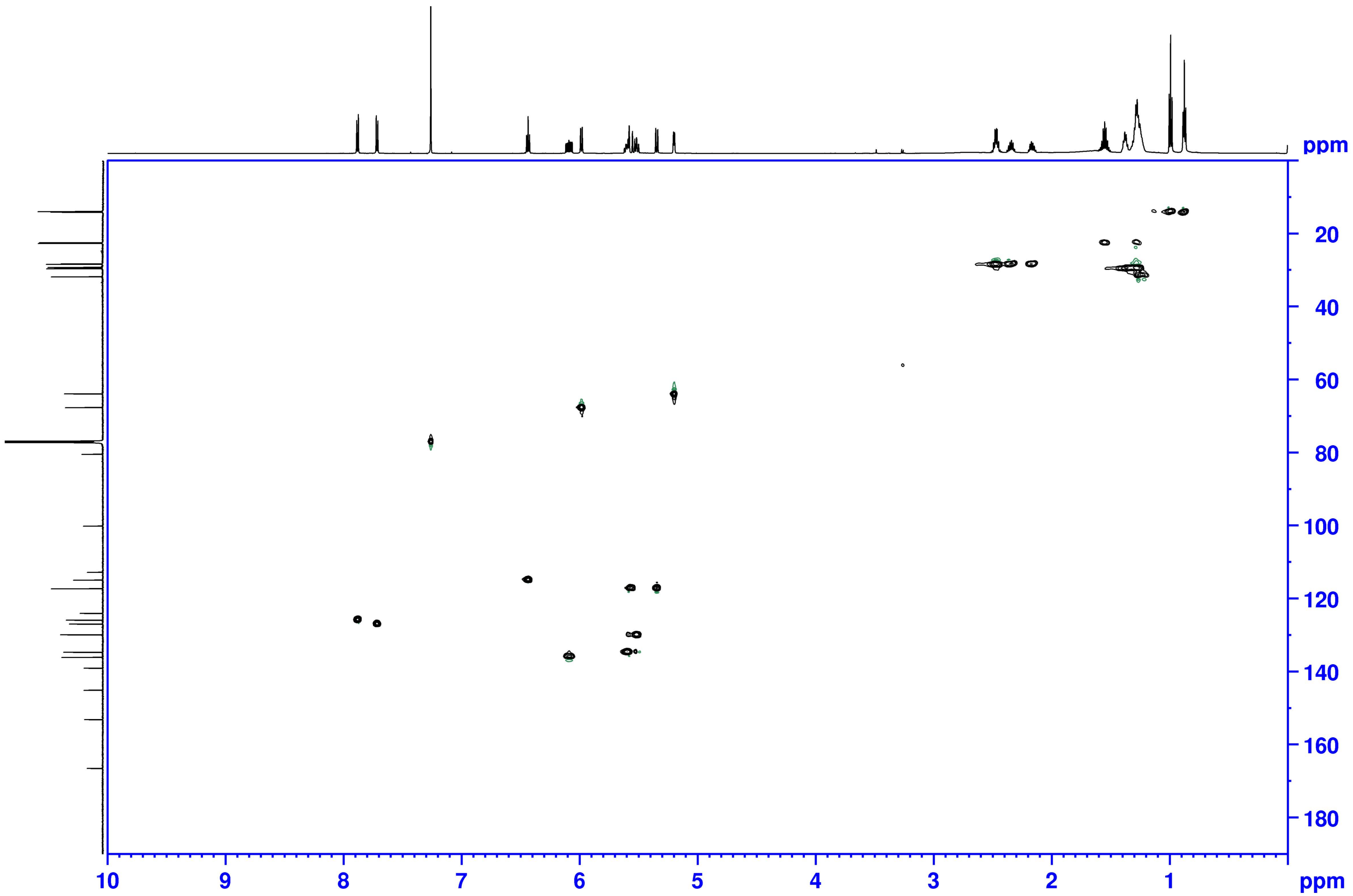

Figure S46. HSQC spectrum of falcarinphthalide B (**2**) ($CDCl_3$)

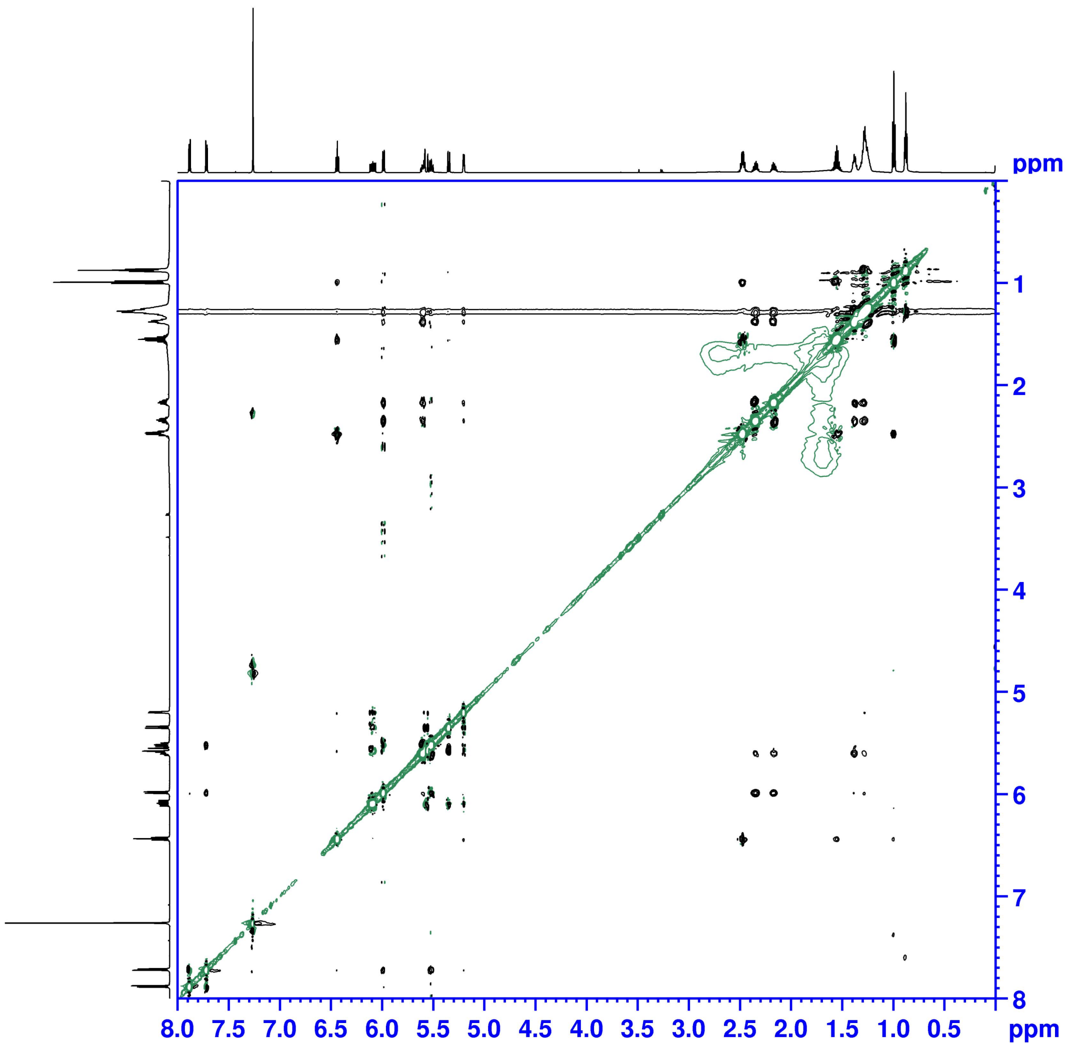

Figure S47. NOESY spectrum of falcarinphthalide B (**2**) ($CDCl_3$)

11. The experimental spectra of total synthesis of falcarinphthalide A (1)

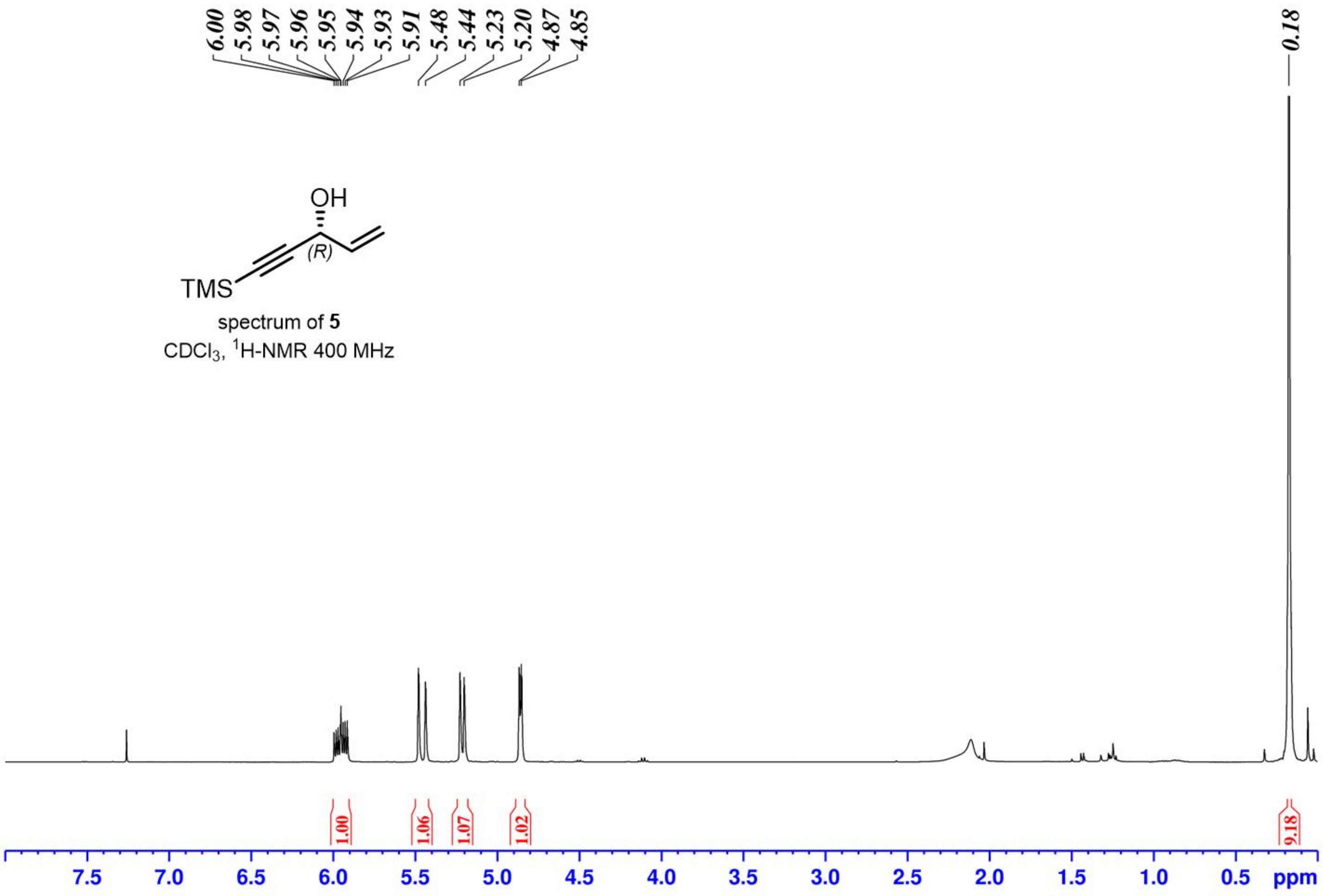

Figure S48. ^{1}H NMR (400 MHz, $CDCl_3$) of **5.**

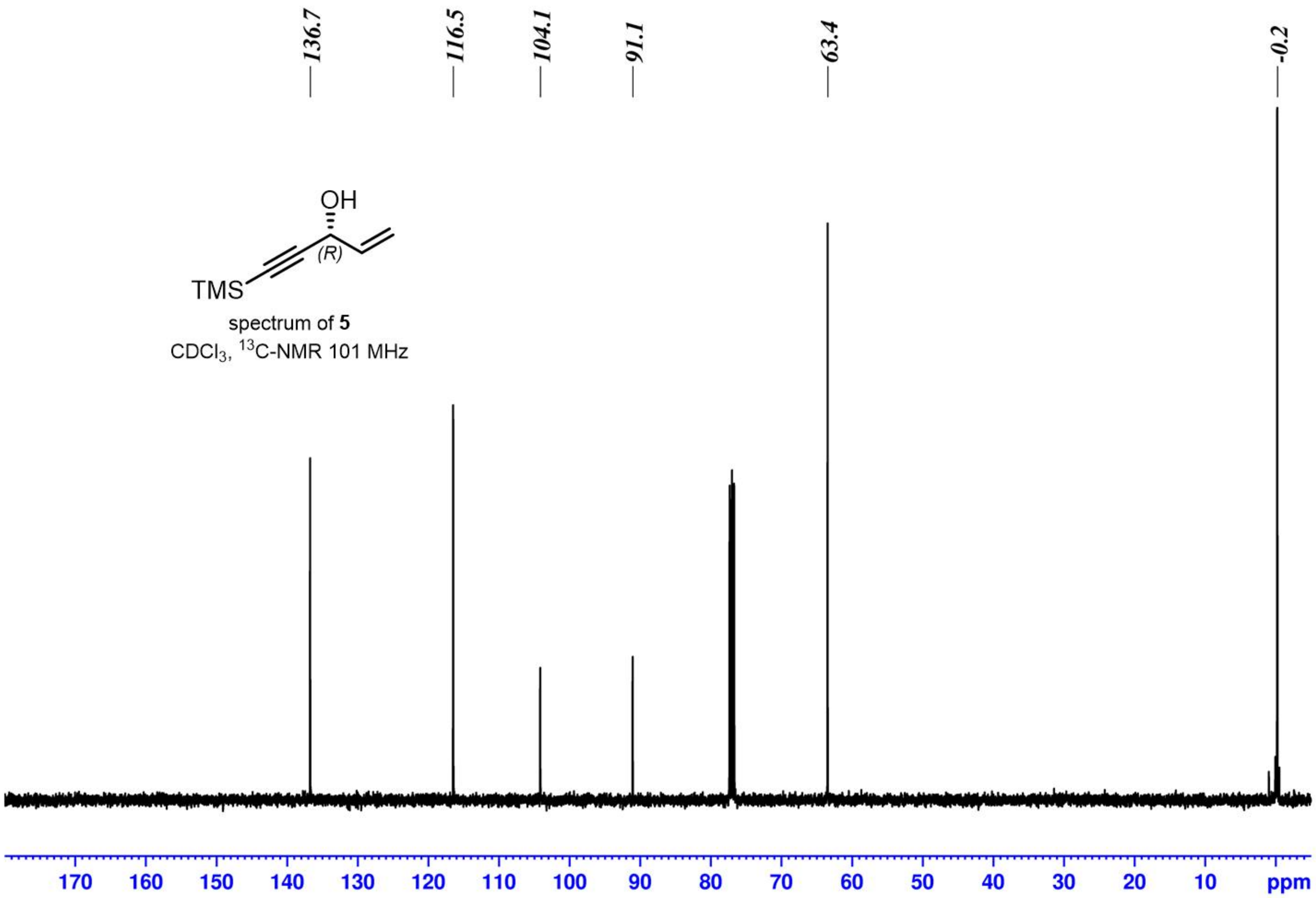

Figure S49. ^{13}C NMR (101 MHz, $CDCl_3$) of **5.**

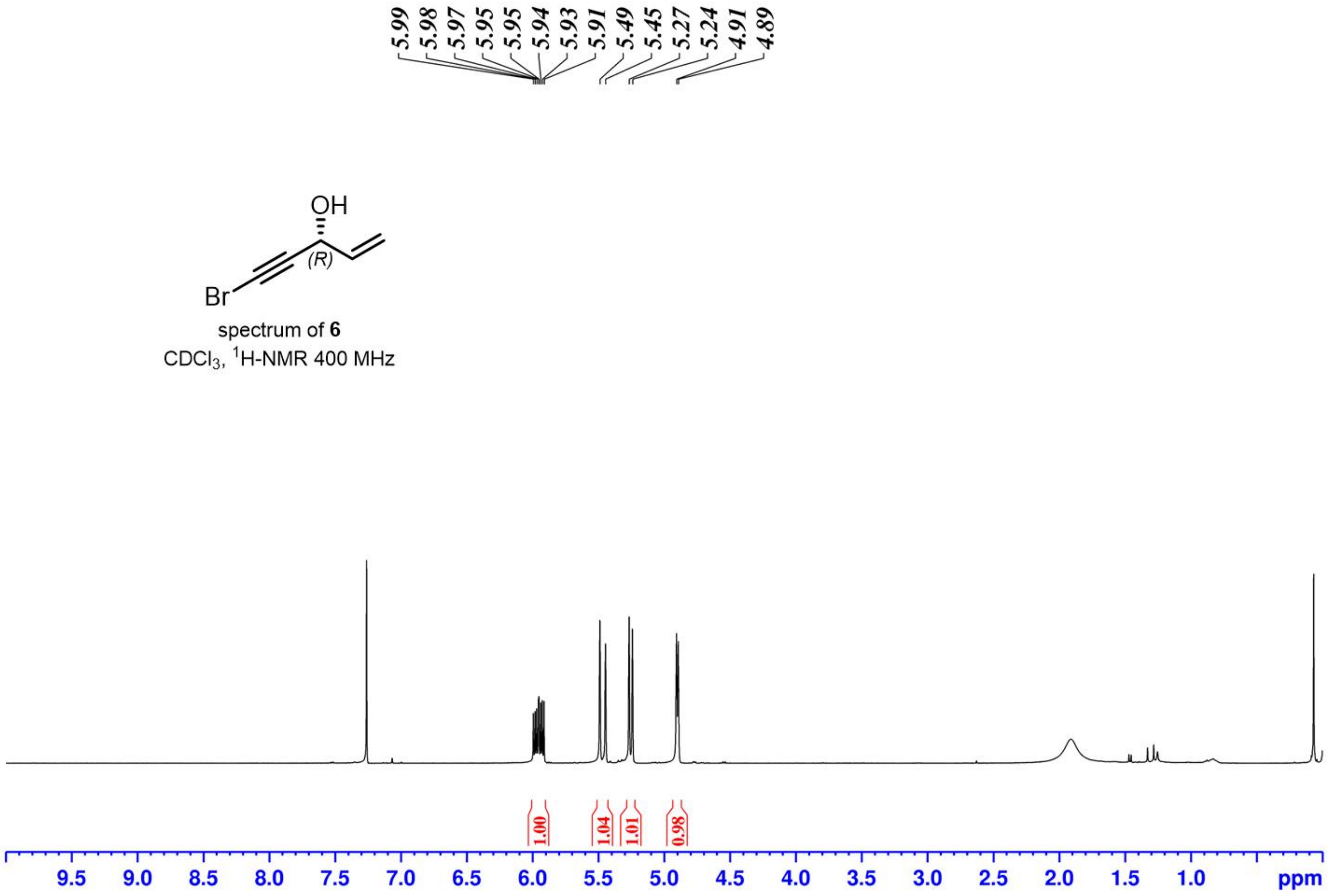

Figure S50. ^{1}H NMR (400 MHz, $CDCl_3$) of **6.**

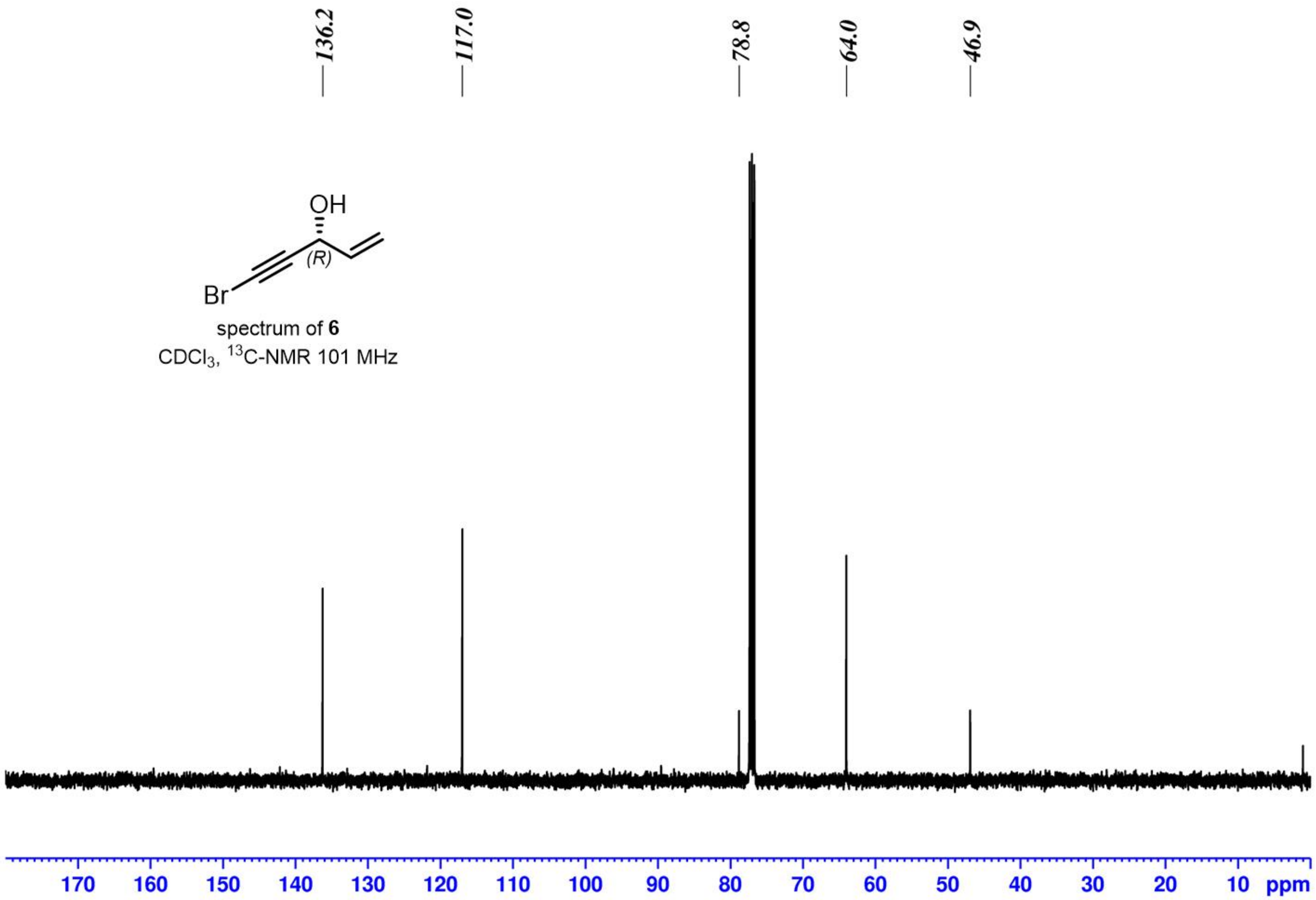

Figure S51. ^{13}C NMR (101 MHz, $CDCl_3$) of **6.**

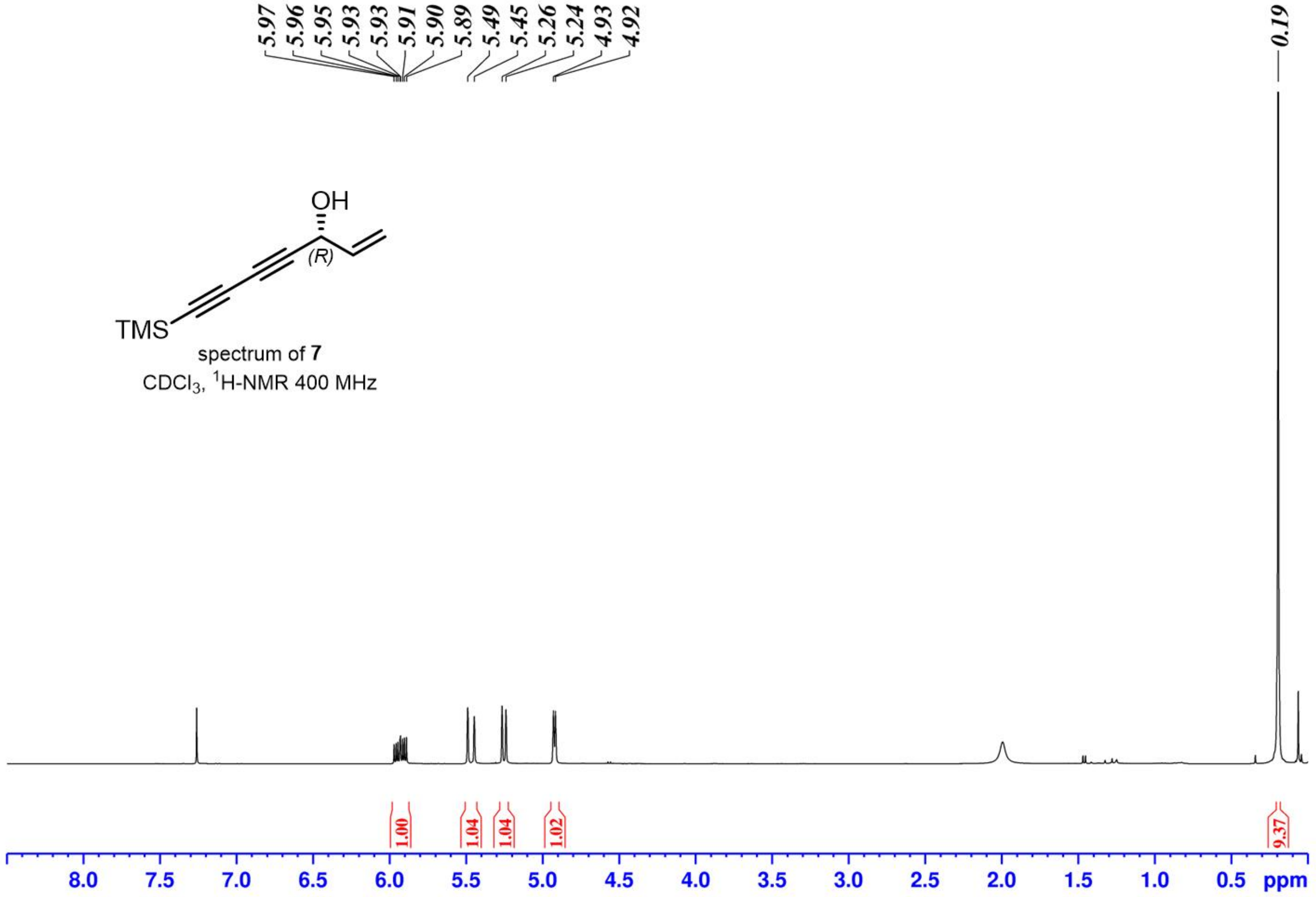

Figure S52. ^{1}H NMR (400 MHz, $CDCl_3$) of **7.**

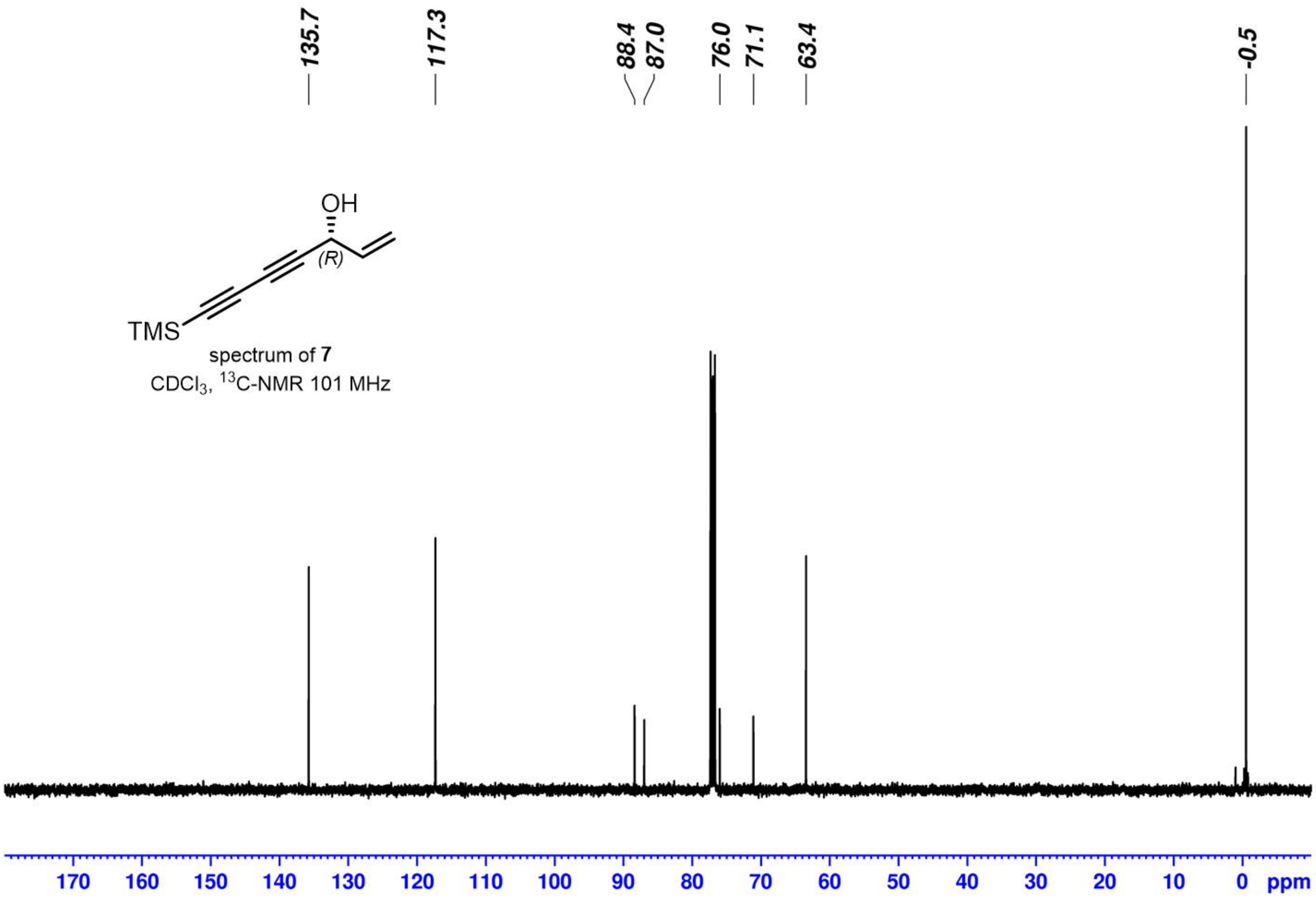

Figure S53. ^{13}C NMR (101 MHz, $CDCl_3$) of **7.**

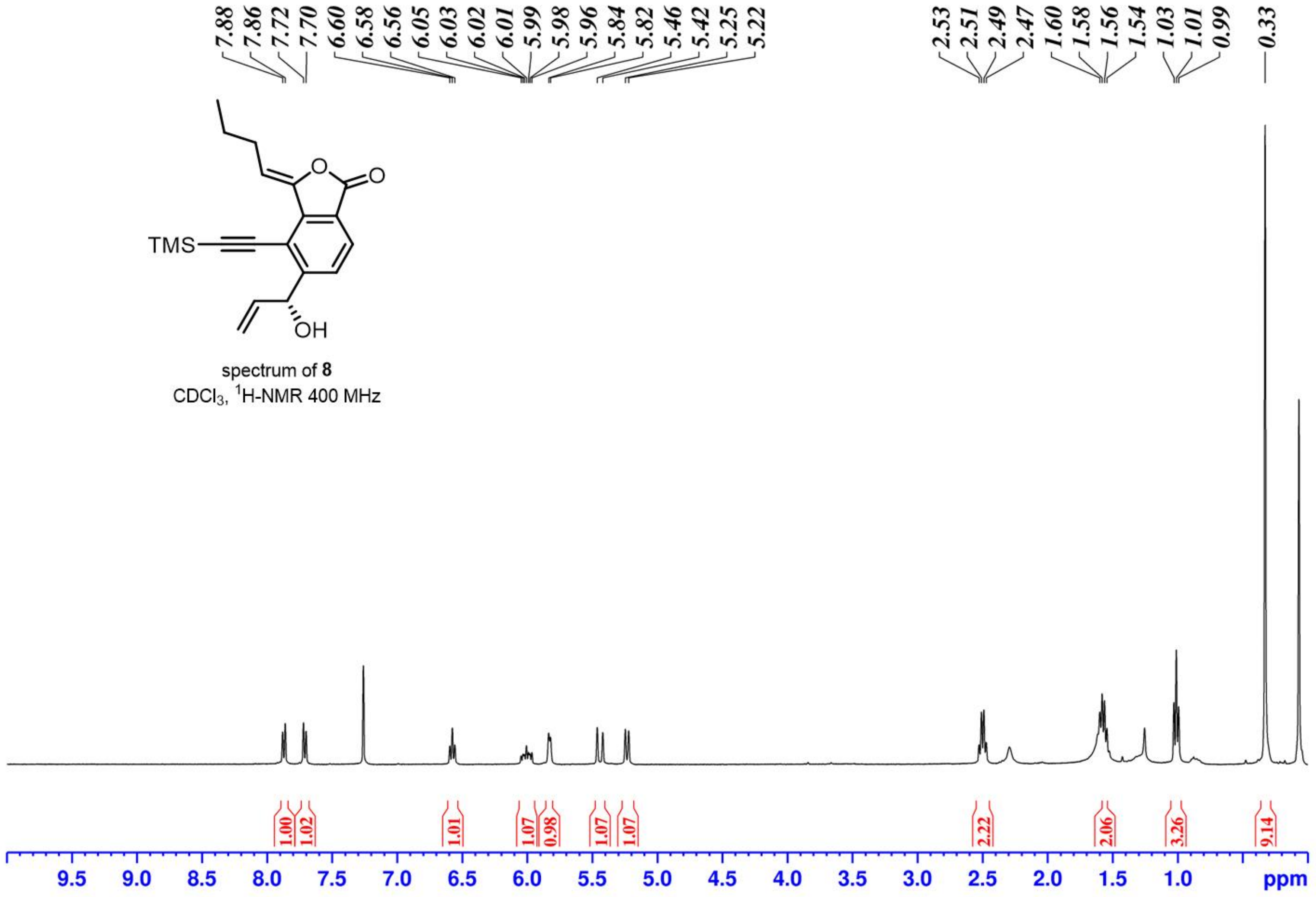

Figure S54. ^{1}H NMR (400 MHz, $CDCl_3$) of **8.**

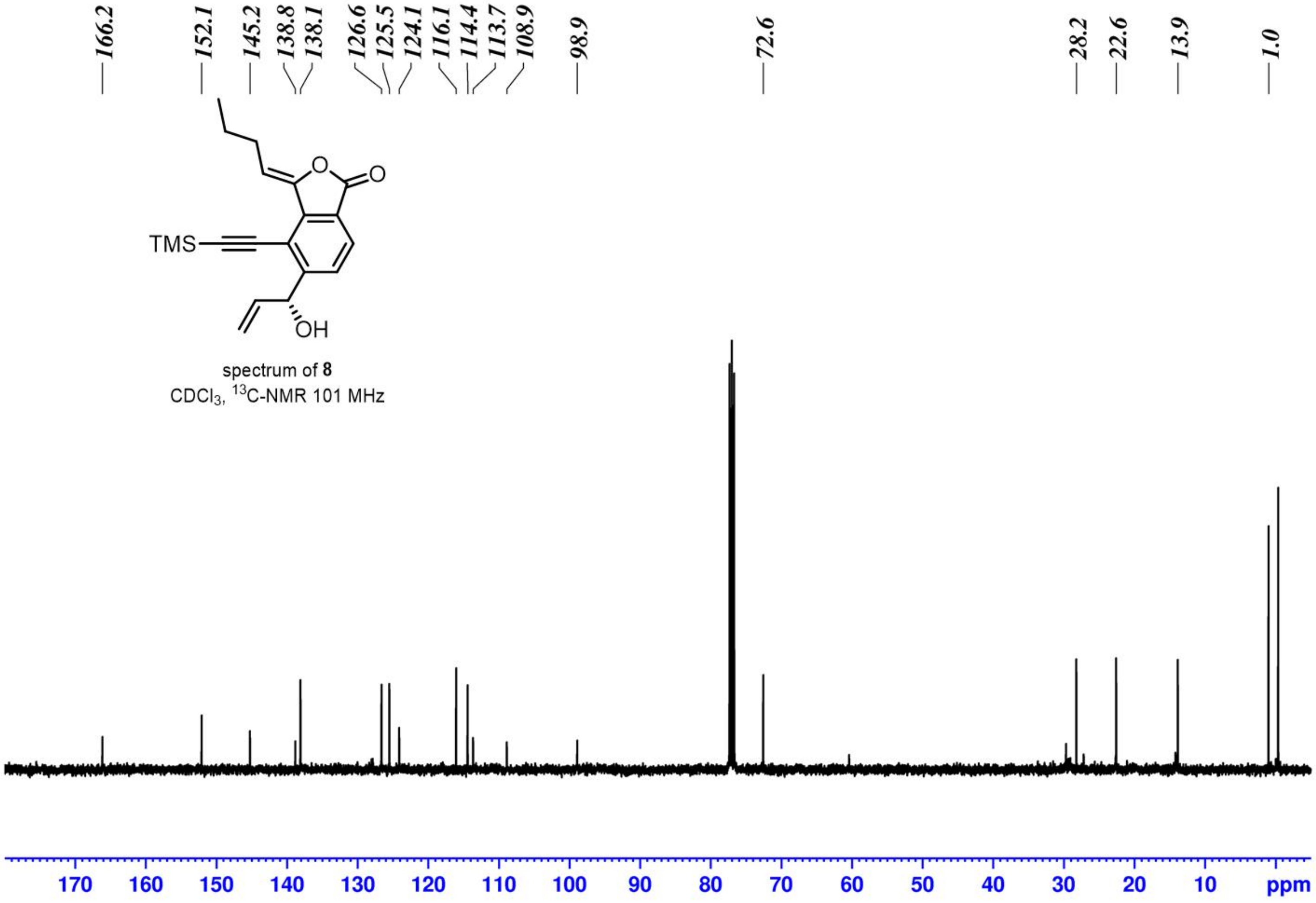

Figure S55. ^{13}C NMR (101 MHz, $CDCl_3$) of **8.**

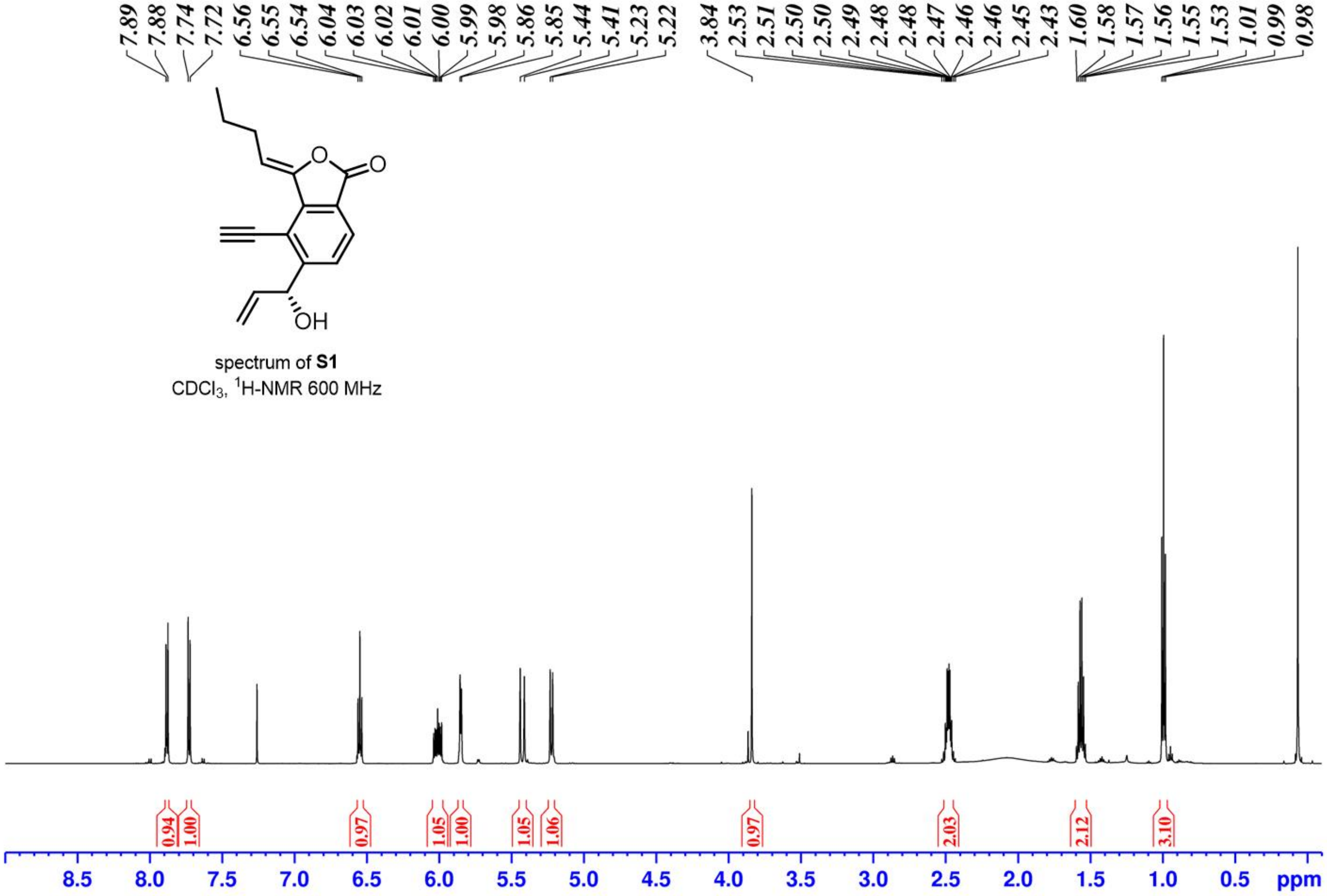

Figure S56. ^{1}H NMR (600 MHz, $CDCl_3$) of **S1.**

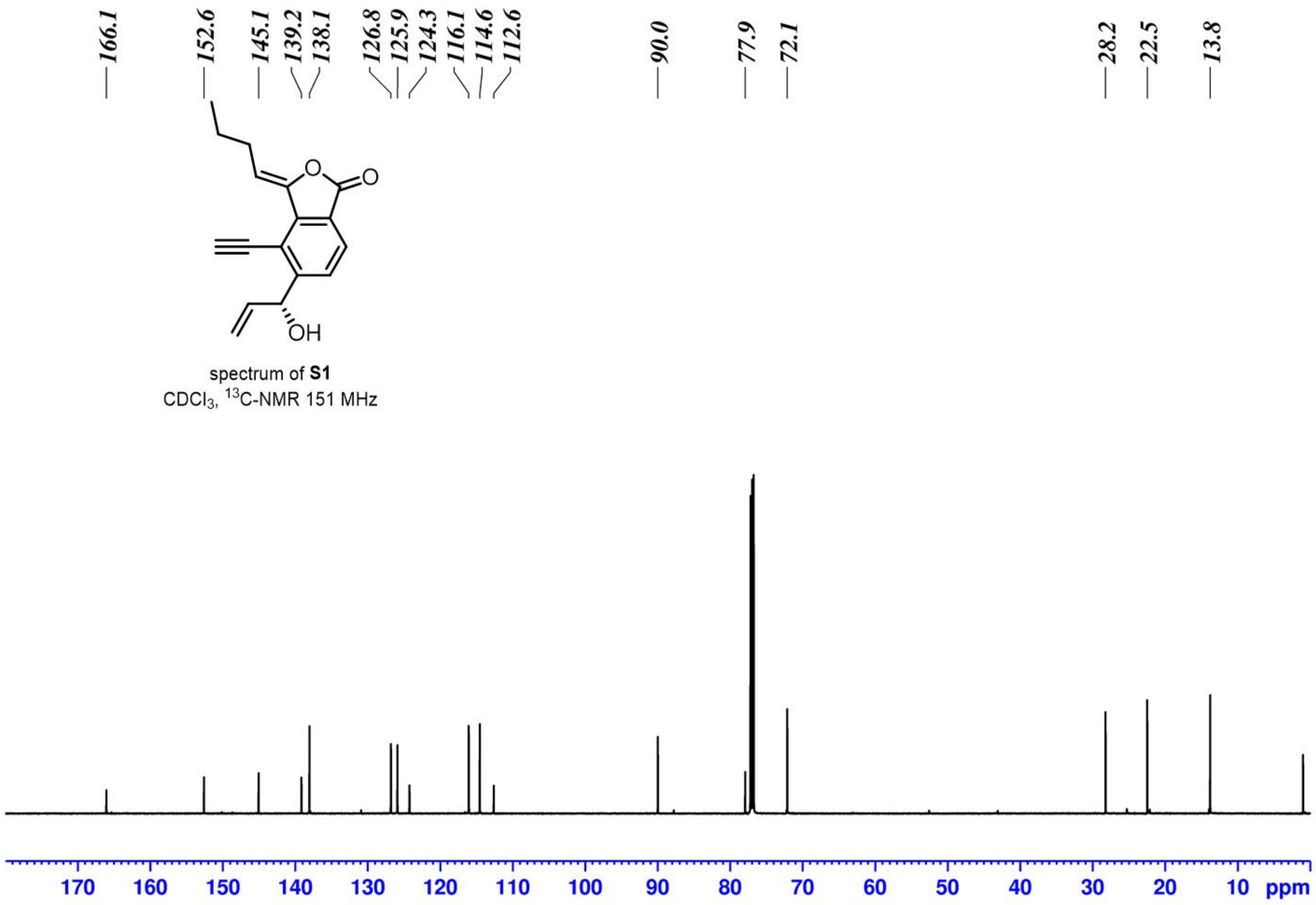

Figure S57. ^{13}C NMR (151 MHz, $CDCl_3$) of **S1.**

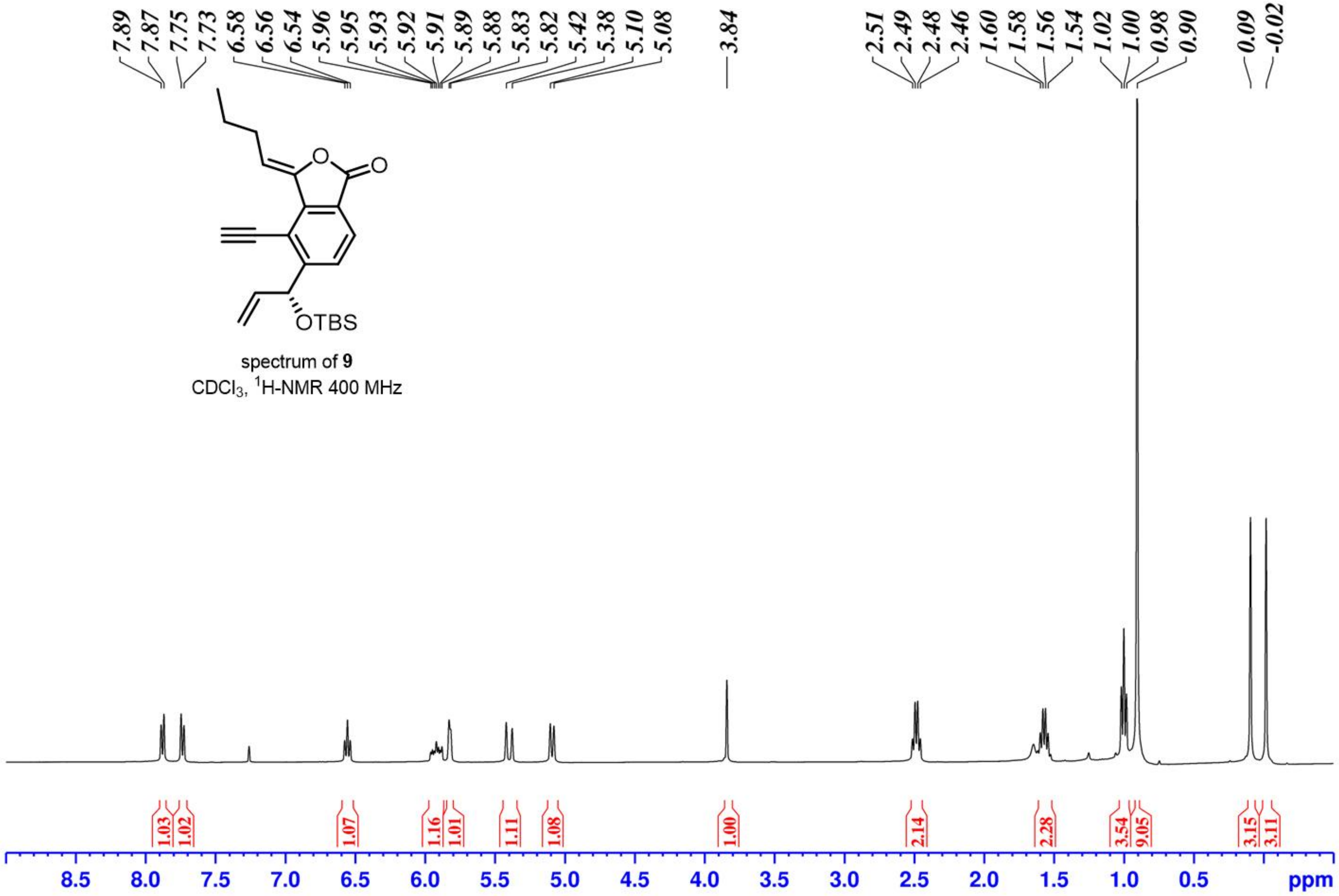

Figure S58. ^{1}H NMR (400 MHz, $CDCl_3$) of **9.**

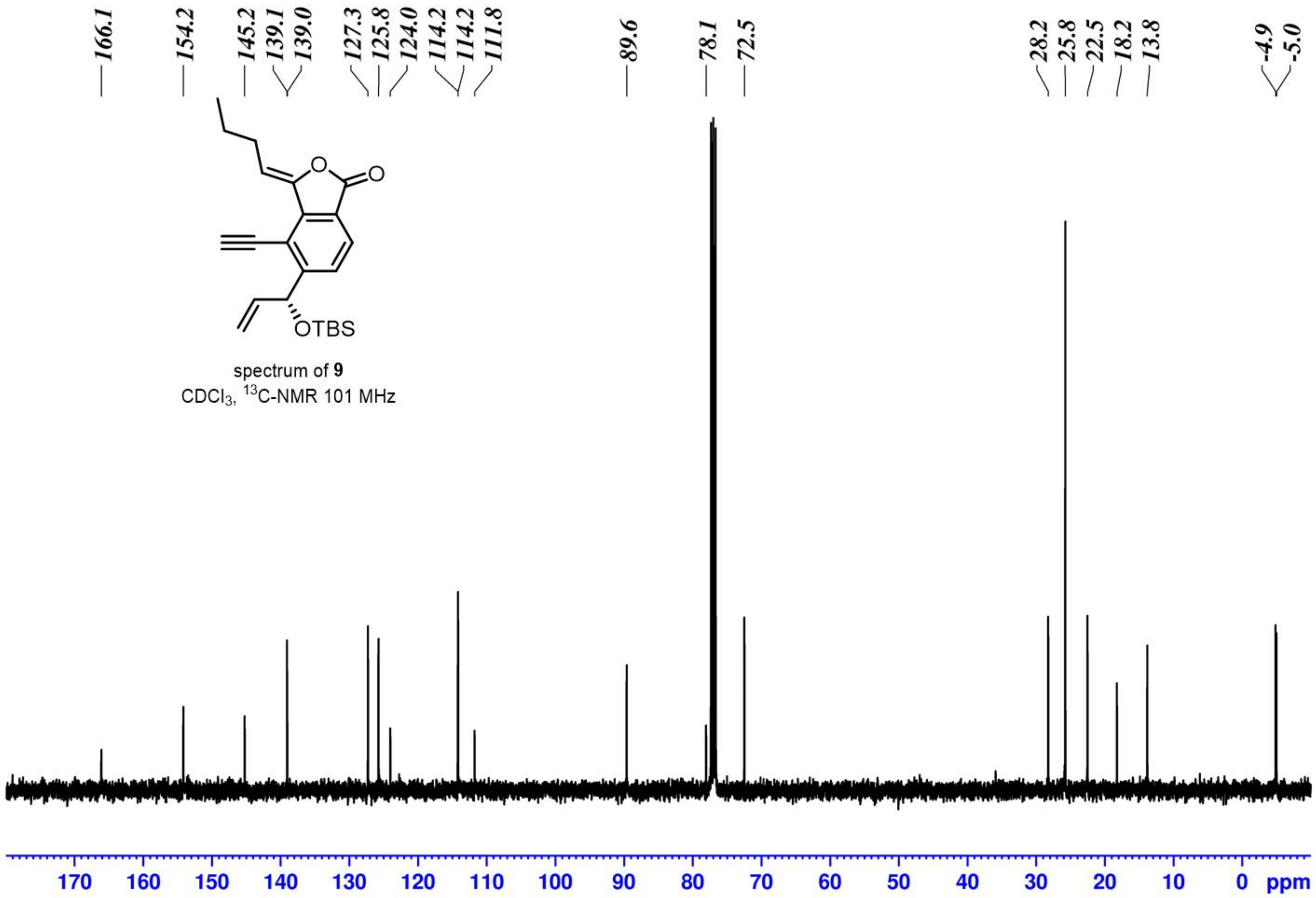

Figure S59. ^{13}C NMR (101 MHz, $CDCl_3$) of **9.**

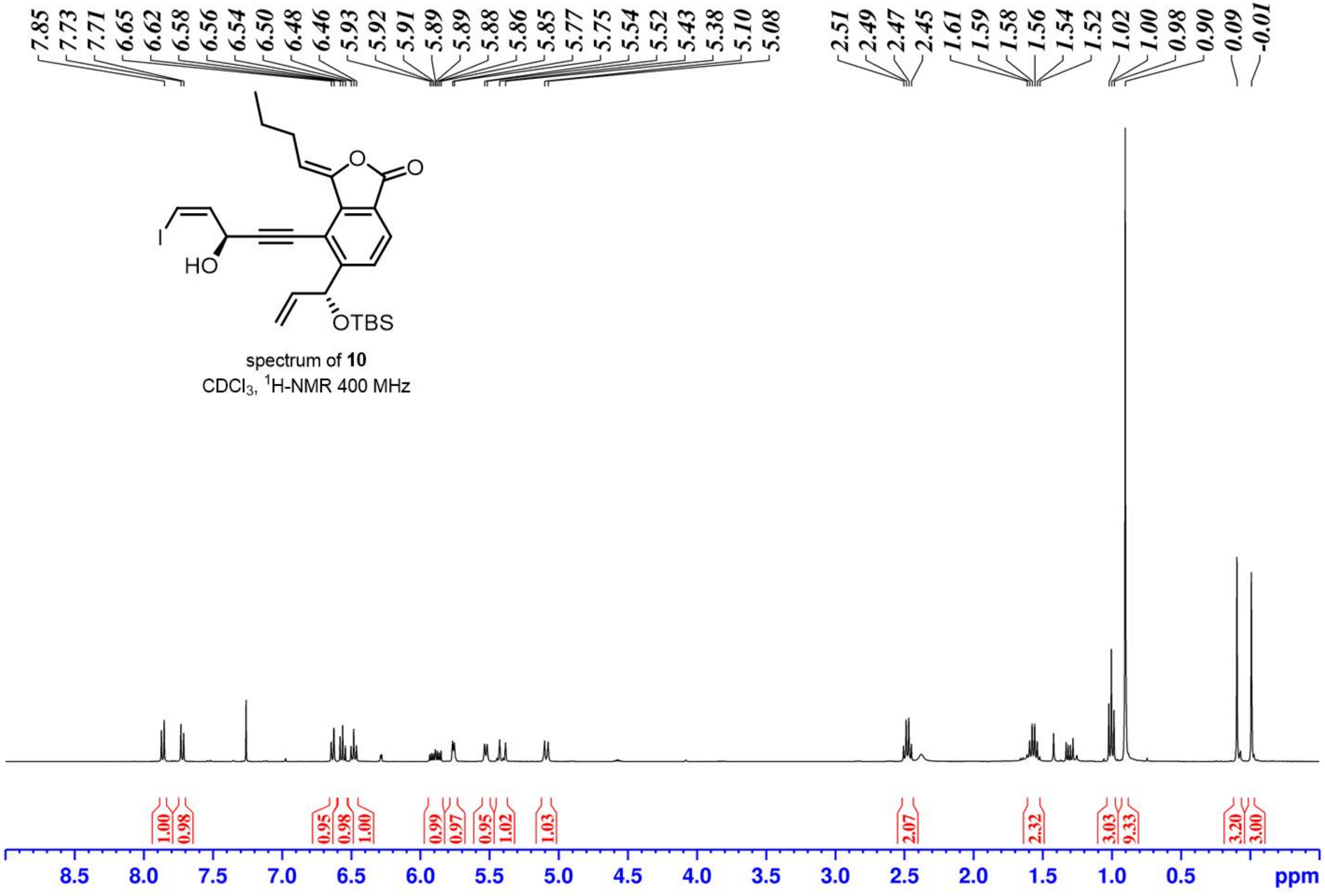

Figure S60. ^{1}H NMR (400 MHz, $CDCl_3$) of **10.**

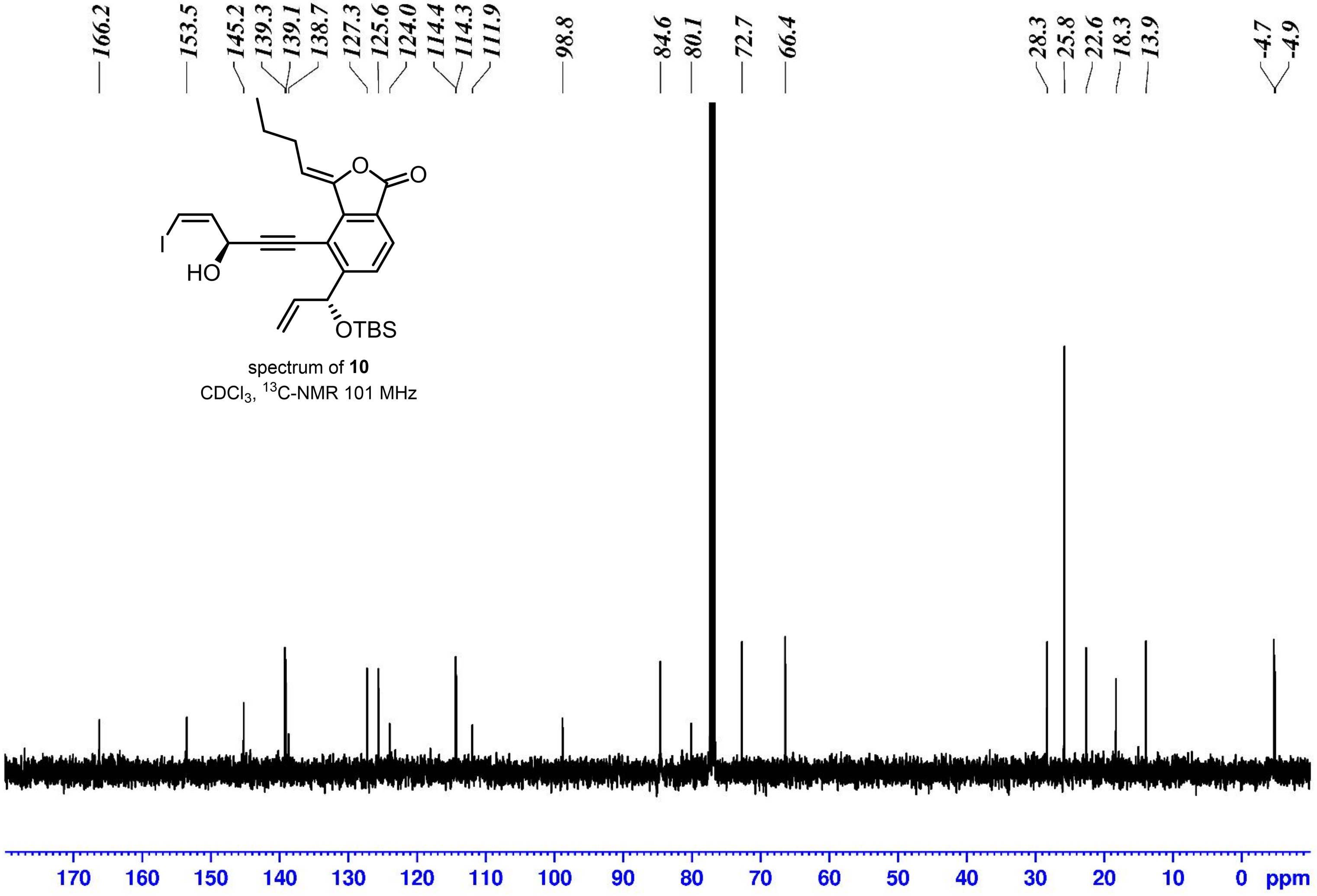

Figure S61. ^{13}C NMR (101 MHz, $CDCl_3$) of **10.**

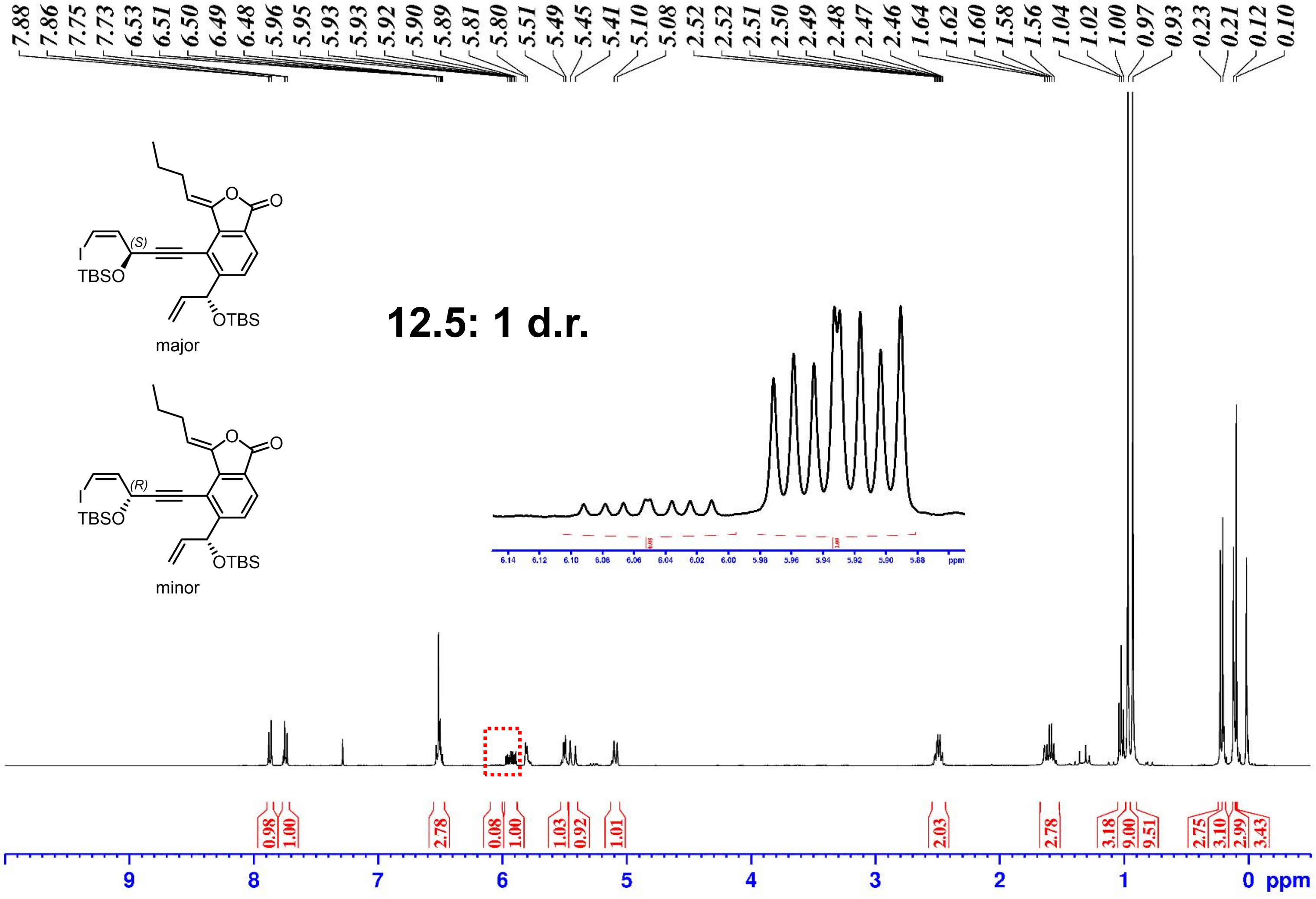

Figure S62. ^{1}H NMR (400 MHz, $CDCl_3$) of **S2.**

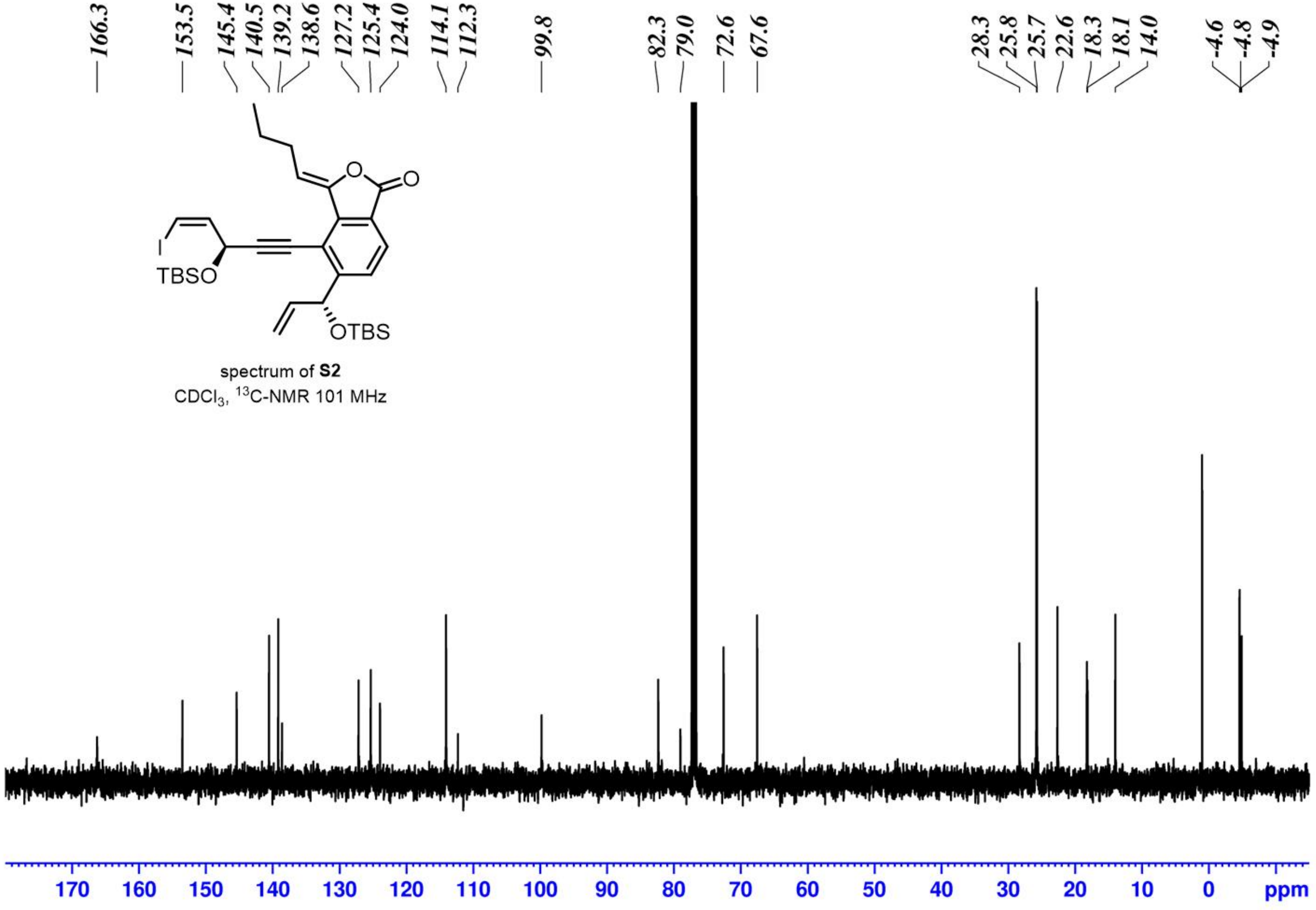

Figure S63. ^{13}C NMR (101 MHz, $CDCl_3$) of **S2.**

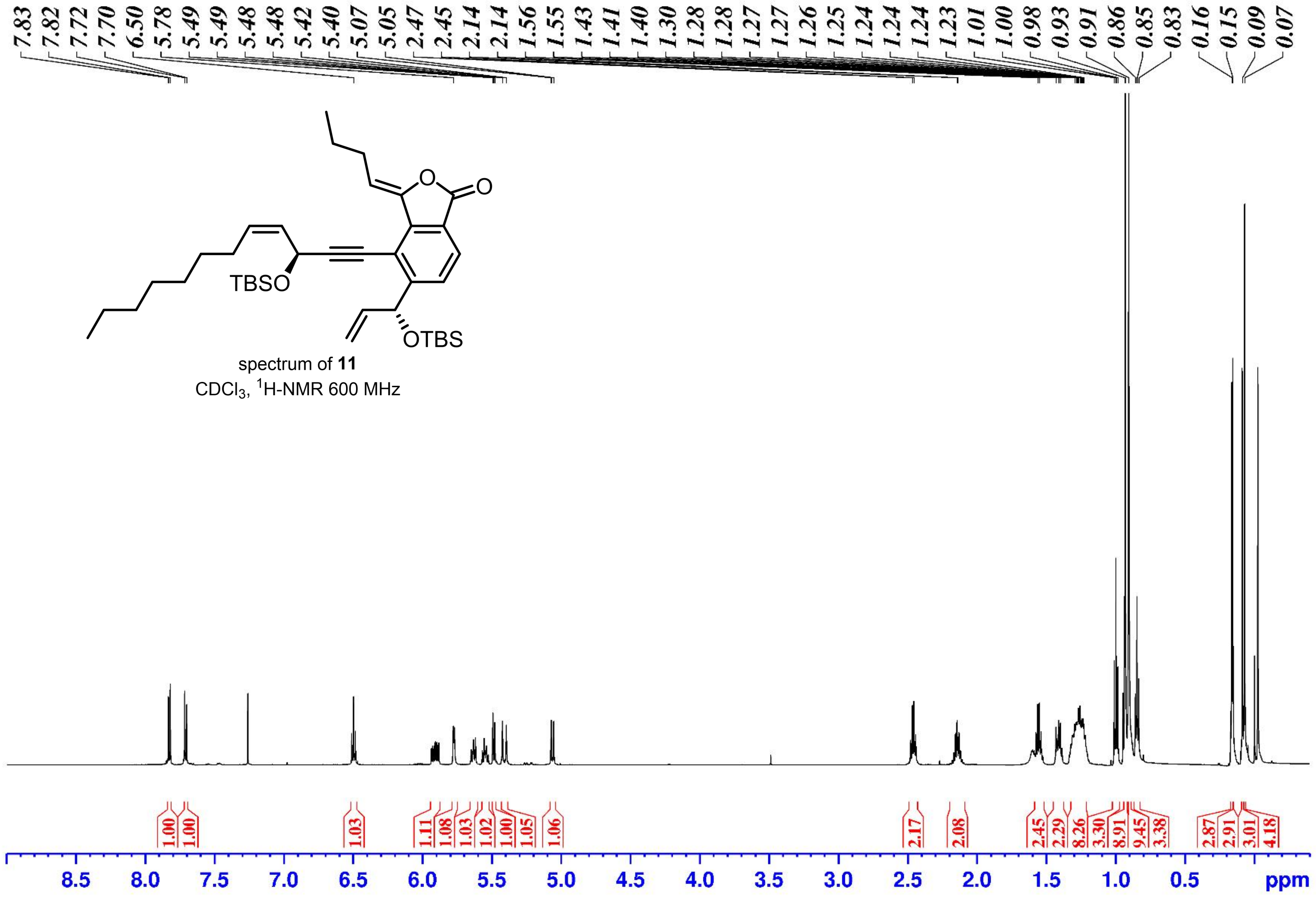

Figure S64. ^{1}H NMR (600 MHz, $CDCl_3$) of **11.**

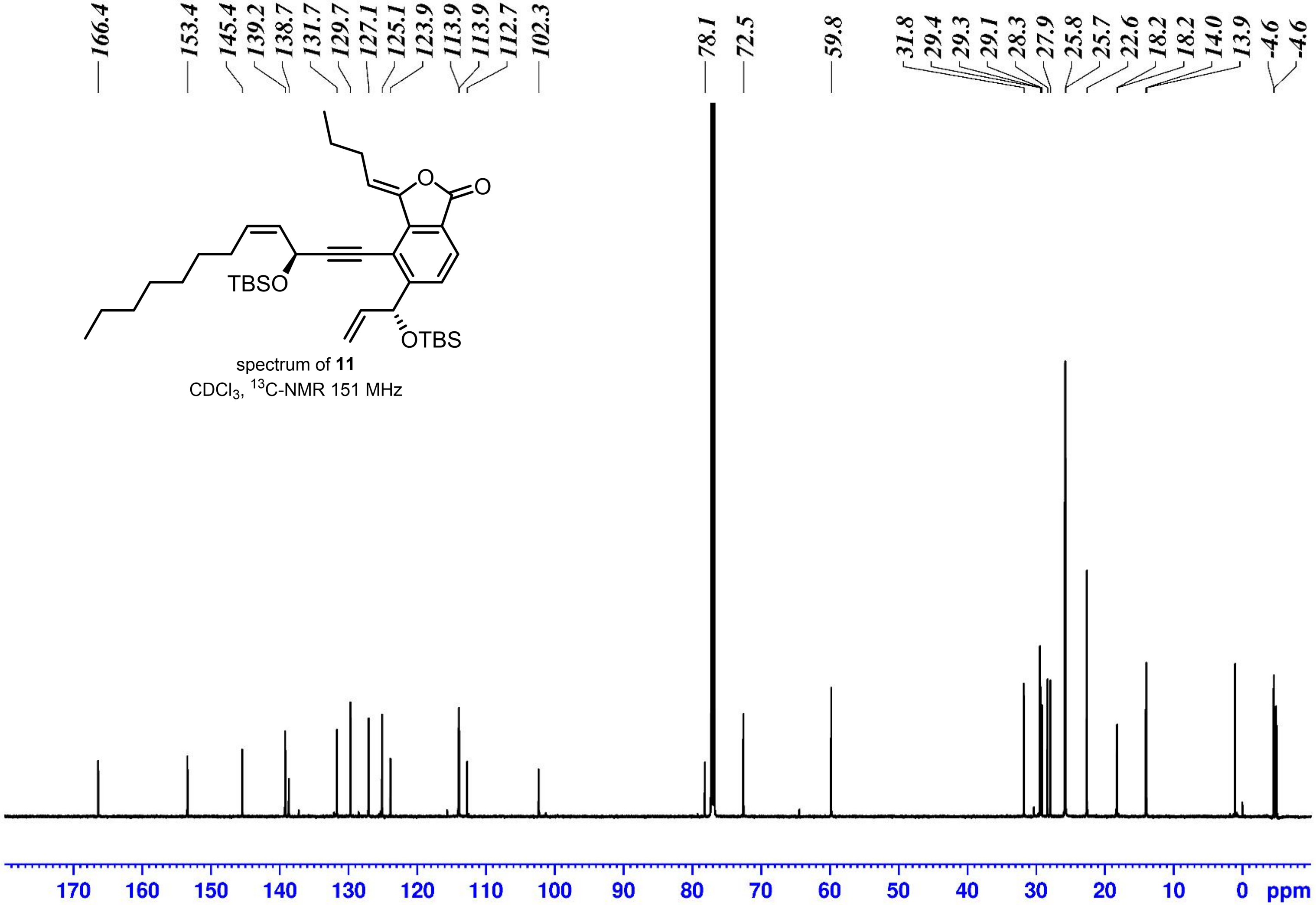

Figure S65. ^{13}C NMR (151 MHz, $CDCl_3$) of **11.**

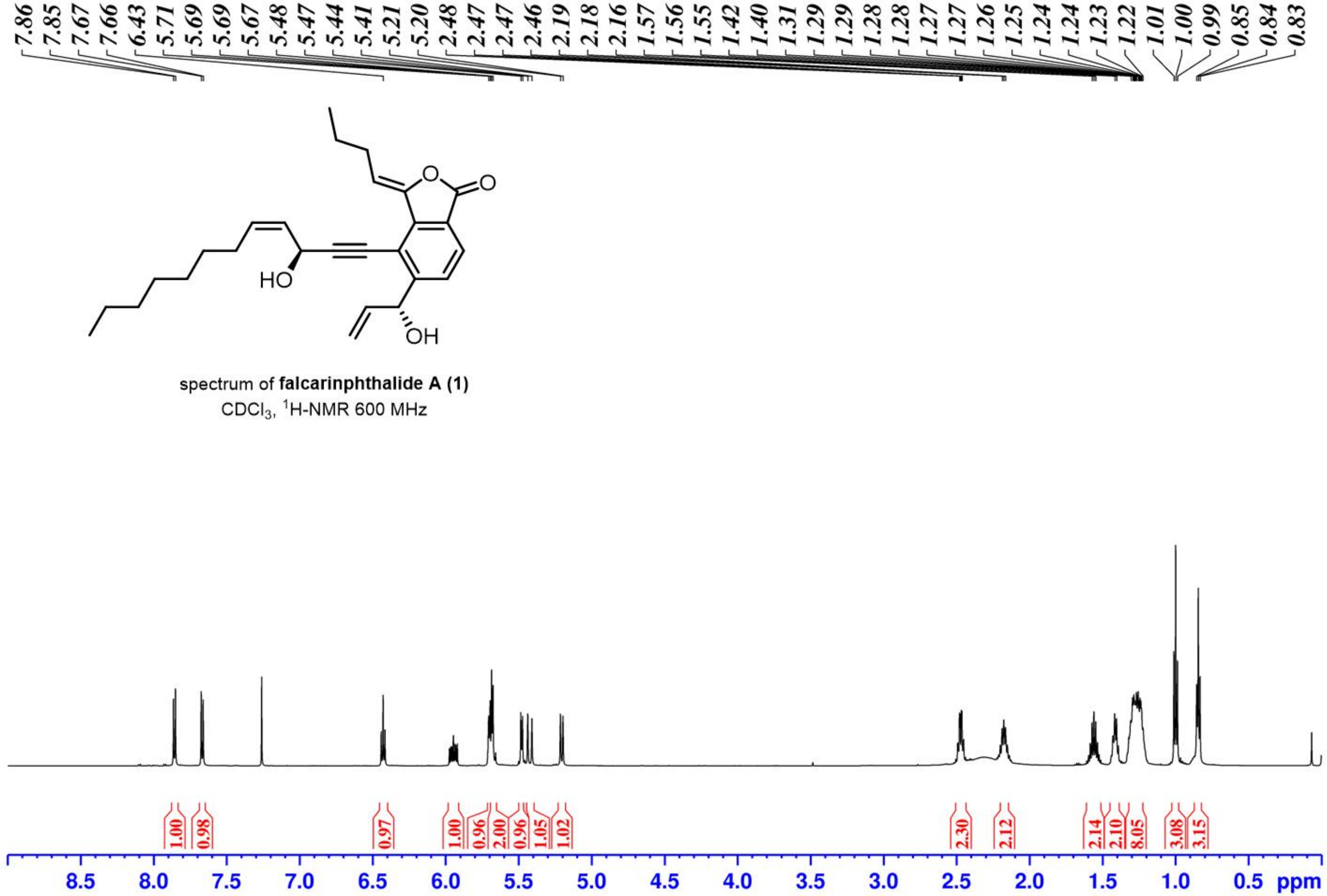

Figure S66. ^{1}H NMR (600 MHz, $CDCl_3$) of falcarinphthalide A (**1**).

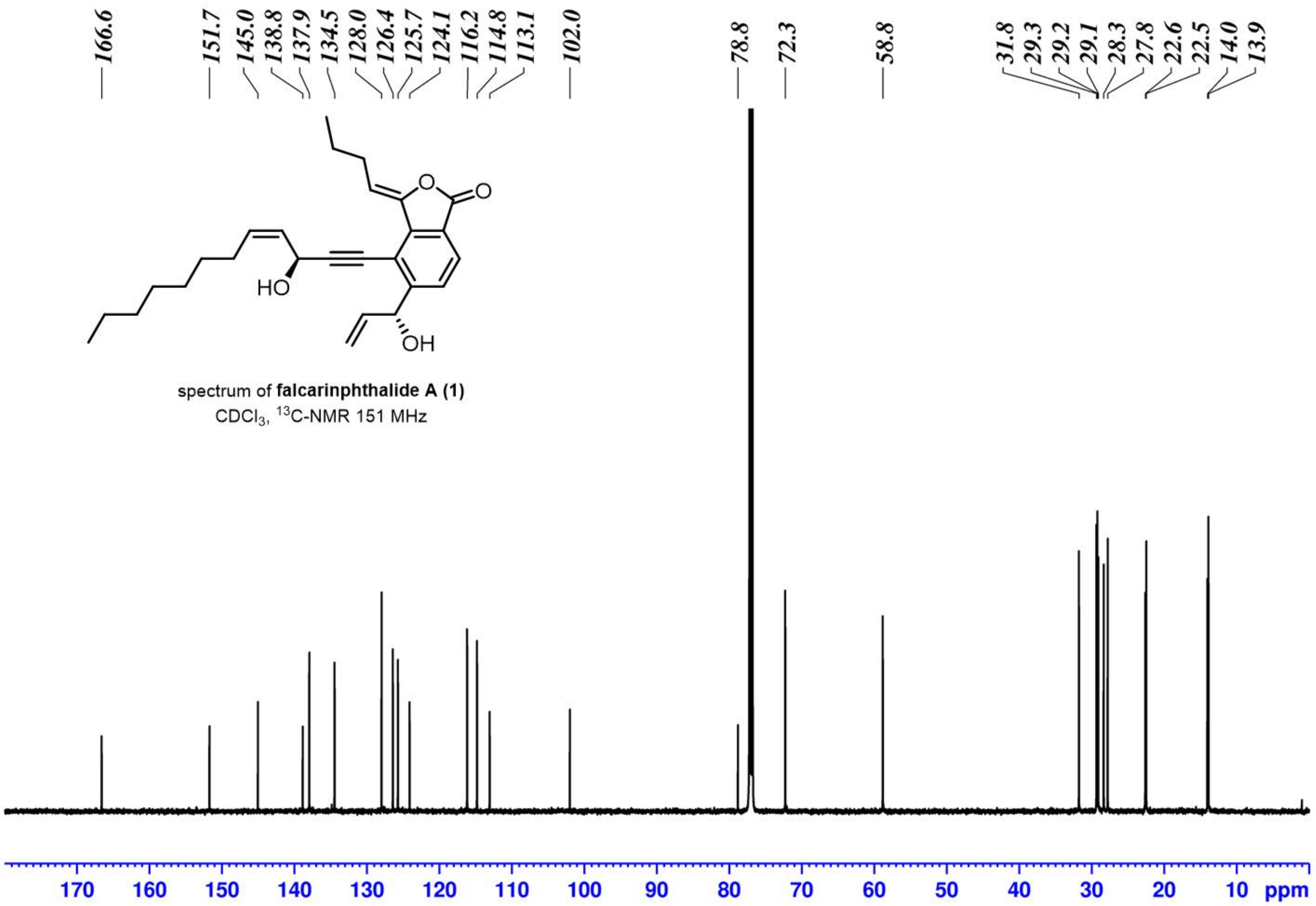

Figure S67. ^{13}C NMR (151 MHz, $CDCl_3$) of falcarinphthalide A (**1**).
